# Therapeutic Targets for Lung Squamous Cell Carcinoma: Proteome‐Wide Mendelian Randomization and Potential Drug Prediction

**DOI:** 10.1111/crj.70182

**Published:** 2026-03-17

**Authors:** Tao Xiang, Tingting Hu, Jiantong Sun, Qikun Geng, Jiazun Yang, Zhongyu Jian

**Affiliations:** ^1^ West China Hospital Sichuan University Chengdu China; ^2^ Institute of Thoracic Oncology and Department of Thoracic Surgery, West China Hospital Sichuan University Chengdu China; ^3^ Department of Urology, Institute of Urology (Laboratory of Reconstructive Urology) West China Hospital, Sichuan University Chengdu China

**Keywords:** lung squamous carcinoma, Mendelian randomization, plasma protein, survival analysis

## Abstract

**Background:**

Lung squamous cell carcinoma (LUSC) is one of the most prevalent subtypes of lung cancer, accounting for approximately 25%. Mendelian randomization (MR) analysis of plasma proteins can identify potential drug targets for LUSC.

**Methods:**

We obtained genetic instruments for plasma proteins from a GWAS by Sun BB et al. conducted on 3301 healthy adults. GWAS summary statistics of LUSC were from ILCCO. We conducted a two‐sample MR analysis to investigate the causal relationships between plasma proteins and the risk to LUSC. Colocalization analysis was used to test whether two traits share the same causal variables. Survival analysis based on potential proteins was conducted using transcriptomic data from TCGA database to determine whether gene expression levels are associated with the prognosis of LUSC patients. Moreover, predictions of potential drug ligands and molecular dockings were performed to evaluate the pharmaceutical properties of the target genes.

**Results:**

Proteome‐wide MR analysis identified three proteins (MICB, SPINK2, TXNDC11) that showed a strong association with decreased risk of LUSC after FDR correction. Increased levels of MICB [OR(95% CI) = 0.72(0.63, 0.83); *p* = 3.90E−06], SPINK2 [OR (95% CI) = 0.74 (0.66, 0.84); *p* = 1.25E−06], TXNDC11 [OR (95% CI) = 0.63(0.51, 0.78); *p* = 2.69E−05] per SD decreased the risk of LUSC specifically. Sensitivity analysis revealed that there was no significant heterogeneity, pleiotropy, or reverse causality between these proteins and LUSC. Colocalization analysis indicated that three proteins shared the same variants with LUSC. Survival analysis showed that upregulation of SPINK2 was associated with a favorable prognosis in LUSC patients, while MICB and TXNDC11 were not. The expression of MICB, SPINK2, TXNDC11 was found to be potentially affected by specific substances, and our molecular docking showed a stable binding between SPINK2 and danazol, suggesting its potential as a drug target.

**Conclusion:**

This study identified SPINK2 causally associated with the risk and prognosis of LUSC, which is a promising target for LUSC. The drug prediction we performed illustrated the medicinal potential of SPINK2, and the high binding activity of molecular docking indicated its strong potential as a drug target.

## Graphical abstract

This flowchart shows the main contents of our study, including main analysis, sensitivity analysis, and survival analysis. The figure briefly shows the source of our data, as well as some other screening requirements. The main finding of mendelian randomization was that three of the plasma proteins were shown to be associated with the risk of LUSC.

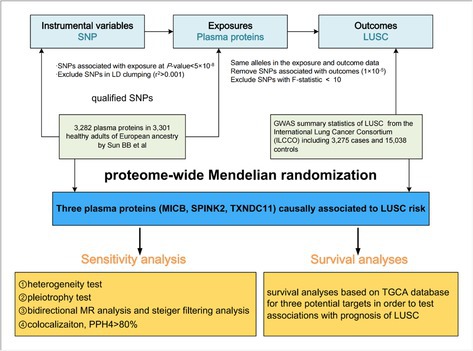

## 1 Introduction

Lung cancer (LC) is a primary malignant tumor of the lung originating from the mucosa or glands of the trachea and bronchus. It is estimated that the United States will experience 238 340 new cases of LC and 127 070 cancer‐related deaths in the year 2023 [1]. Lung squamous cell carcinoma (LUSC) is one of the most prevalent subtypes, accounting for approximately 25% of all LC cases [2, 3]. Despite the high prevalence of LUSC, available treatments for it are unsatisfactory, since which LUSC has a poor prognosis. More than half of patients diagnosed with LUSC die within a year after diagnosis, and the survival rate after 5 years is below 20% [3].

In recent years, remarkable technological advancements, particularly in the field of genome‐wide association studies (GWASs), have resulted in the discovery of numerous novel biomarkers [4, 5, 6]. These GWASs show promise in revolutionizing the diagnosis, treatment, and prognosis of LC. Nevertheless, it remains crucial to identify more promising drug targets to facilitate the development of targeted therapies for LUSC, thereby improving the prognosis of patients with LUSC.

Human plasma proteins play important roles in the occurrence of various cancers through intricate pathophysiological mechanisms [7, 8]. Exploring the associations between proteins and cancer can provide invaluable insights into the complexities of cancer progression and ultimately pave the way for therapeutic approaches. Previous GWAS studies have identified genetic variants associated with thousands of plasma proteins [9] and LUSC [10]. Therefore, we can explore potential drug targets for LUSC through Mendelian randomization (MR) analysis on plasma proteins.

MR is a data analysis method for assessing etiological inferences in epidemiological studies that uses genetic variants with strong correlations with exposure factors as instrumental variables to assess causal association between exposure factors and outcomes [11]. In our study, a two‐sample MR was applied to investigate the causal effects of up to 3282 human plasma proteins on LUSC.

**FIGURE 1:**
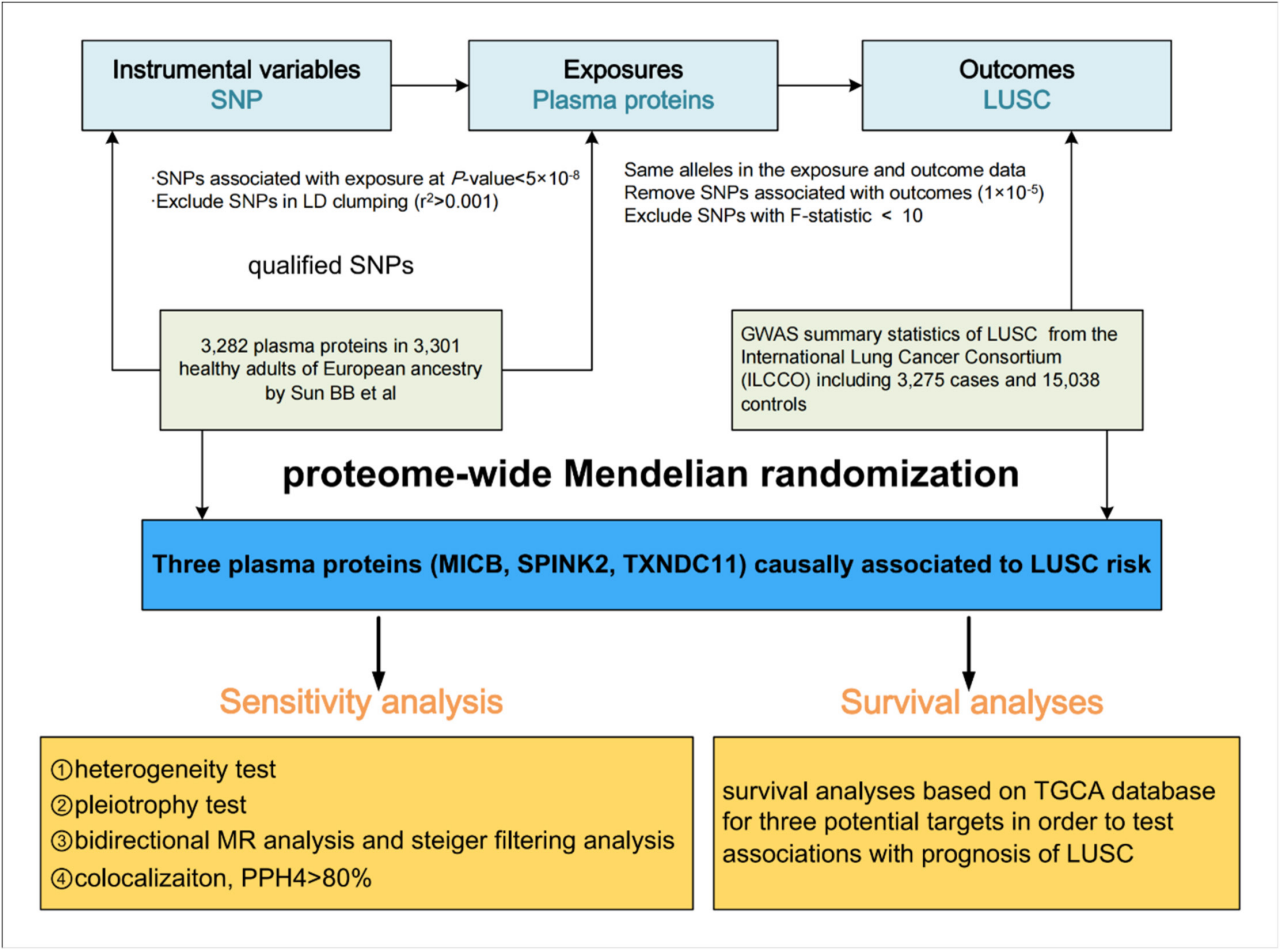
Graphical table of contents.

This study aimed at searching for potential drug targets for lung LUSC. Figure 1 is the graphical table of contents.

## 2 Materials and Methods

### 2.1 Proteomic Data Sources

In this study, we obtained genetic instruments for plasma proteins from a GWAS by Sun BB et al. conducted on 3301 healthy adults of European ancestry [9]. This large GWAS study provides data on up to 3282 plasma proteins, and all of them are available on IEU (IEU OpenGWAS project (mrcieu.ac.uk)).

### 2.2 Outcome Data Sources

GWAS summary statistics from the International Lung Cancer Consortium (ILCCO) of LUSC including 3275 cases and 15 038 controls is available on IEU. All participants involved are of European ancestry. LUSC cases were diagnosed according to ICD‐O‐3: 8070/3, 8071/3, 8072/3, and 8074/3 [10]. The sample of exposure data was all British, while the outcome sample included Americans, Finns, British, and French. The proportion of British in the outcome is only 7%, so the maximum sample between exposure and outcome overlap was 7%.

### 2.3 MR Analysis

We conducted a two‐sample MR analysis using index SNPs for proteins to investigate the associations between proteomic proteins and the risk of LUSC. All relevant SNPs met the significance threshold of *p* ≤ 5 × 10^−8^. Independent SNPs were identified by linkage disequilibrium (LD) clumping (*r* ^*2*^ < 0.001 within 10 Mb). In cases where only one SNP was available for a specific protein, we utilized the Wald ratio method. When there were two or more genetic instruments available, we employed the inverse variance weighted (IVW) method. The value of the ratio (OR) expresses the strength of the causal relationship between the exposure factor and the outcome factor, which indicates increased LUSC risk per standard deviation (SD) increase in plasma protein levels.

Heterogeneity analysis and pleiotropy analysis were conducted with the aim of examining whether there is any bias in MR analysis. For multiple testing, false discovery rate (FDR) correction method was applied to prioritize the MR results (*p* values after correction < 0.05) for further analysis.

All statistical procedures and plots were implemented on R (version 4.3.1). The MR main analysis used the TwosampleMR package (version 0.5.7). The MR study we did was strictly based on the STROBE‐MR checklist [12].

### 2.4 Reverse Causality Detection

For proteins that presented a strong association with LUSC after the FDR correction, we used bidirectional MR analysis and Steiger filtering analysis to exclude the possibility of reverse causality. In bidirectional MR analysis, genetic instruments for LUSC were extracted from the GWAS of ILCCO [10] and the selection followed the identical screening criteria for plasma proteins [13]. Complete summary statistics for proteins were obtained from the same GWAS from Sun BB et al. [9] We used the IVW method to estimate the effect of reverse causality. We also conducted Steiger filtering analysis to ensure the directionality of the causal association between proteins and LUSC. Reverse causation was considered statistically significant only if *p* < 0.05.

### 2.5 Colocalization Analysis

Colocalization analysis is used to test the possibility that two traits share the same causal variables. If two traits are colocalized, they are likely to be inherited at the same time and thus highly correlated [14]. Bayesian co‐localization proposes five hypotheses (H0, H1, H2, H3, H4) whether two traits share a single genetic variant. Our study aimed to test the posterior probability of hypothesis 4 (PPH4) through Bayesian co‐localization analysis. Two traits were considered to have a strong association within a certain region by shared variants if PPH_4_ was ≥ 0.8.

### 2.6 Survival Analysis Based on Potential Proteins

Theoretically, potential targets should have an impact on the prognosis of LUSC patients. We extracted transcriptomic data of potential targets based on the TCGA database (The Cancer Genome Atlas Program (TCGA)‐NCI) to indicate the gene expression level. Survival data of LUSC patients were also obtained from the TCGA database to correlate with the transcriptome data. We utilized the Kaplan–Meier estimator to compare the overall survival (OS) time between cancer patients with higher gene expression (Fragments Per Kilobase per Million (FPKM) > median) and those with lower gene expression (FPKM < median) [15]. A significant difference between the OS times of the two groups was considered to exist if the log‐rank test *p* < 0.05.

### 2.7 Potential Drug Prediction and Molecular Docking

We acquired spatial structural information of proteins from the PDB database (http://www.rcsb.org/) and the Alphafold database of EMBL‐EBI (https://www.ebi.ac.uk/). Drug–Gene Interaction Database (DGIdb) 4.0 (https://dgidb.genome.wustl.edu/) was used to predict the ligand drugs of two target proteins. The required drug structure data were obtained from the PubChem compound database (https://pubchem.ncbi.nlm.nih.gov/).

To enhance the comprehension of the impact of potential drugs on their target genes and the medicinal characteristics of these genes, this research utilized molecular docking techniques at the atomic level to assess the binding affinity and mode of interaction between the candidate drugs and their targets. Through molecular docking simulations, we can conduct a detailed analysis of the affinity and interaction pattern between ligands and their targets. After identifying ligands with high binding affinity and superior interaction patterns, the corresponding target sites can be prioritized for further experimental validation and optimization of candidate drug designs. In this study, AutodockVina 1.2.2 (http://autodock.scripps.edu/) was used to perform molecular docking studies on the proteins encoded by two key target genes and their corresponding ligand drugs. Initially, PyMOL 2.0 (https://pymol.org/) was used to all water molecules from the protein and ligand files and adding polar hydrogen atoms. Then, binding sites were identified using PyMOL's GetBox Plugin. After preprocessing the three‐dimensional structures of proteins and corresponding drugs using the AutodockVina software, a semiflexible docking strategy was employed for the docking of the two sets of target proteins with drug molecules. Subsequently, the lowest binding energy pairing was selected using the PyMOL software, and its molecular interaction diagram and three‐dimensional structure was depicted. Furthermore, Molecular Operating Environment (MOE) 2022 (https://www.chemcomp.com/) was used to predict the interaction details of their secondary structures.

## 3 Results

### 3.1 Proteome‐Wide MR Analysis

**FIGURE 2:**
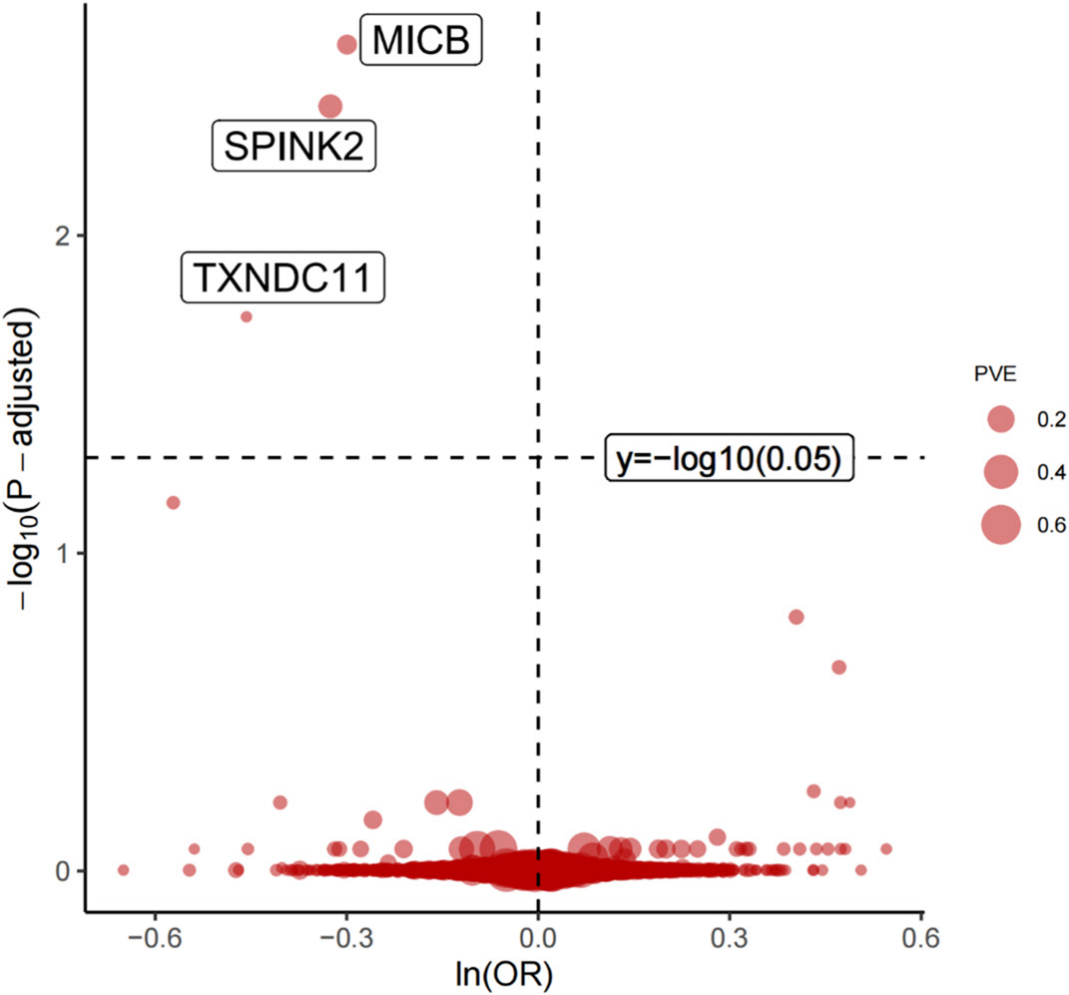
Volcano plot of MR results. The volcano plot illustrates the MR findings on the association between 2014 plasma proteins and the risk of LUSC. Odds ratios (OR) indicates increased LUSC risk per standard deviation (SD) increase in plasma protein levels. ln, natural logarithm; P‐adjusted, *p* values after FDR correction; PVE, proportion of variance explained.

A total of 3875 SNPs from 2014 plasma proteins' GWASs were included in the MR analysis, while other proteins were excluded due to lack of qualified SNPs. Finally, MR analysis reveals three proteins that still show strong association with LUSC after FDR correction, including MHC class I polypeptide‐related sequence B (MICB), serine protease inhibitor Kazal‐type 2 (SPINK2), and thioredoxin domain‐containing protein 11 (TXNDC11) (Figure 2; Table 1). All three proteins are protective factors, and increased MICB [OR (95% CI) = 0.72 (0.63, 0.83); *p* = 3.90E−06], SPINK2 [OR (95% CI) = 0.74 (0.66, 0.84); *p* = 1.25E−06], and TXNDC11 [OR (95% CI) = 0.63 (0.51, 0.78); *p* = 2.69E−05] decreased the risk of LUSC specifically.

**TABLE 1:** Three proteins show strong association with LUSC after FDR correction.

Protein	Method	SNP	Effect allele	*F* statistics	OR (95% CI)^a^	*p*s	P‐adjusted	PVE	Author
MICB	IVW	rs1611236	A	36.32	0.72 (0.63, 0.83)	3.90E−06	3.92E−03	13.24%	Sun et al.
		rs3134900	G	456.52					
SPINK2	IVW	rs11941335	T	145.04	0.74 (0.66, 0.84)	1.25E−06	2.51E−03	6.86%	Sun et al.
		rs1687391	T	49.87					
		rs41363847	T	38.95					
TXNDC11	Wald ratio	rs114561324	A	30.66	0.63 (0.51, 0.78)	2.69E−05	1.80E−02	0.92%	Sun et al.

Abbreviations: IVW, inverse variance weighted; PVE, proportion of variance explained.

^a^ Odds ratios (OR) indicate increased LUSC risk per standard deviation (SD) increase in plasma protein levels.

The SNPs used in the two‐sample MR analysis of the three proteins all possessed *F*‐values larger than 10, which further proved the credibility of their causal relationship (Table 1) [16]. The extent of the effect of the three proteins on the increased risk of LUSC was expressed as proportion of variance explained (PVE), with MICB accounting for the most at 13.24%, while SPINK2 and TXNDC11 were 6.86% and 0.92%, respectively (Table 1).

### 3.2 Sensitivity Analysis for LUSC Causal Proteins

**FIGURE 3:**
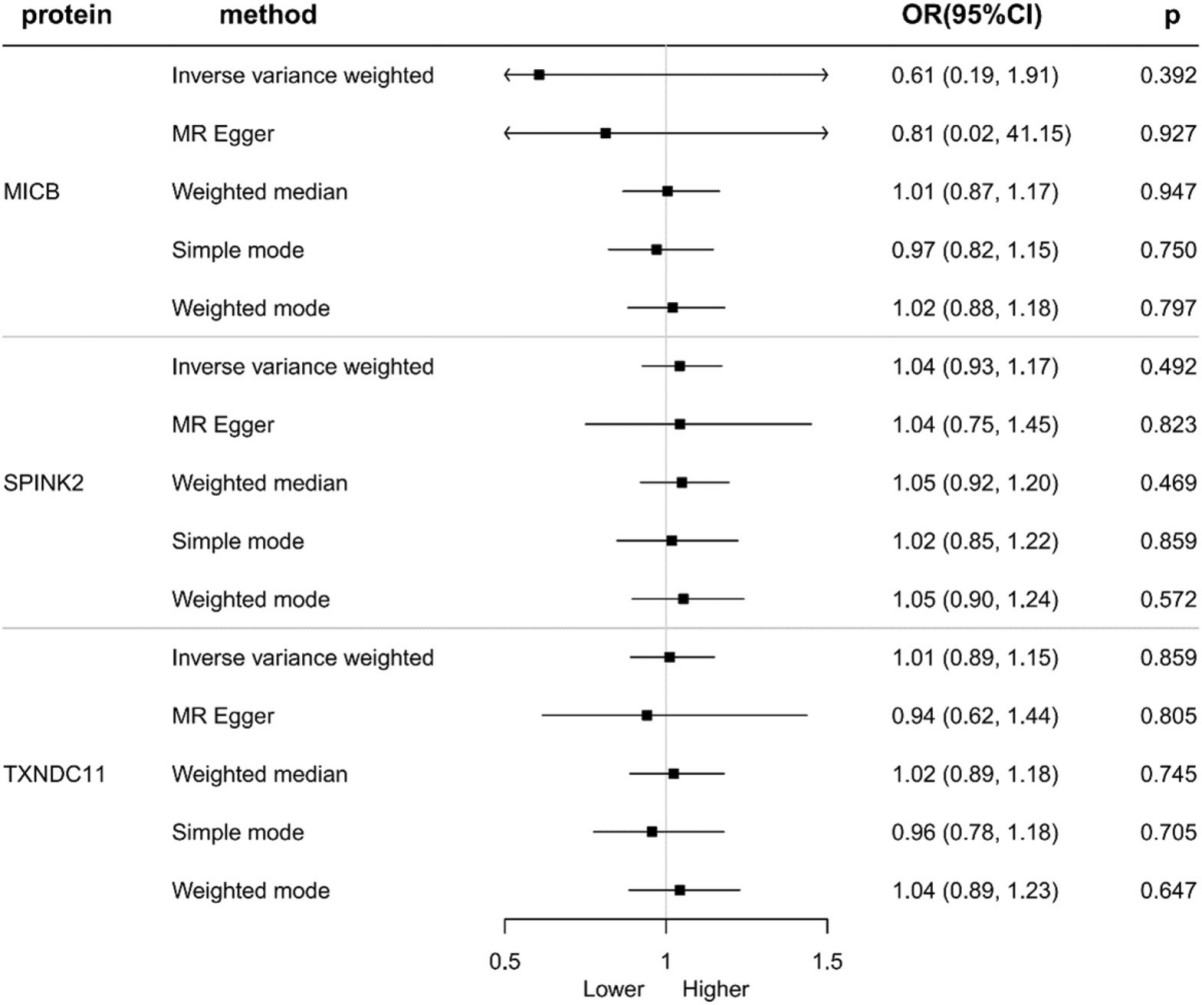
Bidirectional MR analysis for LUSC on levels of three potential causal proteins. OR stood for the odds ratios for per standard deviation (SD) increase in plasma protein levels as LUSC risk increased.

**FIGURE 4:**
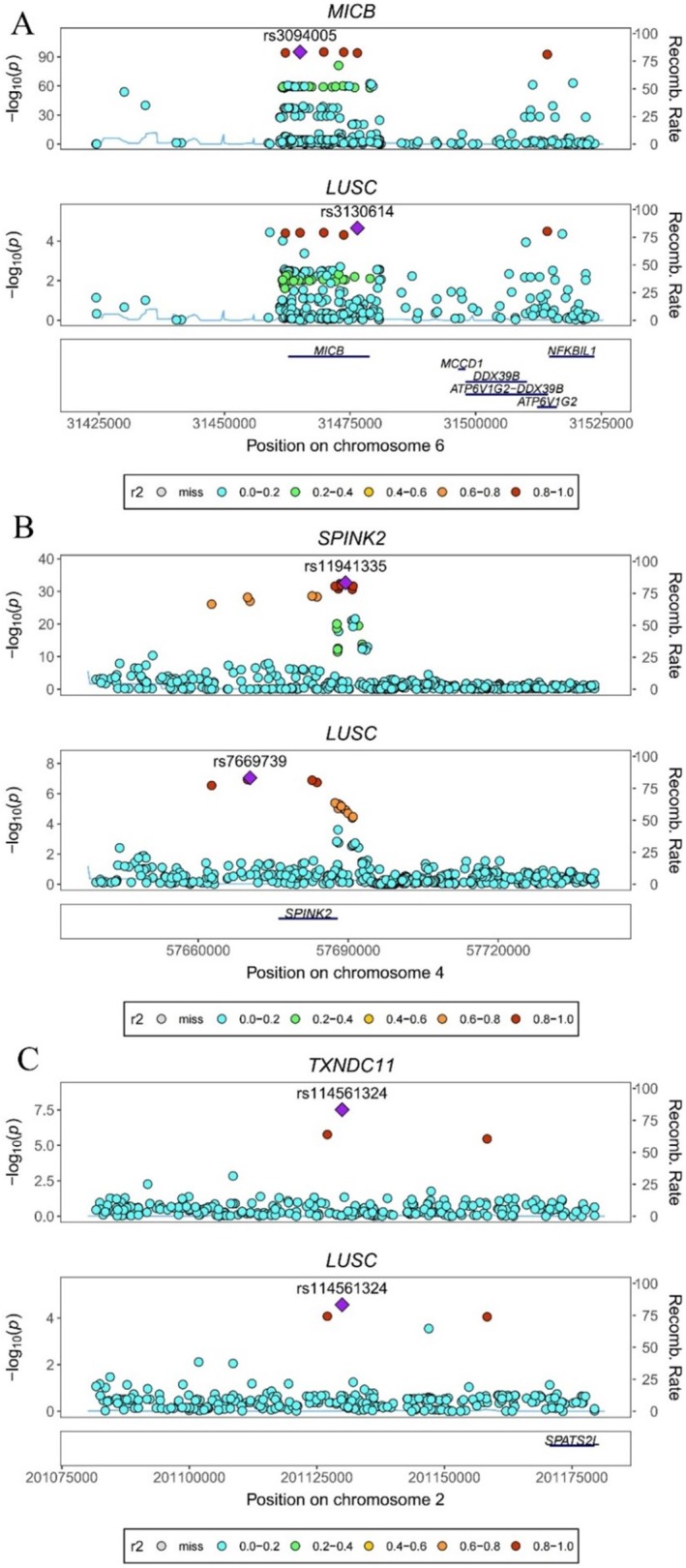
Colocalization between three proteins and LUSC. (A) Colocalization analysis between MICB and LUSC; (B) colocalization analysis between SPINK2 and LUSC; (C) colocalization analysis between TXNDC11 and LUSC.

First, no heterogeneity and pleiotropy were found in three proteins. Second, bidirectional MR analysis revealed no statistically significant reverse causality between LUSC and three proteins (Table 2; Figure 3), and Steiger filtering analysis ensured directionality furthermore (Table 2). Third, colocalization analysis revealed that all three proteins share the same genetic variants with LUSC, indicating a strong genetic link between them in terms of genetics. More specifically, the coloc.abf‐PPH4 values for MICB, SPINK2, and TXNDC11 are 97.70%, 83.20%, and 89.6%, respectively (Figure 4; Table 2).

**TABLE 2:** Three proteins show strong association with LUSC after FDR correction.

Protein	snp	Steiger filtering	Pleiotropy test	Heterogeneity test (IVW)	Bidirectional MR (MR‐IVW)	Colocalization PPH_4_ (coloc.abf)
MICB	rs1611236	1.43E−06	NA^a^	0.85	0.61 (−0.54, 1.75)	97.70%
	rs3134900	3.45E−69				
SPINK2	rs11941335	1.44E−20	0.78	0.83	1.04 (0.92, 1.16)	83.20%
	rs1687391	1.06E−08				
	rs41363847	8.92E−08				
TXNDC11	rs114561324	5.64E−04	NA^a^	NA^b^	1.01 (0.88, 1.14)	89.6%

Abbreviations: IVW, inverse variance weighted method; NA, not available; PP, posterior probability; SNP, single nucleotide polymorphism.

^a^ Not enough SNPs are available for pleiotropy analysis.

^b^ Not enough SNPs available for heterogeneity analysis.

### 3.3 Survival Analysis

**FIGURE 5:**
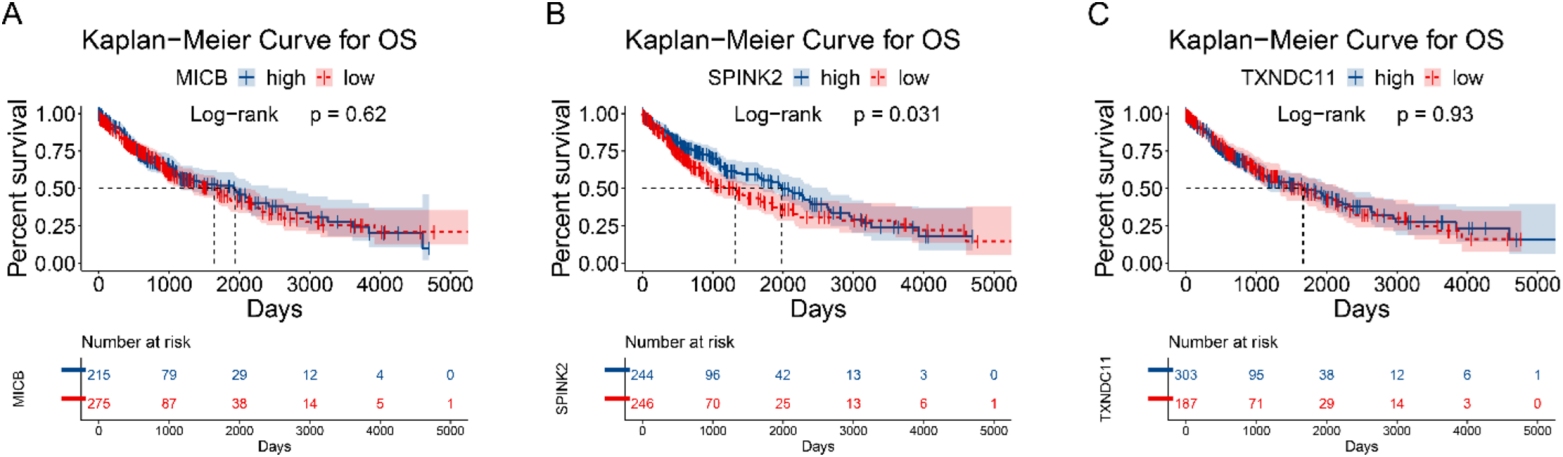
Kaplan–Meier curves for three proteins. (A) Survival analysis for MICB; (B) survival analysis for SPINK2; (C) survival analysis for TXNDC11.

We performed survival analysis to evaluate the influence of three potential targets on prognosis of LUSC patients, which revealed only upregulation of SPINK2 was associated with a favorable patient prognosis (*p* = 0.031). And the expression of MICB (*p* = 0.62) and TXNDC11 (*p* = 0.93) was not associated with OS time in LUSC patients (Figure 5).

### 3.4 Potential Drug Prediction and Molecular Docking

Drug–gene interactions of MICB, TXNDC11, and SPINK2 were searched in DGIdb 4.0. Danazol was found to be potentially associated with the upregulation of SPINK2, and vorinostat with MICB, while no drugs were found to directly interact with TXNDC11.

**FIGURE 6:**
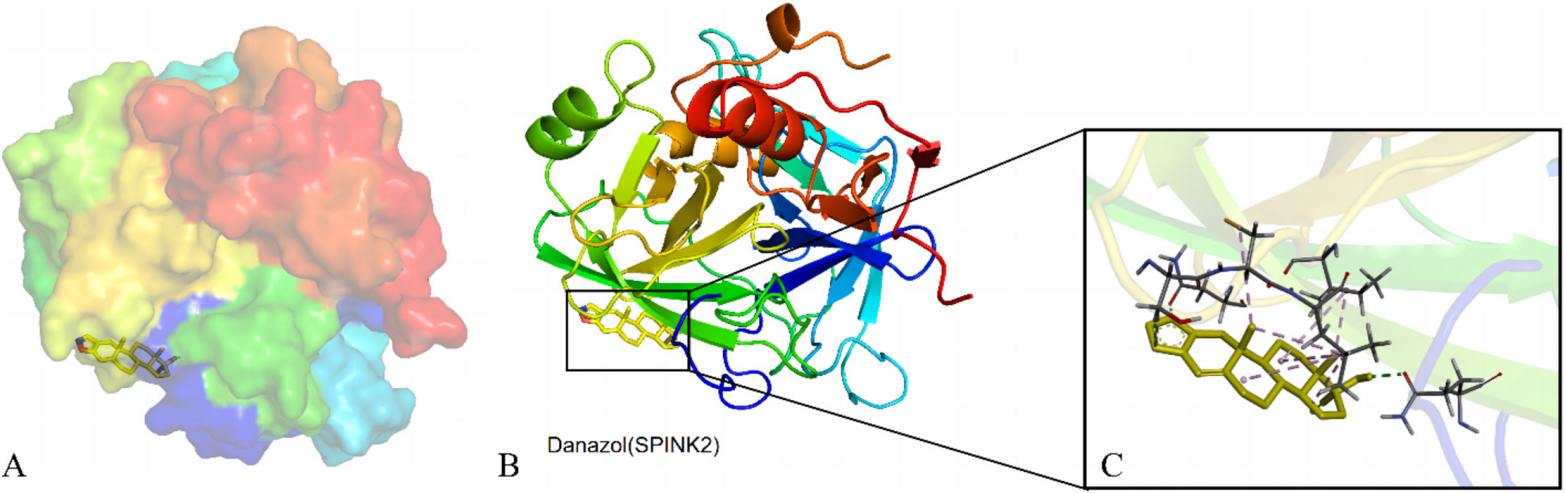
Molecular docking simulation for SPINK2 and danazol. (A) Overall binding position of danazol (stick) on SPINK2 (surface shown). (B) Overall binding position of danazol (stick) on SPINK2 (cartoon). (C) Interaction of danazol (yellow) with related residues (grey) on SPINK2.

**FIGURE 7:**
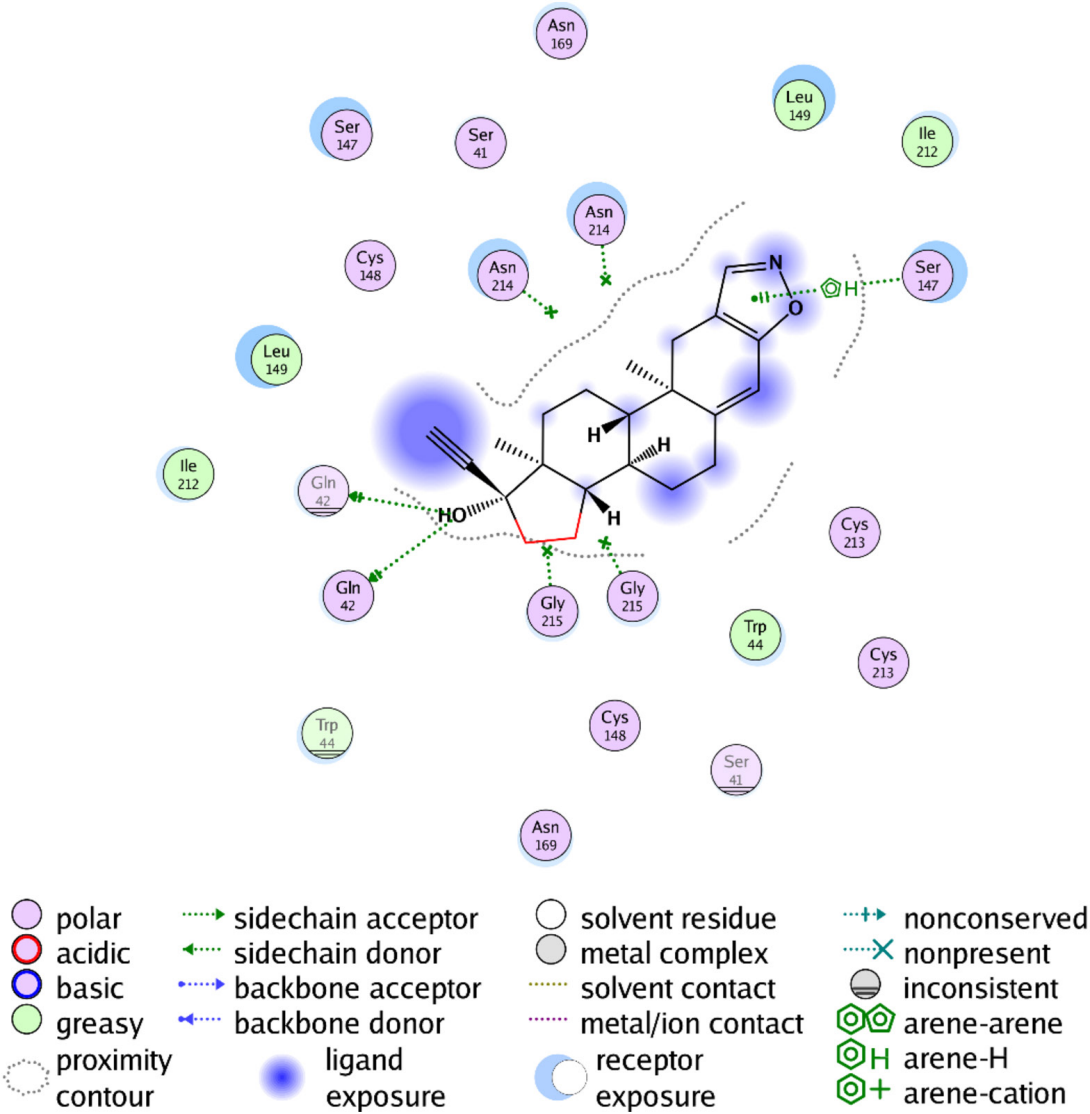
Two‐dimensional plan for observing the hydrogen bonding and hydrophobic interactions between danazol and SPINK2.

To investigate the binding affinity of two discovered potential drugs to the proteins discovered and subsequently assess their potential pharmaceutical properties and the drug‐target suitability for clinical development, this study employed molecular docking techniques. Utilizing the Autodock Vina v.1.2.2 software, the binding sites and interaction mechanisms of candidate drugs with specific proteins were successfully predicted, with the binding for each interaction mode being calculated. After effectively docking the two proteins with corresponding drugs, the data (Table 3; Figures 6 and 7) were obtained. The results indicate that both candidate drugs tightly bind to their respective protein targets through hydrogen bonding and significant electrostatic interactions, successfully occupying the binding pocket of the target protein. Notably, the lowest binding energy between SPINK2 and danazol reached −6.46 kcal/mol, indicating a stable binding, while that between MICB and vorinostat was higher than −5 kcal/mol, indicating that vorinostat was not an appropriate ligand for MICB.

**TABLE 3:** Molecular dockings of two protein targets with corresponding drugs.

Protein	Ligand	Lowest binding energy	Mean binding energy
SPINK2	Danazol	−6.46 kcal/mol	−6.43 kcal/mol
MICB	Vorinostat	−5.72 kcal/mol	−5.22 kcal/mol

## 4 Discussion

This study represents the first attempt to explore potential targets for LUSC based on plasma proteomic data utilizing MR analysis. We obtained instrumental variables from the currently largest and most comprehensive GWAS sources on plasma proteins, which contain up to 3282 human plasma proteins. MR analysis identified three proteins causally associated with LUSC, and all of them were protective factors, including MICB, SPINK2, and TXNDC11. Colocalization analyses for three proteins further confirmed the causal relationship. We also excluded the possibility of reverse causation by Steiger filtering analysis and bidirectional MR analysis. Survival analyses demonstrated that SPINK2 is associated with the prognosis of LUSC patients, and we consider it a promising target for LUSC, while MICB and TXNDC11 were considered potential targets.

MICB refers to a protein that belongs to the major histocompatibility complex (MHC) class I family [17]. Particularly, unlike classical MHC‐I molecules, MICB molecules are usually not expressed in normal tissue cells but only on the surface of those cells infected by pathogens or malignant proliferating tumor cells [18]. MICB serves as a ligand for the activating receptor known as NKG2D, which is found on activated immune cells. Once MICB binds to NKG2D, it triggers signaling pathways that enhance diverse antitumor innate NK cells and antigen‐specific T cells [19]. A previous clinical study showed that upregulation of MICB was associated with a favorable prognosis in non‐small‐cell LC [20]. However, our survival analysis study did not reveal the prognostic impact of MICB on LUSC, probably because patients in the TCGA database generally had low MICB expression.

SPINK2 is a type of protein encoded by human genes, also known as serine peptidase inhibitor, Kazal Type 2. It belongs to the Kazal family, which includes several proteins with similar structures and functions [21]. SPINK2 refers to a serine protease inhibitor, regulating the function of serine protease, which plays an important role in various biological processes, including digestion, immune response, and tissue repair [21, 22]. Therefore, the function of SPINK2 protein is to maintain the balance of serine protease activity, ensuring that it functions within the normal range. There are very few previous studies about SPINK2, and only several studies have reported that SPINK2 is associated with a poorer prognosis in acute myeloid leukemia [23] and associated with male infertility [24]. Therefore, the mechanism of how SPINK2 acts on LUSC occurrence and prognosis is not clear. However, after the validation of MR analysis and colocalization analysis in this study, as well as the survival analysis demonstrating that SPINK2 has an effect on the increase of OS time in LUSC patients, we identified SPINK2 as a potential target for LUSC. Our molecular docking revealed that danazol, a steroid heterocyclic compound used in the treatment of endometriosis [25], hereditary angioedema [26], and myelodysplastic syndromes [27], showed good affinity with SPINK2, indicating its potentiality as a drug target for the treatment of tumor.

TXNDC11 is a protein containing five thioredoxin (TXN)‐like domains expressed in the cellular endoplasmic reticulum (ER) [28]. TXN is an essential factor involved in maintaining redox homeostasis and plays a critical role in the cellular response to oxidative stress. Previously, a bioinformatics analysis has demonstrated an association between TXN and the development and prognosis of LC [29]. Previous studies have shown that the TXN‐like domains of TXNDC11 make it a reductase, and therefore, TXNDC11 plays an important role in the degradation of misfolded proteins synthesized in the ER [30]. It is known that the development of LUSC is associated with a series of cellular dysfunctions caused by ER stress [31]. As a result, we hypothesized that TXNDC11 may have a role in alleviating ER stress in the process of ER stress in bronchial epithelial cells and thus be causally related to the development of LUSC. In our study, MR analysis revealed that TXNDC11 is a protective factor for LUSC, which is consistent with previous studies.

However, several limitations should also be considered in our study. First, in order to ensure strong correlation, we only extracted SNPs from the plasma protein GWAS data that met the significance threshold of *p* ≤ 5 × 10–8, and thus, 1268 plasma proteins were excluded from the study due to the lack of suitable SNPs. Second, our study only focused on populations of European ancestry, and therefore may not be well generalizable to populations of all ancestries. Thirdly, in survival analysis, MICB and TXNDC11 did not show an effect on prognosis in LUSC as we expected, possibly due to the fact that samples in TGCA had generally low expression for both genes. Fourth, due to the current lack of research on SPINK2 and the whole SPINK family, we were unable to have a more thorough discussion on how SPINK2 is involved in lung carcinogenesis.

## 5 Conclusions

This study identified SPINK2 causally associated with the risk and prognosis of LUSC, which is a promising target for LUSC. The drug prediction we performed illustrated the medicinal potential of SPINK2, and the high binding activity of molecular docking indicated its strong potential as a drug target.

## Author Contributions

Zhongyu Jian conceived this research. Tao Xiang and Zhongyu Jian extracted the GWAS data and performed two‐sample MR analysis and sensitivity analysis, Tingting Hu performed the survival analysis, and Qikun Geng prepared the figures. Tao Xiang and Jiantong Sun wrote the first version of our manuscript, and other authors helped revise it. All authors have reviewed and approved the final manuscript. The work reported in the paper has been performed by the authors, unless clearly specified in the text.

## Funding

The authors have nothing to report.

## Ethics Statement

The authors declare that there are no ethical issues involved in this study.

## Conflicts of Interest

The authors declare no conflicts of interest.

## Supporting information


**TABLE S1:** Genetic instruments of plasma proteins for MR.
**TABLE S2:** Genetic instruments of lung squamous carcinoma for bidirectional MR.
**TABLE S3:** Heterogeneity analysis on plasma proteins with two or more instruments.
**TABLE S4:** STROBE‐MR checklist of recommended items to address in reports of Mendelian randomization studies1,2.

## Data Availability

GWAS summary statistics for both plasma proteins and LUSC can be downloaded from IEU (IEU OpenGWAS project (mrcieu.ac.uk)). Transcriptomic data and survival data of LUSC patients are available on TCGA (https://www.cancer.gov/ccg/research/genome‐sequencing/tcga). For further information about this study, you can contact the corresponding author by email. The other data generated or analyzed during this study are available in the supporting information files.
