# Supplementary material for: Therapeutic Targets for Lung Squamous Cell Carcinoma: Proteome‐Wide Mendelian Randomization and Potential Drug Prediction
Source: Clin Respir J. 2026 Mar 17;20(3):e70182. doi: 10.1111/crj.70182 (PMC13093309; doi:10.1111/crj.70182)
Supplement: Supplementary file 1 — TABLE S1: Genetic instruments of plasma proteins for MR. TABLE S2: Genetic instruments of lung squamous carcinoma for bidirectional MR. TABLE S3: Heterogeneity analysis on plasma proteins with two or more instruments. TABLE S4: STROBE‐MR checklist of recommended items to address in reports of Mendelian randomization studies1,2. [file CRJ-20-e70182-s001.docx]

**Therapeutic targets for lung squamous cell carcinoma: proteome-wide Mendelian randomization and potential drug prediction**

**Molecular Genetics & Genomic Medicine**

**Author Names**

Tao Xiang^1^, Tingting Hu^1^, Jiantong Sun^2^, Qikun Geng^1^, Jiazun Yang^1^, Zhongyu Jian^3*^,

*Correspondence: Zhongyu Jian

West China Hospital, Sichuan University, No. 37 Guo Xue Xiang, Chengdu 610041, Sichuan, China

E-mail: [zhongyu_jian@163.com](mailto:zhongyu_jian@163.com)

Telephone number：13551398795

ORCID：0000000285329336

| **Supplementary table1： Genetic instruments of plasma proteins for MR** | | | | | | | | | | | |
| --- | --- | --- | --- | --- | --- | --- | --- | --- | --- | --- | --- |
| **exposure** | **SNP** | **effect_allele** | **other_allele** | **chr** | **pos** | **beta** | **pval** | **se** | **eaf** | **t_stat** | **f_stat** |
| [Pyruvate dehydrogenase (acetyl-transferring)] kinase isozyme 1, mitochondrial || id:prot-a-2235 | rs704 | A | G | 17 | 26694861 | 0.4579 | 1.86E-85 | 0.0234 | 0.46665 | 19.56837607 | 382.921342 |
| 1-phosphatidylinositol 4,5-bisphosphate phosphodiesterase gamma-1 || id:prot-a-2296 | rs4632248 | T | G | 19 | 54324995 | 0.3337 | 1.02E-29 | 0.0295 | 0.21439 | 11.31186441 | 127.9582764 |
| 1-phosphatidylinositol 4,5-bisphosphate phosphodiesterase gamma-1 || id:prot-a-2296 | rs2076146 | C | G | 20 | 39798418 | 0.1551 | 3.16E-10 | 0.0247 | 0.47698 | 6.279352227 | 39.43026439 |
| 14-3-3 protein family || id:prot-a-3248 | rs62143197 | A | G | 19 | 54320716 | 0.4752 | 5.01E-59 | 0.0293 | 0.22311 | 16.21843003 | 263.0374728 |
| 14-3-3 protein sigma || id:prot-a-2708 | rs4241819 | T | C | 4 | 187157140 | -0.1427 | 8.32E-09 | 0.0248 | 0.50721 | -5.754032258 | 33.10888723 |
| 14-3-3 protein sigma || id:prot-a-2708 | rs142694121 | T | C | 8 | 135146293 | -0.4224 | 4.37E-08 | 0.0771 | 0.03338 | -5.478599222 | 30.01504943 |
| 14-3-3 protein zeta/delta || id:prot-a-3251 | rs62143197 | A | G | 19 | 54320716 | 0.2701 | 2.95E-19 | 0.0301 | 0.22311 | 8.973421927 | 80.52230108 |
| 15-hydroxyprostaglandin dehydrogenase [NAD(+)] || id:prot-a-1370 | rs17580 | A | T | 14 | 94847262 | 0.7138 | 1.26E-37 | 0.0557 | 0.04977 | 12.81508079 | 164.2262957 |
| 15-hydroxyprostaglandin dehydrogenase [NAD(+)] || id:prot-a-1370 | rs62143198 | A | G | 19 | 54320939 | 0.1884 | 7.08E-10 | 0.0306 | 0.21453 | 6.156862745 | 37.90695886 |
| 17-beta-hydroxysteroid dehydrogenase 14 || id:prot-a-1381 | rs3130047 | T | C | 6 | 31610479 | -0.1921 | 4.47E-10 | 0.0308 | 0.19955 | -6.237012987 | 38.900331 |
| 2'-5'-oligoadenylate synthase 1 || id:prot-a-2136 | rs4767027 | C | T | 12 | 113359157 | -0.2701 | 6.17E-26 | 0.0256 | 0.65384 | -10.55078125 | 111.318985 |
| 2'-5'-oligoadenylate synthase 1 || id:prot-a-2136 | rs62143197 | A | G | 19 | 54320716 | 0.2819 | 7.08E-21 | 0.0301 | 0.22311 | 9.365448505 | 87.7116257 |
| 2-phosphoxylose phosphatase 1 || id:prot-a-21 | rs9274306 | G | T | 6 | 32632006 | 0.1863 | 4.47E-11 | 0.0283 | 0.57111 | 6.583038869 | 43.33640075 |
| 2-phosphoxylose phosphatase 1 || id:prot-a-21 | rs704 | A | G | 17 | 26694861 | 0.3583 | 7.76E-51 | 0.0239 | 0.46665 | 14.9916318 | 224.749024 |
| 26S proteasome non-ATPase regulatory subunit 1 || id:prot-a-2416 | rs191696335 | A | T | 1 | 215012155 | -0.2694 | 4.27E-08 | 0.0492 | 0.07901 | -5.475609756 | 29.9823022 |
| 26S proteasome non-ATPase regulatory subunit 5 || id:prot-a-2418 | rs56401446 | G | A | 14 | 106445681 | -0.2294 | 6.17E-18 | 0.0266 | 0.61852 | -8.62406015 | 74.37441348 |
| 3-hydroxyanthranilate 3,4-dioxygenase || id:prot-a-1306 | rs6809081 | C | T | 3 | 194060475 | 0.712 | 1.00E-200 | 0.0224 | 0.42319 | 31.78571429 | 1010.331633 |
| 3-hydroxyanthranilate 3,4-dioxygenase || id:prot-a-1306 | rs72840032 | T | C | 10 | 101889964 | -0.7891 | 2.69E-41 | 0.0586 | 0.04656 | -13.46587031 | 181.3296631 |
| 3-hydroxyanthranilate 3,4-dioxygenase || id:prot-a-1306 | rs704 | A | G | 17 | 26694861 | 0.1796 | 2.29E-13 | 0.0245 | 0.46665 | 7.330612245 | 53.73787589 |
| 3-hydroxyanthranilate 3,4-dioxygenase || id:prot-a-1306 | rs138948484 | T | G | 18 | 25875634 | -0.6676 | 2.40E-08 | 0.1196 | 0.01274 | -5.581939799 | 31.15805192 |
| 3-mercaptopyruvate sulfurtransferase || id:prot-a-1933 | rs56012317 | T | C | 13 | 86606931 | 0.2791 | 4.37E-08 | 0.051 | 0.06618 | 5.47254902 | 29.94879277 |
| 39S ribosomal protein L14, mitochondrial || id:prot-a-1940 | rs704 | A | G | 17 | 26694861 | 0.1344 | 4.57E-08 | 0.0246 | 0.46665 | 5.463414634 | 29.84889946 |
| 39S ribosomal protein L33, mitochondrial || id:prot-a-1942 | rs17090693 | C | T | 14 | 94841331 | 0.5466 | 1.51E-93 | 0.0266 | 0.25344 | 20.54887218 | 422.2561479 |
| 39S ribosomal protein L33, mitochondrial || id:prot-a-1942 | rs28929474 | T | C | 14 | 94844947 | 1.7387 | 4.27E-109 | 0.0784 | 0.02269 | 22.17729592 | 491.8324543 |
| 39S ribosomal protein L34, mitochondrial || id:prot-a-1943 | rs62143206 | T | G | 19 | 54326212 | -0.1875 | 3.55E-10 | 0.0299 | 0.2131 | -6.27090301 | 39.32422456 |
| 40S ribosomal protein S3 || id:prot-a-2593 | rs62143197 | A | G | 19 | 54320716 | 0.2379 | 3.31E-15 | 0.0302 | 0.22311 | 7.877483444 | 62.05474541 |
| 40S ribosomal protein S3a || id:prot-a-2594 | rs62143197 | A | G | 19 | 54320716 | 0.5819 | 3.63E-91 | 0.0287 | 0.22311 | 20.27526132 | 411.0862218 |
| 40S ribosomal protein S4, X isoform || id:prot-a-2595 | rs13276307 | T | C | 8 | 144646352 | 0.171 | 2.00E-09 | 0.0285 | 0.25219 | 6 | 36 |
| 40S ribosomal protein S4, X isoform || id:prot-a-2595 | rs62143206 | T | G | 19 | 54326212 | 0.6593 | 2.09E-124 | 0.0278 | 0.2131 | 23.71582734 | 562.4404663 |
| 40S ribosomal protein S7 || id:prot-a-2597 | rs13276307 | T | C | 8 | 144646352 | 0.1727 | 1.38E-09 | 0.0285 | 0.25219 | 6.059649123 | 36.71934749 |
| 40S ribosomal protein S7 || id:prot-a-2597 | rs62143206 | T | G | 19 | 54326212 | 0.7535 | 1.17E-170 | 0.0271 | 0.2131 | 27.80442804 | 773.0862189 |
| 40S ribosomal protein SA || id:prot-a-2598 | rs34436714 | A | C | 19 | 54327313 | 0.4491 | 1.82E-53 | 0.0292 | 0.21294 | 15.38013699 | 236.5486137 |
| 40S ribosomal protein SA || id:prot-a-2598 | rs2838522 | C | T | 21 | 45627976 | -0.1576 | 1.55E-08 | 0.0279 | 0.29511 | -5.64874552 | 31.90832595 |
| 5'(3')-deoxyribonucleotidase, cytosolic type || id:prot-a-2112 | rs78625720 | A | G | 17 | 73140941 | -0.7529 | 1.20E-25 | 0.0719 | 0.02954 | -10.47148818 | 109.6520647 |
| 5-formyltetrahydrofolate cyclo-ligase || id:prot-a-1962 | rs7173566 | C | T | 15 | 80211691 | -0.2126 | 6.03E-16 | 0.0263 | 0.34288 | -8.08365019 | 65.3454004 |
| 5-formyltetrahydrofolate cyclo-ligase || id:prot-a-1962 | rs62143198 | A | G | 19 | 54320939 | 0.6538 | 5.37E-116 | 0.0286 | 0.21453 | 22.86013986 | 522.5859944 |
| 6-pyruvoyl tetrahydrobiopterin synthase || id:prot-a-2450 | rs72960445 | T | C | 11 | 91720183 | -0.4665 | 3.09E-08 | 0.0843 | 0.02221 | -5.533807829 | 30.62302909 |
| 60S ribosomal protein L30 || id:prot-a-2586 | rs62143206 | T | G | 19 | 54326212 | 0.5266 | 1.55E-75 | 0.0286 | 0.2131 | 18.41258741 | 339.0233752 |
| 72 kDa inositol polyphosphate 5-phosphatase || id:prot-a-1557 | rs6993770 | T | A | 8 | 106581528 | -0.1805 | 4.07E-11 | 0.0274 | 0.28225 | -6.587591241 | 43.39635836 |
| A disintegrin and metalloproteinase with thrombospondin motifs 13 || id:prot-a-32 | rs7047076 | T | A | 9 | 136125329 | 0.3011 | 4.79E-10 | 0.0483 | 0.07554 | 6.233954451 | 38.8621881 |
| A disintegrin and metalloproteinase with thrombospondin motifs 13 || id:prot-a-32 | rs2301613 | G | C | 9 | 136314677 | -0.2545 | 9.77E-24 | 0.0253 | 0.38026 | -10.05928854 | 101.1892859 |
| A disintegrin and metalloproteinase with thrombospondin motifs 13 || id:prot-a-32 | rs71503194 | G | T | 9 | 136298131 | -0.8719 | 4.37E-103 | 0.0404 | 0.09123 | -21.58168317 | 465.7690484 |
| A disintegrin and metalloproteinase with thrombospondin motifs 4 || id:prot-a-35 | rs10418046 | G | T | 19 | 54327869 | -0.2296 | 1.12E-14 | 0.0297 | 0.21727 | -7.730639731 | 59.76279064 |
| A disintegrin and metalloproteinase with thrombospondin motifs 5 || id:prot-a-36 | rs7259081 | A | T | 19 | 54339243 | 0.1728 | 3.89E-08 | 0.0314 | 0.75126 | 5.503184713 | 30.28504199 |
| A disintegrin and metalloproteinase with thrombospondin motifs 5 || id:prot-a-36 | rs2830586 | G | T | 21 | 28305477 | -0.582 | 2.24E-72 | 0.0323 | 0.16451 | -18.01857585 | 324.6690757 |
| A disintegrin and metalloproteinase with thrombospondin motifs 6 || id:prot-a-37 | rs13235513 | A | G | 7 | 81821589 | 0.1539 | 2.57E-08 | 0.0276 | 0.67503 | 5.576086957 | 31.09274575 |
| A disintegrin and metalloproteinase with thrombospondin motifs 6 || id:prot-a-37 | rs2956632 | A | G | 11 | 18257017 | -1.4523 | 4.07E-82 | 0.0757 | 0.9741 | -19.18494055 | 368.0619441 |
| A disintegrin and metalloproteinase with thrombospondin motifs 6 || id:prot-a-37 | rs118076271 | A | G | 18 | 31959768 | -0.6441 | 1.29E-08 | 0.1133 | 0.01238 | -5.684907326 | 32.3181713 |
| Abelson tyrosine-protein kinase 2 || id:prot-a-8 | rs967645 | T | C | 17 | 26713970 | -0.1962 | 6.76E-16 | 0.0243 | 0.50945 | -8.074074074 | 65.19067215 |
| Acetyl-CoA carboxylase 2 || id:prot-a-11 | rs74480769 | G | A | 5 | 40972211 | -0.4363 | 2.75E-09 | 0.0734 | 0.0317 | -5.944141689 | 35.33282042 |
| Acetyl-CoA carboxylase 2 || id:prot-a-11 | rs34879232 | G | GA | 17 | 26722039 | -0.1541 | 3.55E-10 | 0.0246 | 0.45209 | -6.264227642 | 39.24054795 |
| Acid ceramidase || id:prot-a-178 | rs77903926 | C | T | 4 | 12656113 | 0.469 | 2.40E-09 | 0.0786 | 0.0301 | 5.96692112 | 35.60414765 |
| Acid ceramidase || id:prot-a-178 | rs62143197 | A | G | 19 | 54320716 | 0.3715 | 1.05E-35 | 0.0298 | 0.22311 | 12.46644295 | 155.4121999 |
| Acid sphingomyelinase-like phosphodiesterase 3a || id:prot-a-2780 | rs28385609 | T | C | 6 | 123122464 | -1.0756 | 1.00E-200 | 0.03 | 0.14505 | -35.85333333 | 1285.461511 |
| Acid sphingomyelinase-like phosphodiesterase 3a || id:prot-a-2780 | rs7964859 | G | C | 12 | 102220783 | -0.4001 | 1.20E-51 | 0.0265 | 0.2939 | -15.09811321 | 227.9530224 |
| Acidic leucine-rich nuclear phosphoprotein 32 family member B || id:prot-a-105 | rs13412535 | A | G | 2 | 224874874 | 0.1743 | 2.14E-08 | 0.0311 | 0.23946 | 5.604501608 | 31.41043827 |
| Acidic leucine-rich nuclear phosphoprotein 32 family member B || id:prot-a-105 | rs4241819 | T | C | 4 | 187157140 | -0.2037 | 1.32E-16 | 0.0246 | 0.50721 | -8.280487805 | 68.56647829 |
| Actin-related protein 2/3 complex subunit 3 || id:prot-a-166 | rs241775 | T | C | 17 | 26644668 | -0.1431 | 4.47E-09 | 0.0244 | 0.47792 | -5.864754098 | 34.39534063 |
| Activated Protein C || id:prot-a-2380 | rs13402560 | G | C | 2 | 3639915 | -0.1829 | 2.51E-08 | 0.0328 | 0.81367 | -5.576219512 | 31.09422405 |
| Activated Protein C || id:prot-a-2380 | rs8119351 | A | G | 20 | 33754405 | 0.2672 | 3.24E-09 | 0.0451 | 0.08331 | 5.924611973 | 35.10102704 |
| Activating signal cointegrator 1 complex subunit 1 || id:prot-a-181 | rs4253254 | T | C | 4 | 187158508 | 0.2294 | 1.10E-20 | 0.0246 | 0.51206 | 9.325203252 | 86.95941569 |
| Activating signal cointegrator 1 complex subunit 1 || id:prot-a-181 | rs2731674 | G | T | 5 | 176839890 | 0.2223 | 2.24E-15 | 0.028 | 0.75301 | 7.939285714 | 63.03225765 |
| Activating signal cointegrator 1 complex subunit 1 || id:prot-a-181 | rs117425389 | G | A | 17 | 25607347 | 0.598 | 6.92E-09 | 0.1033 | 0.01492 | 5.788964182 | 33.5121063 |
| Activator of 90 kDa heat shock protein ATPase homolog 1 || id:prot-a-60 | rs10418046 | G | T | 19 | 54327869 | 0.5017 | 1.62E-68 | 0.0287 | 0.21727 | 17.48083624 | 305.5796355 |
| Activin receptor type-1B || id:prot-a-22 | rs62143196 | G | A | 19 | 54320636 | -0.1983 | 5.25E-11 | 0.0302 | 0.22404 | -6.566225166 | 43.11531292 |
| Acyl-CoA-binding domain-containing protein 6 || id:prot-a-14 | rs62143197 | A | G | 19 | 54320716 | 0.4012 | 1.12E-41 | 0.0297 | 0.22311 | 13.50841751 | 182.4773436 |
| Acyl-CoA-binding domain-containing protein 7 || id:prot-a-15 | rs3917532 | T | A | 7 | 94940119 | 0.2034 | 1.15E-13 | 0.0274 | 0.28253 | 7.423357664 | 55.10623901 |
| Acyl-CoA-binding domain-containing protein 7 || id:prot-a-15 | rs10418046 | G | T | 19 | 54327869 | 0.2334 | 3.89E-15 | 0.0297 | 0.21727 | 7.858585859 | 61.7573717 |
| Acylphosphatase-2 || id:prot-a-24 | rs13417064 | G | A | 2 | 54362726 | 0.3598 | 3.16E-11 | 0.0542 | 0.05494 | 6.638376384 | 44.06804101 |
| Acylphosphatase-2 || id:prot-a-24 | rs62143206 | T | G | 19 | 54326212 | 0.448 | 1.12E-53 | 0.029 | 0.2131 | 15.44827586 | 238.6492271 |
| Adenosine 3'-phospho 5'-phosphosulfate transporter 2 || id:prot-a-2755 | rs147759747 | C | A | 8 | 14807493 | 0.4803 | 4.37E-08 | 0.0877 | 0.02076 | 5.476624857 | 29.99341983 |
| Adenosine deaminase CECR1 || id:prot-a-506 | rs5748977 | C | T | 22 | 17719458 | -0.2218 | 6.03E-18 | 0.0257 | 0.54455 | -8.630350195 | 74.48294448 |
| Adenosine deaminase CECR1 || id:prot-a-506 | rs2231495 | C | T | 22 | 17669306 | -0.8794 | 1.00E-200 | 0.0214 | 0.33252 | -41.09345794 | 1688.672286 |
| Adenylosuccinate synthetase isozyme 2 || id:prot-a-51 | rs13276307 | T | C | 8 | 144646352 | 0.1561 | 4.57E-08 | 0.0285 | 0.25219 | 5.477192982 | 29.99964297 |
| Adenylosuccinate synthetase isozyme 2 || id:prot-a-51 | rs10418046 | G | T | 19 | 54327869 | 0.5001 | 4.90E-68 | 0.0287 | 0.21727 | 17.42508711 | 303.6336607 |
| Adenylyltransferase and sulfurtransferase MOCS3 || id:prot-a-1926 | rs7212510 | A | T | 17 | 26703682 | 0.1354 | 2.88E-08 | 0.0244 | 0.50911 | 5.549180328 | 30.79340231 |
| Adhesion G-protein coupled receptor F1 || id:prot-a-43 | rs704 | A | G | 17 | 26694861 | 0.4648 | 2.34E-88 | 0.0233 | 0.46665 | 19.94849785 | 397.9425666 |
| Adhesion G-protein coupled receptor F1 || id:prot-a-43 | rs429358 | C | T | 19 | 45411941 | 0.3769 | 6.61E-30 | 0.0332 | 0.15301 | 11.35240964 | 128.8772046 |
| Adhesion G protein-coupled receptor F5 || id:prot-a-44 | rs140477171 | G | A | 5 | 55331303 | 0.1742 | 3.80E-08 | 0.0317 | 0.19311 | 5.495268139 | 30.19797192 |
| Adhesion G protein-coupled receptor F5 || id:prot-a-44 | rs499116 | G | A | 6 | 46833695 | 1.3657 | 1.86E-197 | 0.0456 | 0.93784 | 29.9495614 | 896.9762283 |
| Adhesion G protein-coupled receptor F5 || id:prot-a-44 | rs13204743 | C | T | 6 | 46618456 | -0.1479 | 1.78E-08 | 0.0263 | 0.33876 | -5.623574144 | 31.62458616 |
| Adhesion G protein-coupled receptor F5 || id:prot-a-44 | rs41307428 | T | C | 9 | 136336804 | 0.6671 | 1.23E-16 | 0.0806 | 0.02484 | 8.276674938 | 68.50334803 |
| Adhesion G protein-coupled receptor F5 || id:prot-a-44 | rs2519093 | T | C | 9 | 136141870 | -0.7777 | 1.38E-155 | 0.0293 | 0.17823 | -26.54266212 | 704.5129122 |
| Adipocyte plasma membrane-associated protein || id:prot-a-124 | rs147545924 | T | G | 2 | 114124349 | -0.162 | 7.08E-09 | 0.028 | 0.28484 | -5.785714286 | 33.4744898 |
| Adipocyte plasma membrane-associated protein || id:prot-a-124 | rs8125909 | C | A | 20 | 24975835 | 0.2967 | 2.75E-16 | 0.0363 | 0.13207 | 8.173553719 | 66.8069804 |
| ADP-ribose pyrophosphatase, mitochondrial || id:prot-a-2129 | rs28696943 | G | A | 4 | 88310135 | -0.3571 | 2.29E-28 | 0.0323 | 0.16685 | -11.05572755 | 122.2291118 |
| ADP-ribose pyrophosphatase, mitochondrial || id:prot-a-2129 | rs112635299 | T | G | 14 | 94838142 | 0.4711 | 2.04E-08 | 0.084 | 0.02261 | 5.608333333 | 31.45340278 |
| ADP-ribose pyrophosphatase, mitochondrial || id:prot-a-2129 | rs1801689 | C | A | 17 | 64210580 | 1.0267 | 1.38E-49 | 0.0694 | 0.03043 | 14.79394813 | 218.8609012 |
| ADP-ribosyl cyclase/cyclic ADP-ribose hydrolase 1 || id:prot-a-444 | rs7080536 | A | G | 10 | 115348046 | -0.3942 | 2.45E-10 | 0.0623 | 0.04384 | -6.327447833 | 40.03659608 |
| ADP-ribosyl cyclase/cyclic ADP-ribose hydrolase 1 || id:prot-a-444 | rs704 | A | G | 17 | 26694861 | 0.1411 | 9.33E-09 | 0.0246 | 0.46665 | 5.735772358 | 32.89908454 |
| ADP-ribosyl cyclase/cyclic ADP-ribose hydrolase 1 || id:prot-a-444 | rs36020076 | A | G | 19 | 15580290 | -0.1991 | 7.24E-11 | 0.0306 | 0.21124 | -6.506535948 | 42.33501004 |
| ADP-ribosyl cyclase/cyclic ADP-ribose hydrolase 2 || id:prot-a-276 | rs73224660 | A | G | 4 | 15714762 | -1.3967 | 1.00E-200 | 0.0239 | 0.159 | -58.43933054 | 3415.155354 |
| ADP-ribosyl cyclase/cyclic ADP-ribose hydrolase 2 || id:prot-a-276 | rs13116208 | T | G | 4 | 15430626 | 0.2309 | 4.68E-18 | 0.0267 | 0.68803 | 8.647940075 | 74.78686754 |
| ADP-ribosylation factor-binding protein GGA1 || id:prot-a-1205 | rs62143198 | A | G | 19 | 54320939 | 0.4711 | 6.03E-57 | 0.0296 | 0.21453 | 15.91554054 | 253.3044307 |
| ADP-ribosylation factor-binding protein GGA3 || id:prot-a-1206 | rs62143198 | A | G | 19 | 54320939 | 0.5049 | 7.59E-66 | 0.0295 | 0.21453 | 17.11525424 | 292.9319276 |
| ADP-ribosylation factor-like protein 1 || id:prot-a-161 | rs6993770 | T | A | 8 | 106581528 | -0.1735 | 2.29E-10 | 0.0274 | 0.28225 | -6.332116788 | 40.09570302 |
| ADP-ribosylation factor-like protein 1 || id:prot-a-161 | rs429358 | C | T | 19 | 45411941 | -0.3034 | 1.15E-19 | 0.0334 | 0.15301 | -9.083832335 | 82.5160099 |
| ADP-ribosylation factor-like protein 11 || id:prot-a-160 | rs2668202 | G | A | 3 | 165490468 | -0.1634 | 1.58E-10 | 0.0255 | 0.6373 | -6.407843137 | 41.06045367 |
| ADP-ribosylation factor-like protein 3 || id:prot-a-162 | rs201096208 | CA | C | 10 | 104348149 | -0.3048 | 8.71E-10 | 0.0497 | 0.0748 | -6.132796781 | 37.61119635 |
| ADP-ribosylation factor-like protein 3 || id:prot-a-162 | rs62143197 | A | G | 19 | 54320716 | 0.5768 | 2.24E-89 | 0.0288 | 0.22311 | 20.02777778 | 401.1118827 |
| ADP-ribosylation factor 6 || id:prot-a-143 | rs77611151 | C | A | 7 | 19098463 | 0.4512 | 3.98E-08 | 0.0822 | 0.03146 | 5.489051095 | 30.12968192 |
| ADP-ribosylation factor 6 || id:prot-a-143 | rs4632248 | T | G | 19 | 54324995 | 0.4793 | 8.13E-62 | 0.0289 | 0.21439 | 16.58477509 | 275.0547647 |
| ADP-ribosylation factor GTPase-activating protein 2 || id:prot-a-144 | rs62143198 | A | G | 19 | 54320939 | 0.5906 | 2.14E-92 | 0.029 | 0.21453 | 20.36551724 | 414.7542925 |
| Adrenomedullin || id:prot-a-48 | rs2668196 | T | A | 3 | 165502709 | 0.1788 | 2.95E-08 | 0.0323 | 0.81693 | 5.535603715 | 30.64290849 |
| Adrenomedullin || id:prot-a-48 | rs2304456 | G | T | 3 | 186445052 | -0.2614 | 2.88E-12 | 0.0374 | 0.12046 | -6.989304813 | 48.85038177 |
| Adrenomedullin || id:prot-a-48 | rs71640034 | A | G | 4 | 187161048 | -0.1941 | 3.72E-15 | 0.0247 | 0.51294 | -7.858299595 | 61.75287253 |
| Adrenomedullin || id:prot-a-48 | rs2545801 | C | T | 5 | 176841339 | -0.1658 | 3.39E-09 | 0.0281 | 0.75043 | -5.900355872 | 34.81419941 |
| Adrenomedullin || id:prot-a-48 | rs3813135 | C | T | 19 | 15587345 | -0.2687 | 2.34E-26 | 0.0253 | 0.37913 | -10.62055336 | 112.7961537 |
| Adseverin || id:prot-a-2650 | rs181978376 | C | T | 10 | 101797515 | 0.4358 | 3.16E-13 | 0.0598 | 0.04586 | 7.287625418 | 53.10948423 |
| Adseverin || id:prot-a-2650 | rs1126605 | T | C | 12 | 7242204 | 0.3535 | 1.45E-15 | 0.0443 | 0.08581 | 7.979683973 | 63.67535631 |
| Advanced glycosylation end product-specific receptor, soluble || id:prot-a-54 | rs1569419 | C | T | 1 | 2996602 | -0.1751 | 2.82E-09 | 0.0295 | 0.75795 | -5.93559322 | 35.23126688 |
| Advanced glycosylation end product-specific receptor, soluble || id:prot-a-54 | rs9469156 | T | C | 6 | 32545255 | -0.1744 | 9.33E-10 | 0.0285 | 0.40319 | -6.119298246 | 37.44581102 |
| Advanced glycosylation end product-specific receptor, soluble || id:prot-a-54 | rs2070600 | T | C | 6 | 32151443 | -0.5749 | 3.89E-33 | 0.0479 | 0.06687 | -12.00208768 | 144.0501087 |
| Advanced glycosylation end product-specific receptor, soluble || id:prot-a-54 | rs57942103 | C | A | 8 | 106513461 | 0.2208 | 1.82E-14 | 0.0288 | 0.24742 | 7.666666667 | 58.77777778 |
| Advanced glycosylation end product-specific receptor, soluble || id:prot-a-54 | rs28767468 | G | C | 9 | 134426376 | -0.2084 | 3.72E-16 | 0.0256 | 0.55395 | -8.140625 | 66.26977539 |
| Advanced glycosylation end product-specific receptor, soluble || id:prot-a-54 | rs35393359 | T | C | 12 | 623456 | -0.2167 | 5.01E-11 | 0.033 | 0.16843 | -6.566666667 | 43.12111111 |
| Advanced glycosylation end product-specific receptor, soluble || id:prot-a-54 | rs4899178 | T | C | 14 | 65862987 | 0.2406 | 8.71E-22 | 0.0251 | 0.60705 | 9.585657371 | 91.88482722 |
| Agouti-related protein || id:prot-a-59 | rs60697456 | A | G | 16 | 67543522 | 0.4702 | 7.24E-15 | 0.0604 | 0.04208 | 7.784768212 | 60.60261611 |
| Agouti-signaling protein || id:prot-a-187 | rs11473437 | GGC | G | 20 | 34720494 | -0.1876 | 1.26E-12 | 0.0264 | 0.59642 | -7.106060606 | 50.49609734 |
| Agouti-signaling protein || id:prot-a-187 | rs6059655 | G | A | 20 | 32665748 | -0.936 | 2.24E-125 | 0.0393 | 0.90482 | -23.81679389 | 567.2396713 |
| Alanine--tRNA ligase, cytoplasmic || id:prot-a-3 | rs62143197 | A | G | 19 | 54320716 | 0.2068 | 8.32E-12 | 0.0303 | 0.22311 | 6.825082508 | 46.58175124 |
| Alcohol dehydrogenase [NADP(+)] || id:prot-a-67 | rs140964940 | T | TTTGTTGTTG | 1 | 45888431 | -0.2093 | 2.82E-12 | 0.03 | 0.25838 | -6.976666667 | 48.67387778 |
| Alcohol dehydrogenase [NADP(+)] || id:prot-a-67 | rs72688441 | A | G | 1 | 46051053 | -1.18 | 1.20E-126 | 0.0493 | 0.05896 | -23.93509128 | 572.8885945 |
| Alcohol dehydrogenase [NADP(+)] || id:prot-a-67 | rs62143198 | A | G | 19 | 54320939 | 0.6002 | 9.55E-96 | 0.0289 | 0.21453 | 20.76816609 | 431.3167227 |
| Alcohol dehydrogenase 1B || id:prot-a-46 | rs78844723 | T | C | 2 | 211314977 | -0.1371 | 3.16E-08 | 0.0248 | 0.53709 | -5.528225806 | 30.56128057 |
| Alcohol dehydrogenase 1B || id:prot-a-46 | rs13085791 | A | C | 3 | 49721798 | -0.4199 | 3.16E-59 | 0.0259 | 0.29502 | -16.21235521 | 262.8404615 |
| Aldehyde dehydrogenase family 3 member B1 || id:prot-a-76 | rs62143198 | A | G | 19 | 54320939 | 0.5255 | 1.02E-71 | 0.0293 | 0.21453 | 17.93515358 | 321.6697341 |
| Aldehyde dehydrogenase, dimeric NADP-preferring || id:prot-a-75 | rs72668136 | T | C | 1 | 37264855 | 0.6104 | 1.95E-09 | 0.1017 | 0.01705 | 6.001966568 | 36.02360269 |
| Aldehyde dehydrogenase, dimeric NADP-preferring || id:prot-a-75 | rs887241 | C | A | 17 | 19645938 | 0.2421 | 7.76E-21 | 0.0259 | 0.66164 | 9.347490347 | 87.3755758 |
| Aldo-keto reductase family 1 member C1 || id:prot-a-69 | rs145648894 | G | T | 10 | 5009739 | 0.3894 | 3.80E-23 | 0.0393 | 0.11138 | 9.908396947 | 98.17633005 |
| Aldo-keto reductase family 1 member C1 || id:prot-a-69 | rs62143198 | A | G | 19 | 54320939 | 0.1716 | 2.04E-08 | 0.0306 | 0.21453 | 5.607843137 | 31.44790465 |
| Aldose reductase || id:prot-a-68 | rs2229542 | C | T | 7 | 134135621 | -0.8187 | 4.68E-15 | 0.1045 | 0.01434 | -7.834449761 | 61.37860305 |
| Aldose reductase || id:prot-a-68 | rs62143197 | A | G | 19 | 54320716 | 0.3959 | 1.45E-40 | 0.0297 | 0.22311 | 13.32996633 | 177.6880024 |
| All-trans-retinol 13,14-reductase || id:prot-a-2525 | rs6955592 | G | T | 7 | 103689003 | 0.3317 | 2.40E-08 | 0.0595 | 0.04484 | 5.574789916 | 31.07828261 |
| Allergin-1 || id:prot-a-1901 | rs3917532 | T | A | 7 | 94940119 | 0.257 | 4.27E-21 | 0.0273 | 0.28253 | 9.413919414 | 88.62187873 |
| Allergin-1 || id:prot-a-1901 | rs854572 | G | C | 7 | 94954696 | -0.1358 | 3.89E-08 | 0.0247 | 0.54095 | -5.497975709 | 30.22773689 |
| Allograft inflammatory factor 1 || id:prot-a-62 | rs62143206 | T | G | 19 | 54326212 | 0.64 | 3.39E-116 | 0.0279 | 0.2131 | 22.9390681 | 526.2008453 |
| Alpha-(1,3)-fucosyltransferase 10 || id:prot-a-1151 | rs2732317 | C | A | 8 | 33330687 | -0.4501 | 2.45E-77 | 0.0242 | 0.61158 | -18.59917355 | 345.9292569 |
| Alpha-(1,3)-fucosyltransferase 10 || id:prot-a-1151 | rs372743606 | T | G | 8 | 33176900 | 0.3045 | 9.55E-24 | 0.0303 | 0.32004 | 10.04950495 | 100.9925498 |
| Alpha-(1,3)-fucosyltransferase 5 || id:prot-a-1153 | rs8101385 | T | C | 19 | 5838595 | -0.3948 | 2.19E-18 | 0.0451 | 0.08203 | -8.753880266 | 76.63041971 |
| Alpha-(1,3)-fucosyltransferase 5 || id:prot-a-1153 | rs11666151 | A | G | 19 | 5871662 | 0.333 | 5.75E-40 | 0.0252 | 0.58766 | 13.21428571 | 174.6173469 |
| Alpha-(1,3)-fucosyltransferase 5 || id:prot-a-1153 | rs778809 | A | G | 19 | 5830302 | -0.58 | 1.32E-118 | 0.025 | 0.3014 | -23.2 | 538.24 |
| Alpha-(1,3)-fucosyltransferase 9 || id:prot-a-1155 | rs704 | A | G | 17 | 26694861 | -0.5457 | 1.20E-126 | 0.0228 | 0.46665 | -23.93421053 | 572.8464335 |
| Alpha-(1,3)-fucosyltransferase 9 || id:prot-a-1155 | rs62143206 | T | G | 19 | 54326212 | 0.2895 | 1.58E-22 | 0.0296 | 0.2131 | 9.780405405 | 95.65632989 |
| Alpha-(1,6)-fucosyltransferase || id:prot-a-1154 | rs6993770 | T | A | 8 | 106581528 | -0.1756 | 1.38E-10 | 0.0274 | 0.28225 | -6.408759124 | 41.07219351 |
| Alpha-(1,6)-fucosyltransferase || id:prot-a-1154 | rs61987801 | G | A | 14 | 65715424 | 0.1793 | 1.78E-09 | 0.0298 | 0.20947 | 6.016778523 | 36.2016238 |
| Alpha-(1,6)-fucosyltransferase || id:prot-a-1154 | rs2127870 | C | G | 14 | 65796846 | -1.049 | 1.00E-200 | 0.0246 | 0.78457 | -42.64227642 | 1818.363739 |
| Alpha-1,3-mannosyl-glycoprotein 4-beta-N-acetylglucosaminyltransferase B || id:prot-a-1894 | rs73351608 | T | A | 5 | 179232064 | -0.9586 | 2.19E-29 | 0.0852 | 0.02152 | -11.25117371 | 126.5889098 |
| Alpha-1,6-mannosyl-glycoprotein 2-beta-N-acetylglucosaminyltransferase || id:prot-a-1893 | rs2005918 | G | T | 12 | 102080976 | 0.1508 | 5.25E-09 | 0.0258 | 0.59074 | 5.84496124 | 34.1635719 |
| Alpha-1,6-mannosyl-glycoprotein 2-beta-N-acetylglucosaminyltransferase || id:prot-a-1893 | rs28396798 | T | C | 14 | 50075319 | 0.1949 | 1.26E-15 | 0.0244 | 0.54747 | 7.987704918 | 63.80342986 |
| Alpha-2-antiplasmin || id:prot-a-2700 | rs11657394 | A | C | 17 | 1636950 | -0.3459 | 5.37E-13 | 0.0479 | 0.0774 | -7.221294363 | 52.14709228 |
| Alpha-2-HS-glycoprotein || id:prot-a-61 | rs35094235 | G | T | 3 | 186328951 | 0.6252 | 2.04E-132 | 0.0255 | 0.731 | 24.51764706 | 601.1150173 |
| Alpha-2-HS-glycoprotein || id:prot-a-61 | rs55972847 | T | C | 3 | 186400172 | 0.3153 | 3.31E-08 | 0.0571 | 0.05148 | 5.521891419 | 30.49128484 |
| alpha-2-macroglobulin receptor-associated protein || id:prot-a-1781 | rs78770234 | A | G | 4 | 3496683 | -0.6821 | 3.63E-23 | 0.0688 | 0.03262 | -9.914244186 | 98.29223778 |
| alpha-2-macroglobulin receptor-associated protein || id:prot-a-1781 | rs7739937 | C | G | 6 | 29502630 | 0.7639 | 1.29E-11 | 0.1128 | 0.01177 | 6.772163121 | 45.86219333 |
| alpha-2-macroglobulin receptor-associated protein || id:prot-a-1781 | rs41552714 | A | G | 6 | 31324756 | 1.8105 | 1.00E-200 | 0.0547 | 0.03927 | 33.09872029 | 1095.525285 |
| alpha-2-macroglobulin receptor-associated protein || id:prot-a-1781 | rs9264277 | C | T | 6 | 31224667 | -0.2177 | 8.91E-18 | 0.0254 | 0.62091 | -8.570866142 | 73.45974642 |
| alpha-2-macroglobulin receptor-associated protein || id:prot-a-1781 | rs41284523 | T | C | 6 | 31810266 | 0.3564 | 2.19E-12 | 0.0508 | 0.06014 | 7.015748031 | 49.22072044 |
| alpha-2-macroglobulin receptor-associated protein || id:prot-a-1782 | rs41284527 | G | A | 6 | 31818279 | 0.3303 | 8.71E-11 | 0.0509 | 0.05965 | 6.489194499 | 42.10964525 |
| alpha-2-macroglobulin receptor-associated protein || id:prot-a-1782 | rs9264277 | C | T | 6 | 31224667 | -0.1798 | 1.55E-12 | 0.0254 | 0.62091 | -7.078740157 | 50.10856222 |
| alpha-2-macroglobulin receptor-associated protein || id:prot-a-1782 | rs41552714 | A | G | 6 | 31324756 | 1.5535 | 1.45E-163 | 0.057 | 0.03927 | 27.25438596 | 742.8015543 |
| alpha-2-macroglobulin receptor-associated protein || id:prot-a-1782 | rs6937034 | G | A | 6 | 33079766 | 0.4152 | 3.02E-09 | 0.07 | 0.03135 | 5.931428571 | 35.1818449 |
| Alpha-2,8-sialyltransferase 8F || id:prot-a-2861 | rs62143194 | G | C | 19 | 54319624 | -0.1832 | 2.09E-09 | 0.0306 | 0.22364 | -5.986928105 | 35.84330813 |
| Alpha-amylase 1 || id:prot-a-89 | rs151171462 | A | G | 1 | 104091681 | 0.523 | 4.68E-08 | 0.0957 | 0.0214 | 5.464994775 | 29.86616789 |
| Alpha-amylase 1 || id:prot-a-89 | rs7538379 | T | C | 1 | 104067356 | -1.0399 | 4.90E-63 | 0.062 | 0.03995 | -16.77258065 | 281.3194615 |
| Alpha-amylase 1 || id:prot-a-89 | rs114922930 | T | C | 1 | 104131457 | -0.8428 | 3.98E-15 | 0.1073 | 0.01399 | -7.854613234 | 61.69494905 |
| Alpha-amylase 1 || id:prot-a-89 | rs61788971 | T | C | 1 | 104777083 | -0.4392 | 4.79E-10 | 0.0706 | 0.03054 | -6.220963173 | 38.7003828 |
| alpha-Fetoprotein || id:prot-a-53 | rs6855349 | A | G | 4 | 73956736 | 0.1366 | 3.98E-08 | 0.0249 | 0.43922 | 5.485943775 | 30.0955791 |
| Alpha-L-iduronidase || id:prot-a-1411 | rs3822020 | G | A | 4 | 985727 | 0.608 | 7.24E-148 | 0.0235 | 0.63658 | 25.87234043 | 669.3779991 |
| Alpha-L-iduronidase || id:prot-a-1411 | rs142576171 | A | G | 4 | 1187703 | 0.5106 | 3.24E-14 | 0.0673 | 0.03611 | 7.58692422 | 57.56141912 |
| Alpha-L-iduronidase || id:prot-a-1411 | rs6479908 | G | C | 10 | 65333648 | -0.1391 | 2.95E-08 | 0.0251 | 0.46934 | -5.541832669 | 30.71190933 |
| Alpha-L-iduronidase || id:prot-a-1411 | rs7964859 | G | C | 12 | 102220783 | -0.2399 | 7.24E-19 | 0.027 | 0.2939 | -8.885185185 | 78.94651578 |
| Alpha-N-acetylgalactosaminide alpha-2,6-sialyltransferase 1 || id:prot-a-2852 | rs12038333 | A | G | 1 | 196672454 | -0.1387 | 4.57E-08 | 0.0254 | 0.6091 | -5.460629921 | 29.81847914 |
| Alpha-N-acetylgalactosaminide alpha-2,6-sialyltransferase 1 || id:prot-a-2852 | rs75335238 | G | A | 5 | 67560173 | -0.6029 | 4.68E-08 | 0.1104 | 0.01402 | -5.461050725 | 29.82307502 |
| Alpha-N-acetylgalactosaminide alpha-2,6-sialyltransferase 1 || id:prot-a-2852 | rs74480769 | G | A | 5 | 40972211 | -0.5178 | 1.51E-12 | 0.0732 | 0.0317 | -7.073770492 | 50.03822897 |
| Alpha-N-acetylgalactosaminide alpha-2,6-sialyltransferase 1 || id:prot-a-2852 | rs704 | A | G | 17 | 26694861 | 0.2702 | 7.41E-29 | 0.0242 | 0.46665 | 11.16528926 | 124.6636842 |
| Alpha-N-acetylgalactosaminide alpha-2,6-sialyltransferase 2 || id:prot-a-2853 | rs33944729 | T | TA | 1 | 196652124 | 0.1659 | 5.62E-11 | 0.0253 | 0.38701 | 6.557312253 | 42.99834398 |
| Alpha-N-acetylgalactosaminide alpha-2,6-sialyltransferase 2 || id:prot-a-2853 | rs74480769 | G | A | 5 | 40972211 | -0.5824 | 1.55E-15 | 0.0731 | 0.0317 | -7.967168263 | 63.47577013 |
| Alpha-N-acetylgalactosaminide alpha-2,6-sialyltransferase 3 || id:prot-a-2854 | rs4632248 | T | G | 19 | 54324995 | 0.2981 | 8.13E-24 | 0.0296 | 0.21439 | 10.07094595 | 101.4239522 |
| Alpha-synuclein || id:prot-a-2789 | rs2245801 | C | T | 4 | 90757840 | -0.2507 | 1.17E-16 | 0.0303 | 0.78828 | -8.273927393 | 68.4578745 |
| Alpha-synuclein || id:prot-a-2790 | rs2245801 | C | T | 4 | 90757840 | -0.2303 | 2.88E-14 | 0.0303 | 0.78828 | -7.600660066 | 57.77003344 |
| Alpha-taxilin || id:prot-a-3120 | rs10418046 | G | T | 19 | 54327869 | 0.2445 | 1.74E-16 | 0.0297 | 0.21727 | 8.232323232 | 67.7711458 |
| Alpha/beta hydrolase domain-containing protein 14A || id:prot-a-6 | rs62143198 | A | G | 19 | 54320939 | 0.6208 | 2.88E-103 | 0.0288 | 0.21453 | 21.55555556 | 464.6419753 |
| Ameloblastin || id:prot-a-82 | rs9269233 | C | A | 6 | 32451762 | 0.162 | 3.72E-08 | 0.0294 | 0.75355 | 5.510204082 | 30.36234902 |
| Ameloblastin || id:prot-a-82 | rs1071803 | C | T | 14 | 106209119 | 0.5615 | 3.63E-108 | 0.0254 | 0.66685 | 22.10629921 | 488.6884649 |
| Amelogenin, X isoform || id:prot-a-83 | rs61804164 | C | G | 1 | 161623025 | -0.2408 | 1.70E-10 | 0.0377 | 0.14644 | -6.387267905 | 40.79719128 |
| Amelogenin, X isoform || id:prot-a-83 | rs1048372 | C | T | 6 | 32610436 | 0.1755 | 5.01E-12 | 0.0254 | 0.60918 | 6.909448819 | 47.74048298 |
| Amelogenin, X isoform || id:prot-a-83 | rs112206797 | G | A | 14 | 106264935 | -0.4221 | 2.95E-21 | 0.0446 | 0.115 | -9.464125561 | 89.56967263 |
| AMP Kinase (alpha2beta2gamma1) || id:prot-a-2366 | rs62143197 | A | G | 19 | 54320716 | 0.2023 | 2.34E-11 | 0.0303 | 0.22311 | 6.676567657 | 44.57655568 |
| Amphoterin-induced protein 1 || id:prot-a-87 | rs4253311 | G | A | 4 | 187174683 | 0.1414 | 1.29E-08 | 0.0249 | 0.51063 | 5.678714859 | 32.24780245 |
| Amyloid-like protein 2 || id:prot-a-122 | rs6993770 | T | A | 8 | 106581528 | -0.2387 | 1.78E-18 | 0.0272 | 0.28225 | -8.775735294 | 77.01352995 |
| Amyloid-like protein 2 || id:prot-a-122 | rs6590438 | G | A | 11 | 129973928 | 0.3622 | 1.51E-10 | 0.0565 | 0.05173 | 6.410619469 | 41.09604198 |
| Amyloid-like protein 2 || id:prot-a-123 | rs3900989 | C | T | 3 | 116970253 | -0.1657 | 1.29E-08 | 0.0291 | 0.38862 | -5.694158076 | 32.42343619 |
| Amyloid beta A4 precursor protein-binding family B member 1 || id:prot-a-114 | rs35788064 | C | T | 9 | 10765210 | 0.2767 | 2.63E-08 | 0.0497 | 0.07855 | 5.567404427 | 30.99599205 |
| Amyloid beta A4 precursor protein-binding family B member 1 || id:prot-a-114 | rs7212114 | G | A | 17 | 3779277 | 0.2176 | 4.57E-08 | 0.0398 | 0.87849 | 5.467336683 | 29.89177041 |
| Amyloid beta A4 precursor protein-binding family B member 1 || id:prot-a-115 | rs6993770 | T | A | 8 | 106581528 | 0.1549 | 1.55E-08 | 0.0274 | 0.28225 | 5.653284672 | 31.95962758 |
| Amyloid beta A4 precursor protein-binding family B member 2 || id:prot-a-116 | rs7412 | T | C | 19 | 45412079 | 0.5726 | 1.95E-36 | 0.0454 | 0.07776 | 12.6123348 | 159.0709892 |
| Amyloid beta A4 precursor protein-binding family B member 2 || id:prot-a-117 | rs113747960 | A | G | 12 | 7181948 | -0.3441 | 2.75E-21 | 0.0363 | 0.13276 | -9.479338843 | 89.8578649 |
| Amyloid beta A4 protein || id:prot-a-139 | rs6993770 | T | A | 8 | 106581528 | -0.1577 | 8.51E-09 | 0.0274 | 0.28225 | -5.755474453 | 33.12548617 |
| Anaphase-promoting complex subunit 7 || id:prot-a-91 | rs523427 | G | A | 1 | 197223447 | -0.3467 | 7.24E-09 | 0.0599 | 0.95536 | -5.787979967 | 33.50071209 |
| Anaphase-promoting complex subunit 7 || id:prot-a-91 | rs528298 | T | A | 1 | 196660995 | 0.876 | 1.00E-200 | 0.0204 | 0.61415 | 42.94117647 | 1843.944637 |
| Angiogenic factor with G patch and FHA domains 1 || id:prot-a-56 | rs62143196 | G | A | 19 | 54320636 | 0.2186 | 4.07E-13 | 0.0301 | 0.22404 | 7.262458472 | 52.74330305 |
| Angiopoietin-1 || id:prot-a-92 | rs6993770 | T | A | 8 | 106581528 | -0.2331 | 1.10E-17 | 0.0272 | 0.28225 | -8.569852941 | 73.44237943 |
| Angiopoietin-1 receptor, soluble || id:prot-a-2944 | rs1050518 | A | T | 6 | 31324641 | -0.1702 | 2.95E-08 | 0.0307 | 0.26228 | -5.543973941 | 30.73564706 |
| Angiopoietin-1 receptor, soluble || id:prot-a-2944 | rs35030851 | T | G | 9 | 27197486 | 0.5573 | 1.41E-22 | 0.057 | 0.04665 | 9.777192982 | 95.59350262 |
| Angiopoietin-1 receptor, soluble || id:prot-a-2944 | rs8176693 | T | C | 9 | 136137657 | 0.5635 | 1.41E-30 | 0.049 | 0.06647 | 11.5 | 132.25 |
| Angiopoietin-related protein 1 || id:prot-a-96 | rs60209294 | T | G | 1 | 178868787 | -0.374 | 1.02E-15 | 0.0466 | 0.08272 | -8.025751073 | 64.41268029 |
| Angiopoietin-related protein 1 || id:prot-a-96 | rs114711590 | G | A | 1 | 179660993 | 0.6959 | 3.47E-09 | 0.1178 | 0.01134 | 5.907470289 | 34.89820521 |
| Angiopoietin-related protein 1 || id:prot-a-96 | rs16853043 | G | A | 1 | 178545926 | 0.4305 | 5.37E-68 | 0.0247 | 0.35239 | 17.4291498 | 303.7752627 |
| Angiopoietin-related protein 1 || id:prot-a-97 | rs34983651 | CAT | C | 2 | 234668879 | 0.5496 | 1.95E-102 | 0.0256 | 0.3047 | 21.46875 | 460.9072266 |
| Angiopoietin-related protein 4 || id:prot-a-99 | rs13402561 | G | C | 2 | 3639921 | -0.206 | 3.72E-10 | 0.0329 | 0.81414 | -6.261398176 | 39.20510712 |
| Angiopoietin-related protein 4 || id:prot-a-99 | rs967645 | T | C | 17 | 26713970 | -0.1429 | 4.90E-09 | 0.0244 | 0.50945 | -5.856557377 | 34.29926431 |
| Angiopoietin-related protein 4 || id:prot-a-99 | rs911155 | G | T | 20 | 56357247 | 0.1822 | 3.39E-08 | 0.033 | 0.17544 | 5.521212121 | 30.48378329 |
| Angiopoietin-related protein 7 || id:prot-a-100 | rs4806509 | T | G | 19 | 54337096 | -0.1755 | 1.00E-09 | 0.0287 | 0.6723 | -6.114982578 | 37.39301193 |
| Angiostatin || id:prot-a-2301 | rs11751347 | T | C | 6 | 161092438 | -0.4032 | 4.68E-23 | 0.0408 | 0.10061 | -9.882352941 | 97.66089965 |
| Angiostatin || id:prot-a-2301 | rs150683617 | C | T | 6 | 161205283 | 0.6133 | 2.19E-16 | 0.0747 | 0.03093 | 8.210174029 | 67.40695759 |
| Angiostatin || id:prot-a-2301 | rs7090660 | A | G | 10 | 17580890 | 0.1452 | 1.70E-08 | 0.0257 | 0.36375 | 5.649805447 | 31.92030159 |
| Ankyrin-2 || id:prot-a-101 | rs13302990 | C | T | 1 | 203428170 | 1.6211 | 6.17E-54 | 0.1048 | 0.01568 | 15.46851145 | 239.2748465 |
| Ankyrin repeat domain-containing protein 46 || id:prot-a-103 | rs114119740 | C | T | 3 | 26357265 | 0.521 | 3.98E-08 | 0.0949 | 0.01716 | 5.489989463 | 30.1399843 |
| Ankyrin repeat domain-containing protein 46 || id:prot-a-103 | rs2232613 | T | C | 20 | 36997655 | -0.2568 | 2.40E-08 | 0.046 | 0.0768 | -5.582608696 | 31.16551985 |
| Annexin A1 || id:prot-a-107 | rs62143206 | T | G | 19 | 54326212 | 0.608 | 1.86E-103 | 0.0281 | 0.2131 | 21.63701068 | 468.160231 |
| Annexin A10 || id:prot-a-106 | rs2073867 | C | G | 17 | 26678960 | 0.214 | 8.91E-19 | 0.0242 | 0.5081 | 8.842975207 | 78.1982105 |
| Annexin A2 || id:prot-a-108 | rs62143206 | T | G | 19 | 54326212 | 0.8673 | 1.00E-200 | 0.026 | 0.2131 | 33.35769231 | 1112.735636 |
| Annexin A2 || id:prot-a-109 | rs62143206 | T | G | 19 | 54326212 | 0.8607 | 1.00E-200 | 0.0261 | 0.2131 | 32.97701149 | 1087.483287 |
| Annexin A9 || id:prot-a-110 | rs145480373 | T | G | 4 | 128089685 | -0.6262 | 4.79E-08 | 0.1147 | 0.01278 | -5.459459459 | 29.80569759 |
| Annexin A9 || id:prot-a-110 | rs72654473 | A | C | 19 | 45414399 | -0.2498 | 1.05E-09 | 0.041 | 0.10398 | -6.092682927 | 37.12078525 |
| Anosmin-1 || id:prot-a-1606 | rs16860992 | C | G | 3 | 186394038 | 0.1736 | 8.13E-09 | 0.0301 | 0.20414 | 5.76744186 | 33.26338561 |
| Anosmin-1 || id:prot-a-1606 | rs4241818 | C | T | 4 | 187153786 | -0.1362 | 3.55E-08 | 0.0247 | 0.51359 | -5.51417004 | 30.40607124 |
| Anosmin-1 || id:prot-a-1606 | rs188270072 | C | T | 14 | 106208166 | -0.3 | 3.89E-08 | 0.0546 | 0.06372 | -5.494505495 | 30.18959063 |
| Antigen-presenting glycoprotein CD1d || id:prot-a-421 | rs2141551 | A | C | 1 | 107538505 | 0.1497 | 6.92E-09 | 0.0258 | 0.35708 | 5.802325581 | 33.66698215 |
| Antithrombin-III || id:prot-a-2695 | rs7159711 | A | G | 14 | 97169037 | -0.1664 | 4.90E-09 | 0.0284 | 0.74891 | -5.85915493 | 34.32969649 |
| AP-4 complex accessory subunit tepsin || id:prot-a-949 | rs61745945 | A | G | 17 | 79205421 | -0.8768 | 5.89E-19 | 0.0986 | 0.01636 | -8.892494929 | 79.07646606 |
| AP-4 complex subunit mu-1 || id:prot-a-112 | rs612563 | C | G | 1 | 57397738 | 0.1984 | 5.89E-14 | 0.0264 | 0.32394 | 7.515151515 | 56.4775023 |
| AP-4 complex subunit mu-1 || id:prot-a-112 | rs12493107 | T | G | 3 | 52706724 | -0.1806 | 8.32E-13 | 0.0252 | 0.3865 | -7.166666667 | 51.36111111 |
| AP-4 complex subunit mu-1 || id:prot-a-112 | rs79204107 | A | C | 9 | 71173918 | 0.397 | 3.47E-08 | 0.072 | 0.02996 | 5.513888889 | 30.40297068 |
| AP-4 complex subunit mu-1 || id:prot-a-112 | rs7862602 | T | G | 9 | 139840471 | -0.1489 | 1.66E-09 | 0.0247 | 0.52553 | -6.028340081 | 36.34088413 |
| AP-4 complex subunit mu-1 || id:prot-a-112 | rs80167373 | A | G | 10 | 7766581 | -0.3957 | 1.29E-13 | 0.0534 | 0.05791 | -7.41011236 | 54.90976518 |
| AP-4 complex subunit mu-1 || id:prot-a-112 | rs10418046 | G | T | 19 | 54327869 | -0.228 | 1.66E-14 | 0.0297 | 0.21727 | -7.676767677 | 58.93276196 |
| APOBEC1 complementation factor || id:prot-a-1 | rs2304456 | G | T | 3 | 186445052 | 0.4647 | 1.62E-36 | 0.0368 | 0.12046 | 12.62771739 | 159.4592465 |
| Apolipoprotein A-V || id:prot-a-125 | rs964184 | C | G | 11 | 116648917 | 0.2625 | 4.90E-13 | 0.0363 | 0.86898 | 7.231404959 | 52.29321768 |
| Apolipoprotein A-V || id:prot-a-126 | rs967645 | T | C | 17 | 26713970 | 0.2498 | 4.57E-25 | 0.0242 | 0.50945 | 10.32231405 | 106.5501673 |
| Apolipoprotein B || id:prot-a-127 | rs1065853 | T | G | 19 | 45413233 | -0.4265 | 1.48E-20 | 0.0459 | 0.07778 | -9.291938998 | 86.34013034 |
| Apolipoprotein D || id:prot-a-129 | rs7539005 | T | A | 1 | 196667252 | -0.2373 | 5.75E-21 | 0.0253 | 0.61028 | -9.37944664 | 87.97401928 |
| Apolipoprotein D || id:prot-a-129 | rs74480769 | G | A | 5 | 40972211 | -0.4931 | 1.70E-11 | 0.0733 | 0.0317 | -6.727148704 | 45.25452969 |
| Apolipoprotein D || id:prot-a-130 | rs139828053 | C | T | 3 | 195298892 | -0.4064 | 2.19E-08 | 0.0726 | 0.03228 | -5.597796143 | 31.33532166 |
| Apolipoprotein E (isoform E2) || id:prot-a-132 | rs814573 | T | A | 19 | 45424351 | 0.2472 | 5.37E-15 | 0.0316 | 0.1977 | 7.82278481 | 61.19596219 |
| Apolipoprotein E (isoform E3) || id:prot-a-131 | rs1065853 | T | G | 19 | 45413233 | -0.7217 | 1.86E-58 | 0.0448 | 0.07778 | -16.109375 | 259.5119629 |
| Apolipoprotein F || id:prot-a-133 | rs369160772 | CA | C | 12 | 56673561 | -0.3752 | 2.95E-15 | 0.0476 | 0.07124 | -7.882352941 | 62.13148789 |
| Apolipoprotein L1 || id:prot-a-134 | rs71403839 | C | T | 16 | 71246530 | 0.2478 | 3.24E-08 | 0.0448 | 0.08254 | 5.53125 | 30.59472656 |
| Apolipoprotein L1 || id:prot-a-134 | rs3794693 | G | C | 16 | 72092390 | -0.3188 | 2.82E-24 | 0.0314 | 0.19649 | -10.15286624 | 103.0806929 |
| Apolipoprotein L1 || id:prot-a-134 | rs763665 | T | C | 16 | 72078043 | 0.6157 | 7.41E-82 | 0.0321 | 0.16362 | 19.18068536 | 367.8986908 |
| Apolipoprotein L1 || id:prot-a-134 | rs71314970 | T | C | 22 | 36638705 | -0.3659 | 3.47E-19 | 0.0409 | 0.10576 | -8.946210269 | 80.03467818 |
| Apolipoprotein L1 || id:prot-a-135 | rs6809081 | C | T | 3 | 194060475 | 0.7302 | 1.00E-200 | 0.0222 | 0.42319 | 32.89189189 | 1081.876552 |
| Apolipoprotein L1 || id:prot-a-135 | rs147121860 | A | G | 10 | 101331237 | -0.7409 | 4.79E-08 | 0.1357 | 0.00963 | -5.459837878 | 29.80982965 |
| Apolipoprotein L1 || id:prot-a-135 | rs12769233 | G | C | 10 | 101636163 | -0.1584 | 1.48E-10 | 0.0247 | 0.50098 | -6.412955466 | 41.1259978 |
| Apolipoprotein L1 || id:prot-a-135 | rs61751507 | T | C | 10 | 101829514 | -1.3296 | 3.72E-127 | 0.0554 | 0.04472 | -24 | 576 |
| Apolipoprotein L1 || id:prot-a-135 | rs704 | A | G | 17 | 26694861 | 0.1615 | 4.57E-11 | 0.0245 | 0.46665 | 6.591836735 | 43.45231154 |
| Apolipoprotein L1 || id:prot-a-135 | rs5167 | G | T | 19 | 45448465 | 0.1849 | 7.41E-13 | 0.0258 | 0.34696 | 7.166666667 | 51.36111111 |
| Apolipoprotein M || id:prot-a-136 | rs1803274 | T | C | 3 | 165491280 | 0.2433 | 2.88E-15 | 0.0308 | 0.20345 | 7.899350649 | 62.39974068 |
| Apolipoprotein M || id:prot-a-136 | rs1266078 | G | T | 6 | 31622043 | -0.4899 | 3.02E-12 | 0.0702 | 0.03188 | -6.978632479 | 48.70131127 |
| Apolipoprotein M || id:prot-a-137 | rs2736158 | C | G | 6 | 31600304 | -0.4623 | 9.33E-11 | 0.0714 | 0.03063 | -6.474789916 | 41.92290446 |
| Apoptosis-inducing factor 1, mitochondrial || id:prot-a-63 | rs61983942 | T | G | 14 | 106219445 | -0.4083 | 1.41E-52 | 0.0268 | 0.66348 | -15.23507463 | 232.1074989 |
| Apoptosis-inducing factor 1, mitochondrial || id:prot-a-63 | rs78745329 | G | A | 14 | 106479137 | 0.6545 | 5.13E-61 | 0.0397 | 0.1096 | 16.4861461 | 271.7930131 |
| Apoptotic protease-activating factor 1 || id:prot-a-113 | rs62143197 | A | G | 19 | 54320716 | 0.3951 | 2.09E-40 | 0.0297 | 0.22311 | 13.3030303 | 176.9706152 |
| Arachidonate 15-lipoxygenase B || id:prot-a-80 | rs4792145 | A | C | 17 | 7947328 | -0.1817 | 1.07E-12 | 0.0255 | 0.49655 | -7.125490196 | 50.77261053 |
| Arachidonate 15-lipoxygenase B || id:prot-a-80 | rs10424405 | G | A | 19 | 54321933 | 0.4371 | 4.79E-50 | 0.0294 | 0.2203 | 14.86734694 | 221.038005 |
| Arf-GAP domain and FG repeat-containing protein 1 || id:prot-a-55 | rs62143198 | A | G | 19 | 54320939 | 0.7009 | 3.09E-136 | 0.0282 | 0.21453 | 24.85460993 | 617.7516347 |
| Arf-GAP with coiled-coil, ANK repeat and PH domain-containing protein 2 || id:prot-a-13 | rs62143198 | A | G | 19 | 54320939 | 0.2792 | 3.72E-20 | 0.0304 | 0.21453 | 9.184210526 | 84.34972299 |
| Arf-GAP with SH3 domain, ANK repeat and PH domain-containing protein 2 || id:prot-a-180 | rs704 | A | G | 17 | 26694861 | 0.6097 | 1.32E-164 | 0.0223 | 0.46665 | 27.34080717 | 747.519737 |
| Arf-GAP with SH3 domain, ANK repeat and PH domain-containing protein 2 || id:prot-a-180 | rs62143197 | A | G | 19 | 54320716 | 0.2049 | 1.29E-11 | 0.0303 | 0.22311 | 6.762376238 | 45.72973238 |
| Arfaptin-1 || id:prot-a-145 | rs4619875 | T | C | 4 | 153701130 | 0.239 | 6.61E-22 | 0.0248 | 0.40363 | 9.637096774 | 92.87363424 |
| Arfaptin-1 || id:prot-a-145 | rs62143197 | A | G | 19 | 54320716 | 0.3909 | 1.48E-39 | 0.0297 | 0.22311 | 13.16161616 | 173.22814 |
| Arfaptin-2 || id:prot-a-146 | rs28929474 | T | C | 14 | 94844947 | -0.6448 | 9.55E-15 | 0.0833 | 0.02269 | -7.740696279 | 59.91837888 |
| Argininosuccinate lyase || id:prot-a-188 | rs6460315 | T | C | 7 | 66289040 | -0.1512 | 4.37E-08 | 0.0276 | 0.27224 | -5.47826087 | 30.01134216 |
| Armadillo repeat-containing protein 5 || id:prot-a-164 | rs9653560 | T | C | 2 | 26626836 | -0.7601 | 3.80E-08 | 0.1382 | 0.00855 | -5.5 | 30.25 |
| Armadillo repeat-containing protein 5 || id:prot-a-165 | rs74480769 | G | A | 5 | 40972211 | -0.4176 | 1.29E-08 | 0.0734 | 0.0317 | -5.689373297 | 32.36896851 |
| Arrestin domain-containing protein 3 || id:prot-a-170 | rs45488800 | A | C | 1 | 120267061 | 0.2103 | 2.40E-08 | 0.0377 | 0.12872 | 5.578249337 | 31.11686566 |
| Arylamine N-acetyltransferase 1 || id:prot-a-2001 | rs678 | T | A | 3 | 52820981 | -0.3365 | 1.10E-40 | 0.0252 | 0.35906 | -13.3531746 | 178.307272 |
| Arylamine N-acetyltransferase 1 || id:prot-a-2001 | rs41290289 | C | A | 10 | 7769806 | -0.3808 | 1.74E-12 | 0.054 | 0.05572 | -7.051851852 | 49.72861454 |
| Arylsulfatase B || id:prot-a-172 | rs13159135 | C | G | 5 | 78196689 | -0.214 | 5.89E-18 | 0.0248 | 0.43391 | -8.629032258 | 74.46019771 |
| Arylsulfatase K || id:prot-a-173 | rs11056782 | A | G | 12 | 8141652 | -0.1995 | 4.47E-16 | 0.0246 | 0.56806 | -8.109756098 | 65.76814396 |
| Arylsulfatase K || id:prot-a-173 | rs10745925 | C | T | 12 | 102218899 | -0.2449 | 1.07E-19 | 0.027 | 0.29402 | -9.07037037 | 82.27161866 |
| Aspartate aminotransferase, cytoplasmic || id:prot-a-1241 | rs4253282 | T | C | 4 | 187164399 | 0.1648 | 2.82E-11 | 0.0248 | 0.51241 | 6.64516129 | 44.15816857 |
| Aspartate aminotransferase, cytoplasmic || id:prot-a-1241 | rs1956456 | T | C | 14 | 94642962 | -0.33 | 5.62E-23 | 0.0334 | 0.83603 | -9.880239521 | 97.61913299 |
| Aspartyl/asparaginyl beta-hydroxylase || id:prot-a-191 | rs112760834 | T | G | 8 | 62540134 | -0.5784 | 4.07E-17 | 0.0688 | 0.03321 | -8.406976744 | 70.67725798 |
| Asporin || id:prot-a-192 | rs2516568 | T | A | 9 | 95187380 | -0.4724 | 2.34E-79 | 0.025 | 0.32324 | -18.896 | 357.058816 |
| AT-rich interactive domain-containing protein 3A || id:prot-a-159 | rs12950335 | G | A | 17 | 38215314 | -0.1392 | 4.07E-08 | 0.0254 | 0.58952 | -5.480314961 | 30.03385207 |
| ATP-citrate synthase || id:prot-a-17 | rs8178824 | T | C | 17 | 64224775 | -0.4797 | 3.16E-11 | 0.0723 | 0.02967 | -6.634854772 | 44.02129784 |
| ATP-dependent RNA helicase DDX25 || id:prot-a-777 | rs4846303 | T | G | 1 | 219730799 | -0.1444 | 3.55E-08 | 0.0262 | 0.31941 | -5.511450382 | 30.37608531 |
| ATP-dependent RNA helicase DDX25 || id:prot-a-777 | rs10418046 | G | T | 19 | 54327869 | -0.1779 | 2.45E-09 | 0.0298 | 0.21727 | -5.969798658 | 35.63849601 |
| ATP-dependent RNA helicase DHX8 || id:prot-a-816 | rs112376176 | T | C | 6 | 160874032 | -0.3317 | 3.89E-08 | 0.0604 | 0.04163 | -5.491721854 | 30.15900893 |
| ATP-dependent RNA helicase DHX8 || id:prot-a-816 | rs8178854 | G | A | 17 | 64214270 | -0.7667 | 1.00E-200 | 0.0236 | 0.30092 | -32.48728814 | 1055.42389 |
| ATP synthase subunit beta, mitochondrial || id:prot-a-203 | rs74480769 | G | A | 5 | 40972211 | -0.4053 | 3.39E-08 | 0.0734 | 0.0317 | -5.521798365 | 30.49025719 |
| ATPase family AAA domain-containing protein 1 || id:prot-a-195 | rs13090388 | T | C | 3 | 49391082 | -0.1558 | 3.09E-09 | 0.0263 | 0.31362 | -5.923954373 | 35.09323541 |
| ATPase family AAA domain-containing protein 2 || id:prot-a-196 | rs11879623 | C | T | 19 | 2543621 | 0.1334 | 3.63E-08 | 0.0242 | 0.51405 | 5.512396694 | 30.38651731 |
| Atrial natriuretic factor || id:prot-a-2076 | rs112193373 | A | G | 16 | 3748761 | 0.1822 | 3.80E-08 | 0.0331 | 0.17414 | 5.504531722 | 30.29986948 |
| Augurin || id:prot-a-310 | rs7539005 | T | A | 1 | 196667252 | -0.1968 | 8.71E-15 | 0.0254 | 0.61028 | -7.748031496 | 60.03199206 |
| Augurin || id:prot-a-310 | rs74480769 | G | A | 5 | 40972211 | -0.4429 | 1.55E-09 | 0.0734 | 0.0317 | -6.034059946 | 36.40987943 |
| Augurin || id:prot-a-310 | rs967645 | T | C | 17 | 26713970 | 0.1501 | 7.59E-10 | 0.0244 | 0.50945 | 6.151639344 | 37.84266662 |
| Aurora kinase B || id:prot-a-205 | rs528298 | T | A | 1 | 196660995 | -0.1587 | 3.89E-10 | 0.0254 | 0.61415 | -6.248031496 | 39.03789758 |
| Aurora kinase B || id:prot-a-205 | rs74480769 | G | A | 5 | 40972211 | -0.4682 | 1.70E-10 | 0.0733 | 0.0317 | -6.38744884 | 40.79950269 |
| Aurora kinase B || id:prot-a-205 | rs704 | A | G | 17 | 26694861 | 0.1723 | 2.09E-12 | 0.0245 | 0.46665 | 7.032653061 | 49.45820908 |
| Axin-2 || id:prot-a-206 | rs74480769 | G | A | 5 | 40972211 | -0.4245 | 7.24E-09 | 0.0734 | 0.0317 | -5.783378747 | 33.44746973 |
| Axin-2 || id:prot-a-207 | rs1374203 | T | C | 2 | 46490443 | 0.1883 | 1.20E-08 | 0.033 | 0.81017 | 5.706060606 | 32.55912764 |
| Axin-2 || id:prot-a-207 | rs12147642 | T | G | 14 | 106205022 | -1.0039 | 1.00E-200 | 0.0206 | 0.6618 | -48.73300971 | 2374.906235 |
| Axin-2 || id:prot-a-207 | rs11625865 | A | G | 14 | 105644421 | -0.1552 | 1.05E-09 | 0.0254 | 0.60539 | -6.11023622 | 37.33498667 |
| B-cell antigen receptor complex-associated protein alpha chain || id:prot-a-461 | rs3917539 | TAA | T | 7 | 94937696 | 0.2334 | 1.23E-17 | 0.0273 | 0.28157 | 8.549450549 | 73.0931047 |
| B-cell antigen receptor complex-associated protein beta chain || id:prot-a-462 | rs61911033 | C | T | 11 | 129086105 | 0.1729 | 3.02E-08 | 0.0312 | 0.20954 | 5.541666667 | 30.71006944 |
| B-cell lymphoma 6 protein || id:prot-a-241 | rs146123796 | A | G | 5 | 162173720 | 0.7374 | 4.57E-08 | 0.1348 | 0.01135 | 5.470326409 | 29.92447103 |
| B-cell lymphoma/leukemia 10 || id:prot-a-235 | rs141738059 | C | T | 9 | 116832007 | 0.936 | 2.88E-17 | 0.1108 | 0.01278 | 8.44765343 | 71.36284847 |
| B-cell lymphoma/leukemia 10 || id:prot-a-235 | rs62143197 | A | G | 19 | 54320716 | 0.3744 | 2.95E-36 | 0.0298 | 0.22311 | 12.56375839 | 157.8480249 |
| B-cell receptor-associated protein 29 || id:prot-a-233 | rs7212510 | A | T | 17 | 26703682 | 0.1424 | 5.25E-09 | 0.0244 | 0.50911 | 5.836065574 | 34.05966138 |
| B-cell receptor CD22 || id:prot-a-427 | rs368465 | C | T | 1 | 196671981 | -0.1803 | 1.41E-12 | 0.0254 | 0.61344 | -7.098425197 | 50.38764028 |
| B-cell receptor CD22 || id:prot-a-427 | rs74480769 | G | A | 5 | 40972211 | -0.4909 | 2.09E-11 | 0.0733 | 0.0317 | -6.697135061 | 44.85161803 |
| B melanoma antigen 2 || id:prot-a-227 | rs10424405 | G | A | 19 | 54321933 | 0.5414 | 1.45E-78 | 0.0288 | 0.2203 | 18.79861111 | 353.3877797 |
| B melanoma antigen 3 || id:prot-a-228 | rs11903880 | T | C | 2 | 125068007 | 0.1448 | 3.02E-08 | 0.0261 | 0.38171 | 5.54789272 | 30.77911364 |
| Bactericidal permeability-increasing protein || id:prot-a-266 | rs62143198 | A | G | 19 | 54320939 | 0.169 | 3.24E-08 | 0.0306 | 0.21453 | 5.522875817 | 30.50215729 |
| Bactericidal permeability-increasing protein || id:prot-a-266 | rs1780617 | G | A | 20 | 36974157 | -0.6444 | 1.10E-67 | 0.0371 | 0.12241 | -17.36927224 | 301.691618 |
| Bactericidal permeability-increasing protein || id:prot-a-266 | rs1780628 | T | A | 20 | 37001409 | -0.1818 | 2.40E-13 | 0.0248 | 0.44162 | -7.330645161 | 53.73835848 |
| Baculoviral IAP repeat-containing protein 5 || id:prot-a-252 | rs7539005 | T | A | 1 | 196667252 | -0.1837 | 4.79E-13 | 0.0254 | 0.61028 | -7.232283465 | 52.30592411 |
| Baculoviral IAP repeat-containing protein 5 || id:prot-a-252 | rs74480769 | G | A | 5 | 40972211 | -0.4192 | 1.12E-08 | 0.0734 | 0.0317 | -5.711171662 | 32.61748175 |
| BAG family molecular chaperone regulator 3 || id:prot-a-225 | rs2234962 | C | T | 10 | 121429633 | 0.1854 | 5.62E-10 | 0.0299 | 0.22406 | 6.200668896 | 38.44829476 |
| Basal Cell Adhesion Molecule || id:prot-a-231 | rs8176747 | G | C | 9 | 136131315 | 0.3356 | 8.91E-12 | 0.0492 | 0.06686 | 6.821138211 | 46.5279265 |
| Basal Cell Adhesion Molecule || id:prot-a-231 | rs28399656 | A | T | 19 | 45316704 | 0.9208 | 2.34E-13 | 0.1256 | 0.01075 | 7.331210191 | 53.74664287 |
| Basic leucine zipper transcriptional factor ATF-like 3 || id:prot-a-230 | rs1831282 | C | A | 1 | 196673993 | -0.2354 | 1.82E-21 | 0.0247 | 0.57182 | -9.530364372 | 90.82784507 |
| Basic leucine zipper transcriptional factor ATF-like 3 || id:prot-a-230 | rs11721974 | C | G | 4 | 8524209 | -0.1841 | 3.63E-08 | 0.0334 | 0.16771 | -5.511976048 | 30.38187995 |
| Basic salivary proline-rich protein 4 || id:prot-a-2354 | rs58452280 | C | A | 14 | 106252435 | -0.4343 | 2.69E-23 | 0.0437 | 0.1235 | -9.938215103 | 98.76811943 |
| Basigin || id:prot-a-275 | rs13402560 | G | C | 2 | 3639915 | -0.1937 | 3.47E-09 | 0.0328 | 0.81367 | -5.905487805 | 34.87478621 |
| Bcl-2-like protein 1 || id:prot-a-238 | rs570618 | G | T | 1 | 196657064 | -0.1582 | 4.27E-10 | 0.0253 | 0.61149 | -6.252964427 | 39.09956412 |
| Bcl-2-like protein 1 || id:prot-a-238 | rs74480769 | G | A | 5 | 40972211 | -0.5695 | 6.61E-15 | 0.0731 | 0.0317 | -7.790697674 | 60.69497025 |
| Bcl-2-related protein A1 || id:prot-a-237 | rs2393969 | C | A | 10 | 65140440 | 0.1388 | 3.31E-08 | 0.0251 | 0.47282 | 5.529880478 | 30.5795781 |
| Bcl10-interacting CARD protein || id:prot-a-325 | rs4632248 | T | G | 19 | 54324995 | 0.7489 | 4.68E-168 | 0.0271 | 0.21439 | 27.63468635 | 763.6758895 |
| Bcl2-associated agonist of cell death || id:prot-a-224 | rs55688436 | T | C | 10 | 73643968 | 0.1729 | 1.66E-08 | 0.0306 | 0.20963 | 5.650326797 | 31.92619292 |
| BCL2/adenovirus E1B 19 kDa protein-interacting protein 3 || id:prot-a-261 | rs145093376 | T | C | 1 | 109169062 | 0.5011 | 2.57E-08 | 0.09 | 0.02246 | 5.567777778 | 31.00014938 |
| BCL2/adenovirus E1B 19 kDa protein-interacting protein 3 || id:prot-a-261 | rs9866126 | G | A | 3 | 186391067 | 0.1472 | 1.07E-08 | 0.0257 | 0.34566 | 5.727626459 | 32.80570486 |
| BET1-like protein || id:prot-a-245 | rs1277777 | A | G | 13 | 112666614 | 0.1929 | 2.63E-08 | 0.0347 | 0.15138 | 5.55907781 | 30.9033461 |
| Beta-1,3-galactosyl-O-glycosyl-glycoprotein beta-1,6-N-acetylglucosaminyltransferase 4 || id:prot-a-1188 | rs74480769 | G | A | 5 | 40972211 | -0.5638 | 1.23E-14 | 0.0731 | 0.0317 | -7.712722298 | 59.48608525 |
| Beta-1,3-galactosyl-O-glycosyl-glycoprotein beta-1,6-N-acetylglucosaminyltransferase 4 || id:prot-a-1188 | rs704 | A | G | 17 | 26694861 | 0.1554 | 2.40E-10 | 0.0245 | 0.46665 | 6.342857143 | 40.23183673 |
| Beta-1,3-galactosyltransferase 6 || id:prot-a-209 | rs34879232 | G | GA | 17 | 26722039 | -0.159 | 9.33E-11 | 0.0246 | 0.45209 | -6.463414634 | 41.77572873 |
| Beta-1,3-glucosyltransferase || id:prot-a-210 | rs4943306 | T | C | 13 | 31894515 | 0.1829 | 6.17E-10 | 0.0296 | 0.77309 | 6.179054054 | 38.180709 |
| Beta-1,3-N-acetylglucosaminyltransferase radical fringe || id:prot-a-2530 | rs1013579 | T | C | 1 | 57422484 | 0.4335 | 2.75E-08 | 0.078 | 0.97386 | 5.557692308 | 30.88794379 |
| Beta-1,3-N-acetylglucosaminyltransferase radical fringe || id:prot-a-2530 | rs438811 | T | C | 19 | 45416741 | -0.2294 | 2.29E-15 | 0.0289 | 0.23219 | -7.937716263 | 63.00733947 |
| Beta-1,4-galactosyltransferase 1 || id:prot-a-216 | rs7019909 | T | C | 9 | 33113322 | 0.4478 | 7.94E-29 | 0.0402 | 0.10257 | 11.13930348 | 124.0840821 |
| Beta-1,4-galactosyltransferase 1 || id:prot-a-216 | rs600038 | C | T | 9 | 136151806 | 0.2287 | 3.80E-14 | 0.0302 | 0.20651 | 7.572847682 | 57.34802202 |
| Beta-1,4-galactosyltransferase 1 || id:prot-a-216 | rs2386660 | T | C | 10 | 5672059 | -0.2019 | 1.35E-08 | 0.0355 | 0.14734 | -5.687323944 | 32.34565364 |
| Beta-1,4-galactosyltransferase 1 || id:prot-a-216 | rs7247412 | C | T | 19 | 52313868 | -0.2625 | 4.90E-21 | 0.0279 | 0.27059 | -9.408602151 | 88.52179443 |
| Beta-1,4-galactosyltransferase 1 || id:prot-a-216 | rs10418046 | G | T | 19 | 54327869 | -0.1676 | 1.95E-08 | 0.0298 | 0.21727 | -5.624161074 | 31.63118778 |
| Beta-1,4-galactosyltransferase 2 || id:prot-a-217 | rs2286241 | C | G | 1 | 44440769 | -0.673 | 3.02E-38 | 0.052 | 0.05617 | -12.94230769 | 167.5033284 |
| Beta-1,4-galactosyltransferase 2 || id:prot-a-217 | rs550057 | T | C | 9 | 136146597 | 0.1621 | 1.35E-08 | 0.0285 | 0.24577 | 5.687719298 | 32.35015082 |
| Beta-1,4-galactosyltransferase 3 || id:prot-a-218 | rs10418046 | G | T | 19 | 54327869 | -0.1686 | 1.62E-08 | 0.0298 | 0.21727 | -5.657718121 | 32.00977433 |
| Beta-1,4-galactosyltransferase 6 || id:prot-a-220 | rs3815217 | G | A | 7 | 156976981 | 0.1345 | 4.90E-08 | 0.0246 | 0.48839 | 5.467479675 | 29.89333399 |
| Beta-1,4-galactosyltransferase 6 || id:prot-a-220 | rs201022770 | CT | C | 18 | 29213359 | -1.0818 | 1.74E-77 | 0.0581 | 0.04343 | -18.61962134 | 346.6902989 |
| Beta-1,4-galactosyltransferase 6 || id:prot-a-220 | rs78796387 | T | G | 18 | 28787634 | 0.443 | 3.63E-11 | 0.067 | 0.03435 | 6.611940299 | 43.71775451 |
| Beta-1,4-galactosyltransferase 7 || id:prot-a-221 | rs6993770 | T | A | 8 | 106581528 | -0.1595 | 5.75E-09 | 0.0274 | 0.28225 | -5.821167883 | 33.88599552 |
| Beta-1,4-glucuronyltransferase 1 || id:prot-a-212 | rs55636108 | C | T | 3 | 186381262 | 0.3313 | 2.57E-29 | 0.0295 | 0.21184 | 11.23050847 | 126.1243206 |
| Beta-1,4-glucuronyltransferase 1 || id:prot-a-212 | rs76148901 | T | G | 14 | 106029906 | 0.7017 | 7.24E-09 | 0.1212 | 0.01315 | 5.78960396 | 33.51951402 |
| Beta-1,4-glucuronyltransferase 1 || id:prot-a-212 | rs58452280 | C | A | 14 | 106252435 | 0.9249 | 4.37E-111 | 0.0413 | 0.1235 | 22.39467312 | 501.5213843 |
| beta-adrenergic receptor kinase 1 || id:prot-a-50 | rs62143198 | A | G | 19 | 54320939 | 0.6637 | 4.57E-120 | 0.0285 | 0.21453 | 23.2877193 | 542.3178701 |
| Beta-arrestin-1 || id:prot-a-169 | rs62143197 | A | G | 19 | 54320716 | 0.3913 | 1.26E-39 | 0.0297 | 0.22311 | 13.17508418 | 173.582843 |
| Beta-crystallin B2 || id:prot-a-674 | rs3197999 | A | G | 3 | 49721532 | -0.1986 | 2.00E-13 | 0.027 | 0.28831 | -7.355555556 | 54.10419753 |
| Beta-crystallin B2 || id:prot-a-674 | rs4576502 | A | G | 9 | 26463378 | 0.1512 | 3.31E-08 | 0.0274 | 0.29045 | 5.518248175 | 30.45106292 |
| Beta-crystallin B2 || id:prot-a-674 | rs4774039 | C | T | 14 | 106427958 | -0.1642 | 7.59E-10 | 0.0267 | 0.62539 | -6.149812734 | 37.82019666 |
| Beta-defensin 1 || id:prot-a-804 | rs9857914 | A | G | 3 | 194063611 | -0.1994 | 8.51E-13 | 0.0279 | 0.28893 | -7.146953405 | 51.07894297 |
| Beta-defensin 1 || id:prot-a-804 | rs13355364 | T | C | 5 | 115274393 | 0.151 | 2.04E-09 | 0.0252 | 0.42945 | 5.992063492 | 35.90482489 |
| Beta-defensin 1 || id:prot-a-804 | rs2738176 | A | T | 8 | 6738228 | -0.3893 | 5.62E-55 | 0.0249 | 0.36503 | -15.63453815 | 244.4387832 |
| Beta-defensin 103 || id:prot-a-781 | rs62143194 | G | C | 19 | 54319624 | -0.1735 | 1.41E-08 | 0.0306 | 0.22364 | -5.669934641 | 32.14815883 |
| Beta-defensin 104 || id:prot-a-782 | rs147238933 | A | T | 8 | 12482661 | 0.8942 | 1.78E-09 | 0.1487 | 0.00907 | 6.013449899 | 36.16157969 |
| Beta-defensin 104 || id:prot-a-782 | rs183772362 | T | C | 8 | 7243016 | 0.5043 | 2.14E-12 | 0.0718 | 0.03257 | 7.02367688 | 49.33203692 |
| Beta-defensin 105 || id:prot-a-783 | rs704 | A | G | 17 | 26694861 | 0.2741 | 1.17E-29 | 0.0242 | 0.46665 | 11.32644628 | 128.2883854 |
| Beta-defensin 106 || id:prot-a-784 | rs139953802 | T | C | 6 | 169288009 | 0.434 | 2.88E-08 | 0.0782 | 0.03109 | 5.549872123 | 30.80108058 |
| Beta-defensin 106 || id:prot-a-784 | rs1885993 | C | T | 13 | 95411980 | -0.1425 | 2.19E-08 | 0.0255 | 0.5694 | -5.588235294 | 31.2283737 |
| Beta-defensin 108B || id:prot-a-786 | rs117277979 | G | A | 15 | 23969820 | -0.6055 | 2.09E-08 | 0.108 | 0.01653 | -5.606481481 | 31.4326346 |
| Beta-defensin 112 || id:prot-a-788 | rs35162472 | G | A | 14 | 106748982 | -0.3565 | 2.82E-32 | 0.0301 | 0.30278 | -11.84385382 | 140.2768733 |
| Beta-defensin 113 || id:prot-a-789 | rs71640036 | T | G | 4 | 187161120 | 0.1562 | 3.80E-10 | 0.0249 | 0.50238 | 6.273092369 | 39.35168788 |
| Beta-defensin 113 || id:prot-a-789 | rs2545801 | C | T | 5 | 176841339 | 0.1534 | 4.68E-08 | 0.0281 | 0.75043 | 5.459074733 | 29.80149694 |
| Beta-defensin 118 || id:prot-a-792 | rs13333572 | A | C | 16 | 19662758 | 0.3234 | 2.29E-08 | 0.0579 | 0.04609 | 5.585492228 | 31.19772343 |
| Beta-defensin 119 || id:prot-a-794 | rs144611086 | G | C | 3 | 94459509 | -0.5997 | 3.24E-08 | 0.1085 | 0.01422 | -5.52718894 | 30.54981758 |
| Beta-defensin 119 || id:prot-a-794 | rs79966773 | C | T | 6 | 32560341 | -0.1811 | 1.51E-09 | 0.03 | 0.23992 | -6.036666667 | 36.44134444 |
| Beta-defensin 119 || id:prot-a-794 | rs115050884 | A | T | 6 | 31756802 | 0.637 | 7.41E-11 | 0.0978 | 0.01528 | 6.513292434 | 42.42297832 |
| Beta-defensin 119 || id:prot-a-794 | rs9296004 | C | A | 6 | 31933977 | 0.6062 | 2.51E-40 | 0.0456 | 0.08156 | 13.29385965 | 176.7267044 |
| Beta-defensin 119 || id:prot-a-794 | rs2523582 | G | A | 6 | 31328092 | 0.2199 | 3.31E-16 | 0.0269 | 0.71608 | 8.17472119 | 66.82606653 |
| Beta-defensin 119 || id:prot-a-794 | rs12301299 | C | T | 12 | 991710 | 0.2542 | 2.04E-14 | 0.0332 | 0.163 | 7.656626506 | 58.62392945 |
| Beta-defensin 119 || id:prot-a-794 | rs11845244 | T | C | 14 | 107170077 | 0.2412 | 2.29E-20 | 0.0261 | 0.35433 | 9.24137931 | 85.40309156 |
| Beta-defensin 119 || id:prot-a-795 | rs72673751 | C | T | 8 | 106578940 | -0.1902 | 3.02E-09 | 0.0321 | 0.18949 | -5.925233645 | 35.10839375 |
| Beta-defensin 119 || id:prot-a-796 | rs1065853 | T | G | 19 | 45413233 | -0.4137 | 2.19E-19 | 0.0459 | 0.07778 | -9.013071895 | 81.23546499 |
| Beta-defensin 119 || id:prot-a-796 | rs34436714 | A | C | 19 | 54327313 | -0.1735 | 7.94E-09 | 0.0301 | 0.21294 | -5.764119601 | 33.22507478 |
| Beta-defensin 121 || id:prot-a-797 | rs704 | A | G | 17 | 26694861 | -0.1583 | 1.12E-10 | 0.0245 | 0.46665 | -6.46122449 | 41.74742191 |
| Beta-defensin 125 || id:prot-a-799 | rs5030049 | C | T | 3 | 186450863 | -0.2055 | 3.89E-08 | 0.0374 | 0.12183 | -5.494652406 | 30.19120507 |
| Beta-defensin 128 || id:prot-a-800 | rs62143206 | T | G | 19 | 54326212 | -0.21 | 2.00E-12 | 0.0299 | 0.2131 | -7.023411371 | 49.32830729 |
| Beta-defensin 134 || id:prot-a-801 | rs74480769 | G | A | 5 | 40972211 | -0.4214 | 9.33E-09 | 0.0734 | 0.0317 | -5.741144414 | 32.96073918 |
| Beta-endorphin || id:prot-a-2325 | rs7124741 | T | A | 11 | 116752219 | -0.4284 | 3.55E-24 | 0.0422 | 0.90585 | -10.15165877 | 103.0561757 |
| Beta-endorphin || id:prot-a-2325 | rs670 | T | C | 11 | 116708413 | -0.5442 | 8.13E-64 | 0.0323 | 0.16684 | -16.84829721 | 283.865119 |
| Beta-mannosidase || id:prot-a-1836 | rs140214719 | G | GC | 4 | 104004826 | -0.1893 | 2.75E-10 | 0.03 | 0.79334 | -6.31 | 39.8161 |
| Beta-mannosidase || id:prot-a-1836 | rs227370 | C | T | 4 | 103612043 | -0.5596 | 7.76E-113 | 0.0248 | 0.67461 | -22.56451613 | 509.1573881 |
| Beta-mannosidase || id:prot-a-1836 | rs10418046 | G | T | 19 | 54327869 | -0.2132 | 7.76E-13 | 0.0297 | 0.21727 | -7.178451178 | 51.53016132 |
| Beta-microseminoprotein || id:prot-a-1949 | rs1890736 | C | G | 10 | 45720222 | 0.1775 | 7.59E-13 | 0.0248 | 0.55473 | 7.157258065 | 51.226343 |
| Beta-microseminoprotein || id:prot-a-1949 | rs10993994 | C | T | 10 | 51549496 | 0.9821 | 1.00E-200 | 0.0189 | 0.59627 | 51.96296296 | 2700.14952 |
| beta-nerve growth factor || id:prot-a-2042 | rs2646172 | T | C | 2 | 175653671 | -0.1512 | 2.63E-08 | 0.0272 | 0.34663 | -5.558823529 | 30.90051903 |
| Beta-sarcoglycan || id:prot-a-2713 | rs200295770 | T | C | 6 | 33028734 | 0.2019 | 4.68E-08 | 0.037 | 0.14416 | 5.456756757 | 29.7761943 |
| Beta-sarcoglycan || id:prot-a-2713 | rs11849582 | G | A | 14 | 106195255 | 0.4308 | 4.47E-60 | 0.0264 | 0.66802 | 16.31818182 | 266.2830579 |
| Beta-soluble NSF attachment protein || id:prot-a-2000 | rs7658970 | C | T | 4 | 74713950 | -0.1839 | 4.37E-12 | 0.0266 | 0.7058 | -6.913533835 | 47.79695008 |
| Beta-soluble NSF attachment protein || id:prot-a-2000 | rs429358 | C | T | 19 | 45411941 | 0.3487 | 1.17E-25 | 0.0333 | 0.15301 | 10.47147147 | 109.6517148 |
| Bifunctional heparan sulfate N-deacetylase/N-sulfotransferase 1 || id:prot-a-2021 | rs6870546 | T | C | 5 | 149845731 | -0.3743 | 1.58E-09 | 0.062 | 0.04316 | -6.037096774 | 36.44653746 |
| Bifunctional heparan sulfate N-deacetylase/N-sulfotransferase 1 || id:prot-a-2021 | rs10418046 | G | T | 19 | 54327869 | -0.1856 | 4.79E-10 | 0.0298 | 0.21727 | -6.228187919 | 38.79032476 |
| Bifunctional polynucleotide phosphatase/kinase || id:prot-a-2314 | rs62143197 | A | G | 19 | 54320716 | 0.2676 | 6.46E-19 | 0.0301 | 0.22311 | 8.890365449 | 79.03859781 |
| Bifunctional polynucleotide phosphatase/kinase || id:prot-a-2314 | rs4812075 | G | A | 20 | 57950622 | -0.1996 | 1.51E-08 | 0.0353 | 0.83519 | -5.654390935 | 31.97213684 |
| Biglycan || id:prot-a-247 | rs62295996 | A | G | 3 | 165482064 | 0.3355 | 6.03E-28 | 0.0306 | 0.20233 | 10.96405229 | 120.2104426 |
| Biglycan || id:prot-a-247 | rs9574989 | A | C | 13 | 82479999 | 0.1648 | 2.82E-08 | 0.0297 | 0.24044 | 5.548821549 | 30.78942058 |
| Bile salt-activated lipase || id:prot-a-507 | rs8193016 | T | C | 9 | 135917744 | 0.6854 | 8.32E-24 | 0.0681 | 0.03478 | 10.06461087 | 101.2963919 |
| Bile salt sulfotransferase || id:prot-a-2893 | rs112285002 | T | C | 19 | 48374320 | -0.1981 | 1.29E-09 | 0.0327 | 0.1704 | -6.058103976 | 36.70062378 |
| Biliverdin reductase A || id:prot-a-253 | rs62143197 | A | G | 19 | 54320716 | 0.3168 | 4.27E-26 | 0.03 | 0.22311 | 10.56 | 111.5136 |
| Bis(5'-adenosyl)-triphosphatase || id:prot-a-1107 | rs34894181 | T | A | 3 | 60942431 | 0.1839 | 3.39E-10 | 0.0293 | 0.25322 | 6.276450512 | 39.39383103 |
| Bis(5'-adenosyl)-triphosphatase || id:prot-a-1107 | rs62143198 | A | G | 19 | 54320939 | 0.4798 | 3.80E-59 | 0.0296 | 0.21453 | 16.20945946 | 262.746576 |
| BMP-binding endothelial regulator protein || id:prot-a-258 | rs34157787 | C | G | 13 | 43495933 | -0.1401 | 4.79E-08 | 0.0256 | 0.48066 | -5.47265625 | 29.94996643 |
| Bombesin receptor-activated protein C6orf89 || id:prot-a-320 | rs919791 | A | G | 19 | 15576817 | -0.2714 | 6.31E-19 | 0.0305 | 0.21427 | -8.898360656 | 79.18082236 |
| Bone morphogenetic protein 6 || id:prot-a-256 | rs11607100 | T | C | 11 | 74414919 | -0.4334 | 1.78E-12 | 0.0615 | 0.04167 | -7.047154472 | 49.66238615 |
| Bone morphogenetic protein 7 || id:prot-a-257 | rs4241818 | C | T | 4 | 187153786 | 0.2067 | 3.72E-17 | 0.0245 | 0.51359 | 8.436734694 | 71.17849229 |
| Bone morphogenetic protein 7 || id:prot-a-257 | rs2731674 | G | T | 5 | 176839890 | 0.162 | 8.71E-09 | 0.0282 | 0.75301 | 5.744680851 | 33.00135808 |
| Bone sialoprotein 2 || id:prot-a-1395 | rs35530899 | A | G | 3 | 186363031 | -0.2405 | 3.31E-11 | 0.0363 | 0.13407 | -6.625344353 | 43.89518779 |
| Bone sialoprotein 2 || id:prot-a-1395 | rs967645 | T | C | 17 | 26713970 | -0.2563 | 2.19E-26 | 0.0241 | 0.50945 | -10.63485477 | 113.100136 |
| Bone sialoprotein 2 || id:prot-a-1395 | rs9610638 | C | T | 22 | 37445668 | -0.1567 | 4.68E-10 | 0.0252 | 0.56541 | -6.218253968 | 38.66668241 |
| Bone sialoprotein 2 || id:prot-a-1396 | rs1511802 | C | T | 4 | 187150806 | -0.1895 | 5.01E-14 | 0.0252 | 0.38174 | -7.51984127 | 56.54801272 |
| BPI fold-containing family B member 1 || id:prot-a-268 | rs28894750 | T | A | 19 | 49213531 | 0.1567 | 1.38E-10 | 0.0244 | 0.53494 | 6.422131148 | 41.24376848 |
| BPI fold-containing family B member 1 || id:prot-a-268 | rs2424961 | T | C | 20 | 31694060 | -0.4366 | 3.89E-71 | 0.0245 | 0.57858 | -17.82040816 | 317.5669471 |
| Brain-derived neurotrophic factor || id:prot-a-242 | rs6993770 | T | A | 8 | 106581528 | -0.1852 | 1.26E-11 | 0.0273 | 0.28225 | -6.783882784 | 46.02106563 |
| Brain-specific serine protease 4 || id:prot-a-2391 | rs73355007 | T | C | 7 | 57259939 | 0.6674 | 1.29E-08 | 0.1174 | 0.01137 | 5.68483816 | 32.31738491 |
| Brain-specific serine protease 4 || id:prot-a-2391 | rs3810801 | A | C | 16 | 2892370 | 0.3066 | 3.72E-33 | 0.0256 | 0.33282 | 11.9765625 | 143.4380493 |
| Breast cancer anti-estrogen resistance protein 3 || id:prot-a-234 | rs62143198 | A | G | 19 | 54320939 | 0.5361 | 7.59E-75 | 0.0293 | 0.21453 | 18.29692833 | 334.7775862 |
| Brevican core protein || id:prot-a-232 | rs2365715 | G | A | 1 | 156615114 | -0.2024 | 4.07E-13 | 0.0279 | 0.39614 | -7.254480287 | 52.62748423 |
| BRICHOS domain-containing protein 5 || id:prot-a-272 | rs62143198 | A | G | 19 | 54320939 | 0.5378 | 2.45E-75 | 0.0293 | 0.21453 | 18.35494881 | 336.9041457 |
| Bromodomain-containing protein 1 || id:prot-a-269 | rs4632248 | T | G | 19 | 54324995 | 0.4698 | 2.88E-59 | 0.0289 | 0.21439 | 16.25605536 | 264.259336 |
| Brorin || id:prot-a-3216 | rs148681119 | A | C | 7 | 49757758 | 0.7535 | 2.34E-18 | 0.0862 | 0.02264 | 8.741299304 | 76.41031352 |
| Brorin || id:prot-a-3216 | rs77263941 | T | C | 7 | 49748311 | -0.4396 | 2.34E-08 | 0.0787 | 0.02504 | -5.585768742 | 31.20081244 |
| Brorin || id:prot-a-3216 | rs79259707 | A | C | 7 | 49812564 | -0.5094 | 1.35E-47 | 0.0352 | 0.14742 | -14.47159091 | 209.4269434 |
| Brother of CDO || id:prot-a-263 | rs775222 | C | G | 3 | 113003595 | 0.1816 | 2.14E-08 | 0.0324 | 0.17717 | 5.604938272 | 31.41533303 |
| C-C motif chemokine 1 || id:prot-a-388 | rs570618 | G | T | 1 | 196657064 | -0.1478 | 5.62E-09 | 0.0254 | 0.61149 | -5.818897638 | 33.85956972 |
| C-C motif chemokine 1 || id:prot-a-388 | rs74480769 | G | A | 5 | 40972211 | -0.4306 | 4.47E-09 | 0.0734 | 0.0317 | -5.866485014 | 34.41564642 |
| C-C motif chemokine 14 || id:prot-a-390 | rs13962 | A | G | 1 | 159175527 | 0.2424 | 5.75E-13 | 0.0336 | 0.17136 | 7.214285714 | 52.04591837 |
| C-C motif chemokine 14 || id:prot-a-390 | rs9903158 | C | T | 17 | 34312337 | -1.0813 | 3.55E-104 | 0.0499 | 0.05615 | -21.66933868 | 469.5602387 |
| C-C motif chemokine 14 || id:prot-a-390 | rs34111696 | G | A | 17 | 34350659 | -0.5139 | 1.20E-18 | 0.0583 | 0.04984 | -8.814751286 | 77.69984024 |
| C-C motif chemokine 15 || id:prot-a-391 | rs28929474 | T | C | 14 | 94844947 | 0.5086 | 1.15E-09 | 0.0835 | 0.02269 | 6.091017964 | 37.10049984 |
| C-C motif chemokine 15 || id:prot-a-391 | rs854628 | A | C | 17 | 34329822 | -1.6963 | 1.00E-200 | 0.0391 | 0.93021 | -43.38363171 | 1882.139501 |
| C-C motif chemokine 15 || id:prot-a-391 | rs681343 | T | C | 19 | 49206462 | -0.2614 | 2.19E-27 | 0.0241 | 0.50746 | -10.84647303 | 117.6459772 |
| C-C motif chemokine 15 || id:prot-a-392 | rs854628 | A | C | 17 | 34329822 | -1.592 | 1.00E-200 | 0.0404 | 0.93021 | -39.40594059 | 1552.828154 |
| C-C motif chemokine 15 || id:prot-a-392 | rs601338 | A | G | 19 | 49206674 | -0.2347 | 3.47E-22 | 0.0242 | 0.50711 | -9.698347107 | 94.05793662 |
| C-C motif chemokine 16 || id:prot-a-393 | rs112689088 | C | T | 17 | 34307457 | -1.5245 | 1.00E-200 | 0.0336 | 0.09753 | -45.37202381 | 2058.620545 |
| C-C motif chemokine 17 || id:prot-a-394 | rs112751691 | G | A | 3 | 16937239 | -0.397 | 4.57E-10 | 0.0637 | 0.03931 | -6.232339089 | 38.84205053 |
| C-C motif chemokine 17 || id:prot-a-394 | rs2228467 | C | T | 3 | 42906116 | 0.5899 | 6.92E-34 | 0.0486 | 0.06675 | 12.13786008 | 147.3276474 |
| C-C motif chemokine 17 || id:prot-a-394 | rs6993770 | T | A | 8 | 106581528 | -0.212 | 7.76E-15 | 0.0273 | 0.28225 | -7.765567766 | 60.30404272 |
| C-C motif chemokine 17 || id:prot-a-394 | rs10886430 | G | A | 10 | 121010256 | -0.2352 | 3.63E-10 | 0.0375 | 0.14121 | -6.272 | 39.337984 |
| C-C motif chemokine 17 || id:prot-a-394 | rs223895 | C | T | 16 | 57440896 | -0.1564 | 1.00E-09 | 0.0256 | 0.6547 | -6.109375 | 37.32446289 |
| C-C motif chemokine 17 || id:prot-a-394 | rs113022368 | A | AT | 16 | 57442335 | -0.4494 | 1.58E-21 | 0.0472 | 0.9218 | -9.521186441 | 90.65299124 |
| C-C motif chemokine 19 || id:prot-a-395 | rs13064576 | T | C | 3 | 49642430 | -0.2365 | 2.04E-19 | 0.0262 | 0.30396 | -9.026717557 | 81.48162986 |
| C-C motif chemokine 19 || id:prot-a-395 | rs116763083 | A | T | 6 | 32569932 | 0.6269 | 3.80E-16 | 0.077 | 0.02622 | 8.141558442 | 66.28497386 |
| C-C motif chemokine 21 || id:prot-a-397 | rs11924792 | C | A | 3 | 132240100 | -0.6072 | 1.45E-57 | 0.038 | 0.10951 | -15.97894737 | 255.326759 |
| C-C motif chemokine 21 || id:prot-a-397 | rs12722013 | C | T | 6 | 33048460 | 0.4523 | 1.26E-15 | 0.0566 | 0.0488 | 7.991166078 | 63.85873528 |
| C-C motif chemokine 22 || id:prot-a-398 | rs79928291 | T | C | 16 | 57562973 | -0.8502 | 5.62E-16 | 0.105 | 0.01585 | -8.097142857 | 65.56372245 |
| C-C motif chemokine 22 || id:prot-a-398 | rs1787782 | G | C | 16 | 57341568 | -0.3809 | 3.80E-10 | 0.0608 | 0.04465 | -6.264802632 | 39.24775201 |
| C-C motif chemokine 22 || id:prot-a-398 | rs4550435 | G | A | 16 | 57349794 | -0.3359 | 1.23E-11 | 0.0496 | 0.93299 | -6.772177419 | 45.862387 |
| C-C motif chemokine 22 || id:prot-a-398 | rs77542162 | G | A | 17 | 67081278 | 0.8314 | 7.24E-22 | 0.0865 | 0.02254 | 9.611560694 | 92.38209897 |
| C-C motif chemokine 23 || id:prot-a-399 | rs72830000 | A | G | 17 | 34312581 | 1.0154 | 1.45E-89 | 0.0506 | 0.05558 | 20.06719368 | 402.692262 |
| C-C motif chemokine 23 || id:prot-a-399 | rs80219613 | T | C | 17 | 34340325 | -1.3859 | 2.40E-40 | 0.1042 | 0.01382 | -13.30038388 | 176.9002113 |
| C-C motif chemokine 23 || id:prot-a-399 | rs7207185 | A | G | 17 | 34350776 | -0.4147 | 1.12E-11 | 0.0611 | 0.04588 | -6.787234043 | 46.06654595 |
| C-C motif chemokine 24 || id:prot-a-401 | rs10778863 | T | A | 12 | 82516610 | -0.1368 | 4.27E-08 | 0.025 | 0.51363 | -5.472 | 29.942784 |
| C-C motif chemokine 24 || id:prot-a-401 | rs62143196 | G | A | 19 | 54320636 | -0.2002 | 3.47E-11 | 0.0302 | 0.22404 | -6.629139073 | 43.94548485 |
| C-C motif chemokine 25 || id:prot-a-402 | rs550057 | T | C | 9 | 136146597 | -0.1973 | 4.07E-12 | 0.0285 | 0.24577 | -6.922807018 | 47.925257 |
| C-C motif chemokine 25 || id:prot-a-402 | rs214099 | C | T | 11 | 17291210 | -0.1932 | 1.66E-14 | 0.0252 | 0.59374 | -7.666666667 | 58.77777778 |
| C-C motif chemokine 25 || id:prot-a-402 | rs62124688 | G | A | 19 | 8120498 | 0.4716 | 3.72E-48 | 0.0323 | 0.16682 | 14.6006192 | 213.1780809 |
| C-C motif chemokine 25 || id:prot-a-402 | rs35106244 | T | C | 19 | 49203829 | -0.1986 | 5.50E-15 | 0.0254 | 0.48491 | -7.818897638 | 61.13516027 |
| C-C motif chemokine 25 || id:prot-a-403 | rs550057 | T | C | 9 | 136146597 | -0.199 | 2.75E-12 | 0.0285 | 0.24577 | -6.98245614 | 48.75469375 |
| C-C motif chemokine 25 || id:prot-a-403 | rs214068 | C | T | 11 | 17305650 | -0.1646 | 8.51E-11 | 0.0254 | 0.58786 | -6.480314961 | 41.99448199 |
| C-C motif chemokine 25 || id:prot-a-403 | rs35106244 | T | C | 19 | 49203829 | -0.1642 | 1.20E-10 | 0.0255 | 0.48491 | -6.439215686 | 41.46349865 |
| C-C motif chemokine 25 || id:prot-a-403 | rs74959615 | A | G | 19 | 8121096 | -0.9718 | 5.25E-114 | 0.0428 | 0.07829 | -22.70560748 | 515.5446109 |
| C-C motif chemokine 25 || id:prot-a-403 | rs10424096 | G | A | 19 | 8135170 | 0.3909 | 6.61E-20 | 0.0428 | 0.09548 | 9.13317757 | 83.41493253 |
| C-C motif chemokine 26 || id:prot-a-404 | rs12075 | A | G | 1 | 159175354 | 0.1521 | 1.95E-09 | 0.0253 | 0.5838 | 6.011857708 | 36.1424331 |
| C-C motif chemokine 27 || id:prot-a-405 | rs12551313 | T | G | 9 | 34646340 | -0.4077 | 3.72E-08 | 0.0741 | 0.02797 | -5.502024291 | 30.2722713 |
| C-C motif chemokine 27 || id:prot-a-405 | rs135556 | T | C | 22 | 46543485 | 0.1804 | 6.76E-11 | 0.0276 | 0.71338 | 6.536231884 | 42.72232724 |
| C-C motif chemokine 28 || id:prot-a-406 | rs12694039 | G | A | 2 | 206953057 | -0.2471 | 1.10E-23 | 0.0246 | 0.42666 | -10.04471545 | 100.8963084 |
| C-C motif chemokine 28 || id:prot-a-406 | rs4759076 | C | T | 12 | 54729872 | 0.2005 | 5.37E-16 | 0.0247 | 0.44943 | 8.117408907 | 65.89232736 |
| C-C motif chemokine 3-like 1 || id:prot-a-408 | rs74480769 | G | A | 5 | 40972211 | -0.4274 | 5.75E-09 | 0.0734 | 0.0317 | -5.822888283 | 33.90602796 |
| C-C motif chemokine 3-like 1 || id:prot-a-408 | rs2015086 | G | A | 17 | 34391617 | 0.496 | 3.09E-42 | 0.0364 | 0.13145 | 13.62637363 | 185.6780582 |
| C-C motif chemokine 3-like 1 || id:prot-a-408 | rs1719175 | C | T | 17 | 34333388 | -0.19 | 2.82E-09 | 0.032 | 0.81016 | -5.9375 | 35.25390625 |
| C-C motif chemokine 3 || id:prot-a-407 | rs854682 | A | T | 17 | 34314334 | 0.1843 | 2.00E-09 | 0.0307 | 0.20061 | 6.003257329 | 36.03909856 |
| C-C motif chemokine 3 || id:prot-a-407 | rs712042 | A | G | 17 | 34392880 | -0.7144 | 1.35E-94 | 0.0346 | 0.86235 | -20.64739884 | 426.315079 |
| C-C motif chemokine 5 || id:prot-a-409 | rs7080386 | A | C | 10 | 65048306 | 0.1467 | 6.46E-09 | 0.0253 | 0.41587 | 5.798418972 | 33.62166258 |
| C-C motif chemokine 5 || id:prot-a-409 | rs2291299 | C | T | 17 | 34191406 | -0.2126 | 2.82E-11 | 0.0319 | 0.1768 | -6.664576803 | 44.41658396 |
| C-C motif chemokine 7 || id:prot-a-410 | rs1133763 | C | A | 17 | 32647831 | -0.5878 | 7.08E-72 | 0.0328 | 0.15824 | -17.92073171 | 321.1526249 |
| C-C motif chemokine 8 || id:prot-a-411 | rs1133763 | C | A | 17 | 32647831 | -0.2309 | 1.29E-11 | 0.0341 | 0.15824 | -6.771260997 | 45.84997549 |
| C-reactive protein || id:prot-a-670 | rs28480230 | T | C | 1 | 66124075 | -0.1829 | 5.01E-13 | 0.0253 | 0.36232 | -7.229249012 | 52.26204128 |
| C-reactive protein || id:prot-a-670 | rs2808628 | A | G | 1 | 159676011 | -0.182 | 5.37E-12 | 0.0264 | 0.33393 | -6.893939394 | 47.52640037 |
| C-reactive protein || id:prot-a-670 | rs2259852 | A | G | 12 | 121434833 | -0.1619 | 3.47E-10 | 0.0258 | 0.34328 | -6.275193798 | 39.37805721 |
| C-reactive protein || id:prot-a-670 | rs814573 | T | A | 19 | 45424351 | -0.2143 | 1.35E-11 | 0.0317 | 0.1977 | -6.760252366 | 45.70101205 |
| C-type lectin domain family 10 member A || id:prot-a-568 | rs62143196 | G | A | 19 | 54320636 | -0.1766 | 5.37E-09 | 0.0303 | 0.22404 | -5.828382838 | 33.97004651 |
| C-type lectin domain family 12 member A || id:prot-a-570 | rs7968634 | A | C | 12 | 10089806 | -0.3022 | 1.26E-12 | 0.0426 | 0.0923 | -7.093896714 | 50.32337058 |
| C-type lectin domain family 12 member A || id:prot-a-570 | rs479499 | C | A | 12 | 10137557 | -1.2562 | 1.00E-200 | 0.0132 | 0.64024 | -95.16666667 | 9056.694444 |
| C-type lectin domain family 2 member A || id:prot-a-572 | rs1243420 | A | G | 10 | 9000404 | 0.1414 | 4.57E-08 | 0.0259 | 0.44079 | 5.459459459 | 29.80569759 |
| C-type lectin domain family 2 member D || id:prot-a-574 | rs704 | A | G | 17 | 26694861 | 0.2877 | 1.23E-32 | 0.0242 | 0.46665 | 11.88842975 | 141.334762 |
| C-type lectin domain family 2 member L || id:prot-a-575 | rs1042779 | G | A | 3 | 52821011 | 0.1677 | 5.50E-11 | 0.0256 | 0.36871 | 6.55078125 | 42.91273499 |
| C-type lectin domain family 4 member D || id:prot-a-576 | rs1501815 | T | A | 5 | 40960166 | -0.1586 | 3.98E-08 | 0.0289 | 0.24772 | -5.487889273 | 30.11692868 |
| C-type lectin domain family 4 member E || id:prot-a-577 | rs28577986 | A | G | 14 | 106200232 | 0.2113 | 5.75E-14 | 0.0281 | 0.27359 | 7.519572954 | 56.54397741 |
| C-type lectin domain family 4 member K || id:prot-a-425 | rs4632248 | T | G | 19 | 54324995 | -0.2417 | 4.68E-16 | 0.0298 | 0.21439 | -8.110738255 | 65.78407504 |
| C-type lectin domain family 4 member M || id:prot-a-578 | rs33944729 | T | TA | 1 | 196652124 | 0.186 | 1.82E-13 | 0.0253 | 0.38701 | 7.351778656 | 54.04864941 |
| C-type lectin domain family 5 member A || id:prot-a-579 | rs7080536 | A | G | 10 | 115348046 | -1.0066 | 6.03E-63 | 0.0601 | 0.04384 | -16.74875208 | 280.5206962 |
| C-type lectin domain family 6 member A || id:prot-a-580 | rs7258858 | T | A | 19 | 54321872 | 0.1595 | 1.02E-08 | 0.0279 | 0.71275 | 5.716845878 | 32.68232679 |
| C-type lectin domain family 7 member A || id:prot-a-581 | rs4845342 | A | C | 1 | 153210808 | -0.1953 | 9.77E-09 | 0.034 | 0.15467 | -5.744117647 | 32.99488754 |
| C-type mannose receptor 2 || id:prot-a-1938 | rs4055121 | T | C | 11 | 126232337 | -0.2238 | 2.88E-10 | 0.0355 | 0.14038 | -6.304225352 | 39.74325729 |
| C-type mannose receptor 2 || id:prot-a-1938 | rs146385050 | A | C | 17 | 60637258 | -0.2188 | 1.29E-11 | 0.0323 | 0.19505 | -6.773993808 | 45.88699211 |
| C-type mannose receptor 2 || id:prot-a-1938 | rs975527 | A | G | 21 | 18049199 | -0.1719 | 8.32E-09 | 0.0298 | 0.24931 | -5.768456376 | 33.27508896 |
| C-X-C motif chemokine 11 || id:prot-a-739 | rs10031452 | C | T | 4 | 76924933 | -0.2328 | 2.40E-21 | 0.0245 | 0.52873 | -9.502040816 | 90.28877968 |
| C-X-C motif chemokine 11 || id:prot-a-739 | rs2393969 | C | A | 10 | 65140440 | 0.1385 | 3.47E-08 | 0.0251 | 0.47282 | 5.517928287 | 30.44753258 |
| C-X-C motif chemokine 16 || id:prot-a-745 | rs55711612 | G | A | 2 | 111845422 | 0.1582 | 3.47E-08 | 0.0287 | 0.2565 | 5.512195122 | 30.38429506 |
| C-X-C motif chemokine 16 || id:prot-a-745 | rs13317619 | C | T | 3 | 46099437 | -0.1724 | 1.05E-08 | 0.0301 | 0.20553 | -5.727574751 | 32.80511253 |
| C-X-C motif chemokine 16 || id:prot-a-745 | rs144830084 | T | A | 17 | 4618101 | -0.2245 | 1.38E-15 | 0.0281 | 0.25565 | -7.989323843 | 63.82929547 |
| C-X-C motif chemokine 16 || id:prot-a-745 | rs8080014 | C | T | 17 | 4671566 | -0.1457 | 6.76E-09 | 0.0251 | 0.42534 | -5.804780876 | 33.69548102 |
| C-X-C motif chemokine 16 || id:prot-a-745 | rs62143197 | A | G | 19 | 54320716 | -0.1949 | 1.23E-10 | 0.0303 | 0.22311 | -6.432343234 | 41.37503948 |
| C-X-C motif chemokine 5 || id:prot-a-748 | rs425535 | C | T | 4 | 74863997 | -0.2359 | 2.82E-10 | 0.0374 | 0.88107 | -6.307486631 | 39.7843876 |
| C-X-C motif chemokine 5 || id:prot-a-748 | rs62143196 | G | A | 19 | 54320636 | -0.2044 | 1.32E-11 | 0.0302 | 0.22404 | -6.768211921 | 45.8086926 |
| C1GALT1-specific chaperone 1 || id:prot-a-294 | rs9267920 | T | C | 6 | 32206243 | -0.1793 | 4.57E-08 | 0.0328 | 0.17033 | -5.466463415 | 29.88222226 |
| C1GALT1-specific chaperone 1 || id:prot-a-294 | rs7787942 | T | A | 7 | 7270012 | 0.2696 | 1.02E-28 | 0.0242 | 0.44073 | 11.14049587 | 124.1106482 |
| C1GALT1-specific chaperone 1 || id:prot-a-294 | rs2519093 | T | C | 9 | 136141870 | 0.6106 | 1.91E-89 | 0.0305 | 0.17823 | 20.01967213 | 400.7872722 |
| Cadherin-1 || id:prot-a-480 | rs34436714 | A | C | 19 | 54327313 | -0.1815 | 1.55E-09 | 0.03 | 0.21294 | -6.05 | 36.6025 |
| Cadherin-12 || id:prot-a-478 | rs3093692 | A | G | 17 | 26670627 | -0.1969 | 5.13E-16 | 0.0243 | 0.50816 | -8.102880658 | 65.65667496 |
| Cadherin-15 || id:prot-a-481 | rs4241818 | C | T | 4 | 187153786 | -0.2877 | 2.51E-32 | 0.0243 | 0.51359 | -11.83950617 | 140.1739064 |
| Cadherin-15 || id:prot-a-481 | rs1801020 | G | A | 5 | 176836532 | -0.2222 | 3.47E-15 | 0.0282 | 0.75032 | -7.879432624 | 62.08545848 |
| Cadherin-2 || id:prot-a-483 | rs380003 | C | T | 10 | 119200307 | -0.1452 | 7.94E-09 | 0.0252 | 0.42701 | -5.761904762 | 33.19954649 |
| Cadherin-3 || id:prot-a-484 | rs4241819 | T | C | 4 | 187157140 | 0.1515 | 9.12E-10 | 0.0247 | 0.50721 | 6.133603239 | 37.62108869 |
| Cadherin-3 || id:prot-a-484 | rs4795433 | T | C | 17 | 26716821 | 0.2971 | 3.80E-35 | 0.024 | 0.50963 | 12.37916667 | 153.2437674 |
| Cadherin-5 || id:prot-a-485 | rs8176746 | T | G | 9 | 136131322 | 1.1894 | 1.23E-153 | 0.045 | 0.06663 | 26.43111111 | 698.6036346 |
| Cadherin-5 || id:prot-a-485 | rs2289150 | A | G | 16 | 66442499 | 0.1632 | 1.82E-10 | 0.0256 | 0.62508 | 6.375 | 40.640625 |
| Cadherin-7 || id:prot-a-486 | rs72708013 | G | T | 1 | 169481731 | -0.6111 | 5.01E-30 | 0.0537 | 0.05472 | -11.37988827 | 129.501857 |
| Cadherin-7 || id:prot-a-486 | rs2223303 | T | C | 1 | 169574887 | -0.2628 | 2.63E-24 | 0.0258 | 0.33944 | -10.18604651 | 103.7555435 |
| Cadherin-7 || id:prot-a-486 | rs3132514 | C | T | 6 | 31142758 | 0.1777 | 3.47E-09 | 0.0301 | 0.20938 | 5.903654485 | 34.85313628 |
| Cadherin-7 || id:prot-a-486 | rs10159712 | A | G | 10 | 20260369 | 0.2603 | 1.02E-21 | 0.0272 | 0.70648 | 9.569852941 | 91.58208532 |
| Cadherin-related family member 1 || id:prot-a-487 | rs17580 | A | T | 14 | 94847262 | 0.8347 | 9.77E-52 | 0.0552 | 0.04977 | 15.12137681 | 228.6560367 |
| Cadherin-related family member 3 || id:prot-a-488 | rs10168787 | C | G | 2 | 35552428 | 0.1842 | 1.66E-08 | 0.0326 | 0.17966 | 5.650306748 | 31.92596635 |
| Cadherin-related family member 3 || id:prot-a-488 | rs111844913 | C | A | 10 | 7761745 | -0.2959 | 4.47E-08 | 0.0541 | 0.0569 | -5.469500924 | 29.91544036 |
| Cadherin-related family member 3 || id:prot-a-488 | rs7315927 | A | T | 12 | 101101073 | -0.2035 | 8.32E-09 | 0.0353 | 0.8441 | -5.764872521 | 33.23375519 |
| Cadherin-related family member 5 || id:prot-a-490 | rs7412 | T | C | 19 | 45412079 | -0.4743 | 3.55E-25 | 0.0458 | 0.07776 | -10.3558952 | 107.2445653 |
| Calbindin || id:prot-a-340 | rs35145884 | CA | C | 13 | 46650531 | -0.3351 | 1.35E-36 | 0.0265 | 0.42264 | -12.64528302 | 159.9031826 |
| Calbindin || id:prot-a-340 | rs9567617 | G | C | 13 | 46651108 | 0.5463 | 4.90E-109 | 0.0246 | 0.32393 | 22.20731707 | 493.1649316 |
| Calcineurin B homologous protein 1 || id:prot-a-546 | rs11068300 | C | T | 12 | 117482277 | 0.1517 | 5.01E-09 | 0.0259 | 0.33656 | 5.857142857 | 34.30612245 |
| Calcineurin B homologous protein 1 || id:prot-a-546 | rs10418046 | G | T | 19 | 54327869 | 0.1857 | 4.68E-10 | 0.0298 | 0.21727 | 6.231543624 | 38.83213594 |
| Calcineurin B homologous protein 3 || id:prot-a-2947 | rs704 | A | G | 17 | 26694861 | 0.1484 | 1.55E-09 | 0.0246 | 0.46665 | 6.032520325 | 36.39130147 |
| Calcineurin subunit B type 1 || id:prot-a-2350 | rs7539005 | T | A | 1 | 196667252 | -0.1679 | 3.98E-11 | 0.0254 | 0.61028 | -6.61023622 | 43.69522289 |
| Calcineurin subunit B type 1 || id:prot-a-2350 | rs74480769 | G | A | 5 | 40972211 | -0.4523 | 6.92E-10 | 0.0733 | 0.0317 | -6.17053206 | 38.0754659 |
| Calcineurin subunit B type 1 || id:prot-a-2350 | rs3032928 | CTTT | C | 17 | 26679563 | -0.1498 | 1.82E-09 | 0.0249 | 0.45639 | -6.016064257 | 36.19302914 |
| Calcipressin-1 || id:prot-a-2509 | rs145548733 | A | G | 2 | 89266416 | -0.338 | 5.13E-14 | 0.0449 | 0.08475 | -7.527839644 | 56.6683697 |
| Calcipressin-1 || id:prot-a-2509 | rs704 | A | G | 17 | 26694861 | 0.1971 | 7.76E-16 | 0.0245 | 0.46665 | 8.044897959 | 64.72038317 |
| Calcium/calmodulin-dependent 3',5'-cyclic nucleotide phosphodiesterase 1A || id:prot-a-2218 | rs1972498 | A | G | 17 | 5951088 | 0.1563 | 4.79E-08 | 0.0286 | 0.73165 | 5.465034965 | 29.86660717 |
| Calcium/calmodulin-dependent protein kinase type 1 || id:prot-a-346 | rs4525 | C | T | 1 | 169511734 | -1.0486 | 1.00E-200 | 0.0215 | 0.26297 | -48.77209302 | 2378.717058 |
| Calcium/calmodulin-dependent protein kinase type 1 || id:prot-a-346 | rs61751507 | T | C | 10 | 101829514 | 0.654 | 1.70E-28 | 0.0591 | 0.04472 | 11.06598985 | 122.4561313 |
| Calcium/calmodulin-dependent protein kinase type 1 || id:prot-a-346 | rs72706368 | A | G | 14 | 100100230 | 0.6514 | 2.24E-10 | 0.1027 | 0.01614 | 6.342745862 | 40.23042507 |
| Calcium/calmodulin-dependent protein kinase type 1D || id:prot-a-347 | rs10922103 | A | G | 1 | 196671118 | -0.1799 | 1.12E-12 | 0.0253 | 0.61067 | -7.110671937 | 50.56165539 |
| Calcium/calmodulin-dependent protein kinase type 1D || id:prot-a-347 | rs74480769 | G | A | 5 | 40972211 | -0.4927 | 1.78E-11 | 0.0733 | 0.0317 | -6.721691678 | 45.18113901 |
| Calcium/calmodulin-dependent protein kinase type 1D || id:prot-a-347 | rs704 | A | G | 17 | 26694861 | 0.2943 | 3.89E-34 | 0.0242 | 0.46665 | 12.16115702 | 147.8937402 |
| Calcium/calmodulin-dependent protein kinase type II subunit alpha || id:prot-a-348 | rs4632248 | T | G | 19 | 54324995 | 0.4331 | 4.90E-50 | 0.0291 | 0.21439 | 14.88316151 | 221.5084966 |
| Calcium/calmodulin-dependent protein kinase type II subunit beta || id:prot-a-349 | rs62143206 | T | G | 19 | 54326212 | 0.562 | 5.25E-87 | 0.0284 | 0.2131 | 19.78873239 | 391.5939298 |
| Calcyclin-binding protein || id:prot-a-336 | rs62143198 | A | G | 19 | 54320939 | 0.5739 | 9.77E-87 | 0.0291 | 0.21453 | 19.72164948 | 388.9434584 |
| Calnexin || id:prot-a-353 | rs67908756 | G | T | 1 | 196821380 | -0.2346 | 3.89E-14 | 0.031 | 0.19374 | -7.567741935 | 57.270718 |
| Calpain-2 catalytic subunit || id:prot-a-354 | rs150394890 | T | G | 1 | 223864380 | -1.074 | 1.10E-13 | 0.1446 | 0.00791 | -7.427385892 | 55.16606119 |
| Calpain-2 catalytic subunit || id:prot-a-354 | rs17599 | C | A | 1 | 223954080 | -0.1721 | 2.57E-09 | 0.0289 | 0.24007 | -5.955017301 | 35.46223106 |
| Calpain-2 catalytic subunit || id:prot-a-354 | rs5030088 | G | A | 3 | 186460222 | 0.4006 | 1.58E-09 | 0.0664 | 0.03612 | 6.03313253 | 36.39868813 |
| Calpastatin || id:prot-a-366 | rs61751507 | T | C | 10 | 101829514 | -0.6688 | 7.41E-30 | 0.0589 | 0.04472 | -11.35483871 | 128.9323621 |
| Calpastatin || id:prot-a-366 | rs57510498 | G | A | 11 | 87982859 | -0.1415 | 4.68E-08 | 0.0259 | 0.62068 | -5.463320463 | 29.84787048 |
| Calpastatin || id:prot-a-366 | rs704 | A | G | 17 | 26694861 | -0.1405 | 1.07E-08 | 0.0246 | 0.46665 | -5.711382114 | 32.61988565 |
| Calpastatin || id:prot-a-366 | rs709012 | G | T | 20 | 3675498 | 0.1486 | 2.95E-09 | 0.025 | 0.59971 | 5.944 | 35.331136 |
| Calsyntenin-3 || id:prot-a-594 | rs72795155 | T | C | 10 | 53301063 | -0.2698 | 2.88E-08 | 0.0486 | 0.07076 | -5.551440329 | 30.81848973 |
| cAMP-dependent protein kinase catalytic subunit alpha || id:prot-a-2367 | rs10418046 | G | T | 19 | 54327869 | 0.2894 | 1.23E-22 | 0.0296 | 0.21727 | 9.777027027 | 95.59025749 |
| cAMP-dependent protein kinase type I-beta regulatory subunit || id:prot-a-2368 | rs10418046 | G | T | 19 | 54327869 | 0.318 | 3.80E-27 | 0.0295 | 0.21727 | 10.77966102 | 116.2010916 |
| cAMP-regulated phosphoprotein 19 || id:prot-a-167 | rs62143198 | A | G | 19 | 54320939 | 0.5263 | 6.03E-72 | 0.0293 | 0.21453 | 17.96245734 | 322.6498736 |
| cAMP-specific 3',5'-cyclic phosphodiesterase 4D || id:prot-a-2221 | rs41542719 | C | T | 6 | 31239593 | -0.1938 | 3.24E-14 | 0.0255 | 0.36703 | -7.6 | 57.76 |
| cAMP-specific 3',5'-cyclic phosphodiesterase 4D || id:prot-a-2221 | rs9378193 | C | T | 6 | 31293942 | -0.4231 | 8.51E-27 | 0.0395 | 0.14196 | -10.71139241 | 114.7339273 |
| cAMP-specific 3',5'-cyclic phosphodiesterase 4D || id:prot-a-2221 | rs1611236 | A | G | 6 | 29748690 | -0.1509 | 1.35E-08 | 0.0266 | 0.31728 | -5.672932331 | 32.18216123 |
| cAMP-specific 3',5'-cyclic phosphodiesterase 4D || id:prot-a-2221 | rs3132451 | C | G | 6 | 31582025 | -0.4602 | 7.08E-56 | 0.0292 | 0.21656 | -15.76027397 | 248.3862357 |
| cAMP-specific 3',5'-cyclic phosphodiesterase 4D || id:prot-a-2221 | rs200374399 | A | T | 6 | 32482335 | 0.2195 | 1.15E-16 | 0.0265 | 0.50165 | 8.283018868 | 68.60840157 |
| Carbohydrate sulfotransferase 1 || id:prot-a-554 | rs7247412 | C | T | 19 | 52313868 | -0.1948 | 3.80E-12 | 0.0281 | 0.27059 | -6.932384342 | 48.05795266 |
| Carbohydrate sulfotransferase 11 || id:prot-a-550 | rs6993770 | T | A | 8 | 106581528 | -0.1646 | 1.82E-09 | 0.0274 | 0.28225 | -6.00729927 | 36.08764452 |
| Carbohydrate sulfotransferase 11 || id:prot-a-550 | rs8176645 | A | T | 9 | 136149098 | 0.1817 | 2.57E-12 | 0.026 | 0.33926 | 6.988461538 | 48.83859467 |
| Carbohydrate sulfotransferase 11 || id:prot-a-550 | rs111789728 | C | T | 12 | 104851112 | -0.244 | 1.12E-09 | 0.0401 | 0.11521 | -6.08478803 | 37.02464537 |
| Carbohydrate sulfotransferase 11 || id:prot-a-550 | rs1650132 | G | A | 12 | 104981657 | -0.3161 | 3.89E-39 | 0.0242 | 0.48419 | -13.06198347 | 170.6154122 |
| Carbohydrate sulfotransferase 15 || id:prot-a-552 | rs550057 | T | C | 9 | 136146597 | -0.2927 | 3.24E-25 | 0.0282 | 0.24577 | -10.37943262 | 107.7326216 |
| Carbohydrate sulfotransferase 15 || id:prot-a-552 | rs7247412 | C | T | 19 | 52313868 | -0.1538 | 4.57E-08 | 0.0281 | 0.27059 | -5.473309609 | 29.95711807 |
| Carbohydrate sulfotransferase 15 || id:prot-a-553 | rs550057 | T | C | 9 | 136146597 | -0.3239 | 1.02E-30 | 0.0281 | 0.24577 | -11.52669039 | 132.8645914 |
| Carbohydrate sulfotransferase 15 || id:prot-a-553 | rs7247412 | C | T | 19 | 52313868 | -0.1613 | 9.77E-09 | 0.0281 | 0.27059 | -5.740213523 | 32.95005129 |
| Carbohydrate sulfotransferase 3 || id:prot-a-556 | rs10424405 | G | A | 19 | 54321933 | -0.2025 | 1.86E-11 | 0.0301 | 0.2203 | -6.727574751 | 45.26026203 |
| Carbohydrate sulfotransferase 5 || id:prot-a-557 | rs142255518 | C | A | 14 | 106782037 | 0.4919 | 3.63E-08 | 0.0893 | 0.02328 | 5.508398656 | 30.34245576 |
| Carbohydrate sulfotransferase 9 || id:prot-a-559 | rs9952639 | C | G | 18 | 24709604 | 0.3331 | 7.24E-37 | 0.0263 | 0.31788 | 12.66539924 | 160.4123379 |
| Carbonic anhydrase-related protein 10 || id:prot-a-326 | rs12740374 | T | G | 1 | 109817590 | -0.2979 | 2.09E-23 | 0.0299 | 0.22252 | -9.963210702 | 99.2655675 |
| Carbonic anhydrase-related protein 10 || id:prot-a-326 | rs117399000 | A | G | 17 | 50213731 | -0.5939 | 3.02E-19 | 0.0662 | 0.03744 | -8.971299094 | 80.48420743 |
| Carbonic anhydrase-related protein 10 || id:prot-a-326 | rs2938139 | A | G | 17 | 50179416 | -0.1484 | 1.15E-08 | 0.026 | 0.64965 | -5.707692308 | 32.57775148 |
| Carbonic anhydrase-related protein 10 || id:prot-a-327 | rs74480769 | G | A | 5 | 40972211 | -0.517 | 1.66E-12 | 0.0732 | 0.0317 | -7.06284153 | 49.88373048 |
| Carbonic anhydrase-related protein 10 || id:prot-a-327 | rs117506641 | C | G | 16 | 56693581 | -0.4527 | 2.29E-08 | 0.081 | 0.02651 | -5.588888889 | 31.23567901 |
| Carbonic anhydrase 1 || id:prot-a-328 | rs147156208 | G | C | 8 | 86445351 | -0.8025 | 3.09E-10 | 0.1275 | 0.00916 | -6.294117647 | 39.61591696 |
| Carbonic anhydrase 1 || id:prot-a-328 | rs34655806 | C | T | 15 | 75362586 | -0.1752 | 1.66E-09 | 0.0291 | 0.23338 | -6.020618557 | 36.24784781 |
| Carbonic anhydrase 3 || id:prot-a-330 | rs75737704 | A | G | 4 | 153230984 | -0.5371 | 1.15E-08 | 0.0941 | 0.01782 | -5.707757705 | 32.57849801 |
| Carbonic anhydrase 3 || id:prot-a-330 | rs2072696 | C | G | 8 | 86351051 | -0.2169 | 6.76E-14 | 0.0289 | 0.23803 | -7.505190311 | 56.32788161 |
| Carbonic anhydrase 4 || id:prot-a-331 | rs570618 | G | T | 1 | 196657064 | -0.1599 | 2.75E-10 | 0.0253 | 0.61149 | -6.320158103 | 39.94439844 |
| Carbonic anhydrase 4 || id:prot-a-331 | rs74480769 | G | A | 5 | 40972211 | -0.5771 | 2.88E-15 | 0.0731 | 0.0317 | -7.894664843 | 62.32573298 |
| Carbonic anhydrase 5A, mitochondrial || id:prot-a-332 | rs1157745 | T | G | 7 | 94941038 | 0.2174 | 1.91E-15 | 0.0274 | 0.28335 | 7.934306569 | 62.95322074 |
| Carbonic anhydrase 6 || id:prot-a-333 | rs79066421 | A | G | 1 | 8997062 | 0.2363 | 5.13E-15 | 0.0302 | 0.21517 | 7.824503311 | 61.22285207 |
| Carbonic anhydrase 6 || id:prot-a-333 | rs3765963 | G | A | 1 | 9034598 | 0.6766 | 4.07E-185 | 0.0233 | 0.40236 | 29.03862661 | 843.2418354 |
| Carbonic anhydrase 9 || id:prot-a-334 | rs138851862 | A | C | 3 | 124821528 | 0.3823 | 4.17E-08 | 0.0697 | 0.03236 | 5.484935438 | 30.08451675 |
| Carbonic anhydrase 9 || id:prot-a-334 | rs16990766 | A | G | 22 | 23023396 | 0.1735 | 1.12E-09 | 0.0285 | 0.26284 | 6.087719298 | 37.06032625 |
| Carbonic anhydrase 9 || id:prot-a-334 | rs5751516 | T | G | 22 | 23040767 | 0.1592 | 4.79E-08 | 0.0292 | 0.41488 | 5.452054795 | 29.72490148 |
| Carbonyl reductase [NADPH] 1 || id:prot-a-373 | rs62143197 | A | G | 19 | 54320716 | 0.2228 | 1.70E-13 | 0.0302 | 0.22311 | 7.377483444 | 54.42726196 |
| Carbonyl reductase [NADPH] 1 || id:prot-a-373 | rs16993864 | A | C | 21 | 37446599 | -1.2151 | 6.61E-49 | 0.0827 | 0.02198 | -14.69286578 | 215.8803048 |
| Carbonyl reductase [NADPH] 1 || id:prot-a-373 | rs4817770 | T | C | 21 | 37461348 | 0.2867 | 3.16E-14 | 0.0378 | 0.12323 | 7.584656085 | 57.52700792 |
| Carbonyl reductase [NADPH] 3 || id:prot-a-374 | rs1028997 | A | G | 21 | 37532222 | -0.764 | 1.00E-200 | 0.0222 | 0.37555 | -34.41441441 | 1184.351919 |
| Carboxypeptidase A2 || id:prot-a-642 | rs919791 | A | G | 19 | 15576817 | -0.3304 | 1.41E-27 | 0.0304 | 0.21427 | -10.86842105 | 118.1225762 |
| Carboxypeptidase A2 || id:prot-a-642 | rs5167 | G | T | 19 | 45448465 | 0.2951 | 3.98E-31 | 0.0254 | 0.34696 | 11.61811024 | 134.9804855 |
| Carboxypeptidase A4 || id:prot-a-643 | rs34587586 | T | G | 7 | 129938598 | -1.1658 | 1.00E-200 | 0.0154 | 0.39274 | -75.7012987 | 5730.686625 |
| Carboxypeptidase A4 || id:prot-a-643 | rs117052523 | T | C | 7 | 130070242 | -0.5409 | 5.75E-10 | 0.0873 | 0.02198 | -6.195876289 | 38.38888298 |
| Carboxypeptidase B || id:prot-a-644 | rs13318853 | A | G | 3 | 148562399 | 0.2563 | 2.00E-18 | 0.0293 | 0.23152 | 8.747440273 | 76.51771133 |
| Carboxypeptidase B || id:prot-a-644 | rs72802342 | A | C | 16 | 75234872 | -0.4145 | 8.51E-20 | 0.0455 | 0.08312 | -9.10989011 | 82.99009781 |
| Carboxypeptidase M || id:prot-a-645 | rs1908671 | C | G | 12 | 69433404 | -0.1859 | 8.13E-12 | 0.0272 | 0.29212 | -6.834558824 | 46.71119431 |
| Carboxypeptidase M || id:prot-a-645 | rs34667640 | CT | C | 20 | 25458562 | -0.1475 | 3.31E-09 | 0.0249 | 0.42745 | -5.923694779 | 35.09015984 |
| Carboxypeptidase Z || id:prot-a-649 | rs2631738 | G | A | 4 | 8479754 | 0.1949 | 1.02E-15 | 0.0243 | 0.51937 | 8.020576132 | 64.32964148 |
| Carcinoembryonic antigen-related cell adhesion molecule 1 || id:prot-a-502 | rs3019565 | C | T | 11 | 60859990 | 0.1671 | 3.09E-08 | 0.0302 | 0.23755 | 5.533112583 | 30.61533485 |
| Carcinoembryonic antigen-related cell adhesion molecule 1 || id:prot-a-502 | rs10136766 | G | A | 14 | 106232585 | -0.7653 | 1.00E-200 | 0.0244 | 0.66538 | -31.3647541 | 983.7477997 |
| Cardiotrophin-1 || id:prot-a-710 | rs429358 | C | T | 19 | 45411941 | 0.7994 | 3.47E-148 | 0.0308 | 0.15301 | 25.95454545 | 673.6384298 |
| Cardiotrophin-1 || id:prot-a-710 | rs34827707 | A | G | 19 | 45541782 | -0.6324 | 2.19E-15 | 0.0797 | 0.02784 | -7.934755332 | 62.96034219 |
| Cardiotrophin-1 || id:prot-a-710 | rs144311893 | T | C | 19 | 45423944 | -0.9059 | 8.51E-27 | 0.0845 | 0.02301 | -10.72071006 | 114.9336242 |
| Cardiotrophin-1 || id:prot-a-711 | rs74480769 | G | A | 5 | 40972211 | -0.4373 | 2.51E-09 | 0.0734 | 0.0317 | -5.957765668 | 35.49497175 |
| Cartilage intermediate layer protein 1 || id:prot-a-560 | rs62143198 | A | G | 19 | 54320939 | 0.3556 | 3.55E-32 | 0.0301 | 0.21453 | 11.81395349 | 139.569497 |
| Cas scaffolding protein family member 4 || id:prot-a-365 | rs10418046 | G | T | 19 | 54327869 | 0.166 | 2.63E-08 | 0.0298 | 0.21727 | 5.570469799 | 31.03013378 |
| Casein kinase I isoform delta || id:prot-a-693 | rs438811 | T | C | 19 | 45416741 | -0.1604 | 3.47E-08 | 0.0291 | 0.23219 | -5.512027491 | 30.38244707 |
| Casein kinase I isoform gamma-2 || id:prot-a-694 | rs62143197 | A | G | 19 | 54320716 | 0.3244 | 2.45E-27 | 0.0299 | 0.22311 | 10.84949833 | 117.711614 |
| Casein kinase II 2-alpha:2-beta heterotetramer || id:prot-a-697 | rs61900292 | T | C | 11 | 110748408 | 0.138 | 3.47E-08 | 0.025 | 0.47035 | 5.52 | 30.4704 |
| Casein kinase II 2-alpha:2-beta heterotetramer || id:prot-a-697 | rs4890634 | T | C | 18 | 43854259 | -0.1627 | 1.32E-08 | 0.0286 | 0.74889 | -5.688811189 | 32.36257274 |
| Caspase-10 || id:prot-a-362 | rs62143197 | A | G | 19 | 54320716 | 0.2736 | 9.55E-20 | 0.0301 | 0.22311 | 9.089700997 | 82.62266421 |
| Caspase-3 || id:prot-a-364 | rs10418046 | G | T | 19 | 54327869 | 0.2842 | 7.24E-22 | 0.0296 | 0.21727 | 9.601351351 | 92.18594777 |
| Catalase || id:prot-a-367 | rs2745924 | T | G | 11 | 34399185 | 0.1894 | 2.69E-14 | 0.0249 | 0.54506 | 7.606425703 | 57.85771197 |
| Cathepsin B || id:prot-a-718 | rs1692819 | A | G | 8 | 11705448 | 0.4246 | 5.25E-54 | 0.0274 | 0.29182 | 15.49635036 | 240.1368746 |
| Cathepsin F || id:prot-a-722 | rs1260326 | C | T | 2 | 27730940 | -0.1642 | 6.92E-11 | 0.0252 | 0.59849 | -6.515873016 | 42.45660116 |
| Cathepsin F || id:prot-a-722 | rs1791679 | A | C | 11 | 66337874 | 0.2349 | 2.51E-18 | 0.0269 | 0.28947 | 8.732342007 | 76.25379693 |
| Cathepsin F || id:prot-a-722 | rs10745925 | C | T | 12 | 102218899 | -0.2874 | 9.12E-27 | 0.0268 | 0.29402 | -10.7238806 | 115.0016151 |
| Cathepsin H || id:prot-a-724 | rs7150060 | T | G | 14 | 74953967 | -0.231 | 6.92E-20 | 0.0253 | 0.59185 | -9.130434783 | 83.36483932 |
| Cathepsin H || id:prot-a-724 | rs185905398 | A | G | 14 | 74155957 | 0.7459 | 6.76E-09 | 0.1287 | 0.01047 | 5.795648796 | 33.58954496 |
| Cathepsin H || id:prot-a-725 | rs34593439 | A | G | 15 | 79234957 | -1.147 | 1.00E-200 | 0.0346 | 0.11066 | -33.15028902 | 1098.941662 |
| Cathepsin H || id:prot-a-725 | rs62013235 | A | G | 15 | 79246166 | 0.402 | 1.35E-20 | 0.0432 | 0.091 | 9.305555556 | 86.5933642 |
| Cathepsin L2 || id:prot-a-728 | rs199544945 | A | AT | 4 | 146928659 | 0.1502 | 4.37E-08 | 0.0274 | 0.66171 | 5.481751825 | 30.04960307 |
| Cathepsin L2 || id:prot-a-728 | rs10817163 | A | T | 9 | 99902524 | 0.1983 | 7.08E-11 | 0.0304 | 0.22045 | 6.523026316 | 42.54987232 |
| Cathepsin S || id:prot-a-727 | rs41271951 | G | A | 1 | 150737220 | -0.8605 | 7.08E-94 | 0.0419 | 0.08349 | -20.53699284 | 421.7680749 |
| Cathepsin Z || id:prot-a-729 | rs10745925 | C | T | 12 | 102218899 | -0.3624 | 2.29E-42 | 0.0266 | 0.29402 | -13.62406015 | 185.615015 |
| Cathepsin Z || id:prot-a-729 | rs148370779 | T | C | 20 | 57602212 | -1.7632 | 2.57E-57 | 0.1105 | 0.01316 | -15.95656109 | 254.6118417 |
| Cation-dependent mannose-6-phosphate receptor || id:prot-a-1823 | rs570618 | G | T | 1 | 196657064 | -0.1589 | 3.55E-10 | 0.0253 | 0.61149 | -6.280632411 | 39.44634348 |
| Cation-dependent mannose-6-phosphate receptor || id:prot-a-1823 | rs74480769 | G | A | 5 | 40972211 | -0.4595 | 3.63E-10 | 0.0733 | 0.0317 | -6.268758527 | 39.29733346 |
| Cation-dependent mannose-6-phosphate receptor || id:prot-a-1823 | rs967645 | T | C | 17 | 26713970 | 0.2263 | 9.55E-21 | 0.0242 | 0.50945 | 9.351239669 | 87.44568335 |
| Cation-independent mannose-6-phosphate receptor || id:prot-a-1445 | rs111918389 | C | T | 6 | 160368234 | 0.559 | 3.80E-16 | 0.0686 | 0.03345 | 8.148688047 | 66.40111688 |
| Cation-independent mannose-6-phosphate receptor || id:prot-a-1445 | rs629849 | G | A | 6 | 160494409 | 0.8992 | 9.55E-163 | 0.0331 | 0.87065 | 27.16616314 | 738.0004199 |
| Cation-independent mannose-6-phosphate receptor || id:prot-a-1445 | rs117010682 | T | C | 6 | 160559348 | 0.853 | 9.33E-14 | 0.1145 | 0.01167 | 7.449781659 | 55.49924677 |
| Cation-independent mannose-6-phosphate receptor || id:prot-a-1445 | rs117901488 | T | C | 6 | 160603132 | -0.3402 | 6.03E-09 | 0.0585 | 0.04691 | -5.815384615 | 33.81869822 |
| Cation-independent mannose-6-phosphate receptor || id:prot-a-1445 | rs17777713 | T | C | 6 | 160361357 | 0.5969 | 1.48E-08 | 0.1054 | 0.01567 | 5.663187856 | 32.07169669 |
| Cation-independent mannose-6-phosphate receptor || id:prot-a-1445 | rs9272202 | T | C | 6 | 32601885 | 0.2244 | 1.95E-10 | 0.0352 | 0.15014 | 6.375 | 40.640625 |
| Cation-independent mannose-6-phosphate receptor || id:prot-a-1445 | rs2442483 | C | G | 8 | 6348967 | -0.174 | 4.47E-08 | 0.0318 | 0.80291 | -5.471698113 | 29.93948024 |
| CD109 antigen || id:prot-a-418 | rs6903575 | A | G | 6 | 74503730 | 0.4146 | 6.46E-69 | 0.0236 | 0.52155 | 17.56779661 | 308.6274777 |
| CD109 antigen || id:prot-a-418 | rs10901252 | C | G | 9 | 136128000 | 0.2888 | 7.24E-09 | 0.0499 | 0.06738 | 5.78757515 | 33.49602612 |
| CD166 antigen || id:prot-a-74 | rs9830049 | C | T | 3 | 105284045 | -0.306 | 2.82E-16 | 0.0374 | 0.12027 | -8.181818182 | 66.94214876 |
| CD166 antigen || id:prot-a-74 | rs62143198 | A | G | 19 | 54320939 | -0.1743 | 1.23E-08 | 0.0306 | 0.21453 | -5.696078431 | 32.4453095 |
| CD177 antigen || id:prot-a-420 | rs113467819 | C | T | 19 | 43588208 | 0.478 | 9.77E-09 | 0.0834 | 0.0262 | 5.731414868 | 32.84911639 |
| CD177 antigen || id:prot-a-420 | rs73554000 | C | G | 19 | 43825494 | 1.8032 | 4.37E-116 | 0.0787 | 0.02383 | 22.91232529 | 524.97465 |
| CD177 antigen || id:prot-a-420 | rs17711542 | C | A | 19 | 43837552 | 0.2763 | 4.07E-28 | 0.0251 | 0.37766 | 11.00796813 | 121.1753623 |
| CD177 antigen || id:prot-a-420 | rs147910249 | T | C | 19 | 43873831 | 0.6379 | 1.51E-13 | 0.0864 | 0.0246 | 7.383101852 | 54.51019295 |
| CD177 antigen || id:prot-a-420 | rs192431294 | T | C | 19 | 43876976 | 0.7112 | 2.14E-23 | 0.0714 | 0.03578 | 9.960784314 | 99.21722414 |
| CD209 antigen || id:prot-a-426 | rs505922 | C | T | 9 | 136149229 | 0.8311 | 1.00E-200 | 0.0224 | 0.31319 | 37.10267857 | 1376.608757 |
| CD209 antigen || id:prot-a-426 | rs3124759 | G | A | 9 | 136342415 | -0.1837 | 2.24E-08 | 0.0328 | 0.18087 | -5.600609756 | 31.36682964 |
| CD209 antigen || id:prot-a-426 | rs151212242 | C | T | 19 | 7782146 | -0.4047 | 7.76E-42 | 0.0299 | 0.21458 | -13.53511706 | 183.1993937 |
| CD226 antigen || id:prot-a-428 | rs1839468 | T | C | 4 | 139524119 | -0.1429 | 4.27E-08 | 0.0261 | 0.34203 | -5.475095785 | 29.97667386 |
| CD226 antigen || id:prot-a-429 | rs62143197 | A | G | 19 | 54320716 | 0.3212 | 8.13E-27 | 0.03 | 0.22311 | 10.70666667 | 114.6327111 |
| CD48 antigen || id:prot-a-451 | rs12124234 | C | G | 1 | 160675269 | 0.2639 | 1.15E-25 | 0.0252 | 0.40515 | 10.47222222 | 109.6674383 |
| CD48 antigen || id:prot-a-451 | rs7259081 | A | T | 19 | 54339243 | 0.1791 | 1.20E-08 | 0.0314 | 0.75126 | 5.703821656 | 32.53358148 |
| CD59 glycoprotein || id:prot-a-455 | rs2273121 | A | G | 11 | 33757770 | -0.362 | 4.68E-41 | 0.027 | 0.2516 | -13.40740741 | 179.7585734 |
| CD59 glycoprotein || id:prot-a-455 | rs35703183 | C | T | 15 | 32975513 | -0.1512 | 2.75E-08 | 0.0272 | 0.28777 | -5.558823529 | 30.90051903 |
| CD63 antigen || id:prot-a-456 | rs10754199 | G | A | 1 | 196670839 | -0.172 | 1.07E-11 | 0.0253 | 0.61068 | -6.798418972 | 46.21850052 |
| CD63 antigen || id:prot-a-456 | rs4253272 | T | C | 4 | 187163614 | 0.2451 | 1.91E-23 | 0.0246 | 0.51236 | 9.963414634 | 99.26963117 |
| CD63 antigen || id:prot-a-456 | rs2545801 | C | T | 5 | 176841339 | 0.2481 | 5.50E-19 | 0.0279 | 0.75043 | 8.892473118 | 79.07607816 |
| CD97 antigen || id:prot-a-471 | rs570618 | G | T | 1 | 196657064 | -0.1674 | 3.80E-11 | 0.0253 | 0.61149 | -6.616600791 | 43.77940602 |
| CD97 antigen || id:prot-a-471 | rs74480769 | G | A | 5 | 40972211 | -0.5454 | 8.91E-14 | 0.0731 | 0.0317 | -7.461012312 | 55.66670472 |
| CDGSH iron-sulfur domain-containing protein 1 || id:prot-a-562 | rs136169 | G | A | 22 | 36661149 | -0.3115 | 1.74E-23 | 0.0312 | 0.8124 | -9.983974359 | 99.679744 |
| Cell adhesion molecule-related/down-regulated by oncogenes || id:prot-a-499 | rs3740910 | G | A | 11 | 125889673 | -0.8271 | 4.37E-86 | 0.0421 | 0.08611 | -19.64608076 | 385.9684892 |
| Cell cycle progression protein 1 || id:prot-a-417 | rs528298 | T | A | 1 | 196660995 | -0.1655 | 6.61E-11 | 0.0254 | 0.61415 | -6.515748031 | 42.45497241 |
| Cell cycle progression protein 1 || id:prot-a-417 | rs74480769 | G | A | 5 | 40972211 | -0.4891 | 2.40E-11 | 0.0732 | 0.0317 | -6.681693989 | 44.64503456 |
| Cell growth regulator with EF hand domain protein 1 || id:prot-a-532 | rs10803264 | G | A | 1 | 236236446 | -0.4773 | 3.55E-08 | 0.0866 | 0.02761 | -5.511547344 | 30.37715413 |
| Cell growth regulator with EF hand domain protein 1 || id:prot-a-532 | rs6809081 | C | T | 3 | 194060475 | 0.2415 | 1.12E-21 | 0.0252 | 0.42319 | 9.583333333 | 91.84027778 |
| Cell growth regulator with EF hand domain protein 1 || id:prot-a-532 | rs927826 | T | G | 10 | 20220846 | 0.1657 | 1.20E-09 | 0.0273 | 0.70585 | 6.06959707 | 36.84000859 |
| Cell growth regulator with EF hand domain protein 1 || id:prot-a-532 | rs61751507 | T | C | 10 | 101829514 | -0.5444 | 4.57E-20 | 0.0593 | 0.04472 | -9.180438449 | 84.28045011 |
| Cell surface glycoprotein CD200 receptor 1 || id:prot-a-423 | rs147866349 | G | T | 1 | 200017256 | -0.8254 | 3.47E-09 | 0.1397 | 0.00932 | -5.908375089 | 34.9088962 |
| Cell surface glycoprotein CD200 receptor 1 || id:prot-a-423 | rs6791672 | A | G | 3 | 112591392 | -0.1759 | 2.75E-12 | 0.0252 | 0.59062 | -6.98015873 | 48.7226159 |
| Cell surface glycoprotein CD200 receptor 2 || id:prot-a-424 | rs113451861 | A | T | 4 | 138574488 | -0.3645 | 3.09E-08 | 0.0658 | 0.04271 | -5.539513678 | 30.68621179 |
| Cell surface glycoprotein CD200 receptor 2 || id:prot-a-424 | rs7412 | T | C | 19 | 45412079 | 0.7212 | 1.82E-58 | 0.0447 | 0.07776 | 16.13422819 | 260.3133192 |
| Cellular retinoic acid-binding protein 1 || id:prot-a-650 | rs74480769 | G | A | 5 | 40972211 | -0.532 | 3.63E-13 | 0.0732 | 0.0317 | -7.267759563 | 52.82032906 |
| Cellular retinoic acid-binding protein 1 || id:prot-a-650 | rs6505079 | A | T | 17 | 26718177 | 0.2268 | 8.13E-21 | 0.0242 | 0.51071 | 9.371900826 | 87.8325251 |
| Cellular retinoic acid-binding protein 2 || id:prot-a-651 | rs13402560 | G | C | 2 | 3639915 | -0.1908 | 6.03E-09 | 0.0328 | 0.81367 | -5.817073171 | 33.83834027 |
| Cellular tumor antigen p53 || id:prot-a-3080 | rs74480769 | G | A | 5 | 40972211 | -0.4211 | 9.55E-09 | 0.0734 | 0.0317 | -5.737057221 | 32.91382555 |
| Cellular tumor antigen p53 || id:prot-a-3080 | rs704 | A | G | 17 | 26694861 | 0.1727 | 1.86E-12 | 0.0245 | 0.46665 | 7.048979592 | 49.68811329 |
| Centrin-2 || id:prot-a-516 | rs62143198 | A | G | 19 | 54320939 | 0.2272 | 9.12E-14 | 0.0305 | 0.21453 | 7.449180328 | 55.49028756 |
| Centromere protein W || id:prot-a-511 | rs72673751 | C | T | 8 | 106578940 | -0.2042 | 1.86E-10 | 0.032 | 0.18949 | -6.38125 | 40.72035156 |
| Centrosomal protein of 57 kDa || id:prot-a-512 | rs1065853 | T | G | 19 | 45413233 | 1.3324 | 1.00E-200 | 0.0403 | 0.07778 | 33.06203474 | 1093.098141 |
| Centrosomal protein of 57 kDa || id:prot-a-514 | rs7257867 | G | A | 19 | 54331892 | 0.1477 | 4.79E-08 | 0.0271 | 0.69072 | 5.450184502 | 29.7045111 |
| Cerebellin-1 || id:prot-a-371 | rs62058003 | A | G | 16 | 48930568 | -0.4423 | 2.88E-09 | 0.0745 | 0.03051 | -5.936912752 | 35.24693302 |
| Cerebellin-1 || id:prot-a-371 | rs10852587 | A | T | 16 | 49006458 | -1.0805 | 1.00E-200 | 0.0327 | 0.14362 | -33.04281346 | 1091.827521 |
| Cerebellin-4 || id:prot-a-372 | rs58548607 | T | C | 4 | 57734591 | 0.1992 | 3.98E-08 | 0.0363 | 0.14406 | 5.487603306 | 30.11379004 |
| Cerebellin-4 || id:prot-a-372 | rs74447607 | T | C | 20 | 54447947 | -0.2177 | 3.02E-12 | 0.0312 | 0.19175 | -6.977564103 | 48.68640081 |
| Cerebellin-4 || id:prot-a-372 | rs6069316 | G | C | 20 | 54136681 | -0.1866 | 1.10E-10 | 0.0289 | 0.23292 | -6.456747405 | 41.68958705 |
| Cerebral cavernous malformations 2 protein || id:prot-a-412 | rs368465 | C | T | 1 | 196671981 | -0.1648 | 9.77E-11 | 0.0255 | 0.61344 | -6.462745098 | 41.7670742 |
| Cerebral cavernous malformations 2 protein || id:prot-a-412 | rs3197999 | A | G | 3 | 49721532 | 0.1571 | 6.76E-09 | 0.0271 | 0.28831 | 5.79704797 | 33.60576517 |
| Cerebral cavernous malformations 2 protein || id:prot-a-412 | rs74480769 | G | A | 5 | 40972211 | -0.4499 | 8.51E-10 | 0.0733 | 0.0317 | -6.137789905 | 37.67246491 |
| Cerebral cavernous malformations 2 protein || id:prot-a-412 | rs74628834 | A | G | 14 | 58102057 | -0.1733 | 4.68E-08 | 0.0317 | 0.19142 | -5.466876972 | 29.88674382 |
| Cerebral dopamine neurotrophic factor || id:prot-a-498 | rs61738953 | G | C | 10 | 14862082 | -0.6293 | 2.51E-20 | 0.0681 | 0.04178 | -9.24082232 | 85.39279715 |
| Cerebral dopamine neurotrophic factor || id:prot-a-498 | rs16978810 | A | C | 21 | 47488332 | 0.3141 | 2.69E-08 | 0.0565 | 0.05343 | 5.559292035 | 30.90572793 |
| cGMP-dependent protein kinase 1, beta isozyme || id:prot-a-2374 | rs3917532 | T | A | 7 | 94940119 | 0.2218 | 5.13E-16 | 0.0274 | 0.28253 | 8.094890511 | 65.52725238 |
| cGMP-dependent protein kinase 1, beta isozyme || id:prot-a-2374 | rs117565083 | A | G | 17 | 64263263 | 0.4028 | 4.37E-08 | 0.0736 | 0.03142 | 5.472826087 | 29.95182538 |
| Charged multivesicular body protein 3 || id:prot-a-3202 | rs62143196 | G | A | 19 | 54320636 | 0.5261 | 1.62E-73 | 0.029 | 0.22404 | 18.14137931 | 329.1096433 |
| Chitinase-3-like protein 1 || id:prot-a-540 | rs35405821 | G | C | 1 | 203150899 | -1.0287 | 1.00E-200 | 0.0176 | 0.52283 | -58.44886364 | 3416.26966 |
| Chitinase-3-like protein 1 || id:prot-a-540 | rs12240123 | C | G | 1 | 203288427 | -0.2841 | 6.92E-13 | 0.0396 | 0.89514 | -7.174242424 | 51.46975436 |
| Chitinase-3-like protein 2 || id:prot-a-541 | rs4261897 | C | T | 3 | 18764304 | -0.1469 | 4.37E-08 | 0.0268 | 0.30042 | -5.481343284 | 30.04512419 |
| Chitinase-3-like protein 2 || id:prot-a-541 | rs704 | A | G | 17 | 26694861 | 0.1866 | 2.51E-14 | 0.0245 | 0.46665 | 7.616326531 | 58.00842982 |
| Chitotriosidase-1 || id:prot-a-543 | rs872583 | C | T | 1 | 203184766 | -1.0982 | 1.00E-200 | 0.0247 | 0.20154 | -44.46153846 | 1976.828402 |
| Chitotriosidase-1 || id:prot-a-543 | rs12411260 | T | A | 1 | 203078793 | 0.1847 | 1.00E-11 | 0.0271 | 0.32536 | 6.815498155 | 46.4510151 |
| Chitotriosidase-1 || id:prot-a-543 | rs7072852 | G | T | 10 | 89445249 | 0.1548 | 4.79E-08 | 0.0284 | 0.25502 | 5.450704225 | 29.71017655 |
| Chloride intracellular channel protein 5 || id:prot-a-584 | rs35822882 | T | G | 6 | 45916999 | -0.7339 | 7.41E-27 | 0.0684 | 0.03365 | -10.72953216 | 115.1228605 |
| Chloride intracellular channel protein 5 || id:prot-a-584 | rs9411377 | A | C | 9 | 136145404 | 0.1737 | 5.01E-10 | 0.0279 | 0.28942 | 6.225806452 | 38.76066597 |
| Choline/ethanolamine kinase || id:prot-a-544 | rs704 | A | G | 17 | 26694861 | -0.2414 | 3.31E-23 | 0.0243 | 0.46665 | -9.934156379 | 98.68746295 |
| Chondroadherin || id:prot-a-533 | rs72967312 | C | G | 11 | 91404397 | 0.4353 | 4.90E-08 | 0.0798 | 0.02459 | 5.454887218 | 29.75579456 |
| Chondroitin sulfate N-acetylgalactosaminyltransferase 2 || id:prot-a-687 | rs2435349 | G | A | 10 | 43643466 | -0.1961 | 1.62E-12 | 0.0278 | 0.27282 | -7.053956835 | 49.75830702 |
| Chordin-like protein 2 || id:prot-a-549 | rs11607100 | T | C | 11 | 74414919 | -0.6419 | 5.75E-26 | 0.0609 | 0.04167 | -10.54022989 | 111.096446 |
| Chordin-like protein 2 || id:prot-a-549 | rs7129492 | G | A | 11 | 74381181 | 0.1751 | 1.10E-12 | 0.0246 | 0.49883 | 7.117886179 | 50.66430366 |
| Chordin || id:prot-a-547 | rs1126477 | T | C | 3 | 46501268 | 0.1927 | 2.95E-11 | 0.029 | 0.23937 | 6.644827586 | 44.15373365 |
| Choriogonadotropin subunit beta variant 2 || id:prot-a-531 | rs6993770 | T | A | 8 | 106581528 | -0.1686 | 7.41E-10 | 0.0274 | 0.28225 | -6.153284672 | 37.86291225 |
| Chromobox protein homolog 5 || id:prot-a-376 | rs368465 | C | T | 1 | 196671981 | -0.1584 | 5.13E-10 | 0.0255 | 0.61344 | -6.211764706 | 38.58602076 |
| Chromobox protein homolog 5 || id:prot-a-376 | rs74480769 | G | A | 5 | 40972211 | -0.5488 | 6.31E-14 | 0.0731 | 0.0317 | -7.50752394 | 56.36291571 |
| Chromobox protein homolog 7 || id:prot-a-377 | rs10424405 | G | A | 19 | 54321933 | 0.4955 | 5.25E-65 | 0.0291 | 0.2203 | 17.02749141 | 289.9354637 |
| Chromodomain-helicase-DNA-binding protein 7 || id:prot-a-536 | rs10418046 | G | T | 19 | 54327869 | -0.1631 | 4.68E-08 | 0.0298 | 0.21727 | -5.473154362 | 29.95541867 |
| Chromogranin-A || id:prot-a-538 | rs3775298 | G | A | 4 | 187150478 | -0.2085 | 2.19E-17 | 0.0246 | 0.51335 | -8.475609756 | 71.83596074 |
| Chymotrypsin-like protease CTRL-1 || id:prot-a-716 | rs74480769 | G | A | 5 | 40972211 | -0.4292 | 4.90E-09 | 0.0734 | 0.0317 | -5.847411444 | 34.1922206 |
| Chymotrypsin-like protease CTRL-1 || id:prot-a-716 | rs62143206 | T | G | 19 | 54326212 | 0.4703 | 2.09E-59 | 0.0289 | 0.2131 | 16.2733564 | 264.8221286 |
| Chymotrypsinogen B || id:prot-a-714 | rs1936809 | C | T | 6 | 127477268 | 0.1427 | 1.02E-08 | 0.0249 | 0.54764 | 5.730923695 | 32.8434864 |
| Chymotrypsinogen B || id:prot-a-714 | rs113434972 | G | A | 16 | 75210084 | 0.1618 | 3.72E-10 | 0.0258 | 0.54802 | 6.271317829 | 39.32942732 |
| Chymotrypsinogen B || id:prot-a-714 | rs8051363 | G | A | 16 | 75255217 | 0.6471 | 1.58E-157 | 0.0242 | 0.70435 | 26.73966942 | 715.0099208 |
| Ciliary neurotrophic factor || id:prot-a-604 | rs77822623 | T | G | 11 | 7715586 | 0.5534 | 2.24E-08 | 0.0989 | 0.02181 | 5.595551062 | 31.31019168 |
| Ciliary neurotrophic factor || id:prot-a-604 | rs62143206 | T | G | 19 | 54326212 | -0.1655 | 3.09E-08 | 0.0299 | 0.2131 | -5.535117057 | 30.63752083 |
| Ciliary neurotrophic factor receptor subunit alpha || id:prot-a-605 | rs10972159 | A | G | 9 | 34593086 | -0.6996 | 1.02E-13 | 0.0941 | 0.0179 | -7.434643996 | 55.27393134 |
| Ck-beta-8-1 || id:prot-a-400 | rs712048 | C | A | 17 | 34326215 | 0.7289 | 1.62E-94 | 0.0353 | 0.87372 | 20.64872521 | 426.3698529 |
| Clathrin interactor 1 || id:prot-a-585 | rs62143198 | A | G | 19 | 54320939 | 0.6893 | 4.90E-131 | 0.0283 | 0.21453 | 24.35689046 | 593.2581128 |
| CMP-N-acetylneuraminate-beta-galactosamide-alpha-2,3-sialyltransferase 1 || id:prot-a-2848 | rs72677638 | C | T | 4 | 117876192 | 0.1754 | 1.58E-08 | 0.031 | 0.20014 | 5.658064516 | 32.01369407 |
| CMP-N-acetylneuraminate-beta-galactosamide-alpha-2,3-sialyltransferase 1 || id:prot-a-2848 | rs9643300 | T | C | 8 | 134503148 | -0.2571 | 1.38E-25 | 0.0246 | 0.56265 | -10.45121951 | 109.2279893 |
| CMRF35-like molecule 6 || id:prot-a-433 | rs79874727 | A | G | 14 | 106370437 | -0.3364 | 4.07E-16 | 0.0413 | 0.14464 | -8.14527845 | 66.34556103 |
| CMRF35-like molecule 6 || id:prot-a-434 | rs370664797 | T | TAC | 12 | 89825211 | 0.4074 | 2.04E-43 | 0.0295 | 0.71484 | 13.81016949 | 190.7207814 |
| CMRF35-like molecule 6 || id:prot-a-434 | rs62087214 | G | A | 17 | 72467626 | -0.6787 | 1.74E-26 | 0.0637 | 0.03851 | -10.65463108 | 113.5211635 |
| CMRF35-like molecule 8 || id:prot-a-432 | rs370664797 | T | TAC | 12 | 89825211 | 0.2299 | 2.04E-14 | 0.0301 | 0.71484 | 7.637873754 | 58.33711548 |
| CMRF35-like molecule 8 || id:prot-a-432 | rs2272111 | A | G | 17 | 72469966 | -0.4499 | 8.32E-51 | 0.03 | 0.2005 | -14.99666667 | 224.9000111 |
| Coactosin-like protein || id:prot-a-636 | rs6993770 | T | A | 8 | 106581528 | -0.1625 | 2.95E-09 | 0.0274 | 0.28225 | -5.930656934 | 35.17269167 |
| Coagulation Factor VIII || id:prot-a-1009 | rs11930928 | A | G | 4 | 5684496 | 0.2846 | 1.66E-08 | 0.0504 | 0.06396 | 5.646825397 | 31.88663706 |
| Coagulation Factor VIII || id:prot-a-1009 | rs9411377 | A | C | 9 | 136145404 | 0.506 | 2.82E-80 | 0.0267 | 0.28942 | 18.95131086 | 359.1521834 |
| Coagulation Factor X || id:prot-a-1006 | rs3762056 | T | C | 9 | 117090434 | -0.5851 | 3.02E-34 | 0.0479 | 0.0816 | -12.21503132 | 149.20699 |
| Coagulation Factor X || id:prot-a-1006 | rs547138 | A | T | 13 | 113792170 | 0.2442 | 8.91E-20 | 0.0268 | 0.61461 | 9.111940299 | 83.027456 |
| Coagulation Factor X || id:prot-a-1006 | rs141217364 | A | C | 13 | 113817084 | -0.3072 | 2.57E-19 | 0.0342 | 0.17451 | -8.98245614 | 80.68451831 |
| Coagulation factor Xa || id:prot-a-1005 | rs1725 | G | A | 2 | 239631937 | 0.5625 | 2.24E-08 | 0.1005 | 0.01649 | 5.597014925 | 31.32657607 |
| Coagulation factor Xa || id:prot-a-1005 | rs3762056 | T | C | 9 | 117090434 | -0.5655 | 5.25E-32 | 0.048 | 0.0816 | -11.78125 | 138.7978516 |
| Coagulation factor Xa || id:prot-a-1005 | rs547138 | A | T | 13 | 113792170 | 0.2581 | 5.75E-22 | 0.0268 | 0.61461 | 9.630597015 | 92.74839886 |
| Coagulation factor Xa || id:prot-a-1005 | rs2287250 | T | C | 13 | 113889474 | 0.2303 | 1.12E-14 | 0.0298 | 0.77671 | 7.728187919 | 59.72488852 |
| Coagulation factor Xa || id:prot-a-1005 | rs112924598 | A | C | 18 | 76368365 | -0.5853 | 2.45E-08 | 0.105 | 0.01646 | -5.574285714 | 31.07266122 |
| Cochlin || id:prot-a-613 | rs6993770 | T | A | 8 | 106581528 | -0.3027 | 3.89E-29 | 0.027 | 0.28225 | -11.21111111 | 125.6890123 |
| Cochlin || id:prot-a-613 | rs6580981 | A | G | 12 | 54723028 | 0.1512 | 1.15E-09 | 0.0248 | 0.45457 | 6.096774194 | 37.17065557 |
| Cochlin || id:prot-a-613 | rs34907608 | A | G | 14 | 31330413 | 0.4759 | 1.05E-38 | 0.0366 | 0.11864 | 13.00273224 | 169.0710457 |
| Cofilin-1 || id:prot-a-524 | rs2668196 | T | A | 3 | 165502709 | -0.178 | 3.47E-08 | 0.0323 | 0.81693 | -5.510835913 | 30.36931246 |
| Cofilin-1 || id:prot-a-524 | rs5167 | G | T | 19 | 45448465 | 0.3477 | 3.98E-43 | 0.0253 | 0.34696 | 13.743083 | 188.8723305 |
| Cofilin-1 || id:prot-a-524 | rs62143194 | G | C | 19 | 54319624 | 0.3859 | 7.59E-38 | 0.03 | 0.22364 | 12.86333333 | 165.4653444 |
| Coiled-coil-helix-coiled-coil-helix domain-containing protein 10, mitochondrial || id:prot-a-534 | rs34436714 | A | C | 19 | 54327313 | 0.7106 | 1.12E-146 | 0.0276 | 0.21294 | 25.74637681 | 662.8759189 |
| Coiled-coil domain-containing protein 126 || id:prot-a-380 | rs73165060 | A | C | 3 | 165481418 | 0.2713 | 1.20E-18 | 0.0308 | 0.20245 | 8.808441558 | 77.58864269 |
| Coiled-coil domain-containing protein 126 || id:prot-a-380 | rs227934 | T | C | 7 | 23627287 | -0.2749 | 6.92E-29 | 0.0246 | 0.47897 | -11.17479675 | 124.8760824 |
| Coiled-coil domain-containing protein 134 || id:prot-a-381 | rs12634004 | G | T | 3 | 21674533 | 0.1632 | 1.58E-08 | 0.0289 | 0.25418 | 5.647058824 | 31.88927336 |
| Coiled-coil domain-containing protein 134 || id:prot-a-381 | rs116765893 | A | T | 6 | 32503445 | 0.2589 | 5.01E-10 | 0.0416 | 0.12414 | 6.223557692 | 38.73267035 |
| Coiled-coil domain-containing protein 134 || id:prot-a-381 | rs34436714 | A | C | 19 | 54327313 | -0.1818 | 1.41E-09 | 0.03 | 0.21294 | -6.06 | 36.7236 |
| Cold-inducible RNA-binding protein || id:prot-a-561 | rs62143197 | A | G | 19 | 54320716 | 0.6581 | 4.07E-120 | 0.0282 | 0.22311 | 23.33687943 | 544.6099417 |
| Cold shock domain-containing protein C2 || id:prot-a-678 | rs1157745 | T | G | 7 | 94941038 | 0.1862 | 1.07E-11 | 0.0274 | 0.28335 | 6.795620438 | 46.18045714 |
| Cold shock domain-containing protein C2 || id:prot-a-678 | rs429358 | C | T | 19 | 45411941 | 0.1927 | 1.07E-08 | 0.0337 | 0.15301 | 5.71810089 | 32.69667779 |
| Colipase || id:prot-a-592 | rs9380534 | A | G | 6 | 35751572 | 0.4476 | 3.47E-71 | 0.0251 | 0.60228 | 17.83266932 | 318.0040952 |
| Colipase || id:prot-a-592 | rs112065360 | A | C | 6 | 35982429 | -0.2172 | 7.08E-10 | 0.0352 | 0.14336 | -6.170454545 | 38.0745093 |
| Colipase || id:prot-a-592 | rs2653414 | A | C | 8 | 17726069 | 0.6574 | 2.51E-11 | 0.0986 | 0.01597 | 6.667342799 | 44.45346 |
| Collagen alpha-1(I) chain || id:prot-a-618 | rs7299271 | T | C | 12 | 48414058 | -0.2806 | 8.71E-29 | 0.0252 | 0.3706 | -11.13492063 | 123.9864575 |
| Collagen alpha-1(I) chain || id:prot-a-618 | rs146385050 | A | C | 17 | 60637258 | 0.2894 | 2.24E-19 | 0.0322 | 0.19505 | 8.98757764 | 80.77655183 |
| Collagen alpha-1(VI) chain || id:prot-a-622 | rs434206 | T | G | 21 | 47366802 | -0.2117 | 5.25E-17 | 0.0253 | 0.59389 | -8.367588933 | 70.01654455 |
| Collagen alpha-1(XV) chain || id:prot-a-616 | rs41305481 | G | A | 9 | 101767385 | 0.2437 | 1.91E-19 | 0.027 | 0.30186 | 9.025925926 | 81.46733882 |
| Collagen alpha-1(XXVIII) chain || id:prot-a-621 | rs1536713 | G | T | 14 | 61396104 | -0.521 | 1.86E-10 | 0.0817 | 0.02396 | -6.376988984 | 40.6659885 |
| Collagen alpha-1(XXVIII) chain || id:prot-a-621 | rs3886281 | A | G | 20 | 42550686 | -0.1597 | 1.55E-08 | 0.0282 | 0.74844 | -5.663120567 | 32.07093456 |
| Collagen alpha-2(IX) chain || id:prot-a-626 | rs28577986 | A | G | 14 | 106200232 | 0.3915 | 5.50E-46 | 0.0275 | 0.27359 | 14.23636364 | 202.6740496 |
| Collagen alpha-2(XI) chain || id:prot-a-615 | rs3130227 | C | T | 6 | 33092537 | 0.1608 | 2.00E-10 | 0.0253 | 0.38279 | 6.355731225 | 40.39531941 |
| Collagen alpha-2(XI) chain || id:prot-a-615 | rs3129205 | T | G | 6 | 33125859 | 0.27 | 2.57E-25 | 0.026 | 0.34777 | 10.38461538 | 107.8402367 |
| Collagen alpha-3(VI) chain || id:prot-a-623 | rs1536713 | G | T | 14 | 61396104 | -0.5086 | 5.01E-10 | 0.0818 | 0.02396 | -6.217603912 | 38.65859841 |
| Collectin-10 || id:prot-a-627 | rs3820897 | C | T | 2 | 3642361 | 0.4223 | 2.19E-38 | 0.0326 | 0.82077 | 12.95398773 | 167.8057981 |
| Collectin-10 || id:prot-a-627 | rs1385496 | A | G | 8 | 120079893 | -0.1558 | 1.32E-09 | 0.0257 | 0.44711 | -6.062256809 | 36.75095762 |
| Collectin-10 || id:prot-a-627 | rs1065853 | T | G | 19 | 45413233 | 0.8081 | 2.95E-74 | 0.0443 | 0.07778 | 18.24153499 | 332.7535987 |
| Collectin-11 || id:prot-a-628 | rs7604806 | G | A | 2 | 3653576 | -0.1968 | 8.13E-15 | 0.0253 | 0.61777 | -7.778656126 | 60.50749113 |
| Collectin-11 || id:prot-a-628 | rs6542680 | T | C | 2 | 3640142 | -1.0196 | 1.00E-200 | 0.0283 | 0.82086 | -36.02826855 | 1298.036135 |
| Collectin-12 || id:prot-a-629 | rs2846667 | G | T | 18 | 466810 | 0.202 | 9.33E-12 | 0.0296 | 0.74548 | 6.824324324 | 46.57140248 |
| Collectin-12 || id:prot-a-629 | rs28823595 | A | G | 20 | 3705133 | 0.3591 | 1.48E-15 | 0.045 | 0.08088 | 7.98 | 63.6804 |
| Collectin-12 || id:prot-a-629 | rs71195856 | CTTTTT | C | 20 | 3681344 | -0.1612 | 2.45E-08 | 0.0289 | 0.27798 | -5.577854671 | 31.11246273 |
| Collectin-12 || id:prot-a-630 | rs62143194 | G | C | 19 | 54319624 | -0.1737 | 1.35E-08 | 0.0306 | 0.22364 | -5.676470588 | 32.22231834 |
| COMM domain-containing protein 7 || id:prot-a-634 | rs10418046 | G | T | 19 | 54327869 | 0.4779 | 8.13E-62 | 0.0288 | 0.21727 | 16.59375 | 275.3525391 |
| Complement C1q-like protein 4 || id:prot-a-302 | rs10424405 | G | A | 19 | 54321933 | -0.1874 | 5.37E-10 | 0.0302 | 0.2203 | -6.205298013 | 38.50572343 |
| Complement C1q and tumor necrosis factor-related protein 9A || id:prot-a-306 | rs7539005 | T | A | 1 | 196667252 | -0.1899 | 7.41E-14 | 0.0254 | 0.61028 | -7.476377953 | 55.89622729 |
| Complement C1q and tumor necrosis factor-related protein 9A || id:prot-a-306 | rs74480769 | G | A | 5 | 40972211 | -0.6455 | 8.32E-19 | 0.0729 | 0.0317 | -8.854595336 | 78.40385857 |
| Complement C1q subcomponent subunit C || id:prot-a-301 | rs78865058 | A | G | 1 | 22944209 | 1.0754 | 4.27E-71 | 0.0603 | 0.04171 | 17.83416252 | 318.0573528 |
| Complement C1q subcomponent subunit C || id:prot-a-301 | rs141126950 | T | C | 1 | 22951801 | 0.9376 | 1.45E-18 | 0.1066 | 0.0136 | 8.795497186 | 77.36077074 |
| Complement C1q subcomponent subunit C || id:prot-a-301 | rs139104615 | T | C | 1 | 22941548 | 0.9372 | 1.38E-15 | 0.1173 | 0.0125 | 7.989769821 | 63.83642179 |
| Complement C1q subcomponent subunit C || id:prot-a-301 | rs4934470 | C | T | 10 | 91067438 | 0.2107 | 1.95E-14 | 0.0275 | 0.73038 | 7.661818182 | 58.70345785 |
| Complement C1q tumor necrosis factor-related protein 1 || id:prot-a-303 | rs646776 | T | C | 1 | 109818530 | 0.6399 | 1.82E-114 | 0.0281 | 0.77654 | 22.77224199 | 518.5750054 |
| Complement C1q tumor necrosis factor-related protein 1 || id:prot-a-303 | rs10762481 | C | T | 10 | 73538164 | -0.1846 | 3.72E-13 | 0.0254 | 0.38274 | -7.267716535 | 52.81970364 |
| Complement C1q tumor necrosis factor-related protein 1 || id:prot-a-303 | rs112635299 | T | G | 14 | 94838142 | -0.584 | 3.16E-12 | 0.0838 | 0.02261 | -6.968973747 | 48.56659509 |
| Complement C1q tumor necrosis factor-related protein 1 || id:prot-a-303 | rs72802395 | A | G | 16 | 75286484 | -0.2547 | 9.77E-09 | 0.0444 | 0.08593 | -5.736486486 | 32.90727721 |
| Complement C1q tumor necrosis factor-related protein 1 || id:prot-a-303 | rs5848 | T | C | 17 | 42430244 | -0.2483 | 1.26E-18 | 0.0282 | 0.28735 | -8.804964539 | 77.52740053 |
| Complement C1q tumor necrosis factor-related protein 1 || id:prot-a-303 | rs7259081 | A | T | 19 | 54339243 | 0.1739 | 3.16E-08 | 0.0314 | 0.75126 | 5.538216561 | 30.67184267 |
| Complement C1q tumor necrosis factor-related protein 3 || id:prot-a-304 | rs840382 | T | C | 5 | 34021706 | 0.1411 | 2.82E-08 | 0.0254 | 0.39412 | 5.55511811 | 30.85933722 |
| Complement C1q tumor necrosis factor-related protein 5 || id:prot-a-305 | rs152197 | T | C | 5 | 131461341 | 0.2156 | 1.12E-10 | 0.0334 | 0.16786 | 6.45508982 | 41.66818459 |
| Complement C1q tumor necrosis factor-related protein 5 || id:prot-a-305 | rs11955347 | A | G | 5 | 131567924 | -0.2464 | 9.12E-24 | 0.0245 | 0.47186 | -10.05714286 | 101.1461224 |
| Complement C1q tumor necrosis factor-related protein 5 || id:prot-a-305 | rs9266141 | G | C | 6 | 31324023 | 0.1755 | 1.58E-08 | 0.031 | 0.23402 | 5.661290323 | 32.05020812 |
| Complement C1q tumor necrosis factor-related protein 5 || id:prot-a-305 | rs2248863 | A | G | 11 | 119207341 | 0.3261 | 3.55E-21 | 0.0345 | 0.14179 | 9.452173913 | 89.34359168 |
| Complement C1q tumor necrosis factor-related protein 5 || id:prot-a-305 | rs61932023 | C | A | 12 | 99025406 | -0.1884 | 3.89E-13 | 0.026 | 0.3319 | -7.246153846 | 52.50674556 |
| Complement C1r subcomponent-like protein || id:prot-a-307 | rs77007122 | C | A | 12 | 7339351 | -1.0601 | 2.34E-37 | 0.083 | 0.02348 | -12.77228916 | 163.1313703 |
| Complement C1r subcomponent-like protein || id:prot-a-307 | rs6488561 | A | G | 12 | 7246894 | -0.2948 | 2.51E-26 | 0.0278 | 0.27278 | -10.60431655 | 112.4515294 |
| Complement C4 || id:prot-a-315 | rs28381349 | T | C | 6 | 31709045 | 0.8152 | 6.46E-17 | 0.0976 | 0.01545 | 8.352459016 | 69.76357162 |
| Complement C4 || id:prot-a-315 | rs12524441 | A | G | 6 | 32024234 | -0.8114 | 9.55E-17 | 0.0976 | 0.01587 | -8.31352459 | 69.11469111 |
| Complement C4 || id:prot-a-315 | rs144515162 | G | A | 6 | 32455083 | 0.4781 | 9.55E-30 | 0.0422 | 0.13145 | 11.32938389 | 128.3549392 |
| Complement C4 || id:prot-a-315 | rs169504 | A | C | 6 | 32153406 | -0.3462 | 1.23E-27 | 0.0318 | 0.19795 | -10.88679245 | 118.5222499 |
| Complement C4 || id:prot-a-315 | rs28858179 | A | T | 6 | 32201790 | 0.4216 | 3.09E-09 | 0.0711 | 0.03928 | 5.929676512 | 35.16106354 |
| Complement C4 || id:prot-a-315 | rs406658 | A | C | 6 | 31996524 | 0.5647 | 3.09E-52 | 0.0371 | 0.11862 | 15.22102426 | 231.6795795 |
| Complement component 1 Q subcomponent-binding protein, mitochondrial || id:prot-a-300 | rs9881048 | C | A | 3 | 165506252 | -0.1747 | 4.79E-08 | 0.032 | 0.8162 | -5.459375 | 29.80477539 |
| Complement component 1 Q subcomponent-binding protein, mitochondrial || id:prot-a-300 | rs967645 | T | C | 17 | 26713970 | 0.1722 | 1.55E-12 | 0.0244 | 0.50945 | 7.057377049 | 49.80657081 |
| Complement component C6 || id:prot-a-318 | rs10179260 | G | A | 2 | 226521752 | -0.2613 | 1.20E-08 | 0.0459 | 0.07932 | -5.692810458 | 32.40809091 |
| Complement component C8 || id:prot-a-322 | rs570618 | G | T | 1 | 196657064 | -0.151 | 2.57E-09 | 0.0253 | 0.61149 | -5.968379447 | 35.62155322 |
| Complement component C8 || id:prot-a-322 | rs74480769 | G | A | 5 | 40972211 | -0.5404 | 1.51E-13 | 0.0732 | 0.0317 | -7.382513661 | 54.50150796 |
| Complement component C8 || id:prot-a-322 | rs117506641 | C | G | 16 | 56693581 | -0.464 | 1.00E-08 | 0.081 | 0.02651 | -5.728395062 | 32.81450998 |
| Complement decay-accelerating factor || id:prot-a-453 | rs11580387 | G | A | 1 | 207418408 | -0.5211 | 4.68E-78 | 0.0279 | 0.23394 | -18.67741935 | 348.8459938 |
| Complement factor B || id:prot-a-518 | rs4657825 | G | A | 1 | 196584321 | -0.2795 | 6.31E-22 | 0.029 | 0.77143 | -9.637931034 | 92.88971463 |
| Complement factor B || id:prot-a-518 | rs4151667 | A | T | 6 | 31914024 | -0.9144 | 5.50E-53 | 0.0597 | 0.04184 | -15.31658291 | 234.5977122 |
| Complement factor B || id:prot-a-518 | rs9272547 | C | T | 6 | 32606949 | 0.1484 | 1.58E-08 | 0.0263 | 0.6664 | 5.642585551 | 31.8387717 |
| Complement factor B || id:prot-a-518 | rs114293611 | C | T | 6 | 32487353 | 0.2075 | 4.27E-13 | 0.0286 | 0.58168 | 7.255244755 | 52.63857646 |
| Complement factor H-related protein 5 || id:prot-a-521 | rs72732234 | C | T | 1 | 196303889 | -1.3018 | 1.70E-44 | 0.093 | 0.01857 | -13.99784946 | 195.9397896 |
| Complement factor H-related protein 5 || id:prot-a-521 | rs61822229 | A | G | 1 | 196774412 | -0.2077 | 8.32E-12 | 0.0304 | 0.2587 | -6.832236842 | 46.67946027 |
| Complement factor H-related protein 5 || id:prot-a-521 | rs35662416 | A | G | 1 | 196967354 | -1.2002 | 3.02E-69 | 0.0682 | 0.03039 | -17.59824047 | 309.6980676 |
| Complement factor H-related protein 5 || id:prot-a-522 | rs1694456 | C | G | 1 | 196814953 | 0.257 | 1.23E-22 | 0.0263 | 0.63857 | 9.771863118 | 95.48930879 |
| Complement factor H-related protein 5 || id:prot-a-522 | rs72732234 | C | T | 1 | 196303889 | -1.5026 | 7.76E-60 | 0.0921 | 0.01857 | -16.31487514 | 266.1751507 |
| Complement factor H || id:prot-a-520 | rs2274700 | A | G | 1 | 196682947 | 0.3665 | 1.58E-51 | 0.0243 | 0.39896 | 15.08230453 | 227.4759098 |
| Complement factor H || id:prot-a-520 | rs140998032 | A | G | 1 | 196906466 | -0.2476 | 1.91E-14 | 0.0323 | 0.17702 | -7.665634675 | 58.76195497 |
| Complement factor I || id:prot-a-523 | rs7439493 | A | G | 4 | 110656730 | 0.3116 | 8.91E-37 | 0.0246 | 0.41596 | 12.66666667 | 160.4444444 |
| Contactin-2 || id:prot-a-607 | rs12043231 | A | G | 1 | 205187271 | -0.2802 | 1.78E-23 | 0.0281 | 0.24737 | -9.971530249 | 99.43141551 |
| Contactin-2 || id:prot-a-607 | rs72755831 | T | C | 1 | 204737718 | 0.4878 | 8.71E-09 | 0.0848 | 0.02284 | 5.752358491 | 33.0896282 |
| Contactin-2 || id:prot-a-607 | rs2071533 | G | T | 1 | 205012198 | -0.7705 | 6.92E-108 | 0.0349 | 0.86518 | -22.0773639 | 487.4099966 |
| Contactin-2 || id:prot-a-607 | rs148411008 | T | A | 2 | 171654272 | -0.307 | 1.86E-08 | 0.0546 | 0.05927 | -5.622710623 | 31.61487475 |
| Contactin-4 || id:prot-a-608 | rs61411916 | G | A | 3 | 3026913 | -0.5809 | 4.17E-12 | 0.0838 | 0.0227 | -6.931980907 | 48.05235929 |
| Contactin-4 || id:prot-a-608 | rs13071423 | A | C | 3 | 2140378 | 0.2638 | 5.37E-16 | 0.0326 | 0.19829 | 8.09202454 | 65.48086115 |
| Contactin-4 || id:prot-a-608 | rs163352 | C | G | 3 | 3098041 | -0.3249 | 2.69E-24 | 0.0319 | 0.81339 | -10.18495298 | 103.7332672 |
| Contactin-5 || id:prot-a-609 | rs28849750 | A | G | 11 | 99004348 | -0.2827 | 1.32E-09 | 0.0466 | 0.9236 | -6.066523605 | 36.80270865 |
| Contactin-5 || id:prot-a-609 | rs4316475 | A | G | 11 | 99051522 | -0.3863 | 2.45E-16 | 0.0471 | 0.92173 | -8.201698514 | 67.26785851 |
| Contactin-5 || id:prot-a-609 | rs34436714 | A | C | 19 | 54327313 | 0.4725 | 1.82E-59 | 0.0291 | 0.21294 | 16.2371134 | 263.6438516 |
| Contactin-associated protein-like 2 || id:prot-a-611 | rs10274393 | C | G | 7 | 145378588 | -0.4332 | 1.15E-60 | 0.0264 | 0.71102 | -16.40909091 | 269.2582645 |
| COP9 signalosome complex subunit 2 || id:prot-a-635 | rs704 | A | G | 17 | 26694861 | 0.6486 | 1.07E-191 | 0.022 | 0.46665 | 29.48181818 | 869.1776033 |
| Copine-1 || id:prot-a-646 | rs62143206 | T | G | 19 | 54326212 | 0.5024 | 2.82E-68 | 0.0288 | 0.2131 | 17.44444444 | 304.308642 |
| Copine-1 || id:prot-a-646 | rs12481228 | C | G | 20 | 34218673 | -0.977 | 1.82E-146 | 0.0379 | 0.09766 | -25.77836412 | 664.5240565 |
| Core-binding factor subunit beta || id:prot-a-369 | rs4632248 | T | G | 19 | 54324995 | 0.3685 | 4.47E-36 | 0.0294 | 0.21439 | 12.53401361 | 157.1014971 |
| Corneodesmosin || id:prot-a-500 | rs12075 | A | G | 1 | 159175354 | 0.2857 | 2.69E-30 | 0.025 | 0.5838 | 11.428 | 130.599184 |
| Corneodesmosin || id:prot-a-500 | rs2228467 | C | T | 3 | 42906116 | 0.3139 | 2.04E-10 | 0.0494 | 0.06675 | 6.354251012 | 40.37650593 |
| Corneodesmosin || id:prot-a-500 | rs9893808 | T | C | 17 | 32561013 | 0.2044 | 2.04E-11 | 0.0305 | 0.20975 | 6.701639344 | 44.9119699 |
| Corneodesmosin || id:prot-a-500 | rs3136674 | C | T | 17 | 32682616 | -0.5195 | 4.90E-21 | 0.0552 | 0.05464 | -9.411231884 | 88.57128558 |
| Cortexin-3 || id:prot-a-730 | rs704 | A | G | 17 | 26694861 | 0.1801 | 1.95E-13 | 0.0245 | 0.46665 | 7.351020408 | 54.03750104 |
| Corticotropin-releasing factor-binding protein || id:prot-a-659 | rs631465 | C | T | 5 | 76129053 | 0.3441 | 3.31E-09 | 0.0582 | 0.95027 | 5.912371134 | 34.95613243 |
| Corticotropin-releasing factor-binding protein || id:prot-a-659 | rs6890905 | T | C | 5 | 76209174 | -0.6838 | 4.17E-192 | 0.0231 | 0.63852 | -29.6017316 | 876.2625138 |
| Creatine kinase M-type || id:prot-a-566 | rs4883275 | C | T | 12 | 7690617 | 0.2442 | 7.59E-10 | 0.0397 | 0.11781 | 6.151133501 | 37.83644335 |
| Creatine kinase M-type || id:prot-a-566 | rs11559024 | C | T | 19 | 45821183 | -0.6788 | 9.12E-16 | 0.0844 | 0.02151 | -8.042654028 | 64.68428382 |
| Creatine kinase M-type || id:prot-a-566 | rs12975366 | C | T | 19 | 54759361 | -0.1443 | 9.55E-09 | 0.0251 | 0.40526 | -5.749003984 | 33.05104681 |
| CREB-binding protein || id:prot-a-655 | rs1799807 | C | T | 3 | 165548529 | -0.5177 | 1.91E-09 | 0.0862 | 0.02086 | -6.005800464 | 36.06963921 |
| CREB-binding protein || id:prot-a-655 | rs6864541 | G | A | 5 | 39364474 | 0.2844 | 6.61E-10 | 0.0461 | 0.07852 | 6.169197397 | 38.05899652 |
| CREB-binding protein || id:prot-a-655 | rs62358178 | G | T | 5 | 39784053 | 0.6692 | 8.51E-14 | 0.0897 | 0.02014 | 7.460423634 | 55.6579208 |
| CREB-binding protein || id:prot-a-655 | rs704 | A | G | 17 | 26694861 | 0.1648 | 1.86E-11 | 0.0245 | 0.46665 | 6.726530612 | 45.24621408 |
| Crk-like protein || id:prot-a-665 | rs62143198 | A | G | 19 | 54320939 | 0.2758 | 1.07E-19 | 0.0304 | 0.21453 | 9.072368421 | 82.30786877 |
| Cryptic protein || id:prot-a-519 | rs76020839 | A | G | 14 | 30179479 | 0.3219 | 4.07E-08 | 0.0587 | 0.04858 | 5.483816014 | 30.07223807 |
| Cryptic protein || id:prot-a-519 | rs62143197 | A | G | 19 | 54320716 | 0.4869 | 4.17E-62 | 0.0293 | 0.22311 | 16.61774744 | 276.14953 |
| CUB and sushi domain-containing protein 1 || id:prot-a-690 | rs1973612 | T | C | 4 | 187169167 | 0.1909 | 1.15E-14 | 0.0247 | 0.5115 | 7.728744939 | 59.73349834 |
| CUB and sushi domain-containing protein 1 || id:prot-a-690 | rs2731672 | C | T | 5 | 176842474 | 0.1569 | 3.89E-08 | 0.0286 | 0.75023 | 5.486013986 | 30.09634945 |
| CUB and sushi domain-containing protein 1 || id:prot-a-690 | rs79712184 | C | T | 10 | 101750613 | -0.3045 | 7.24E-09 | 0.0526 | 0.06536 | -5.788973384 | 33.51221284 |
| CUB and sushi domain-containing protein 1 || id:prot-a-690 | rs4795433 | T | C | 17 | 26716821 | -0.16 | 5.50E-11 | 0.0244 | 0.50963 | -6.557377049 | 42.99919377 |
| CUB and sushi domain-containing protein 2 || id:prot-a-691 | rs34436714 | A | C | 19 | 54327313 | 0.3999 | 3.72E-42 | 0.0294 | 0.21294 | 13.60204082 | 185.0155144 |
| CUB and zona pellucida-like domain-containing protein 1 || id:prot-a-735 | rs151330717 | A | G | 19 | 45196964 | -0.6074 | 9.33E-11 | 0.0938 | 0.02012 | -6.475479744 | 41.93183792 |
| CUB and zona pellucida-like domain-containing protein 1 || id:prot-a-735 | rs201837204 | G | GAA | 19 | 45302949 | -0.9959 | 1.62E-31 | 0.0853 | 0.02566 | -11.67526377 | 136.3117842 |
| CUB and zona pellucida-like domain-containing protein 1 || id:prot-a-735 | rs438811 | T | C | 19 | 45416741 | -0.8243 | 1.00E-200 | 0.0254 | 0.23219 | -32.45275591 | 1053.181366 |
| Cullin-3 || id:prot-a-732 | rs3775298 | G | A | 4 | 187150478 | -0.1502 | 1.17E-09 | 0.0247 | 0.51335 | -6.08097166 | 36.97821633 |
| CXADR-like membrane protein || id:prot-a-587 | rs35483681 | T | C | 11 | 123045730 | 0.2494 | 4.47E-24 | 0.0246 | 0.55365 | 10.13821138 | 102.78333 |
| Cyclic AMP-dependent transcription factor ATF-6 alpha || id:prot-a-197 | rs8111 | T | C | 6 | 32083175 | -0.2237 | 1.07E-15 | 0.0279 | 0.26604 | -8.017921147 | 64.28705952 |
| Cyclic AMP-dependent transcription factor ATF-6 alpha || id:prot-a-197 | rs61738953 | G | C | 10 | 14862082 | 0.7574 | 4.79E-29 | 0.0677 | 0.04178 | 11.18759232 | 125.1622219 |
| Cyclic AMP-dependent transcription factor ATF-6 alpha || id:prot-a-197 | rs6089232 | T | G | 20 | 61082940 | 0.1467 | 8.51E-09 | 0.0255 | 0.59664 | 5.752941176 | 33.09633218 |
| Cyclic AMP-responsive element-binding protein 3-like protein 4 || id:prot-a-654 | rs4845586 | G | T | 1 | 153942597 | 0.2556 | 7.08E-25 | 0.0248 | 0.46994 | 10.30645161 | 106.2229448 |
| Cyclic AMP-responsive element-binding protein 3-like protein 4 || id:prot-a-654 | rs28359800 | CAA | C | 6 | 31267774 | -0.1688 | 4.37E-10 | 0.027 | 0.70451 | -6.251851852 | 39.08565158 |
| Cyclic AMP-responsive element-binding protein 3-like protein 4 || id:prot-a-654 | rs9295987 | G | A | 6 | 31349844 | 0.8747 | 3.09E-69 | 0.0497 | 0.06078 | 17.59959759 | 309.7458352 |
| Cyclin-C || id:prot-a-415 | rs148833901 | T | C | 12 | 68796008 | -0.4962 | 9.33E-09 | 0.0864 | 0.02468 | -5.743055556 | 32.98268711 |
| Cyclin-C || id:prot-a-415 | rs58452280 | C | A | 14 | 106252435 | 0.42 | 7.59E-22 | 0.0437 | 0.1235 | 9.610983982 | 92.3710131 |
| Cyclin-dependent kinase 2:Cyclin-A2 complex || id:prot-a-492 | rs62143196 | G | A | 19 | 54320636 | -0.2001 | 3.55E-11 | 0.0302 | 0.22404 | -6.625827815 | 43.90159423 |
| Cyclin-dependent kinase 5:Cyclin-dependent kinase 5 activator 1 complex || id:prot-a-493 | rs570618 | G | T | 1 | 196657064 | -0.1574 | 5.13E-10 | 0.0253 | 0.61149 | -6.221343874 | 38.70511959 |
| Cyclin-dependent kinase 5:Cyclin-dependent kinase 5 activator 1 complex || id:prot-a-493 | rs74480769 | G | A | 5 | 40972211 | -0.6316 | 4.68E-18 | 0.0729 | 0.0317 | -8.663923182 | 75.06356491 |
| Cyclin-dependent kinase 8:Cyclin-C complex || id:prot-a-494 | rs74480769 | G | A | 5 | 40972211 | -0.5095 | 3.39E-12 | 0.0732 | 0.0317 | -6.960382514 | 48.44692474 |
| Cyclin-dependent kinase 8:Cyclin-C complex || id:prot-a-494 | rs62143206 | T | G | 19 | 54326212 | 0.2619 | 1.26E-18 | 0.0297 | 0.2131 | 8.818181818 | 77.76033058 |
| Cyclin-dependent kinase inhibitor 1B || id:prot-a-495 | rs10424405 | G | A | 19 | 54321933 | 0.5056 | 7.24E-68 | 0.029 | 0.2203 | 17.43448276 | 303.9611891 |
| Cyclin-H || id:prot-a-416 | rs2230641 | G | A | 5 | 86695274 | -0.2386 | 1.23E-14 | 0.0309 | 0.20538 | -7.721682848 | 59.624386 |
| Cystatin-8 || id:prot-a-706 | rs116475746 | G | C | 11 | 71880618 | 0.4616 | 1.35E-21 | 0.0484 | 0.06729 | 9.537190083 | 90.95799467 |
| Cystatin-8 || id:prot-a-706 | rs112689088 | C | T | 17 | 34307457 | -1.1107 | 6.17E-186 | 0.0382 | 0.09753 | -29.07591623 | 845.4089046 |
| Cystatin-F || id:prot-a-705 | rs6993770 | T | A | 8 | 106581528 | -0.1694 | 6.17E-10 | 0.0274 | 0.28225 | -6.182481752 | 38.22308061 |
| Cystatin-F || id:prot-a-705 | rs7269564 | C | T | 20 | 24881393 | 0.2517 | 1.02E-22 | 0.0257 | 0.37776 | 9.793774319 | 95.91801541 |
| Cystatin-F || id:prot-a-705 | rs12480685 | T | C | 20 | 25002763 | 1.3949 | 1.91E-68 | 0.0798 | 0.02395 | 17.47994987 | 305.5486476 |
| Cystatin-F || id:prot-a-705 | rs2387362 | T | C | 20 | 24899906 | 0.7592 | 1.07E-189 | 0.0258 | 0.76552 | 29.42635659 | 865.9104621 |
| Cystatin-M || id:prot-a-703 | rs3825068 | G | A | 11 | 65768093 | 0.5724 | 3.98E-14 | 0.0757 | 0.0278 | 7.561426684 | 57.1751735 |
| Cystatin-M || id:prot-a-704 | rs78924983 | T | C | 1 | 8204596 | -0.3329 | 5.50E-09 | 0.0571 | 0.06277 | -5.830122592 | 33.99032944 |
| Cystatin-M || id:prot-a-704 | rs148479829 | T | C | 11 | 65753833 | 0.5949 | 3.16E-15 | 0.0755 | 0.0281 | 7.879470199 | 62.08605061 |
| Cysteine-rich hydrophobic domain-containing protein 2 || id:prot-a-542 | rs11160165 | T | G | 14 | 94689200 | -0.286 | 9.33E-18 | 0.0333 | 0.16412 | -8.588588589 | 73.76385394 |
| Cysteine-rich hydrophobic domain-containing protein 2 || id:prot-a-542 | rs704 | A | G | 17 | 26694861 | 0.1756 | 7.76E-13 | 0.0245 | 0.46665 | 7.167346939 | 51.37086214 |
| Cysteine-rich motor neuron 1 protein || id:prot-a-660 | rs16873402 | T | C | 8 | 106589247 | -0.1531 | 4.68E-08 | 0.028 | 0.28588 | -5.467857143 | 29.89746173 |
| Cysteine-rich secretory protein 2 || id:prot-a-661 | rs478328 | G | A | 6 | 49720877 | 0.5695 | 4.17E-139 | 0.0227 | 0.45408 | 25.08810573 | 629.413049 |
| Cysteine-rich secretory protein 2 || id:prot-a-661 | rs10046220 | G | C | 6 | 49232616 | -0.5024 | 2.75E-24 | 0.0494 | 0.06549 | -10.17004049 | 103.4297235 |
| Cysteine-rich secretory protein 3 || id:prot-a-662 | rs62143196 | G | A | 19 | 54320636 | -0.2189 | 3.89E-13 | 0.0302 | 0.22404 | -7.248344371 | 52.53849612 |
| Cysteine-rich secretory protein LCCL domain-containing 2 || id:prot-a-663 | rs7896518 | G | A | 10 | 65104500 | -0.1406 | 4.27E-08 | 0.0257 | 0.42021 | -5.470817121 | 29.92983997 |
| Cysteine-rich secretory protein LCCL domain-containing 2 || id:prot-a-663 | rs12921670 | A | G | 16 | 84838761 | 0.2615 | 5.37E-19 | 0.0294 | 0.25699 | 8.894557823 | 79.11315887 |
| Cysteine-rich secretory protein LCCL domain-containing 2 || id:prot-a-663 | rs2646117 | C | A | 16 | 84944838 | -0.1679 | 1.55E-08 | 0.0297 | 0.77364 | -5.653198653 | 31.95865501 |
| Cysteine-rich secretory protein LCCL domain-containing 2 || id:prot-a-663 | rs60910901 | G | A | 16 | 84896257 | -1.0884 | 6.92E-13 | 0.1516 | 0.00805 | -7.179419525 | 51.54406472 |
| Cysteine-rich with EGF-like domain protein 1 || id:prot-a-657 | rs191354630 | A | G | 3 | 10226635 | 0.4824 | 3.24E-14 | 0.0636 | 0.04295 | 7.58490566 | 57.53079388 |
| Cysteine-rich with EGF-like domain protein 1 || id:prot-a-657 | rs34810856 | G | A | 3 | 10119928 | 0.5746 | 1.17E-15 | 0.0718 | 0.03054 | 8.002785515 | 64.044576 |
| Cysteine-rich with EGF-like domain protein 1 || id:prot-a-657 | rs59465469 | C | G | 3 | 9990800 | -1.0872 | 1.00E-200 | 0.0213 | 0.24377 | -51.04225352 | 2605.311645 |
| Cysteine-rich with EGF-like domain protein 1 || id:prot-a-657 | rs141317747 | T | C | 3 | 10354974 | 0.5024 | 3.80E-09 | 0.0853 | 0.02249 | 5.889800703 | 34.68975233 |
| Cytochrome b-c1 complex subunit 7 || id:prot-a-3175 | rs6926771 | A | G | 6 | 149743404 | -0.1759 | 1.38E-08 | 0.031 | 0.19531 | -5.674193548 | 32.19647242 |
| Cytochrome b-c1 complex subunit 7 || id:prot-a-3175 | rs241775 | T | C | 17 | 26644668 | -0.1636 | 1.82E-11 | 0.0243 | 0.47792 | -6.732510288 | 45.32669478 |
| Cytochrome c || id:prot-a-754 | rs13210074 | A | C | 6 | 87822341 | 0.1394 | 3.80E-08 | 0.0253 | 0.3826 | 5.509881423 | 30.35879329 |
| Cytochrome c || id:prot-a-754 | rs4807601 | G | A | 19 | 4547917 | 0.153 | 1.55E-09 | 0.0253 | 0.49293 | 6.04743083 | 36.57141964 |
| Cytochrome c oxidase assembly factor 3 homolog, mitochondrial || id:prot-a-612 | rs10801553 | C | A | 1 | 196655743 | -0.1469 | 7.24E-09 | 0.0254 | 0.61291 | -5.783464567 | 33.4484624 |
| Cytochrome c oxidase assembly factor 3 homolog, mitochondrial || id:prot-a-612 | rs74480769 | G | A | 5 | 40972211 | -0.53 | 4.27E-13 | 0.0731 | 0.0317 | -7.250341997 | 52.56745908 |
| Cytochrome c oxidase assembly factor 3 homolog, mitochondrial || id:prot-a-612 | rs399661 | C | T | 5 | 41267562 | 0.1524 | 5.50E-09 | 0.0261 | 0.33618 | 5.83908046 | 34.09486062 |
| Cytochrome c oxidase subunit 4 isoform 2, mitochondrial || id:prot-a-637 | rs7539005 | T | A | 1 | 196667252 | -0.1931 | 2.75E-14 | 0.0254 | 0.61028 | -7.602362205 | 57.79591109 |
| Cytochrome c oxidase subunit 4 isoform 2, mitochondrial || id:prot-a-637 | rs74480769 | G | A | 5 | 40972211 | -0.5804 | 1.95E-15 | 0.0731 | 0.0317 | -7.939808482 | 63.04055872 |
| Cytochrome c oxidase subunit 6C || id:prot-a-639 | rs10418046 | G | T | 19 | 54327869 | 0.2852 | 4.79E-22 | 0.0296 | 0.21727 | 9.635135135 | 92.83582907 |
| Cytochrome c oxidase subunit 8A, mitochondrial || id:prot-a-641 | rs2232613 | T | C | 20 | 36997655 | -0.9393 | 1.35E-104 | 0.0432 | 0.0768 | -21.74305556 | 472.7604649 |
| Cytochrome c oxidase subunit 8A, mitochondrial || id:prot-a-641 | rs6092380 | A | G | 20 | 36998551 | -0.6401 | 3.63E-08 | 0.1162 | 0.0112 | -5.508605852 | 30.34473843 |
| Cytochrome P450 3A4 || id:prot-a-757 | rs9274901 | T | A | 6 | 32641213 | 0.1699 | 2.63E-08 | 0.0305 | 0.29657 | 5.570491803 | 31.03037893 |
| Cytoglobin || id:prot-a-755 | rs10916376 | A | G | 1 | 229020078 | -0.1433 | 3.72E-08 | 0.026 | 0.3445 | -5.511538462 | 30.37705621 |
| Cytohesin-2 || id:prot-a-759 | rs704 | A | G | 17 | 26694861 | 0.1662 | 1.23E-11 | 0.0245 | 0.46665 | 6.783673469 | 46.01822574 |
| Cytohesin-4 || id:prot-a-760 | rs13184629 | A | C | 5 | 153972821 | 0.2121 | 4.79E-08 | 0.0389 | 0.12183 | 5.452442159 | 29.7291255 |
| Cytohesin-4 || id:prot-a-760 | rs11574991 | G | A | 13 | 46633052 | -0.3166 | 3.55E-10 | 0.0505 | 0.06643 | -6.269306931 | 39.30420939 |
| Cytohesin-4 || id:prot-a-760 | rs62143197 | A | G | 19 | 54320716 | 0.1663 | 4.27E-08 | 0.0303 | 0.22311 | 5.488448845 | 30.12307072 |
| Cytokine receptor common subunit beta || id:prot-a-683 | rs1534881 | A | G | 22 | 37329448 | -0.3158 | 2.63E-38 | 0.0244 | 0.4458 | -12.94262295 | 167.5114888 |
| Cytoskeleton-associated protein 2 || id:prot-a-564 | rs570618 | G | T | 1 | 196657064 | -0.1667 | 4.57E-11 | 0.0253 | 0.61149 | -6.588932806 | 43.41403553 |
| Cytoskeleton-associated protein 2 || id:prot-a-564 | rs142517401 | G | T | 3 | 66161137 | 0.2216 | 3.24E-08 | 0.0401 | 0.1145 | 5.526184539 | 30.53871556 |
| Cytoskeleton-associated protein 2 || id:prot-a-564 | rs74480769 | G | A | 5 | 40972211 | -0.4232 | 7.94E-09 | 0.0734 | 0.0317 | -5.765667575 | 33.24292258 |
| Cytoskeleton-associated protein 2 || id:prot-a-564 | rs2299257 | C | A | 7 | 94942765 | 0.1543 | 2.24E-09 | 0.0258 | 0.38257 | 5.980620155 | 35.76781744 |
| Cytoskeleton-associated protein 2 || id:prot-a-564 | rs967645 | T | C | 17 | 26713970 | 0.1759 | 5.13E-13 | 0.0243 | 0.50945 | 7.238683128 | 52.39853342 |
| Cytoskeleton-associated protein 2 || id:prot-a-564 | rs1065853 | T | G | 19 | 45413233 | 0.5975 | 1.10E-39 | 0.0453 | 0.07778 | 13.18984547 | 173.9720236 |
| Cytosolic non-specific dipeptidase || id:prot-a-600 | rs74480769 | G | A | 5 | 40972211 | -0.4527 | 6.46E-10 | 0.0733 | 0.0317 | -6.175989086 | 38.14284119 |
| Cytosolic non-specific dipeptidase || id:prot-a-600 | rs112492117 | T | C | 12 | 12612313 | -0.2421 | 2.00E-08 | 0.0431 | 0.08925 | -5.617169374 | 31.55259177 |
| D-glucuronyl C5-epimerase || id:prot-a-1216 | rs2519093 | T | C | 9 | 136141870 | 0.5757 | 1.07E-78 | 0.0307 | 0.17823 | 18.752443 | 351.6541183 |
| D-glucuronyl C5-epimerase || id:prot-a-1216 | rs11854180 | T | G | 15 | 69559340 | 0.8286 | 1.00E-200 | 0.0248 | 0.76915 | 33.41129032 | 1116.314321 |
| DCN1-like protein 3 || id:prot-a-773 | rs2931409 | A | G | 5 | 73177096 | -0.137 | 3.31E-08 | 0.0248 | 0.57489 | -5.524193548 | 30.51671436 |
| DCN1-like protein 5 || id:prot-a-774 | rs528298 | T | A | 1 | 196660995 | -0.181 | 8.91E-13 | 0.0253 | 0.61415 | -7.154150198 | 51.18186505 |
| DCN1-like protein 5 || id:prot-a-774 | rs2668196 | T | A | 3 | 165502709 | -0.1886 | 4.90E-09 | 0.0322 | 0.81693 | -5.857142857 | 34.30612245 |
| DCN1-like protein 5 || id:prot-a-774 | rs74480769 | G | A | 5 | 40972211 | -0.44 | 2.00E-09 | 0.0733 | 0.0317 | -6.002728513 | 36.0327496 |
| DCN1-like protein 5 || id:prot-a-774 | rs967645 | T | C | 17 | 26713970 | 0.2536 | 7.94E-26 | 0.0241 | 0.50945 | 10.52282158 | 110.7297739 |
| Death-associated protein kinase 2 || id:prot-a-763 | rs62143198 | A | G | 19 | 54320939 | 0.6262 | 3.24E-105 | 0.0287 | 0.21453 | 21.81881533 | 476.0607024 |
| Dedicator of cytokinesis protein 9 || id:prot-a-854 | rs3197999 | A | G | 3 | 49721532 | -0.297 | 1.15E-28 | 0.0267 | 0.28831 | -11.12359551 | 123.734377 |
| Deformed epidermal autoregulatory factor 1 homolog || id:prot-a-780 | rs74480769 | G | A | 5 | 40972211 | -0.4805 | 5.50E-11 | 0.0733 | 0.0317 | -6.555252387 | 42.97133386 |
| Deformed epidermal autoregulatory factor 1 homolog || id:prot-a-780 | rs5167 | G | T | 19 | 45448465 | 0.1993 | 1.00E-14 | 0.0258 | 0.34696 | 7.724806202 | 59.67263085 |
| Delta-like protein 1 || id:prot-a-830 | rs959025 | T | C | 6 | 170588654 | 0.1911 | 1.35E-14 | 0.0248 | 0.41564 | 7.705645161 | 59.37696735 |
| Delta-like protein 1 || id:prot-a-831 | rs2344744 | G | T | 6 | 170588559 | 0.139 | 2.40E-08 | 0.0249 | 0.41462 | 5.582329317 | 31.16240061 |
| Delta-like protein 1 || id:prot-a-831 | rs141738059 | C | T | 9 | 116832007 | 0.6146 | 3.47E-08 | 0.1114 | 0.01278 | 5.517055655 | 30.4379031 |
| Delta-like protein 1 || id:prot-a-831 | rs7259081 | A | T | 19 | 54339243 | 0.1795 | 1.12E-08 | 0.0314 | 0.75126 | 5.71656051 | 32.67906406 |
| Deoxycytidine kinase || id:prot-a-767 | rs12038333 | A | G | 1 | 196672454 | -0.1517 | 2.14E-09 | 0.0253 | 0.6091 | -5.996047431 | 35.95258479 |
| Deoxycytidine kinase || id:prot-a-767 | rs429358 | C | T | 19 | 45411941 | 0.3128 | 7.41E-21 | 0.0334 | 0.15301 | 9.365269461 | 87.70827208 |
| Deoxynucleoside triphosphate triphosphohydrolase SAMHD1 || id:prot-a-2624 | rs7212510 | A | T | 17 | 26703682 | 0.1444 | 3.16E-09 | 0.0244 | 0.50911 | 5.918032787 | 35.02311207 |
| Dermatopontin || id:prot-a-861 | rs79145235 | A | G | 1 | 168930325 | -0.6499 | 4.47E-13 | 0.0898 | 0.01919 | -7.237193764 | 52.37697358 |
| Dermatopontin || id:prot-a-861 | rs1018454 | C | A | 1 | 168697761 | 0.4155 | 7.76E-67 | 0.0241 | 0.58588 | 17.2406639 | 297.2404917 |
| Desmocollin-2 || id:prot-a-866 | rs1789063 | A | T | 18 | 28673913 | -0.2159 | 1.82E-13 | 0.0293 | 0.76883 | -7.368600683 | 54.29627602 |
| Desmocollin-3 || id:prot-a-867 | rs7873022 | G | T | 9 | 14625979 | 0.1419 | 7.08E-09 | 0.0245 | 0.49034 | 5.791836735 | 33.54537276 |
| Desmocollin-3 || id:prot-a-867 | rs12446816 | G | A | 16 | 14092341 | 0.1426 | 2.75E-08 | 0.0257 | 0.37697 | 5.548638132 | 30.78738512 |
| Desmocollin-3 || id:prot-a-867 | rs7258432 | A | G | 19 | 54332041 | 0.1359 | 4.47E-08 | 0.0248 | 0.56463 | 5.47983871 | 30.02863228 |
| Desmoglein-1 || id:prot-a-869 | rs368465 | C | T | 1 | 196671981 | -0.1681 | 4.17E-11 | 0.0255 | 0.61344 | -6.592156863 | 43.4565321 |
| Desmoglein-1 || id:prot-a-869 | rs74480769 | G | A | 5 | 40972211 | -0.5771 | 2.88E-15 | 0.0731 | 0.0317 | -7.894664843 | 62.32573298 |
| Desmoglein-2 || id:prot-a-870 | rs687621 | G | A | 9 | 136137065 | 0.2221 | 3.63E-17 | 0.0264 | 0.31448 | 8.412878788 | 70.7765295 |
| Desmoglein-2 || id:prot-a-870 | rs2704050 | G | A | 18 | 29095888 | -0.1687 | 9.33E-12 | 0.0248 | 0.49311 | -6.802419355 | 46.27290908 |
| Di-N-acetylchitobiase || id:prot-a-708 | rs34636845 | G | A | 7 | 65493198 | -0.3902 | 1.23E-08 | 0.0685 | 0.03505 | -5.696350365 | 32.44840748 |
| Di-N-acetylchitobiase || id:prot-a-708 | rs1071803 | C | T | 14 | 106209119 | 0.5283 | 3.02E-94 | 0.0257 | 0.66685 | 20.55642023 | 422.5664128 |
| Diablo homolog, mitochondrial || id:prot-a-818 | rs1718860 | A | G | 4 | 57949517 | -0.1831 | 5.37E-11 | 0.0279 | 0.73338 | -6.562724014 | 43.06934649 |
| Diamine acetyltransferase 1 || id:prot-a-2628 | rs2688862 | T | C | 10 | 14771809 | -0.1445 | 1.66E-08 | 0.0256 | 0.6527 | -5.64453125 | 31.86073303 |
| Diamine acetyltransferase 2 || id:prot-a-2629 | rs3917503 | T | C | 7 | 94945453 | 0.15 | 1.23E-08 | 0.0263 | 0.33528 | 5.703422053 | 32.52902312 |
| Diamine acetyltransferase 2 || id:prot-a-2629 | rs11078700 | G | A | 17 | 7511936 | -0.3213 | 3.89E-11 | 0.0486 | 0.06986 | -6.611111111 | 43.70679012 |
| Diamine acetyltransferase 2 || id:prot-a-2629 | rs10418046 | G | T | 19 | 54327869 | 0.2006 | 1.66E-11 | 0.0298 | 0.21727 | 6.731543624 | 45.31367956 |
| Dickkopf-like protein 1 || id:prot-a-824 | rs704 | A | G | 17 | 26694861 | -0.1775 | 4.27E-13 | 0.0245 | 0.46665 | -7.244897959 | 52.48854644 |
| Dickkopf-related protein 1 || id:prot-a-821 | rs6993770 | T | A | 8 | 106581528 | -0.2607 | 7.76E-22 | 0.0272 | 0.28225 | -9.584558824 | 91.86376784 |
| Dickkopf-related protein 1 || id:prot-a-821 | rs1194673 | A | G | 10 | 54141652 | 0.1824 | 2.75E-12 | 0.0261 | 0.63246 | 6.988505747 | 48.83921258 |
| Dickkopf-related protein 1 || id:prot-a-821 | rs7080386 | A | C | 10 | 65048306 | 0.1666 | 3.89E-11 | 0.0252 | 0.41587 | 6.611111111 | 43.70679012 |
| Dickkopf-related protein 3 || id:prot-a-822 | rs11022114 | A | G | 11 | 12038874 | 0.3364 | 6.46E-36 | 0.0269 | 0.32795 | 12.50557621 | 156.3894363 |
| Dickkopf-related protein 4 || id:prot-a-823 | rs6993770 | T | A | 8 | 106581528 | -0.2553 | 5.75E-21 | 0.0272 | 0.28225 | -9.386029412 | 88.09754812 |
| Dickkopf-related protein 4 || id:prot-a-823 | rs7080386 | A | C | 10 | 65048306 | 0.1672 | 3.39E-11 | 0.0252 | 0.41587 | 6.634920635 | 44.02217183 |
| Dickkopf-related protein 4 || id:prot-a-823 | rs1194673 | A | G | 10 | 54141652 | 0.1723 | 4.27E-11 | 0.0261 | 0.63246 | 6.601532567 | 43.58023223 |
| Dickkopf-related protein 4 || id:prot-a-823 | rs7213215 | G | A | 17 | 64164559 | 0.433 | 6.61E-15 | 0.0556 | 0.05004 | 7.787769784 | 60.64935821 |
| Dihydrofolate reductase || id:prot-a-812 | rs676457 | T | A | 9 | 136146227 | 0.1851 | 2.45E-12 | 0.0264 | 0.31502 | 7.011363636 | 49.15922004 |
| Dihydrofolate reductase || id:prot-a-812 | rs216311 | C | T | 12 | 6128443 | -0.6169 | 2.57E-148 | 0.0238 | 0.61172 | -25.92016807 | 671.8551126 |
| Dihydropteridine reductase || id:prot-a-2464 | rs2581888 | G | A | 3 | 29813871 | -0.1423 | 4.79E-08 | 0.0261 | 0.3876 | -5.45210728 | 29.72547379 |
| Dihydropteridine reductase || id:prot-a-2464 | rs28719835 | T | C | 4 | 17520066 | -0.5668 | 1.05E-94 | 0.0275 | 0.24258 | -20.61090909 | 424.8095736 |
| Dihydropteridine reductase || id:prot-a-2464 | rs2254844 | T | A | 5 | 153877960 | -0.4244 | 2.95E-08 | 0.0765 | 0.9726 | -5.547712418 | 30.77711308 |
| Dihydropteridine reductase || id:prot-a-2464 | rs62143197 | A | G | 19 | 54320716 | 0.2437 | 6.76E-16 | 0.0302 | 0.22311 | 8.069536424 | 65.1174181 |
| Dipeptidase 2 || id:prot-a-857 | rs34959878 | CT | C | 3 | 52672618 | 0.1431 | 1.23E-08 | 0.0251 | 0.41161 | 5.701195219 | 32.50362693 |
| Dipeptidase 2 || id:prot-a-857 | rs2048 | T | G | 4 | 187148133 | -0.2536 | 2.51E-25 | 0.0244 | 0.51181 | -10.39344262 | 108.0236496 |
| Dipeptidase 2 || id:prot-a-857 | rs2731673 | C | T | 5 | 176839898 | -0.2235 | 1.35E-15 | 0.028 | 0.75237 | -7.982142857 | 63.71460459 |
| Dipeptidase 2 || id:prot-a-857 | rs2519093 | T | C | 9 | 136141870 | -0.2256 | 1.82E-12 | 0.032 | 0.17823 | -7.05 | 49.7025 |
| Dipeptidyl peptidase 2 || id:prot-a-859 | rs13402561 | G | C | 2 | 3639921 | -0.205 | 4.57E-10 | 0.0329 | 0.81414 | -6.23100304 | 38.82539888 |
| Dipeptidyl peptidase 2 || id:prot-a-859 | rs704 | A | G | 17 | 26694861 | 0.2701 | 7.94E-29 | 0.0242 | 0.46665 | 11.16115702 | 124.5714261 |
| Dipeptidyl peptidase 2 || id:prot-a-860 | rs10747049 | C | G | 9 | 140008750 | 0.3641 | 2.29E-39 | 0.0277 | 0.74955 | 13.14440433 | 172.7753652 |
| Discoidin domain-containing receptor 2 || id:prot-a-776 | rs71633870 | A | G | 1 | 199662145 | -0.7065 | 4.37E-08 | 0.129 | 0.01082 | -5.476744186 | 29.99472688 |
| Discoidin, CUB and LCCL domain-containing protein 2 || id:prot-a-766 | rs9864010 | A | G | 3 | 98678173 | -0.6199 | 6.61E-31 | 0.0536 | 0.05461 | -11.56529851 | 133.7561296 |
| Discoidin, CUB and LCCL domain-containing protein 2 || id:prot-a-766 | rs12487717 | A | G | 3 | 98605007 | -1.038 | 1.32E-23 | 0.1036 | 0.01477 | -10.01930502 | 100.3864731 |
| Discoidin, CUB and LCCL domain-containing protein 2 || id:prot-a-766 | rs28929474 | T | C | 14 | 94844947 | 0.5463 | 5.89E-11 | 0.0835 | 0.02269 | 6.54251497 | 42.80450213 |
| Disintegrin and metalloproteinase domain-containing protein 11 || id:prot-a-25 | rs13402475 | G | C | 2 | 3639909 | -0.3383 | 3.89E-26 | 0.032 | 0.80838 | -10.571875 | 111.764541 |
| Disintegrin and metalloproteinase domain-containing protein 11 || id:prot-a-25 | rs7412 | T | C | 19 | 45412079 | -0.3721 | 6.46E-16 | 0.0461 | 0.07776 | -8.071583514 | 65.15046043 |
| Disintegrin and metalloproteinase domain-containing protein 19 || id:prot-a-27 | rs13086583 | T | G | 3 | 22100726 | -0.296 | 4.37E-08 | 0.054 | 0.05813 | -5.481481481 | 30.04663923 |
| Disintegrin and metalloproteinase domain-containing protein 19 || id:prot-a-27 | rs71461575 | A | G | 15 | 25179632 | -0.4169 | 2.57E-08 | 0.0749 | 0.03622 | -5.566088117 | 30.98133693 |
| Disintegrin and metalloproteinase domain-containing protein 23 || id:prot-a-28 | rs1921673 | G | A | 2 | 207324282 | 0.4321 | 3.24E-64 | 0.0255 | 0.6846 | 16.94509804 | 287.1363476 |
| Disintegrin and metalloproteinase domain-containing protein 23 || id:prot-a-28 | rs191343078 | T | C | 2 | 206956586 | -0.3427 | 3.16E-08 | 0.0619 | 0.04768 | -5.53634895 | 30.6511597 |
| Disintegrin and metalloproteinase domain-containing protein 23 || id:prot-a-28 | rs74584500 | A | G | 2 | 207575670 | 0.4725 | 4.37E-22 | 0.0489 | 0.07071 | 9.662576687 | 93.36538823 |
| Disintegrin and metalloproteinase domain-containing protein 23 || id:prot-a-28 | rs114865519 | G | A | 2 | 207708916 | -0.4097 | 2.19E-10 | 0.0645 | 0.04248 | -6.351937984 | 40.34711616 |
| Disintegrin and metalloproteinase domain-containing protein 23 || id:prot-a-28 | rs11244049 | C | T | 9 | 136123092 | 0.2919 | 7.94E-09 | 0.0506 | 0.06645 | 5.768774704 | 33.27876158 |
| Disks large homolog 3 || id:prot-a-826 | rs4253244 | A | C | 4 | 187153775 | -0.1445 | 2.34E-08 | 0.0259 | 0.63535 | -5.579150579 | 31.12692118 |
| DNA-(apurinic or apyrimidinic site) lyase || id:prot-a-121 | rs62143206 | T | G | 19 | 54326212 | 0.6947 | 1.70E-140 | 0.0275 | 0.2131 | 25.26181818 | 638.1594579 |
| DNA-3-methyladenine glycosylase || id:prot-a-1928 | rs62143198 | A | G | 19 | 54320939 | 0.7348 | 2.63E-152 | 0.028 | 0.21453 | 26.24285714 | 688.687551 |
| DNA-binding protein inhibitor ID-1 || id:prot-a-1407 | rs28664709 | A | G | 1 | 196671217 | -0.1489 | 4.27E-09 | 0.0254 | 0.6109 | -5.862204724 | 34.36544423 |
| DNA-binding protein inhibitor ID-2 || id:prot-a-1408 | rs941758 | A | C | 4 | 74718941 | -0.1633 | 8.13E-10 | 0.0266 | 0.70817 | -6.139097744 | 37.68852111 |
| DNA-binding protein SATB1 || id:prot-a-2630 | rs6993770 | T | A | 8 | 106581528 | -0.1576 | 8.71E-09 | 0.0274 | 0.28225 | -5.751824818 | 33.08348873 |
| DNA-directed DNA/RNA polymerase mu || id:prot-a-2323 | rs62143197 | A | G | 19 | 54320716 | 0.2011 | 3.09E-11 | 0.0303 | 0.22311 | 6.636963696 | 44.04928711 |
| DNA-directed RNA polymerases I and III subunit RPAC1 || id:prot-a-2324 | rs368465 | C | T | 1 | 196671981 | -0.1591 | 4.37E-10 | 0.0255 | 0.61344 | -6.239215686 | 38.92781238 |
| DNA-directed RNA polymerases I and III subunit RPAC1 || id:prot-a-2324 | rs74480769 | G | A | 5 | 40972211 | -0.5412 | 1.38E-13 | 0.0732 | 0.0317 | -7.393442623 | 54.66299382 |
| DNA-directed RNA polymerases I and III subunit RPAC1 || id:prot-a-2324 | rs429358 | C | T | 19 | 45411941 | 0.2148 | 1.74E-10 | 0.0336 | 0.15301 | 6.392857143 | 40.86862245 |
| DNA dC->dU-editing enzyme APOBEC-3G || id:prot-a-128 | rs62143197 | A | G | 19 | 54320716 | 0.202 | 2.45E-11 | 0.0303 | 0.22311 | 6.666666667 | 44.44444444 |
| DNA repair protein RAD51 homolog 1 || id:prot-a-2485 | rs570618 | G | T | 1 | 196657064 | -0.1691 | 2.34E-11 | 0.0253 | 0.61149 | -6.683794466 | 44.67310847 |
| DNA repair protein RAD51 homolog 1 || id:prot-a-2485 | rs74480769 | G | A | 5 | 40972211 | -0.5109 | 3.02E-12 | 0.0732 | 0.0317 | -6.979508197 | 48.71353467 |
| DNA repair protein RAD51 homolog 4 || id:prot-a-2486 | rs6838228 | G | A | 4 | 69453239 | 0.273 | 2.29E-20 | 0.0295 | 0.64006 | 9.254237288 | 85.64090779 |
| DNA repair protein RAD51 homolog 4 || id:prot-a-2486 | rs45446698 | G | T | 7 | 99332948 | -1.1051 | 4.68E-74 | 0.0607 | 0.04093 | -18.20593081 | 331.4559166 |
| DNA repair protein RAD51 homolog 4 || id:prot-a-2486 | rs2547231 | A | C | 19 | 48385057 | 0.2028 | 5.25E-10 | 0.0326 | 0.82869 | 6.220858896 | 38.6990854 |
| DNA repair protein XRCC4 || id:prot-a-3242 | rs71631868 | C | T | 1 | 196815711 | -0.2566 | 3.24E-17 | 0.0304 | 0.20641 | -8.440789474 | 71.24692694 |
| DNA repair protein XRCC4 || id:prot-a-3242 | rs5167 | G | T | 19 | 45448465 | 0.26 | 2.75E-24 | 0.0256 | 0.34696 | 10.15625 | 103.1494141 |
| DNA/RNA-binding protein KIN17 || id:prot-a-1638 | rs62143198 | A | G | 19 | 54320939 | 0.2455 | 7.08E-16 | 0.0304 | 0.21453 | 8.075657895 | 65.21625043 |
| DNA/RNA-binding protein KIN17 || id:prot-a-1638 | rs73515693 | G | A | 19 | 14500957 | -0.1523 | 2.34E-08 | 0.0273 | 0.39938 | -5.578754579 | 31.12250265 |
| DNA/RNA-binding protein KIN17 || id:prot-a-1639 | rs3917545 | C | A | 7 | 94936235 | 0.2086 | 2.04E-08 | 0.0372 | 0.13088 | 5.607526882 | 31.44435773 |
| DNA/RNA-binding protein KIN17 || id:prot-a-1639 | rs2519093 | T | C | 9 | 136141870 | -0.3175 | 1.62E-23 | 0.0318 | 0.17823 | -9.98427673 | 99.68578181 |
| DNA/RNA-binding protein KIN17 || id:prot-a-1639 | rs7412 | T | C | 19 | 45412079 | 0.469 | 1.20E-24 | 0.0458 | 0.07776 | 10.24017467 | 104.8611773 |
| DnaJ homolog subfamily A member 4 || id:prot-a-835 | rs11639191 | A | C | 15 | 78547449 | 0.1617 | 2.34E-08 | 0.0289 | 0.23692 | 5.595155709 | 31.30576741 |
| DnaJ homolog subfamily B member 11 || id:prot-a-836 | rs9841006 | T | C | 3 | 186293135 | -0.2769 | 5.25E-10 | 0.0446 | 0.08486 | -6.208520179 | 38.54572282 |
| DnaJ homolog subfamily B member 11 || id:prot-a-836 | rs116795853 | A | G | 6 | 31373985 | 0.6636 | 1.07E-18 | 0.0752 | 0.02637 | 8.824468085 | 77.87123699 |
| DnaJ homolog subfamily B member 11 || id:prot-a-836 | rs6993770 | T | A | 8 | 106581528 | -0.1849 | 1.35E-11 | 0.0273 | 0.28225 | -6.772893773 | 45.87209006 |
| DnaJ homolog subfamily B member 14 || id:prot-a-840 | rs4632248 | T | G | 19 | 54324995 | 0.6563 | 4.90E-123 | 0.0278 | 0.21439 | 23.60791367 | 557.3335878 |
| DnaJ homolog subfamily B member 2 || id:prot-a-841 | rs62143197 | A | G | 19 | 54320716 | 0.5014 | 4.37E-66 | 0.0292 | 0.22311 | 17.17123288 | 294.8512385 |
| DnaJ homolog subfamily B member 9 || id:prot-a-842 | rs72666463 | A | G | 4 | 109693855 | 0.4044 | 3.72E-08 | 0.0735 | 0.02859 | 5.502040816 | 30.27245314 |
| DnaJ homolog subfamily B member 9 || id:prot-a-842 | rs28929474 | T | C | 14 | 94844947 | 0.7288 | 1.70E-18 | 0.083 | 0.02269 | 8.780722892 | 77.1010945 |
| DnaJ homolog subfamily B member 9 || id:prot-a-842 | rs704 | A | G | 17 | 26694861 | 0.3281 | 1.86E-42 | 0.024 | 0.46665 | 13.67083333 | 186.891684 |
| DnaJ homolog subfamily B member 9 || id:prot-a-842 | rs1801689 | C | A | 17 | 64210580 | 0.6578 | 1.35E-20 | 0.0707 | 0.03043 | 9.304101839 | 86.56631103 |
| DnaJ homolog subfamily C member 10 || id:prot-a-843 | rs9265495 | G | A | 6 | 31297473 | -0.2037 | 3.63E-09 | 0.0345 | 0.82103 | -5.904347826 | 34.86132325 |
| DnaJ homolog subfamily C member 15 || id:prot-a-844 | rs9526310 | C | T | 13 | 47891517 | 0.4645 | 9.12E-09 | 0.0808 | 0.02409 | 5.748762376 | 33.04826886 |
| DnaJ homolog subfamily C member 15 || id:prot-a-844 | rs9616018 | A | G | 22 | 46885999 | 0.2521 | 2.82E-08 | 0.0454 | 0.08018 | 5.552863436 | 30.83429234 |
| DnaJ homolog subfamily C member 17 || id:prot-a-845 | rs62143197 | A | G | 19 | 54320716 | 0.3738 | 3.72E-36 | 0.0298 | 0.22311 | 12.54362416 | 157.3425071 |
| DnaJ homolog subfamily C member 30 || id:prot-a-849 | rs73702564 | T | C | 7 | 73084816 | 0.7183 | 6.92E-31 | 0.0622 | 0.0386 | 11.54823151 | 133.361651 |
| Docking protein 2 || id:prot-a-855 | rs62143197 | A | G | 19 | 54320716 | 0.3332 | 8.32E-29 | 0.0299 | 0.22311 | 11.14381271 | 124.1845617 |
| Dolichyl-diphosphooligosaccharide--protein glycosyltransferase subunit 1 || id:prot-a-2588 | rs2712417 | G | A | 3 | 128345179 | -0.3447 | 3.09E-44 | 0.0247 | 0.61802 | -13.95546559 | 194.7550198 |
| Dolichyl-diphosphooligosaccharide--protein glycosyltransferase subunit 1 || id:prot-a-2589 | rs4802831 | T | C | 19 | 52127134 | -0.2416 | 1.86E-12 | 0.0343 | 0.84802 | -7.043731778 | 49.61415737 |
| Dolichyl-diphosphooligosaccharide--protein glycosyltransferase subunit 1 || id:prot-a-2589 | rs1106476 | A | T | 19 | 52130637 | -1.1698 | 1.00E-200 | 0.0319 | 0.11691 | -36.67084639 | 1344.750975 |
| Dorsal root ganglia homeobox protein || id:prot-a-865 | rs663887 | A | G | 1 | 170626953 | 0.7239 | 4.79E-191 | 0.0246 | 0.29329 | 29.42682927 | 865.9382808 |
| Dorsal root ganglia homeobox protein || id:prot-a-865 | rs4987333 | G | A | 1 | 169669570 | 0.5571 | 4.27E-08 | 0.1017 | 0.01487 | 5.477876106 | 30.00712663 |
| Double-stranded RNA-binding protein Staufen homolog 1 || id:prot-a-2871 | rs62143194 | G | C | 19 | 54319624 | 0.4092 | 1.35E-42 | 0.0299 | 0.22364 | 13.68561873 | 187.29616 |
| Double-stranded RNA-binding protein Staufen homolog 2 || id:prot-a-2872 | rs62143198 | A | G | 19 | 54320939 | 0.4861 | 8.13E-61 | 0.0295 | 0.21453 | 16.4779661 | 271.5233668 |
| Down syndrome cell adhesion molecule || id:prot-a-868 | rs78111814 | C | T | 21 | 42060498 | 0.4056 | 1.91E-10 | 0.0637 | 0.04521 | 6.367346939 | 40.54310704 |
| Drebrin-like protein || id:prot-a-764 | rs4241818 | C | T | 4 | 187153786 | 0.2476 | 3.89E-24 | 0.0244 | 0.51359 | 10.14754098 | 102.972588 |
| Drebrin-like protein || id:prot-a-764 | rs75077631 | G | GC | 5 | 176840084 | -0.202 | 4.17E-13 | 0.0279 | 0.25227 | -7.240143369 | 52.41967601 |
| Drebrin-like protein || id:prot-a-764 | rs10424405 | G | A | 19 | 54321933 | 0.2581 | 8.13E-18 | 0.03 | 0.2203 | 8.603333333 | 74.01734444 |
| Dual specificity mitogen-activated protein kinase kinase 1 || id:prot-a-1840 | rs62143197 | A | G | 19 | 54320716 | 0.5531 | 1.55E-81 | 0.0289 | 0.22311 | 19.1384083 | 366.2786724 |
| Dual specificity mitogen-activated protein kinase kinase 3 || id:prot-a-1841 | rs11080055 | C | A | 17 | 26649724 | 0.1565 | 1.15E-10 | 0.0243 | 0.50786 | 6.440329218 | 41.47784044 |
| Dual specificity mitogen-activated protein kinase kinase 3 || id:prot-a-1841 | rs62143197 | A | G | 19 | 54320716 | 0.3027 | 6.46E-24 | 0.03 | 0.22311 | 10.09 | 101.8081 |
| Dual specificity mitogen-activated protein kinase kinase 4 || id:prot-a-1842 | rs17315646 | G | C | 1 | 230295307 | -0.2668 | 8.32E-27 | 0.0249 | 0.59234 | -10.71485944 | 114.8082128 |
| Dual specificity mitogen-activated protein kinase kinase 4 || id:prot-a-1842 | rs187815118 | T | C | 3 | 186410219 | -0.6639 | 1.86E-08 | 0.118 | 0.01318 | -5.626271186 | 31.65492746 |
| Dual specificity mitogen-activated protein kinase kinase 4 || id:prot-a-1842 | rs2228243 | G | A | 3 | 186395113 | 0.8661 | 1.00E-200 | 0.0263 | 0.20387 | 32.93155894 | 1084.487574 |
| Dual specificity protein kinase CLK2 || id:prot-a-586 | rs570618 | G | T | 1 | 196657064 | -0.1789 | 1.51E-12 | 0.0253 | 0.61149 | -7.071146245 | 50.00110922 |
| Dual specificity protein kinase CLK2 || id:prot-a-586 | rs74480769 | G | A | 5 | 40972211 | -0.404 | 3.72E-08 | 0.0734 | 0.0317 | -5.504087193 | 30.29497583 |
| Dual specificity protein kinase CLK2 || id:prot-a-586 | rs704 | A | G | 17 | 26694861 | 0.4086 | 7.08E-67 | 0.0237 | 0.46665 | 17.24050633 | 297.2350585 |
| Dual specificity protein phosphatase 13 isoform A || id:prot-a-873 | rs113194078 | G | C | 6 | 158061683 | -0.3978 | 1.58E-08 | 0.0704 | 0.03295 | -5.650568182 | 31.92892078 |
| Dual specificity protein phosphatase 13 isoform A || id:prot-a-873 | rs705379 | A | G | 7 | 94953895 | -0.2807 | 1.20E-29 | 0.0248 | 0.48664 | -11.31854839 | 128.1095376 |
| Dual specificity protein phosphatase 13 isoform A || id:prot-a-873 | rs6480771 | C | T | 10 | 76861680 | -0.2994 | 8.32E-34 | 0.0247 | 0.41514 | -12.12145749 | 146.9297317 |
| Dual specificity protein phosphatase 16 || id:prot-a-875 | rs113066561 | C | T | 3 | 67908752 | -0.4454 | 3.80E-09 | 0.0756 | 0.02851 | -5.891534392 | 34.71017749 |
| Dual specificity protein phosphatase 4 || id:prot-a-876 | rs2027993 | T | G | 17 | 26706946 | 0.1738 | 1.17E-12 | 0.0244 | 0.51371 | 7.12295082 | 50.73642838 |
| Dual specificity tyrosine-phosphorylation-regulated kinase 3 || id:prot-a-882 | rs2227736 | C | G | 17 | 26693231 | -0.1442 | 6.76E-09 | 0.0249 | 0.59312 | -5.791164659 | 33.5375881 |
| Dynactin-associated protein || id:prot-a-879 | rs12434433 | T | C | 14 | 106203840 | 0.2144 | 1.48E-14 | 0.0279 | 0.28247 | 7.684587814 | 59.05288987 |
| Dynactin-associated protein || id:prot-a-879 | rs967645 | T | C | 17 | 26713970 | -0.1649 | 1.32E-11 | 0.0244 | 0.50945 | -6.758196721 | 45.67322292 |
| Dynactin subunit 2 || id:prot-a-771 | rs1126605 | T | C | 12 | 7242204 | 0.2687 | 1.48E-09 | 0.0444 | 0.08581 | 6.051801802 | 36.62430505 |
| Dynein light chain 1, cytoplasmic || id:prot-a-880 | rs4511431 | T | C | 14 | 106523408 | 0.1868 | 2.63E-13 | 0.0255 | 0.38123 | 7.325490196 | 53.66280661 |
| Dystroglycan || id:prot-a-762 | rs145200155 | C | T | 10 | 129204799 | 0.4389 | 1.17E-08 | 0.077 | 0.0285 | 5.7 | 32.49 |
| E-selectin || id:prot-a-2665 | rs2519093 | T | C | 9 | 136141870 | -1.1673 | 1.00E-200 | 0.025 | 0.17823 | -46.692 | 2180.142864 |
| E-selectin || id:prot-a-2665 | rs12288924 | A | G | 11 | 126286010 | -0.1788 | 1.32E-08 | 0.0315 | 0.20039 | -5.676190476 | 32.21913832 |
| E3 ISG15--protein ligase HERC5 || id:prot-a-1331 | rs74480769 | G | A | 5 | 40972211 | -0.4207 | 1.00E-08 | 0.0734 | 0.0317 | -5.731607629 | 32.85132602 |
| E3 ubiquitin-protein ligase CBL || id:prot-a-370 | rs13276307 | T | C | 8 | 144646352 | 0.165 | 7.41E-09 | 0.0285 | 0.25219 | 5.789473684 | 33.51800554 |
| E3 ubiquitin-protein ligase CBL || id:prot-a-370 | rs62143198 | A | G | 19 | 54320939 | 0.6113 | 9.55E-100 | 0.0288 | 0.21453 | 21.22569444 | 450.5301046 |
| E3 ubiquitin-protein ligase DTX3L || id:prot-a-872 | rs2367277 | G | A | 3 | 192758964 | -0.1383 | 2.14E-08 | 0.0247 | 0.56366 | -5.599190283 | 31.35093183 |
| E3 ubiquitin-protein ligase DTX3L || id:prot-a-872 | rs62143197 | A | G | 19 | 54320716 | 0.3617 | 7.24E-34 | 0.0298 | 0.22311 | 12.13758389 | 147.3209428 |
| E3 ubiquitin-protein ligase Mdm2 || id:prot-a-1873 | rs141170965 | G | T | 2 | 108187572 | -0.7279 | 4.47E-08 | 0.133 | 0.0111 | -5.472932331 | 29.9529883 |
| E3 ubiquitin-protein ligase NEURL1 || id:prot-a-2037 | rs2299255 | C | T | 7 | 94938786 | 0.2718 | 2.29E-13 | 0.0371 | 0.1295 | 7.326145553 | 53.67240866 |
| E3 ubiquitin-protein ligase parkin || id:prot-a-2179 | rs117130630 | A | G | 14 | 89385559 | 0.6221 | 5.01E-09 | 0.1064 | 0.01493 | 5.846804511 | 34.18512299 |
| E3 ubiquitin-protein ligase pellino homolog 2 || id:prot-a-2246 | rs1007398 | G | A | 17 | 26665648 | 0.1676 | 1.55E-11 | 0.0249 | 0.59349 | 6.730923695 | 45.30533378 |
| E3 ubiquitin-protein ligase RBBP6 || id:prot-a-2498 | rs74480769 | G | A | 5 | 40972211 | -0.438 | 2.34E-09 | 0.0734 | 0.0317 | -5.967302452 | 35.60869856 |
| E3 ubiquitin-protein ligase RNF128 || id:prot-a-2562 | rs33944729 | T | TA | 1 | 196652124 | 0.1477 | 5.62E-09 | 0.0253 | 0.38701 | 5.837944664 | 34.0815979 |
| E3 ubiquitin-protein ligase RNF128 || id:prot-a-2562 | rs704 | A | G | 17 | 26694861 | 0.246 | 4.68E-24 | 0.0243 | 0.46665 | 10.12345679 | 102.4843774 |
| E3 ubiquitin-protein ligase RNF13 || id:prot-a-2563 | rs59123177 | A | G | 3 | 186391274 | 0.2623 | 5.37E-25 | 0.0254 | 0.35103 | 10.32677165 | 106.6422128 |
| E3 ubiquitin-protein ligase RNF13 || id:prot-a-2563 | rs704 | A | G | 17 | 26694861 | 0.3745 | 1.05E-55 | 0.0238 | 0.46665 | 15.73529412 | 247.599481 |
| E3 ubiquitin-protein ligase RNF149 || id:prot-a-2566 | rs55852005 | A | G | 1 | 5157880 | 0.5954 | 2.57E-08 | 0.1069 | 0.0152 | 5.5696913 | 31.02146118 |
| E3 ubiquitin-protein ligase RNF34 || id:prot-a-2571 | rs546339 | C | G | 22 | 27628151 | 0.1419 | 2.51E-08 | 0.0255 | 0.5899 | 5.564705882 | 30.96595156 |
| E3 ubiquitin-protein ligase RNF43 || id:prot-a-2572 | rs9913833 | T | C | 17 | 26718592 | 0.1468 | 1.82E-09 | 0.0244 | 0.50976 | 6.016393443 | 36.19699006 |
| E3 ubiquitin-protein ligase RNF8 || id:prot-a-2573 | rs466344 | T | C | 1 | 196726613 | -0.1568 | 2.14E-09 | 0.0262 | 0.36173 | -5.984732824 | 35.81702698 |
| E3 ubiquitin-protein ligase RNF8 || id:prot-a-2573 | rs74480769 | G | A | 5 | 40972211 | -0.4145 | 1.62E-08 | 0.0734 | 0.0317 | -5.647138965 | 31.89017849 |
| E3 ubiquitin-protein ligase RNF8 || id:prot-a-2573 | rs3917529 | G | A | 7 | 94940235 | 0.1956 | 9.12E-13 | 0.0274 | 0.28284 | 7.138686131 | 50.96083968 |
| E3 ubiquitin-protein ligase RNF8 || id:prot-a-2573 | rs704 | A | G | 17 | 26694861 | 0.3156 | 2.82E-39 | 0.0241 | 0.46665 | 13.09543568 | 171.4904358 |
| Early endosome antigen 1 || id:prot-a-893 | rs74480769 | G | A | 5 | 40972211 | -0.4674 | 1.82E-10 | 0.0733 | 0.0317 | -6.376534789 | 40.66019591 |
| Ecto-ADP-ribosyltransferase 3 || id:prot-a-174 | rs4859610 | G | A | 4 | 77000441 | -0.3513 | 7.24E-35 | 0.0285 | 0.77429 | -12.32631579 | 151.9380609 |
| Ecto-ADP-ribosyltransferase 3 || id:prot-a-174 | rs56184627 | A | G | 16 | 210827 | -0.1459 | 1.17E-08 | 0.0256 | 0.41827 | -5.69921875 | 32.48109436 |
| Ecto-ADP-ribosyltransferase 3 || id:prot-a-175 | rs4859610 | G | A | 4 | 77000441 | -0.3378 | 2.95E-32 | 0.0286 | 0.77429 | -11.81118881 | 139.5041811 |
| Ecto-ADP-ribosyltransferase 3 || id:prot-a-175 | rs11646097 | C | T | 16 | 425605 | -0.1524 | 9.12E-10 | 0.0249 | 0.58983 | -6.120481928 | 37.46029903 |
| Ecto-ADP-ribosyltransferase 4 || id:prot-a-176 | rs247340 | T | G | 12 | 14858753 | -0.173 | 3.55E-08 | 0.0314 | 0.19146 | -5.50955414 | 30.35518682 |
| Ecto-ADP-ribosyltransferase 4 || id:prot-a-176 | rs1001096 | A | G | 12 | 14988455 | 0.74 | 1.00E-200 | 0.0217 | 0.39736 | 34.10138249 | 1162.904288 |
| Ectodysplasin-A, secreted form || id:prot-a-885 | rs7539005 | T | A | 1 | 196667252 | -0.1481 | 6.17E-09 | 0.0255 | 0.61028 | -5.807843137 | 33.73104191 |
| Ectodysplasin-A, secreted form || id:prot-a-885 | rs9272309 | G | A | 6 | 32603936 | 0.1409 | 3.47E-08 | 0.0256 | 0.37252 | 5.50390625 | 30.29298401 |
| Ectonucleoside triphosphate diphosphohydrolase 1 || id:prot-a-950 | rs74480769 | G | A | 5 | 40972211 | -0.566 | 9.77E-15 | 0.0731 | 0.0317 | -7.742818057 | 59.95123147 |
| Ectonucleoside triphosphate diphosphohydrolase 1 || id:prot-a-951 | rs11188501 | A | G | 10 | 97600919 | 0.2147 | 2.24E-16 | 0.0262 | 0.33986 | 8.194656489 | 67.15239497 |
| Ectonucleoside triphosphate diphosphohydrolase 3 || id:prot-a-952 | rs368465 | C | T | 1 | 196671981 | -0.1562 | 8.91E-10 | 0.0255 | 0.61344 | -6.125490196 | 37.52163014 |
| Ectonucleoside triphosphate diphosphohydrolase 3 || id:prot-a-952 | rs74480769 | G | A | 5 | 40972211 | -0.486 | 3.24E-11 | 0.0733 | 0.0317 | -6.630286494 | 43.96069899 |
| Ectonucleoside triphosphate diphosphohydrolase 5 || id:prot-a-953 | rs10864411 | T | C | 1 | 9425540 | 0.1489 | 4.79E-09 | 0.0254 | 0.38687 | 5.862204724 | 34.36544423 |
| Ectonucleoside triphosphate diphosphohydrolase 5 || id:prot-a-953 | rs149062332 | C | T | 3 | 186341025 | 0.6366 | 4.07E-08 | 0.116 | 0.01216 | 5.487931034 | 30.11738704 |
| Ectonucleoside triphosphate diphosphohydrolase 5 || id:prot-a-953 | rs57731447 | A | G | 14 | 74487521 | -0.9668 | 7.76E-87 | 0.0489 | 0.05877 | -19.77096115 | 390.8909046 |
| Ectonucleoside triphosphate diphosphohydrolase 5 || id:prot-a-953 | rs28929474 | T | C | 14 | 94844947 | 0.5169 | 6.03E-10 | 0.0835 | 0.02269 | 6.190419162 | 38.3212894 |
| Ectonucleoside triphosphate diphosphohydrolase 6 || id:prot-a-954 | rs10173355 | T | A | 2 | 234597321 | 0.1739 | 3.31E-11 | 0.0262 | 0.31721 | 6.63740458 | 44.05513956 |
| Ectonucleotide pyrophosphatase/phosphodiesterase family member 5 || id:prot-a-947 | rs1047153 | T | C | 6 | 46128745 | -0.7891 | 1.00E-200 | 0.0214 | 0.6464 | -36.87383178 | 1359.67947 |
| Ectonucleotide pyrophosphatase/phosphodiesterase family member 5 || id:prot-a-947 | rs727133 | C | G | 6 | 46156858 | -0.1835 | 6.76E-11 | 0.0281 | 0.26072 | -6.53024911 | 42.64415344 |
| Ectonucleotide pyrophosphatase/phosphodiesterase family member 7 || id:prot-a-948 | rs111596026 | A | G | 17 | 77631598 | 0.2792 | 9.77E-12 | 0.041 | 0.10158 | 6.809756098 | 46.37277811 |
| Ectonucleotide pyrophosphatase/phosphodiesterase family member 7 || id:prot-a-948 | rs11871061 | C | T | 17 | 77706544 | 0.9867 | 1.00E-200 | 0.02 | 0.35938 | 49.335 | 2433.942225 |
| EH domain-binding protein 1 || id:prot-a-911 | rs61808983 | T | C | 1 | 169474397 | -0.4784 | 7.08E-18 | 0.0555 | 0.05938 | -8.61981982 | 74.30129373 |
| Elafin || id:prot-a-2265 | rs16989763 | C | T | 20 | 43779963 | 0.3756 | 1.62E-35 | 0.0302 | 0.19505 | 12.43708609 | 154.6811105 |
| ELAV-like protein 1 || id:prot-a-928 | rs62143198 | A | G | 19 | 54320939 | 0.5035 | 1.74E-65 | 0.0295 | 0.21453 | 17.06779661 | 291.3096811 |
| EMI domain-containing protein 1 || id:prot-a-937 | rs831124 | A | T | 5 | 67574750 | -0.1559 | 5.89E-09 | 0.0268 | 0.65959 | -5.817164179 | 33.83939909 |
| EMI domain-containing protein 1 || id:prot-a-937 | rs74480769 | G | A | 5 | 40972211 | -0.448 | 1.00E-09 | 0.0733 | 0.0317 | -6.111869031 | 37.35494306 |
| EMILIN-3 || id:prot-a-938 | rs61739314 | C | G | 20 | 39990377 | -0.6855 | 3.63E-23 | 0.0691 | 0.03329 | -9.92040521 | 98.41443953 |
| EMILIN-3 || id:prot-a-939 | rs4527404 | C | G | 3 | 22045738 | 0.151 | 3.72E-09 | 0.0256 | 0.40559 | 5.8984375 | 34.79156494 |
| EMILIN-3 || id:prot-a-939 | rs80175408 | A | G | 6 | 162157905 | 0.3627 | 1.45E-08 | 0.064 | 0.0413 | 5.6671875 | 32.11701416 |
| EMILIN-3 || id:prot-a-940 | rs704 | A | G | 17 | 26694861 | -0.1641 | 2.24E-11 | 0.0245 | 0.46665 | -6.697959184 | 44.86265723 |
| Ena/VASP-like protein || id:prot-a-998 | rs10418046 | G | T | 19 | 54327869 | 0.3225 | 6.61E-28 | 0.0294 | 0.21727 | 10.96938776 | 120.3274677 |
| Endogenous retrovirus group V member 1 Env polyprotein || id:prot-a-986 | rs570618 | G | T | 1 | 196657064 | -0.1971 | 5.89E-15 | 0.0252 | 0.61149 | -7.821428571 | 61.1747449 |
| Endoglin || id:prot-a-943 | rs635634 | T | C | 9 | 136155000 | -0.2376 | 1.26E-13 | 0.0321 | 0.17981 | -7.401869159 | 54.78766705 |
| Endoplasmic reticulum aminopeptidase 1 || id:prot-a-971 | rs78740770 | C | T | 5 | 95943979 | 0.4049 | 9.12E-11 | 0.0625 | 0.03991 | 6.4784 | 41.96966656 |
| Endoplasmic reticulum aminopeptidase 1 || id:prot-a-971 | rs17482078 | T | C | 5 | 96118866 | -0.9615 | 1.00E-200 | 0.0251 | 0.21632 | -38.30677291 | 1467.408851 |
| Endoplasmic reticulum aminopeptidase 1 || id:prot-a-971 | rs145753269 | T | C | 5 | 95874744 | 0.705 | 2.57E-08 | 0.1266 | 0.01103 | 5.568720379 | 31.01064666 |
| Endoplasmic reticulum aminopeptidase 1 || id:prot-a-971 | rs1021717 | T | G | 5 | 96039895 | 1.069 | 3.16E-26 | 0.1009 | 0.01518 | 10.59464817 | 112.2465698 |
| Endoplasmic reticulum aminopeptidase 2 || id:prot-a-972 | rs2927608 | A | G | 5 | 96252432 | 1.0523 | 1.00E-200 | 0.0168 | 0.43729 | 62.63690476 | 3923.381838 |
| Endoplasmic reticulum aminopeptidase 2 || id:prot-a-972 | rs17399025 | G | A | 5 | 96013061 | -0.3041 | 1.26E-08 | 0.0534 | 0.06343 | -5.694756554 | 32.43025221 |
| Endoplasmic reticulum aminopeptidase 2 || id:prot-a-972 | rs114668143 | C | T | 5 | 96420331 | -0.9308 | 1.66E-18 | 0.1061 | 0.01393 | -8.772855796 | 76.96299882 |
| Endoplasmic reticulum lectin 1 || id:prot-a-979 | rs58359565 | A | C | 2 | 53958919 | -0.2076 | 2.09E-12 | 0.0295 | 0.21874 | -7.037288136 | 49.5234243 |
| Endoplasmic reticulum mannosyl-oligosaccharide 1,2-alpha-mannosidase || id:prot-a-1834 | rs528298 | T | A | 1 | 196660995 | 0.3669 | 6.31E-50 | 0.0247 | 0.61415 | 14.85425101 | 220.6487731 |
| Endoplasmic reticulum mannosyl-oligosaccharide 1,2-alpha-mannosidase || id:prot-a-1834 | rs75572374 | A | G | 17 | 64855512 | -0.1978 | 4.90E-08 | 0.0363 | 0.13066 | -5.449035813 | 29.69199129 |
| Endoplasmic reticulum resident protein 44 || id:prot-a-985 | rs6993770 | T | A | 8 | 106581528 | -0.246 | 1.48E-19 | 0.0272 | 0.28225 | -9.044117647 | 81.79606401 |
| Endoplasmin || id:prot-a-1386 | rs151057990 | G | GTATTATTCAAGTAAAC | 12 | 104460212 | 0.4006 | 8.91E-19 | 0.0453 | 0.08157 | 8.843267108 | 78.20337315 |
| Endoplasmin || id:prot-a-1386 | rs1165693 | A | G | 12 | 104340204 | 1.0974 | 1.00E-200 | 0.0182 | 0.32137 | 60.2967033 | 3635.692428 |
| Endostatin || id:prot-a-617 | rs12482088 | C | A | 21 | 46901973 | -0.2552 | 1.38E-19 | 0.0282 | 0.25455 | -9.04964539 | 81.89608169 |
| Endothelial cell-selective adhesion molecule || id:prot-a-987 | rs8176751 | T | C | 9 | 136131022 | 0.2739 | 2.24E-10 | 0.0432 | 0.09368 | 6.340277778 | 40.1991223 |
| Endothelial cell-selective adhesion molecule || id:prot-a-987 | rs11219769 | T | G | 11 | 124620147 | -0.2351 | 3.63E-17 | 0.0279 | 0.26406 | -8.426523297 | 71.00629488 |
| Endothelial cell-selective adhesion molecule || id:prot-a-987 | rs4632248 | T | G | 19 | 54324995 | -0.1669 | 2.51E-08 | 0.0299 | 0.21439 | -5.581939799 | 31.15805192 |
| Endothelial cell-selective adhesion molecule || id:prot-a-988 | rs77848689 | G | A | 8 | 23912194 | -0.6755 | 2.82E-08 | 0.1217 | 0.01114 | -5.5505341 | 30.8084288 |
| Endothelial cell-selective adhesion molecule || id:prot-a-988 | rs8176733 | C | T | 9 | 136132168 | 0.2615 | 1.15E-09 | 0.0429 | 0.09278 | 6.095571096 | 37.15598698 |
| Endothelial cell-selective adhesion molecule || id:prot-a-988 | rs11219769 | T | G | 11 | 124620147 | -0.2011 | 6.61E-13 | 0.028 | 0.26406 | -7.182142857 | 51.58317602 |
| Endothelial cell-specific molecule 1 || id:prot-a-990 | rs3815925 | T | C | 5 | 54253765 | 0.1568 | 3.63E-10 | 0.025 | 0.57499 | 6.272 | 39.337984 |
| Endothelial cell-specific molecule 1 || id:prot-a-990 | rs144217210 | C | G | 16 | 60205471 | 0.4185 | 4.17E-08 | 0.0763 | 0.02932 | 5.484927916 | 30.08443425 |
| Endothelial differentiation-related factor 1 || id:prot-a-891 | rs62143206 | T | G | 19 | 54326212 | 0.6862 | 1.74E-136 | 0.0276 | 0.2131 | 24.86231884 | 618.1348981 |
| Endothelial monocyte-activating polypeptide 2 || id:prot-a-65 | rs5167 | G | T | 19 | 45448465 | 0.3319 | 2.88E-39 | 0.0253 | 0.34696 | 13.11857708 | 172.0970645 |
| Endothelin-2 || id:prot-a-892 | rs4241819 | T | C | 4 | 187157140 | -0.1749 | 1.41E-12 | 0.0247 | 0.50721 | -7.08097166 | 50.14015965 |
| Endothelin-2 || id:prot-a-892 | rs1801020 | G | A | 5 | 176836532 | -0.1808 | 1.70E-10 | 0.0283 | 0.75032 | -6.38869258 | 40.81539288 |
| Endothelin-2 || id:prot-a-892 | rs704 | A | G | 17 | 26694861 | -0.3341 | 5.01E-44 | 0.024 | 0.46665 | -13.92083333 | 193.7896007 |
| Endothelin-converting enzyme 1 || id:prot-a-883 | rs4683701 | C | T | 3 | 142605146 | 0.3197 | 2.09E-38 | 0.0247 | 0.36802 | 12.94331984 | 167.5295284 |
| Endothelin-converting enzyme 1 || id:prot-a-883 | rs967645 | T | C | 17 | 26713970 | -0.1794 | 1.66E-13 | 0.0243 | 0.50945 | -7.382716049 | 54.50449627 |
| Endothelin-converting enzyme 1 || id:prot-a-884 | rs33944729 | T | TA | 1 | 196652124 | 0.1564 | 6.61E-10 | 0.0253 | 0.38701 | 6.181818182 | 38.21487603 |
| Endothelin-converting enzyme 1 || id:prot-a-884 | rs74480769 | G | A | 5 | 40972211 | -0.4908 | 2.09E-11 | 0.0733 | 0.0317 | -6.695770805 | 44.83334667 |
| Endothelin-converting enzyme 1 || id:prot-a-884 | rs4795433 | T | C | 17 | 26716821 | 0.2116 | 3.02E-18 | 0.0243 | 0.50963 | 8.70781893 | 75.82611052 |
| Enhancer of rudimentary homolog || id:prot-a-978 | rs62143206 | T | G | 19 | 54326212 | 0.4759 | 6.92E-61 | 0.0289 | 0.2131 | 16.46712803 | 271.1663055 |
| Enteropeptidase || id:prot-a-3025 | rs4632248 | T | G | 19 | 54324995 | -0.1926 | 1.17E-10 | 0.0299 | 0.21439 | -6.441471572 | 41.49255601 |
| Envoplakin || id:prot-a-999 | rs2013780 | G | A | 21 | 37378434 | -0.1556 | 8.91E-09 | 0.0271 | 0.72425 | -5.741697417 | 32.96708923 |
| Eotaxin || id:prot-a-387 | rs12075 | A | G | 1 | 159175354 | 0.2291 | 8.51E-20 | 0.0252 | 0.5838 | 9.091269841 | 82.65118733 |
| Eotaxin || id:prot-a-387 | rs2228467 | C | T | 3 | 42906116 | 0.5552 | 4.47E-30 | 0.0487 | 0.06675 | 11.40041068 | 129.9693636 |
| Eotaxin || id:prot-a-387 | rs757973 | G | A | 7 | 75495667 | -0.2462 | 1.66E-08 | 0.0436 | 0.90797 | -5.646788991 | 31.88622591 |
| EP300-interacting inhibitor of differentiation 3 || id:prot-a-915 | rs77122638 | C | T | 4 | 101362463 | -0.3983 | 1.58E-09 | 0.066 | 0.03684 | -6.034848485 | 36.41939624 |
| EP300-interacting inhibitor of differentiation 3 || id:prot-a-915 | rs10415457 | G | A | 19 | 12771220 | -0.1605 | 1.62E-10 | 0.0251 | 0.395 | -6.394422311 | 40.88863669 |
| Ephrin-A2 || id:prot-a-897 | rs62143198 | A | G | 19 | 54320939 | -0.1677 | 4.17E-08 | 0.0306 | 0.21453 | -5.480392157 | 30.03469819 |
| Ephrin-A4 || id:prot-a-900 | rs78255607 | C | T | 5 | 32861764 | 0.2404 | 4.47E-08 | 0.0439 | 0.08728 | 5.476082005 | 29.98747412 |
| Ephrin-A5 || id:prot-a-901 | rs71358078 | T | C | 18 | 4960876 | -0.4686 | 3.63E-08 | 0.0851 | 0.02184 | -5.506462985 | 30.3211346 |
| Ephrin-B1 || id:prot-a-903 | rs967645 | T | C | 17 | 26713970 | 0.2136 | 1.29E-18 | 0.0243 | 0.50945 | 8.790123457 | 77.26627039 |
| Ephrin-B2 || id:prot-a-905 | rs62337506 | G | C | 5 | 11469109 | -0.3286 | 4.07E-08 | 0.0599 | 0.04629 | -5.485809683 | 30.09410788 |
| Ephrin-B3 || id:prot-a-906 | rs4632248 | T | G | 19 | 54324995 | -0.1774 | 3.02E-09 | 0.0299 | 0.21439 | -5.933110368 | 35.20179864 |
| Ephrin-B3 || id:prot-a-907 | rs704 | A | G | 17 | 26694861 | 0.3091 | 1.20E-37 | 0.0241 | 0.46665 | 12.82572614 | 164.499251 |
| Ephrin type-A receptor 1 || id:prot-a-956 | rs4421280 | A | G | 7 | 143103217 | 0.8798 | 8.71E-83 | 0.0456 | 0.92708 | 19.29385965 | 372.2530202 |
| Ephrin type-A receptor 2 || id:prot-a-957 | rs143933447 | T | C | 1 | 16492053 | 0.5866 | 3.72E-08 | 0.1066 | 0.01552 | 5.502814259 | 30.28096477 |
| Ephrin type-A receptor 5 || id:prot-a-958 | rs62143197 | A | G | 19 | 54320716 | 0.2448 | 4.90E-16 | 0.0302 | 0.22311 | 8.105960265 | 65.70659182 |
| Ephrin type-B receptor 2 || id:prot-a-960 | rs6687487 | A | G | 1 | 23061551 | -0.4559 | 7.94E-24 | 0.0453 | 0.08032 | -10.06401766 | 101.2844515 |
| Ephrin type-B receptor 2 || id:prot-a-961 | rs6687487 | A | G | 1 | 23061551 | -0.6122 | 1.29E-42 | 0.0447 | 0.08032 | -13.69574944 | 187.5735527 |
| Ephrin type-B receptor 2 || id:prot-a-962 | rs6687487 | A | G | 1 | 23061551 | -0.4064 | 3.72E-19 | 0.0454 | 0.08032 | -8.95154185 | 80.1301015 |
| Ephrin type-B receptor 3 || id:prot-a-963 | rs12882946 | G | A | 14 | 107100923 | -0.2591 | 1.82E-24 | 0.0254 | 0.38619 | -10.2007874 | 104.0560636 |
| Ephrin type-B receptor 6 || id:prot-a-965 | rs8177107 | G | A | 7 | 142554145 | -0.3516 | 8.51E-29 | 0.0316 | 0.17857 | -11.12658228 | 123.8008332 |
| Epidermal growth factor || id:prot-a-908 | rs11568972 | C | A | 4 | 110889007 | 0.2654 | 3.09E-25 | 0.0256 | 0.33681 | 10.3671875 | 107.4785767 |
| Epididymal-specific lipocalin-10 || id:prot-a-1707 | rs570618 | G | T | 1 | 196657064 | -0.1539 | 1.23E-09 | 0.0253 | 0.61149 | -6.083003953 | 37.00293709 |
| Epididymal-specific lipocalin-10 || id:prot-a-1707 | rs74480769 | G | A | 5 | 40972211 | -0.6148 | 3.63E-17 | 0.073 | 0.0317 | -8.421917808 | 70.92869957 |
| Epididymal-specific lipocalin-10 || id:prot-a-1707 | rs429358 | C | T | 19 | 45411941 | 0.3116 | 1.10E-20 | 0.0334 | 0.15301 | 9.329341317 | 87.03660942 |
| Epididymal secretory protein E1 || id:prot-a-2071 | rs1079715 | C | A | 11 | 2760409 | 0.2857 | 3.02E-08 | 0.0516 | 0.06525 | 5.536821705 | 30.6563946 |
| Epididymal secretory protein E1 || id:prot-a-2071 | rs4819094 | G | T | 21 | 46812191 | -0.1941 | 1.62E-11 | 0.0288 | 0.27055 | -6.739583333 | 45.42198351 |
| Epididymal secretory protein E1 || id:prot-a-2071 | rs12483377 | A | G | 21 | 46931109 | 1.4241 | 1.00E-200 | 0.0349 | 0.09016 | 40.80515759 | 1665.060886 |
| Epididymis-specific alpha-mannosidase || id:prot-a-1835 | rs2301790 | G | A | 4 | 6600012 | 0.3342 | 1.00E-44 | 0.0238 | 0.48385 | 14.04201681 | 197.178236 |
| Epididymis-specific alpha-mannosidase || id:prot-a-1835 | rs76316902 | C | T | 15 | 82415574 | 0.4867 | 4.07E-08 | 0.0887 | 0.01961 | 5.487034949 | 30.10755253 |
| Epididymis-specific alpha-mannosidase || id:prot-a-1835 | rs429358 | C | T | 19 | 45411941 | 0.2299 | 7.94E-12 | 0.0336 | 0.15301 | 6.842261905 | 46.81654797 |
| Epiphycan || id:prot-a-970 | rs2839695 | G | A | 10 | 44873849 | -0.2046 | 6.17E-11 | 0.0313 | 0.20133 | -6.536741214 | 42.7289857 |
| Epiphycan || id:prot-a-970 | rs77542162 | G | A | 17 | 67081278 | 0.6913 | 1.78E-15 | 0.0869 | 0.02254 | 7.955120829 | 63.2839474 |
| Epiphycan || id:prot-a-970 | rs72631343 | G | C | 17 | 67191270 | -0.2067 | 9.77E-09 | 0.036 | 0.13098 | -5.741666667 | 32.96673611 |
| Epiregulin || id:prot-a-977 | rs1354034 | C | T | 3 | 56849749 | 0.1548 | 6.46E-10 | 0.0251 | 0.59265 | 6.167330677 | 38.03596768 |
| Epiregulin || id:prot-a-977 | rs78803121 | T | G | 4 | 75248505 | -0.2787 | 3.39E-08 | 0.0505 | 0.06345 | -5.518811881 | 30.45728458 |
| Epiregulin || id:prot-a-977 | rs11792848 | A | G | 9 | 99089943 | -0.163 | 1.35E-08 | 0.0287 | 0.23687 | -5.679442509 | 32.25606721 |
| ER membrane protein complex subunit 1 || id:prot-a-935 | rs941758 | A | C | 4 | 74718941 | -0.3564 | 8.91E-43 | 0.026 | 0.70817 | -13.70769231 | 187.9008284 |
| ER membrane protein complex subunit 4 || id:prot-a-936 | rs4151659 | G | A | 6 | 31918464 | -0.9433 | 2.40E-18 | 0.108 | 0.01288 | -8.734259259 | 76.28728481 |
| ER membrane protein complex subunit 4 || id:prot-a-936 | rs6907185 | G | A | 6 | 31844684 | -0.2755 | 1.95E-10 | 0.0433 | 0.08507 | -6.362586605 | 40.48250831 |
| ER membrane protein complex subunit 4 || id:prot-a-936 | rs1065853 | T | G | 19 | 45413233 | 0.3855 | 5.37E-17 | 0.046 | 0.07778 | 8.380434783 | 70.23168715 |
| ER membrane protein complex subunit 4 || id:prot-a-936 | rs8112351 | C | T | 19 | 6706298 | -0.6591 | 1.00E-135 | 0.0266 | 0.76011 | -24.77819549 | 613.9589717 |
| Erlin-1 || id:prot-a-980 | rs704 | A | G | 17 | 26694861 | 0.1943 | 2.00E-15 | 0.0245 | 0.46665 | 7.930612245 | 62.89461058 |
| ERO1-like protein alpha || id:prot-a-982 | rs7539005 | T | A | 1 | 196667252 | -0.1624 | 1.74E-10 | 0.0254 | 0.61028 | -6.393700787 | 40.87940976 |
| ERO1-like protein alpha || id:prot-a-982 | rs74480769 | G | A | 5 | 40972211 | -0.5322 | 3.55E-13 | 0.0732 | 0.0317 | -7.270491803 | 52.86005106 |
| ERO1-like protein alpha || id:prot-a-982 | rs704 | A | G | 17 | 26694861 | 0.2445 | 9.12E-24 | 0.0243 | 0.46665 | 10.0617284 | 101.2383783 |
| ERO1-like protein beta || id:prot-a-983 | rs1254194 | T | G | 1 | 236399442 | -0.3822 | 1.86E-56 | 0.0241 | 0.59576 | -15.85892116 | 251.5053804 |
| ERO1-like protein beta || id:prot-a-983 | rs7523897 | C | A | 1 | 236314718 | 0.1835 | 4.27E-08 | 0.0335 | NA | 5.47761194 | 30.00423257 |
| ERO1-like protein beta || id:prot-a-983 | rs1801689 | C | A | 17 | 64210580 | 0.5211 | 2.24E-13 | 0.0711 | 0.03043 | 7.329113924 | 53.71591091 |
| Erythrocyte band 7 integral membrane protein || id:prot-a-2882 | rs6770670 | C | T | 3 | 49686682 | -0.4734 | 8.32E-77 | 0.0255 | 0.29355 | -18.56470588 | 344.6483045 |
| Erythroid membrane-associated protein || id:prot-a-981 | rs3917545 | C | A | 7 | 94936235 | 0.2771 | 7.76E-14 | 0.0371 | 0.13088 | 7.469002695 | 55.78600126 |
| Erythropoietin || id:prot-a-967 | rs9373124 | C | T | 6 | 135423209 | 0.1495 | 4.79E-08 | 0.0274 | 0.27679 | 5.45620438 | 29.77016623 |
| Essential MCU regulator, mitochondrial || id:prot-a-2776 | rs10418046 | G | T | 19 | 54327869 | -0.1732 | 6.31E-09 | 0.0298 | 0.21727 | -5.812080537 | 33.78028017 |
| Estrogen sulfotransferase || id:prot-a-2892 | rs73165060 | A | C | 3 | 165481418 | 0.3901 | 9.33E-38 | 0.0304 | 0.20245 | 12.83223684 | 164.6663024 |
| Estrogen sulfotransferase || id:prot-a-2892 | rs704 | A | G | 17 | 26694861 | 0.1414 | 8.71E-09 | 0.0246 | 0.46665 | 5.74796748 | 33.03913015 |
| Estrogen sulfotransferase || id:prot-a-2892 | rs429358 | C | T | 19 | 45411941 | -0.404 | 2.88E-34 | 0.0331 | 0.15301 | -12.20543807 | 148.9727184 |
| ETS domain-containing protein Elk-1 || id:prot-a-930 | rs62143206 | T | G | 19 | 54326212 | 0.416 | 4.27E-46 | 0.0292 | 0.2131 | 14.24657534 | 202.964909 |
| ETS domain-containing protein Elk-3 || id:prot-a-931 | rs62143197 | A | G | 19 | 54320716 | 0.4044 | 2.40E-42 | 0.0297 | 0.22311 | 13.61616162 | 185.3998572 |
| Eukaryotic initiation factor 4A-III || id:prot-a-921 | rs34436714 | A | C | 19 | 54327313 | 0.4687 | 2.00E-58 | 0.0291 | 0.21294 | 16.10652921 | 259.4202832 |
| Eukaryotic translation initiation factor 1A, X-chromosomal || id:prot-a-917 | rs62143197 | A | G | 19 | 54320716 | 0.5793 | 3.02E-90 | 0.0288 | 0.22311 | 20.11458333 | 404.5964627 |
| Eukaryotic translation initiation factor 3 subunit G || id:prot-a-919 | rs62143197 | A | G | 19 | 54320716 | 0.6105 | 2.45E-101 | 0.0286 | 0.22311 | 21.34615385 | 455.658284 |
| Eukaryotic translation initiation factor 3 subunit J || id:prot-a-920 | rs62143197 | A | G | 19 | 54320716 | 0.2548 | 2.88E-17 | 0.0302 | 0.22311 | 8.437086093 | 71.18442174 |
| Eukaryotic translation initiation factor 4 gamma 3 || id:prot-a-925 | rs704 | A | G | 17 | 26694861 | 0.1618 | 4.27E-11 | 0.0245 | 0.46665 | 6.604081633 | 43.61389421 |
| Eukaryotic translation initiation factor 4B || id:prot-a-922 | rs62143197 | A | G | 19 | 54320716 | 0.3766 | 1.10E-36 | 0.0298 | 0.22311 | 12.63758389 | 159.7085266 |
| Eukaryotic translation initiation factor 4E type 2 || id:prot-a-923 | rs62143198 | A | G | 19 | 54320939 | 0.4669 | 6.76E-56 | 0.0296 | 0.21453 | 15.77364865 | 248.8079917 |
| Eukaryotic translation initiation factor 5 || id:prot-a-926 | rs10418046 | G | T | 19 | 54327869 | 0.3292 | 4.68E-29 | 0.0294 | 0.21727 | 11.19727891 | 125.379055 |
| Eukaryotic translation initiation factor 5A-1 || id:prot-a-927 | rs62143197 | A | G | 19 | 54320716 | 0.4575 | 1.55E-54 | 0.0294 | 0.22311 | 15.56122449 | 242.1517076 |
| Exosome complex component RRP40 || id:prot-a-1002 | rs4145487 | T | C | 14 | 106978216 | -0.3323 | 1.78E-32 | 0.028 | 0.24661 | -11.86785714 | 140.8460332 |
| Exostosin-like 2 || id:prot-a-1003 | rs13469 | T | C | 17 | 26676135 | 0.2551 | 2.51E-26 | 0.024 | 0.50763 | 10.62916667 | 112.979184 |
| Extracellular sulfatase Sulf-2 || id:prot-a-2891 | rs7614709 | A | G | 3 | 186393786 | 0.2311 | 1.20E-14 | 0.0299 | 0.21475 | 7.72909699 | 59.73894028 |
| Extracellular sulfatase Sulf-2 || id:prot-a-2891 | rs7971133 | T | C | 12 | 57770098 | -0.2304 | 4.57E-16 | 0.0284 | 0.24029 | -8.112676056 | 65.8155128 |
| Extracellular sulfatase Sulf-2 || id:prot-a-2891 | rs117978438 | A | G | 17 | 52967416 | 0.3311 | 3.80E-08 | 0.0602 | 0.04499 | 5.5 | 30.25 |
| Extracellular sulfatase Sulf-2 || id:prot-a-2891 | rs10424405 | G | A | 19 | 54321933 | 0.3055 | 1.55E-24 | 0.0299 | 0.2203 | 10.2173913 | 104.3950851 |
| Extracellular superoxide dismutase [Cu-Zn] || id:prot-a-2800 | rs2695234 | G | A | 4 | 24804238 | -0.3427 | 8.32E-14 | 0.0459 | 0.90931 | -7.466230937 | 55.7446044 |
| Ezrin || id:prot-a-1004 | rs4632248 | T | G | 19 | 54324995 | 0.5662 | 2.40E-88 | 0.0284 | 0.21439 | 19.93661972 | 397.4688058 |
| F-box/LRR-repeat protein 4 || id:prot-a-1065 | rs844200 | G | A | 3 | 165472248 | 0.1368 | 2.82E-08 | 0.0246 | 0.43216 | 5.56097561 | 30.92444973 |
| F-box/LRR-repeat protein 4 || id:prot-a-1067 | rs34845568 | G | A | 14 | 107154000 | 0.2017 | 2.19E-09 | 0.0337 | 0.20745 | 5.985163205 | 35.82217859 |
| Fas apoptotic inhibitory molecule 3 || id:prot-a-1019 | rs1059510 | C | T | 6 | 30457732 | -0.151 | 2.63E-08 | 0.0271 | 0.67999 | -5.57195572 | 31.04669054 |
| Fatty acid-binding protein, liver || id:prot-a-1011 | rs2241883 | C | T | 2 | 88424066 | -0.1905 | 2.40E-12 | 0.0272 | 0.30311 | -7.003676471 | 49.0514841 |
| Fc receptor-like protein 1 || id:prot-a-1079 | rs4971155 | A | T | 1 | 157779182 | -0.2557 | 6.31E-26 | 0.0243 | 0.50623 | -10.52263374 | 110.7258209 |
| Fc receptor-like protein 3 || id:prot-a-1081 | rs7528684 | G | A | 1 | 157670816 | 0.5253 | 1.38E-112 | 0.0233 | 0.46511 | 22.54506438 | 508.2799278 |
| Fc receptor-like protein 3 || id:prot-a-1081 | rs3851215 | G | A | 6 | 108039036 | 0.1526 | 1.82E-08 | 0.0271 | 0.71584 | 5.63099631 | 31.70811944 |
| Fc receptor-like protein 4 || id:prot-a-1082 | rs11582663 | T | C | 1 | 157559122 | -1.1329 | 1.00E-200 | 0.0295 | 0.14428 | -38.40338983 | 1474.82035 |
| Fc receptor-like protein 6 || id:prot-a-1084 | rs58240276 | T | C | 1 | 159783559 | -0.6094 | 3.09E-92 | 0.0299 | 0.19016 | -20.3812709 | 415.3962036 |
| Fc receptor-like protein 6 || id:prot-a-1084 | rs2732515 | A | G | 4 | 102055978 | 0.1495 | 8.91E-09 | 0.026 | 0.41914 | 5.75 | 33.0625 |
| Ferritin || id:prot-a-1148 | rs217181 | T | C | 16 | 72114002 | 0.5241 | 7.41E-69 | 0.0299 | 0.19567 | 17.52842809 | 307.2457914 |
| Ferritin || id:prot-a-1148 | rs7202724 | G | A | 16 | 72030439 | -0.1893 | 1.62E-11 | 0.0281 | 0.32574 | -6.736654804 | 45.38251795 |
| Ferritin || id:prot-a-1148 | rs241775 | T | C | 17 | 26644668 | -0.1637 | 1.78E-11 | 0.0244 | 0.47792 | -6.709016393 | 45.01090097 |
| Fibrinogen C domain-containing protein 1 || id:prot-a-1108 | rs9461792 | A | G | 6 | 32636495 | 0.1533 | 1.62E-08 | 0.0271 | 0.29997 | 5.656826568 | 31.99968682 |
| Fibroblast growth factor 1 || id:prot-a-1088 | rs115973681 | C | A | 4 | 68406861 | -0.5829 | 4.37E-08 | 0.1064 | 0.01643 | -5.478383459 | 30.01268532 |
| Fibroblast growth factor 1 || id:prot-a-1088 | rs62143197 | A | G | 19 | 54320716 | -0.1876 | 6.03E-10 | 0.0303 | 0.22311 | -6.191419142 | 38.33367099 |
| Fibroblast growth factor 16 || id:prot-a-1089 | rs4632248 | T | G | 19 | 54324995 | -0.2197 | 1.78E-13 | 0.0298 | 0.21439 | -7.372483221 | 54.35350885 |
| Fibroblast growth factor 19 || id:prot-a-1091 | rs601338 | A | G | 19 | 49206674 | -0.1797 | 1.66E-13 | 0.0244 | 0.50711 | -7.364754098 | 54.23960293 |
| Fibroblast growth factor 7 || id:prot-a-1097 | rs11785665 | T | A | 8 | 3208225 | -0.1899 | 1.41E-09 | 0.0314 | 0.18538 | -6.047770701 | 36.57553045 |
| Fibroblast growth factor 7 || id:prot-a-1097 | rs12899680 | G | C | 15 | 49871878 | -0.1751 | 1.70E-12 | 0.0248 | 0.48101 | -7.060483871 | 49.85043249 |
| Fibroblast growth factor 7 || id:prot-a-1097 | rs7162172 | C | T | 15 | 50364841 | -0.1856 | 6.76E-13 | 0.0258 | 0.3919 | -7.19379845 | 51.75073613 |
| Fibroblast growth factor 7 || id:prot-a-1097 | rs11287932 | CA | C | 17 | 17955394 | -0.1657 | 2.00E-09 | 0.0276 | 0.42998 | -6.003623188 | 36.04349139 |
| Fibroblast growth factor 7 || id:prot-a-1097 | rs138322808 | C | T | 19 | 55491031 | -0.5692 | 1.17E-08 | 0.0998 | 0.01777 | -5.703406814 | 32.52884928 |
| Fibroblast growth factor 8 isoform A || id:prot-a-1100 | rs34761731 | G | A | 14 | 106526933 | 0.2006 | 1.15E-15 | 0.025 | 0.41029 | 8.024 | 64.384576 |
| Fibroblast growth factor 8 isoform B || id:prot-a-1099 | rs10801557 | T | C | 1 | 196669581 | -0.1413 | 2.57E-08 | 0.0254 | 0.61207 | -5.562992126 | 30.94688139 |
| Fibroblast growth factor 8 isoform B || id:prot-a-1099 | rs62143196 | G | A | 19 | 54320636 | -0.1712 | 1.55E-08 | 0.0303 | 0.22404 | -5.650165017 | 31.92436471 |
| Fibroblast growth factor receptor 2 || id:prot-a-1102 | rs148039675 | T | C | 12 | 78187772 | -0.6247 | 4.47E-09 | 0.1065 | 0.01647 | -5.8657277 | 34.40676145 |
| Fibroblast growth factor receptor 3 || id:prot-a-1103 | rs4533828 | A | G | 4 | 1762974 | -0.1596 | 4.07E-10 | 0.0255 | 0.58161 | -6.258823529 | 39.17287197 |
| Fibroblast growth factor receptor 3 || id:prot-a-1103 | rs71446763 | A | G | 12 | 21418398 | 0.2031 | 1.35E-08 | 0.0358 | 0.14059 | 5.673184358 | 32.18502075 |
| Fibroblast growth factor receptor 3 || id:prot-a-1104 | rs7259081 | A | T | 19 | 54339243 | 0.1811 | 8.32E-09 | 0.0314 | 0.75126 | 5.767515924 | 33.26423993 |
| Fibronectin type III domain-containing protein 5 || id:prot-a-1133 | rs10500671 | A | C | 11 | 6460026 | 0.2425 | 1.66E-16 | 0.0294 | 0.24219 | 8.24829932 | 68.03444167 |
| Ficolin-1 || id:prot-a-1077 | rs11103564 | C | T | 9 | 137779875 | 0.155 | 9.55E-09 | 0.027 | 0.30287 | 5.740740741 | 32.95610425 |
| Ficolin-1 || id:prot-a-1077 | rs11103602 | A | G | 9 | 137854872 | 0.5827 | 1.55E-106 | 0.0266 | 0.25802 | 21.90601504 | 479.8734948 |
| Ficolin-2 || id:prot-a-1078 | rs57136797 | T | A | 9 | 137752540 | -0.6346 | 3.72E-72 | 0.0353 | 0.164 | -17.97733711 | 323.1846496 |
| Ficolin-2 || id:prot-a-1078 | rs7041633 | C | G | 9 | 137776213 | -0.1488 | 1.12E-09 | 0.0244 | 0.43681 | -6.098360656 | 37.19000269 |
| Ficolin-2 || id:prot-a-1078 | rs10891700 | T | C | 11 | 114437886 | -0.3994 | 7.41E-29 | 0.0358 | 0.13204 | -11.15642458 | 124.4658094 |
| Filamin-A || id:prot-a-1117 | rs12038333 | A | G | 1 | 196672454 | -0.1444 | 1.26E-08 | 0.0254 | 0.6091 | -5.68503937 | 32.31967264 |
| Filamin-A || id:prot-a-1117 | rs854921 | C | G | 6 | 90123586 | 0.1414 | 4.68E-08 | 0.0259 | 0.41075 | 5.459459459 | 29.80569759 |
| Filamin-A || id:prot-a-1117 | rs704 | A | G | 17 | 26694861 | 0.1628 | 3.24E-11 | 0.0245 | 0.46665 | 6.644897959 | 44.15466889 |
| Filamin-A || id:prot-a-1118 | rs967645 | T | C | 17 | 26713970 | -0.1752 | 6.31E-13 | 0.0243 | 0.50945 | -7.209876543 | 51.98231977 |
| Follicle stimulating hormone || id:prot-a-528 | rs189852876 | A | G | 3 | 146877544 | 0.5283 | 5.62E-09 | 0.0906 | 0.0213 | 5.831125828 | 34.00202842 |
| Follicle stimulating hormone || id:prot-a-528 | rs6853458 | G | A | 4 | 163414028 | 0.3359 | 3.89E-08 | 0.0611 | 0.95682 | 5.497545008 | 30.22300112 |
| Follistatin-related protein 1 || id:prot-a-1143 | rs1147707 | T | C | 3 | 120169248 | -0.2113 | 1.12E-16 | 0.0255 | 0.38909 | -8.28627451 | 68.66234525 |
| Follistatin-related protein 1 || id:prot-a-1143 | rs62143198 | A | G | 19 | 54320939 | -0.171 | 2.29E-08 | 0.0306 | 0.21453 | -5.588235294 | 31.2283737 |
| Follistatin-related protein 4 || id:prot-a-1146 | rs3749817 | A | G | 5 | 132535046 | -0.1944 | 1.51E-10 | 0.0304 | 0.20412 | -6.394736842 | 40.89265928 |
| Follistatin-related protein 4 || id:prot-a-1146 | rs10418046 | G | T | 19 | 54327869 | -0.2085 | 2.45E-12 | 0.0298 | 0.21727 | -6.996644295 | 48.95303139 |
| Follistatin || id:prot-a-1142 | rs117679020 | A | G | 17 | 76121558 | -0.2967 | 1.07E-08 | 0.0519 | 0.07131 | -5.716763006 | 32.68137926 |
| Forkhead box protein J2 || id:prot-a-1137 | rs6003387 | G | C | 22 | 23251602 | -0.6671 | 5.62E-33 | 0.0558 | 0.05613 | -11.95519713 | 142.9267385 |
| Forkhead box protein J2 || id:prot-a-1137 | rs77018536 | G | A | 22 | 23100442 | -0.1783 | 5.75E-11 | 0.0272 | 0.58386 | -6.555147059 | 42.96995296 |
| Forkhead box protein L2 || id:prot-a-1138 | rs35029497 | TG | T | 3 | 16373853 | 0.1527 | 1.07E-08 | 0.0267 | 0.68189 | 5.719101124 | 32.70811766 |
| Four-jointed box protein 1 || id:prot-a-1112 | rs12740374 | T | G | 1 | 109817590 | -0.2635 | 1.51E-18 | 0.03 | 0.22252 | -8.783333333 | 77.14694444 |
| Four-jointed box protein 1 || id:prot-a-1112 | rs6556224 | G | T | 5 | 175186350 | 0.1546 | 4.27E-08 | 0.0282 | 0.71356 | 5.482269504 | 30.05527891 |
| Four-jointed box protein 1 || id:prot-a-1112 | rs12799443 | T | C | 11 | 36090287 | 0.1726 | 7.08E-11 | 0.0265 | 0.41094 | 6.513207547 | 42.42187255 |
| Fractalkine || id:prot-a-736 | rs74480769 | G | A | 5 | 40972211 | -0.42 | 1.05E-08 | 0.0734 | 0.0317 | -5.722070845 | 32.74209475 |
| Fractalkine || id:prot-a-736 | rs62037115 | A | G | 16 | 57458796 | 0.9123 | 4.68E-11 | 0.1387 | 0.00874 | 6.577505407 | 43.26357738 |
| Fragile X mental retardation syndrome-related protein 1 || id:prot-a-1156 | rs920558 | T | C | 4 | 102320930 | -0.2714 | 3.47E-08 | 0.0492 | 0.07087 | -5.516260163 | 30.42912618 |
| Fructose-1,6-bisphosphatase 1 || id:prot-a-1063 | rs10424405 | G | A | 19 | 54321933 | 0.2659 | 7.76E-19 | 0.03 | 0.2203 | 8.863333333 | 78.55867778 |
| Fructose-1,6-bisphosphatase isozyme 2 || id:prot-a-1064 | rs10418046 | G | T | 19 | 54327869 | -0.1641 | 3.89E-08 | 0.0298 | 0.21727 | -5.506711409 | 30.32387055 |
| Fructose-2,6-bisphosphatase TIGAR || id:prot-a-2979 | rs17690319 | A | G | 7 | 41118061 | 0.3254 | 2.45E-08 | 0.0583 | 0.05657 | 5.581475129 | 31.15286461 |
| Fructose-2,6-bisphosphatase TIGAR || id:prot-a-2979 | rs62143198 | A | G | 19 | 54320939 | 0.5725 | 2.75E-86 | 0.0291 | 0.21453 | 19.67353952 | 387.0481572 |
| Fructose-bisphosphate aldolase A || id:prot-a-77 | rs62143197 | A | G | 19 | 54320716 | 0.3011 | 1.15E-23 | 0.03 | 0.22311 | 10.03666667 | 100.7346778 |
| Fumarylacetoacetase || id:prot-a-1018 | rs11555096 | T | C | 15 | 80472526 | -1.8824 | 1.95E-121 | 0.0803 | 0.02128 | -23.44209215 | 549.5316846 |
| Furin || id:prot-a-1150 | rs4554007 | A | G | 3 | 165484401 | 0.2119 | 6.92E-12 | 0.0309 | 0.20298 | 6.857605178 | 47.02674878 |
| Furin || id:prot-a-1150 | rs5167 | G | T | 19 | 45448465 | 0.3386 | 8.32E-41 | 0.0253 | 0.34696 | 13.38339921 | 179.1153744 |
| G2/mitotic-specific cyclin-B1 || id:prot-a-413 | rs62143206 | T | G | 19 | 54326212 | -0.1702 | 1.29E-08 | 0.0299 | 0.2131 | -5.692307692 | 32.40236686 |
| Galactoside 3(4)-L-fucosyltransferase || id:prot-a-1152 | rs708686 | T | C | 19 | 5840619 | -0.853 | 1.00E-200 | 0.0242 | 0.27402 | -35.24793388 | 1242.416843 |
| Galactoside 3(4)-L-fucosyltransferase || id:prot-a-1152 | rs679574 | G | C | 19 | 49206108 | -0.1994 | 2.09E-16 | 0.0243 | 0.50693 | -8.205761317 | 67.33451879 |
| Galactosylgalactosylxylosylprotein 3-beta-glucuronosyltransferase 3 || id:prot-a-211 | rs12134598 | G | T | 1 | 196681001 | 0.1899 | 4.57E-09 | 0.0324 | 0.17352 | 5.861111111 | 34.35262346 |
| Galactosylgalactosylxylosylprotein 3-beta-glucuronosyltransferase 3 || id:prot-a-211 | rs12794886 | C | G | 11 | 62383715 | 0.4422 | 8.32E-63 | 0.0264 | 0.70703 | 16.75 | 280.5625 |
| Galanin-like peptide || id:prot-a-1174 | rs139148537 | G | GT | 5 | 72460992 | -0.5747 | 1.23E-09 | 0.0946 | 0.9824 | -6.075052854 | 36.90626718 |
| Galanin-like peptide || id:prot-a-1174 | rs967645 | T | C | 17 | 26713970 | -0.16 | 5.25E-11 | 0.0244 | 0.50945 | -6.557377049 | 42.99919377 |
| Galanin-like peptide || id:prot-a-1174 | rs12721111 | T | C | 19 | 45446271 | -0.1633 | 1.78E-09 | 0.0271 | 0.30454 | -6.025830258 | 36.3106303 |
| Galanin-like peptide || id:prot-a-1174 | rs111265125 | C | G | 19 | 56688781 | 0.6468 | 1.02E-12 | 0.0907 | 0.02182 | 7.131201764 | 50.8540386 |
| Galanin peptides || id:prot-a-1166 | rs72708013 | G | T | 1 | 169481731 | -0.3311 | 1.17E-09 | 0.0544 | 0.05472 | -6.086397059 | 37.04422916 |
| Galanin peptides || id:prot-a-1166 | rs2205895 | T | C | 1 | 169572645 | -0.1816 | 1.45E-12 | 0.0257 | 0.35288 | -7.06614786 | 49.93044558 |
| Galanin peptides || id:prot-a-1166 | rs1409338 | G | A | 10 | 20227267 | 0.1847 | 1.74E-11 | 0.0275 | 0.71115 | 6.716363636 | 45.1095405 |
| Galectin-10 || id:prot-a-567 | rs10424405 | G | A | 19 | 54321933 | 0.1989 | 4.17E-11 | 0.0302 | 0.2203 | 6.586092715 | 43.37661725 |
| Galectin-2 || id:prot-a-1727 | rs10424405 | G | A | 19 | 54321933 | 0.2309 | 1.66E-14 | 0.0301 | 0.2203 | 7.671096346 | 58.84571914 |
| Galectin-2 || id:prot-a-1727 | rs5756729 | T | C | 22 | 37961353 | -0.275 | 2.24E-26 | 0.0259 | 0.59251 | -10.61776062 | 112.7368405 |
| Galectin-4 || id:prot-a-1728 | rs7251903 | A | G | 19 | 39184811 | -0.136 | 3.09E-08 | 0.0246 | 0.48481 | -5.528455285 | 30.56381783 |
| Galectin-7 || id:prot-a-1729 | rs6993770 | T | A | 8 | 106581528 | -0.2375 | 2.63E-18 | 0.0272 | 0.28225 | -8.731617647 | 76.24114673 |
| Galectin-7 || id:prot-a-1729 | rs2393969 | C | A | 10 | 65140440 | 0.1392 | 3.02E-08 | 0.0251 | 0.47282 | 5.545816733 | 30.75608324 |
| Galectin-9 || id:prot-a-1732 | rs12034435 | T | C | 1 | 9424050 | -0.1905 | 9.12E-09 | 0.0332 | 0.16545 | -5.737951807 | 32.92409094 |
| Galectin-9 || id:prot-a-1732 | rs201180660 | G | GT | 2 | 196505218 | -0.2955 | 1.58E-08 | 0.0523 | 0.93613 | -5.650095602 | 31.92358032 |
| Galectin-9 || id:prot-a-1732 | rs3794195 | G | A | 17 | 25959355 | -0.2101 | 3.72E-13 | 0.0289 | 0.24391 | -7.269896194 | 52.85139067 |
| Gamma-aminobutyric acid receptor-associated protein-like 1 || id:prot-a-1161 | rs4632248 | T | G | 19 | 54324995 | 0.6953 | 1.00E-140 | 0.0275 | 0.21439 | 25.28363636 | 639.2622678 |
| Gamma-aminobutyric acid receptor-associated protein-like 2 || id:prot-a-1162 | rs62143197 | A | G | 19 | 54320716 | 0.292 | 2.51E-22 | 0.03 | 0.22311 | 9.733333333 | 94.73777778 |
| Gamma-aminobutyric acid type B receptor subunit 2 || id:prot-a-1163 | rs12534274 | A | G | 7 | 95057510 | 0.1621 | 7.24E-09 | 0.028 | 0.2688 | 5.789285714 | 33.51582908 |
| Gamma-aminobutyric acid type B receptor subunit 2 || id:prot-a-1163 | rs4259951 | A | C | 14 | 107172874 | -0.2379 | 1.66E-16 | 0.0289 | 0.7061 | -8.23183391 | 67.76308952 |
| Gamma-crystallin D || id:prot-a-675 | rs2420929 | C | T | 10 | 123153176 | -0.2419 | 4.17E-08 | 0.0441 | 0.90794 | -5.485260771 | 30.08808573 |
| Gamma-enolase || id:prot-a-944 | rs150012467 | G | A | 8 | 103921218 | -0.4104 | 3.39E-08 | 0.0743 | 0.03288 | -5.523553163 | 30.50963954 |
| Gamma-enolase || id:prot-a-945 | rs62143197 | A | G | 19 | 54320716 | 0.2118 | 2.51E-12 | 0.0303 | 0.22311 | 6.99009901 | 48.86148417 |
| Gamma-glutamyl hydrolase || id:prot-a-1207 | rs201996075 | A | AT | 8 | 64026934 | 0.5944 | 1.10E-23 | 0.0592 | 0.95038 | 10.04054054 | 100.8124543 |
| Gamma-glutamyl hydrolase || id:prot-a-1207 | rs10957266 | C | T | 8 | 63923764 | 0.7985 | 1.23E-68 | 0.0456 | 0.0713 | 17.51096491 | 306.6338922 |
| Gamma-glutamyl hydrolase || id:prot-a-1207 | rs7964859 | G | C | 12 | 102220783 | -0.2103 | 8.91E-15 | 0.0271 | 0.2939 | -7.760147601 | 60.2198908 |
| Gamma-interferon-inducible protein 16 || id:prot-a-1414 | rs72709516 | T | C | 1 | 159004851 | 0.8518 | 2.63E-49 | 0.0577 | 0.04404 | 14.76256499 | 217.9333251 |
| Gamma-interferon-inducible protein 16 || id:prot-a-1414 | rs62143198 | A | G | 19 | 54320939 | 0.6405 | 1.02E-110 | 0.0286 | 0.21453 | 22.3951049 | 501.5407233 |
| Gamma-interferon-inducible protein 16 || id:prot-a-1414 | rs4809344 | G | C | 20 | 62413734 | 0.2501 | 2.75E-08 | 0.045 | 0.91762 | 5.557777778 | 30.88889383 |
| Gamma-interferon-inducible protein 16 || id:prot-a-1415 | rs10424405 | G | A | 19 | 54321933 | 0.3717 | 4.90E-36 | 0.0297 | 0.2203 | 12.51515152 | 156.6290174 |
| Gap junction alpha-1 protein || id:prot-a-1213 | rs2019065 | C | T | 3 | 52809525 | 0.2079 | 2.09E-16 | 0.0253 | 0.39181 | 8.217391304 | 67.52551985 |
| Gap junction alpha-1 protein || id:prot-a-1213 | rs158100 | A | G | 14 | 77544030 | 0.1499 | 4.47E-08 | 0.0274 | 0.674 | 5.47080292 | 29.92968459 |
| Gastrin-releasing peptide || id:prot-a-1279 | rs7080536 | A | G | 10 | 115348046 | -0.6519 | 3.72E-26 | 0.0616 | 0.04384 | -10.58279221 | 111.9954909 |
| Gastrin-releasing peptide || id:prot-a-1280 | rs1863622 | C | T | 3 | 186396616 | 0.2499 | 1.62E-16 | 0.0303 | 0.21611 | 8.247524752 | 68.02166454 |
| Gastrin-releasing peptide || id:prot-a-1280 | rs704 | A | G | 17 | 26694861 | 0.1707 | 3.24E-12 | 0.0245 | 0.46665 | 6.967346939 | 48.54392337 |
| GDH/6PGL endoplasmic bifunctional protein || id:prot-a-1305 | rs34603401 | C | A | 1 | 9305445 | 0.7589 | 1.00E-126 | 0.0317 | 0.15022 | 23.94006309 | 573.1266208 |
| GDH/6PGL endoplasmic bifunctional protein || id:prot-a-1305 | rs9435138 | A | G | 1 | 9272231 | 0.2492 | 3.31E-24 | 0.0246 | 0.59697 | 10.1300813 | 102.6185472 |
| GDH/6PGL endoplasmic bifunctional protein || id:prot-a-1305 | rs149062332 | C | T | 3 | 186341025 | 1.6175 | 1.95E-46 | 0.113 | 0.01216 | 14.31415929 | 204.8951562 |
| GDH/6PGL endoplasmic bifunctional protein || id:prot-a-1305 | rs9271421 | C | T | 6 | 32587859 | -0.3948 | 4.90E-29 | 0.0353 | 0.86075 | -11.18413598 | 125.0848976 |
| GDH/6PGL endoplasmic bifunctional protein || id:prot-a-1305 | rs10128781 | C | T | 12 | 29520706 | 0.2766 | 8.51E-25 | 0.0269 | 0.28947 | 10.28252788 | 105.7303796 |
| GDNF family receptor alpha-1 || id:prot-a-1201 | rs10885877 | G | C | 10 | 117966090 | 0.2645 | 2.04E-22 | 0.0272 | 0.29546 | 9.724264706 | 94.56132407 |
| GDNF family receptor alpha-2 || id:prot-a-1202 | rs111959275 | T | G | 6 | 32618796 | -0.2084 | 3.63E-08 | 0.0378 | 0.13018 | -5.513227513 | 30.39567761 |
| GDNF family receptor alpha-2 || id:prot-a-1202 | rs15881 | C | A | 8 | 21550768 | 0.2954 | 4.07E-33 | 0.0246 | 0.4556 | 12.00813008 | 144.195188 |
| GDNF family receptor alpha-2 || id:prot-a-1202 | rs202184555 | G | GT | 8 | 21685174 | 0.2167 | 7.94E-16 | 0.0269 | 0.70271 | 8.055762082 | 64.89530272 |
| GDNF family receptor alpha-like || id:prot-a-1204 | rs11967574 | T | C | 6 | 55505472 | 0.1768 | 6.03E-11 | 0.027 | 0.29474 | 6.548148148 | 42.87824417 |
| GDNF family receptor alpha-like || id:prot-a-1204 | rs12206196 | G | A | 6 | 55316182 | -0.2286 | 8.91E-14 | 0.0307 | 0.19563 | -7.446254072 | 55.4466997 |
| GDNF family receptor alpha-like || id:prot-a-1204 | rs8176672 | T | C | 9 | 136142185 | 0.3704 | 8.71E-14 | 0.0496 | 0.06653 | 7.467741935 | 55.76716961 |
| GDP-fucose protein O-fucosyltransferase 1 || id:prot-a-2318 | rs34436714 | A | C | 19 | 54327313 | -0.3246 | 7.59E-28 | 0.0297 | 0.21294 | -10.92929293 | 119.4494439 |
| GDP-fucose protein O-fucosyltransferase 1 || id:prot-a-2318 | rs76143353 | T | C | 20 | 30815755 | -0.8 | 9.33E-58 | 0.0499 | 0.05956 | -16.03206413 | 257.0270802 |
| GDP-fucose protein O-fucosyltransferase 2 || id:prot-a-2319 | rs4395853 | T | C | 8 | 27808770 | -0.2583 | 2.24E-21 | 0.0272 | 0.72286 | -9.496323529 | 90.18016058 |
| GDP-fucose protein O-fucosyltransferase 2 || id:prot-a-2319 | rs1138545 | T | C | 9 | 117835899 | -0.806 | 6.17E-146 | 0.0313 | 0.15343 | -25.75079872 | 663.1036348 |
| General transcription factor II-I || id:prot-a-1292 | rs62143197 | A | G | 19 | 54320716 | 0.4336 | 8.13E-49 | 0.0295 | 0.22311 | 14.69830508 | 216.0401724 |
| Glia-derived nexin || id:prot-a-2697 | rs68066031 | C | T | 2 | 224880498 | -0.4261 | 1.07E-46 | 0.0297 | 0.258 | -14.34680135 | 205.8307089 |
| Glia-derived nexin || id:prot-a-2697 | rs6993770 | T | A | 8 | 106581528 | -0.1556 | 1.35E-08 | 0.0274 | 0.28225 | -5.678832117 | 32.24913421 |
| Glia maturation factor gamma || id:prot-a-1225 | rs62143206 | T | G | 19 | 54326212 | 0.6062 | 8.51E-103 | 0.0282 | 0.2131 | 21.4964539 | 462.0975303 |
| Glucagon-like peptide 1 receptor || id:prot-a-1219 | rs59753727 | A | C | 10 | 84019699 | 0.3869 | 4.17E-08 | 0.0705 | 0.0409 | 5.487943262 | 30.11752125 |
| Glucagon || id:prot-a-1181 | rs139276089 | C | G | 4 | 187160394 | -0.2308 | 5.62E-21 | 0.0246 | 0.51244 | -9.382113821 | 88.02405975 |
| Glucagon || id:prot-a-1181 | rs2731674 | G | T | 5 | 176839890 | -0.1849 | 4.90E-11 | 0.0281 | 0.75301 | -6.580071174 | 43.29733666 |
| Glucokinase || id:prot-a-1183 | rs12038333 | A | G | 1 | 196672454 | -0.1899 | 5.75E-14 | 0.0253 | 0.6091 | -7.505928854 | 56.33896796 |
| Glucokinase regulatory protein || id:prot-a-1184 | rs62143197 | A | G | 19 | 54320716 | -0.1908 | 3.02E-10 | 0.0303 | 0.22311 | -6.297029703 | 39.65258308 |
| Glucosamine-6-phosphate isomerase 1 || id:prot-a-1230 | rs62143197 | A | G | 19 | 54320716 | 0.2659 | 1.10E-18 | 0.0301 | 0.22311 | 8.833887043 | 78.03756029 |
| Glucosamine 6-phosphate N-acetyltransferase || id:prot-a-1231 | rs11789587 | T | C | 9 | 3088740 | 0.1437 | 4.47E-08 | 0.0263 | 0.43254 | 5.463878327 | 29.85396637 |
| Glucosamine 6-phosphate N-acetyltransferase || id:prot-a-1231 | rs62143197 | A | G | 19 | 54320716 | 0.3885 | 4.79E-39 | 0.0297 | 0.22311 | 13.08080808 | 171.10754 |
| Glucose-6-phosphate isomerase || id:prot-a-1252 | rs62143198 | A | G | 19 | 54320939 | 0.6197 | 8.71E-103 | 0.0288 | 0.21453 | 21.51736111 | 462.9968292 |
| Glucosidase 2 subunit beta || id:prot-a-2372 | rs71640034 | A | G | 4 | 187161048 | 0.2942 | 1.58E-33 | 0.0244 | 0.51294 | 12.05737705 | 145.3803413 |
| Glucosidase 2 subunit beta || id:prot-a-2372 | rs1801020 | G | A | 5 | 176836532 | 0.2607 | 1.74E-20 | 0.0281 | 0.75032 | 9.277580071 | 86.07349198 |
| Glucoside xylosyltransferase 1 || id:prot-a-1295 | rs10745925 | C | T | 12 | 102218899 | -0.3531 | 3.31E-40 | 0.0266 | 0.29402 | -13.27443609 | 176.2106535 |
| Glucoside xylosyltransferase 1 || id:prot-a-1295 | rs67420641 | A | G | 12 | 42536064 | 0.4131 | 2.29E-38 | 0.0319 | 0.17887 | 12.94984326 | 167.6984405 |
| Glutamate carboxypeptidase 2 || id:prot-a-1134 | rs7539005 | T | A | 1 | 196667252 | -0.1611 | 2.45E-10 | 0.0254 | 0.61028 | -6.342519685 | 40.22755596 |
| Glutamate carboxypeptidase 2 || id:prot-a-1134 | rs74480769 | G | A | 5 | 40972211 | -0.6134 | 4.27E-17 | 0.073 | 0.0317 | -8.402739726 | 70.6060349 |
| Glutamate receptor 4 || id:prot-a-1275 | rs3132451 | C | G | 6 | 31582025 | -0.4586 | 1.66E-55 | 0.0292 | 0.21656 | -15.70547945 | 246.6620848 |
| Glutamate receptor 4 || id:prot-a-1275 | rs28361034 | G | A | 6 | 31974723 | 0.3649 | 6.46E-36 | 0.0292 | 0.72248 | 12.49657534 | 156.1643953 |
| Glutamate receptor 4 || id:prot-a-1275 | rs143133357 | C | T | 6 | 32625820 | 0.2313 | 3.80E-10 | 0.0369 | 0.14669 | 6.268292683 | 39.29149316 |
| Glutamate receptor ionotropic, delta-2 || id:prot-a-1276 | rs9295963 | C | T | 6 | 31169494 | 0.1412 | 1.82E-08 | 0.0251 | 0.39134 | 5.625498008 | 31.64622784 |
| Glutamate receptor ionotropic, delta-2 || id:prot-a-1276 | rs117804109 | C | T | 14 | 106721892 | 0.6538 | 5.75E-14 | 0.087 | 0.02449 | 7.514942529 | 56.47436121 |
| Glutamate receptor ionotropic, delta-2 || id:prot-a-1276 | rs4802423 | G | A | 19 | 48547651 | 0.2063 | 8.32E-09 | 0.0358 | 0.85567 | 5.762569832 | 33.20721107 |
| Glutamate receptor ionotropic, delta-2 || id:prot-a-1276 | rs6037651 | T | C | 20 | 3686436 | 0.2697 | 2.40E-27 | 0.0249 | 0.58198 | 10.8313253 | 117.3176078 |
| Glutaminyl-peptide cyclotransferase-like protein || id:prot-a-2466 | rs17850756 | A | G | 19 | 46206262 | -0.2881 | 1.58E-28 | 0.026 | 0.32791 | -11.08076923 | 122.7834467 |
| Glutaminyl-peptide cyclotransferase || id:prot-a-2465 | rs3770749 | G | A | 2 | 37584705 | -1.0872 | 7.08E-09 | 0.1877 | 0.00448 | -5.79222163 | 33.54983141 |
| Glutaredoxin-2, mitochondrial || id:prot-a-1220 | rs148212596 | G | A | 1 | 193074511 | 1.1837 | 3.55E-41 | 0.0881 | 0.0198 | 13.43586833 | 180.5225578 |
| Glutaredoxin-2, mitochondrial || id:prot-a-1220 | rs62143197 | A | G | 19 | 54320716 | 0.381 | 1.48E-37 | 0.0297 | 0.22311 | 12.82828283 | 164.5648403 |
| Glutaredoxin-like protein C5orf63 || id:prot-a-317 | rs704 | A | G | 17 | 26694861 | -0.167 | 9.55E-12 | 0.0245 | 0.46665 | -6.816326531 | 46.46230737 |
| Glutathione peroxidase 7 || id:prot-a-1265 | rs1097234 | A | C | 1 | 53063559 | 0.5633 | 1.62E-73 | 0.0311 | 0.17474 | 18.11254019 | 328.0641122 |
| Glutathione S-transferase A1 || id:prot-a-1283 | rs2290758 | A | G | 6 | 52662153 | 0.4157 | 7.24E-69 | 0.0237 | 0.57002 | 17.54008439 | 307.6545603 |
| Glutathione S-transferase A1 || id:prot-a-1283 | rs79104405 | A | G | 9 | 2379204 | 0.4456 | 4.68E-08 | 0.0816 | 0.0236 | 5.460784314 | 29.82016532 |
| Glutathione S-transferase A4 || id:prot-a-1285 | rs74480769 | G | A | 5 | 40972211 | -0.4166 | 1.38E-08 | 0.0734 | 0.0317 | -5.675749319 | 32.21413033 |
| Glutathione S-transferase A4 || id:prot-a-1285 | rs241775 | T | C | 17 | 26644668 | -0.1783 | 2.29E-13 | 0.0243 | 0.47792 | -7.33744856 | 53.83815137 |
| Glutathione S-transferase Mu 1 || id:prot-a-1287 | rs2234696 | G | T | 1 | 110279602 | 0.2874 | 1.05E-10 | 0.0445 | 0.08226 | 6.458426966 | 41.71127888 |
| Glutathione S-transferase Mu 1 || id:prot-a-1287 | rs483082 | T | G | 19 | 45416178 | 0.2726 | 3.72E-21 | 0.0289 | 0.23119 | 9.432525952 | 88.97254583 |
| Glutathione S-transferase omega-1 || id:prot-a-1288 | rs72809840 | A | G | 10 | 106282325 | -0.5416 | 1.10E-08 | 0.0948 | 0.01814 | -5.713080169 | 32.63928501 |
| Glutathione S-transferase omega-1 || id:prot-a-1288 | rs2282326 | C | A | 10 | 106020398 | -0.9108 | 1.00E-200 | 0.0204 | 0.35354 | -44.64705882 | 1993.359862 |
| Glutathione S-transferase omega-1 || id:prot-a-1288 | rs141731775 | A | G | 10 | 105836606 | 0.5477 | 2.88E-08 | 0.0987 | 0.01625 | 5.549138804 | 30.79294147 |
| Glutathione S-transferase P || id:prot-a-1289 | rs11265492 | A | G | 1 | 160799274 | 0.1935 | 1.35E-10 | 0.0301 | 0.21458 | 6.428571429 | 41.32653061 |
| Glutathione S-transferase P || id:prot-a-1290 | rs1695 | G | A | 11 | 67352689 | -0.1781 | 2.34E-12 | 0.0254 | 0.34538 | -7.011811024 | 49.16549383 |
| Glutathione S-transferase P || id:prot-a-1290 | rs62143206 | T | G | 19 | 54326212 | 0.5436 | 7.41E-81 | 0.0285 | 0.2131 | 19.07368421 | 363.8054294 |
| Glutathione S-transferase theta-2B || id:prot-a-1291 | rs139188992 | C | G | 8 | 139894405 | -0.7191 | 5.13E-09 | 0.123 | 0.01336 | -5.846341463 | 34.17970851 |
| Glutathione S-transferase theta-2B || id:prot-a-1291 | rs5760104 | A | G | 22 | 24249573 | -0.1414 | 1.26E-08 | 0.0248 | 0.53088 | -5.701612903 | 32.5083897 |
| Glycerol-3-phosphate dehydrogenase 1-like protein || id:prot-a-1250 | rs7539005 | T | A | 1 | 196667252 | -0.2294 | 1.17E-19 | 0.0253 | 0.61028 | -9.067193676 | 82.21400116 |
| Glycerol-3-phosphate dehydrogenase 1-like protein || id:prot-a-1250 | rs74480769 | G | A | 5 | 40972211 | -0.538 | 1.95E-13 | 0.0732 | 0.0317 | -7.349726776 | 54.01848368 |
| Glycerol-3-phosphate dehydrogenase 1-like protein || id:prot-a-1250 | rs704 | A | G | 17 | 26694861 | 0.2653 | 7.59E-28 | 0.0243 | 0.46665 | 10.91769547 | 119.1960744 |
| Glycerophosphocholine phosphodiesterase GPCPD1 || id:prot-a-1249 | rs9993274 | A | T | 4 | 166727034 | 0.1456 | 1.15E-08 | 0.0255 | 0.57807 | 5.709803922 | 32.60186082 |
| Glycerophosphocholine phosphodiesterase GPCPD1 || id:prot-a-1249 | rs62143197 | A | G | 19 | 54320716 | 0.1928 | 1.95E-10 | 0.0303 | 0.22311 | 6.363036304 | 40.488231 |
| Glycine N-methyltransferase || id:prot-a-1229 | rs6458314 | A | C | 6 | 42922215 | -0.3794 | 4.17E-55 | 0.0243 | 0.59588 | -15.61316872 | 243.7710376 |
| Glycogen phosphorylase, liver form || id:prot-a-2462 | rs74511171 | T | C | 5 | 25576699 | 0.2798 | 7.94E-09 | 0.0485 | 0.07457 | 5.769072165 | 33.28219364 |
| Glycogen phosphorylase, liver form || id:prot-a-2462 | rs144359893 | T | C | 14 | 51388134 | -0.8006 | 9.77E-12 | 0.1176 | 0.01425 | -6.807823129 | 46.34645576 |
| Glycogen phosphorylase, liver form || id:prot-a-2462 | rs62143198 | A | G | 19 | 54320939 | 0.4367 | 1.15E-48 | 0.0298 | 0.21453 | 14.65436242 | 214.7503378 |
| Glycolipid transfer protein domain-containing protein 2 || id:prot-a-1223 | rs7232 | A | T | 11 | 59940599 | 0.1656 | 2.29E-10 | 0.0261 | 0.36896 | 6.344827586 | 40.2568371 |
| Glycolipid transfer protein domain-containing protein 2 || id:prot-a-1223 | rs34460487 | A | G | 17 | 4685228 | 0.2669 | 6.76E-25 | 0.0259 | 0.34637 | 10.30501931 | 106.1934229 |
| Glycolipid transfer protein domain-containing protein 2 || id:prot-a-1223 | rs34088054 | T | C | 19 | 13545331 | -0.309 | 2.63E-08 | 0.0555 | 0.06856 | -5.567567568 | 30.99780862 |
| Glycoprotein endo-alpha-1,2-mannosidase || id:prot-a-1837 | rs13205922 | A | T | 6 | 96023692 | -1.7002 | 1.00E-200 | 0.0345 | 0.08135 | -49.28115942 | 2428.632674 |
| Glycoprotein endo-alpha-1,2-mannosidase || id:prot-a-1837 | rs9321415 | G | A | 6 | 96212413 | -0.2054 | 6.31E-17 | 0.0246 | 0.45994 | -8.349593496 | 69.71571155 |
| Glycoprotein hormone alpha-2 || id:prot-a-1251 | rs4638719 | G | A | 19 | 15583562 | -0.4636 | 3.16E-82 | 0.0241 | 0.37252 | -19.23651452 | 370.043491 |
| Glycoprotein Xg || id:prot-a-3239 | rs66707192 | A | G | 3 | 186382065 | -0.168 | 2.82E-09 | 0.0283 | 0.24546 | -5.93639576 | 35.24079462 |
| Glycoprotein Xg || id:prot-a-3239 | rs4241818 | C | T | 4 | 187153786 | -0.1922 | 5.37E-15 | 0.0246 | 0.51359 | -7.81300813 | 61.04309604 |
| Glycosaminoglycan xylosylkinase || id:prot-a-1046 | rs12064702 | C | T | 1 | 179008935 | 0.1505 | 2.04E-09 | 0.0251 | 0.41322 | 5.996015936 | 35.95220711 |
| Glycosaminoglycan xylosylkinase || id:prot-a-1046 | rs532436 | A | G | 9 | 136149830 | 0.2632 | 1.26E-16 | 0.0318 | 0.17992 | 8.27672956 | 68.50425221 |
| Glycosaminoglycan xylosylkinase || id:prot-a-1046 | rs62143198 | A | G | 19 | 54320939 | -0.1673 | 4.47E-08 | 0.0306 | 0.21453 | -5.467320261 | 29.89159084 |
| Glycosyltransferase 8 domain-containing protein 1 || id:prot-a-1221 | rs7539005 | T | A | 1 | 196667252 | -0.6913 | 1.00E-200 | 0.0226 | 0.61028 | -30.58849558 | 935.6560616 |
| Glycylpeptide N-tetradecanoyltransferase 1 || id:prot-a-2063 | rs10418046 | G | T | 19 | 54327869 | 0.2701 | 7.41E-20 | 0.0296 | 0.21727 | 9.125 | 83.265625 |
| Glypican-1 || id:prot-a-1245 | rs4074478 | T | C | 2 | 241446340 | 0.4237 | 4.27E-25 | 0.0409 | 0.10644 | 10.3594132 | 107.3174419 |
| Glypican-1 || id:prot-a-1245 | rs704 | A | G | 17 | 26694861 | 0.1523 | 5.62E-10 | 0.0246 | 0.46665 | 6.191056911 | 38.32918567 |
| Glypican-3 || id:prot-a-1246 | rs11751347 | T | C | 6 | 161092438 | -0.2479 | 1.74E-09 | 0.0412 | 0.10061 | -6.016990291 | 36.20417217 |
| Glypican-3 || id:prot-a-1246 | rs313786 | T | C | 19 | 3283789 | -0.1712 | 1.07E-09 | 0.0281 | 0.26362 | -6.09252669 | 37.11888147 |
| Glypican-5 || id:prot-a-1247 | rs342702 | T | G | 13 | 92422946 | -0.8042 | 1.00E-200 | 0.0247 | 0.27402 | -32.55870445 | 1060.069236 |
| Glypican-5 || id:prot-a-1247 | rs7330545 | G | A | 13 | 92047450 | -0.2825 | 1.07E-18 | 0.032 | 0.81995 | -8.828125 | 77.93579102 |
| Glypican-6 || id:prot-a-1248 | rs28929474 | T | C | 14 | 94844947 | 0.6215 | 8.71E-14 | 0.0833 | 0.02269 | 7.460984394 | 55.66628812 |
| Golgi membrane protein 1 || id:prot-a-1238 | rs550057 | T | C | 9 | 136146597 | 0.5503 | 3.55E-92 | 0.027 | 0.24577 | 20.38148148 | 415.4047874 |
| Golgi membrane protein 1 || id:prot-a-1238 | rs601338 | A | G | 19 | 49206674 | -0.268 | 1.12E-28 | 0.0241 | 0.50711 | -11.12033195 | 123.6617827 |
| Golgi SNAP receptor complex member 1 || id:prot-a-1240 | rs11916620 | C | T | 3 | 34553323 | -0.1591 | 2.45E-08 | 0.0285 | 0.2796 | -5.58245614 | 31.16381656 |
| Golgi SNAP receptor complex member 1 || id:prot-a-1240 | rs704 | A | G | 17 | 26694861 | 0.2599 | 9.55E-27 | 0.0243 | 0.46665 | 10.69547325 | 114.3931481 |
| GRAM domain-containing protein 1C || id:prot-a-1266 | rs61077924 | G | C | 3 | 113625933 | 0.3684 | 1.48E-47 | 0.0254 | 0.32402 | 14.50393701 | 210.3641887 |
| GRAM domain-containing protein 1C || id:prot-a-1266 | rs704 | A | G | 17 | 26694861 | -0.2694 | 1.12E-28 | 0.0242 | 0.46665 | -11.1322314 | 123.9265761 |
| GRAM domain-containing protein 1C || id:prot-a-1267 | rs61077924 | G | C | 3 | 113625933 | 0.6198 | 3.39E-148 | 0.0239 | 0.32402 | 25.93305439 | 672.5233102 |
| GRAM domain-containing protein 1C || id:prot-a-1267 | rs704 | A | G | 17 | 26694861 | -0.2099 | 8.32E-18 | 0.0244 | 0.46665 | -8.602459016 | 74.00230113 |
| Grancalcin || id:prot-a-1180 | rs34436714 | A | C | 19 | 54327313 | 0.5292 | 1.35E-75 | 0.0288 | 0.21294 | 18.375 | 337.640625 |
| Granulins || id:prot-a-1277 | rs646776 | T | C | 1 | 109818530 | 0.8254 | 1.00E-200 | 0.0266 | 0.77654 | 31.03007519 | 962.8655662 |
| Granulins || id:prot-a-1277 | rs4747199 | T | C | 10 | 73569318 | -0.2365 | 4.27E-18 | 0.0273 | 0.29406 | -8.663003663 | 75.04763247 |
| Granulins || id:prot-a-1277 | rs8056814 | A | G | 16 | 75252327 | -0.2763 | 1.70E-10 | 0.0433 | 0.08989 | -6.381062356 | 40.71795679 |
| Granulins || id:prot-a-1277 | rs5848 | T | C | 17 | 42430244 | -0.2763 | 8.13E-23 | 0.0281 | 0.28735 | -9.832740214 | 96.68278011 |
| Granulocyte-macrophage colony-stimulating factor || id:prot-a-681 | rs114137635 | A | G | 3 | 1915930 | 0.4059 | 1.45E-08 | 0.0716 | 0.03371 | 5.668994413 | 32.13749766 |
| Granulocyte colony-stimulating factor receptor || id:prot-a-686 | rs4632248 | T | G | 19 | 54324995 | -0.1635 | 4.68E-08 | 0.0299 | 0.21439 | -5.468227425 | 29.90151117 |
| Granulysin || id:prot-a-1228 | rs78809601 | T | A | 2 | 85788476 | 0.3794 | 3.98E-08 | 0.0691 | 0.04159 | 5.490593343 | 30.14661526 |
| Granulysin || id:prot-a-1228 | rs12151621 | A | C | 2 | 85934499 | 0.7736 | 7.41E-189 | 0.0264 | 0.22704 | 29.3030303 | 858.6675849 |
| Granulysin || id:prot-a-1228 | rs12714148 | A | T | 2 | 85859835 | -0.1678 | 3.16E-09 | 0.0283 | 0.28591 | -5.929328622 | 35.15693791 |
| Granulysin || id:prot-a-1228 | rs201376697 | G | GACA | 6 | 31066627 | -0.2133 | 5.01E-10 | 0.0343 | 0.15564 | -6.218658892 | 38.67171842 |
| Granulysin || id:prot-a-1228 | rs7742179 | A | T | 6 | 31159020 | 0.1771 | 2.04E-12 | 0.0252 | 0.36458 | 7.027777778 | 49.38966049 |
| Granzyme A || id:prot-a-1297 | rs1049281 | C | T | 6 | 31236567 | 0.2993 | 3.39E-32 | 0.0253 | 0.64831 | 11.83003953 | 139.9498352 |
| Granzyme A || id:prot-a-1297 | rs28359800 | CAA | C | 6 | 31267774 | -0.1602 | 3.16E-09 | 0.0271 | 0.70451 | -5.911439114 | 34.9451124 |
| Granzyme A || id:prot-a-1297 | rs62454712 | C | T | 7 | 6498057 | -0.1784 | 4.37E-10 | 0.0286 | 0.24551 | -6.237762238 | 38.90967773 |
| Granzyme B || id:prot-a-1298 | rs115959678 | C | T | 6 | 31326080 | 0.3398 | 2.95E-10 | 0.0539 | 0.05691 | 6.304267161 | 39.74378444 |
| Granzyme B || id:prot-a-1298 | rs8192917 | T | C | 14 | 25102160 | 1.005 | 1.00E-200 | 0.025 | 0.78665 | 40.2 | 1616.04 |
| Granzyme B || id:prot-a-1298 | rs9743931 | C | G | 14 | 24965393 | 0.161 | 1.12E-08 | 0.0282 | 0.26469 | 5.709219858 | 32.59519139 |
| Granzyme B || id:prot-a-1298 | rs9916629 | C | T | 17 | 33796260 | -0.1658 | 4.37E-10 | 0.0266 | 0.33132 | -6.233082707 | 38.85132003 |
| Granzyme B || id:prot-a-1299 | rs201573837 | T | TA | 6 | 51408639 | -0.6428 | 4.37E-08 | 0.1174 | 0.98738 | -5.475298126 | 29.97888957 |
| Granzyme B || id:prot-a-1299 | rs9743931 | C | G | 14 | 24965393 | -0.1721 | 1.00E-09 | 0.0282 | 0.26469 | -6.102836879 | 37.24461798 |
| Granzyme B || id:prot-a-1299 | rs8192917 | T | C | 14 | 25102160 | -0.6688 | 7.24E-124 | 0.0283 | 0.78665 | -23.63250883 | 558.4954738 |
| Granzyme H || id:prot-a-1300 | rs34436714 | A | C | 19 | 54327313 | -0.1727 | 9.12E-09 | 0.0301 | 0.21294 | -5.737541528 | 32.91938279 |
| Granzyme K || id:prot-a-1301 | rs6891823 | T | C | 5 | 54317900 | 0.1765 | 2.19E-12 | 0.0251 | 0.56259 | 7.03187251 | 49.447231 |
| Granzyme K || id:prot-a-1301 | rs9266257 | C | G | 6 | 31325886 | 0.1746 | 5.25E-11 | 0.0266 | 0.6812 | 6.563909774 | 43.08491153 |
| Granzyme M || id:prot-a-1302 | rs16989724 | T | C | 19 | 531115 | 0.4303 | 3.89E-16 | 0.0529 | 0.93513 | 8.134215501 | 66.16546182 |
| Gremlin-1 || id:prot-a-1273 | rs12614 | T | C | 6 | 31914179 | 0.4406 | 6.92E-25 | 0.0428 | 0.08711 | 10.29439252 | 105.9745174 |
| Gremlin-2 || id:prot-a-1274 | rs149062332 | C | T | 3 | 186341025 | 0.8593 | 1.05E-13 | 0.1156 | 0.01216 | 7.433391003 | 55.25530181 |
| Gro-beta/gamma || id:prot-a-746 | rs9271732 | A | G | 6 | 32593627 | -0.1962 | 5.89E-11 | 0.03 | 0.78454 | -6.54 | 42.7716 |
| Group 10 secretory phospholipase A2 || id:prot-a-2283 | rs62143196 | G | A | 19 | 54320636 | -0.1778 | 4.17E-09 | 0.0303 | 0.22404 | -5.867986799 | 34.43326907 |
| Group IIE secretory phospholipase A2 || id:prot-a-2289 | rs4632248 | T | G | 19 | 54324995 | -0.1871 | 3.89E-10 | 0.0299 | 0.21439 | -6.257525084 | 39.15662017 |
| Group XIIB secretory phospholipase A2-like protein || id:prot-a-2284 | rs4970836 | A | G | 1 | 109821797 | 0.4224 | 3.80E-48 | 0.029 | 0.77057 | 14.56551724 | 212.1542925 |
| Group XIIB secretory phospholipase A2-like protein || id:prot-a-2284 | rs662 | C | T | 7 | 94937446 | -0.1546 | 1.74E-08 | 0.0274 | 0.2829 | -5.642335766 | 31.8359529 |
| Growth-regulated alpha protein || id:prot-a-742 | rs77689210 | G | C | 4 | 74391568 | 0.7115 | 2.57E-11 | 0.1067 | 0.01344 | 6.668228679 | 44.46527371 |
| Growth-regulated alpha protein || id:prot-a-742 | rs2115691 | G | C | 4 | 74743691 | 0.7462 | 2.04E-178 | 0.0262 | 0.23997 | 28.48091603 | 811.1625779 |
| Growth arrest and DNA damage-inducible proteins-interacting protein 1 || id:prot-a-1165 | rs33944729 | T | TA | 1 | 196652124 | 0.4467 | 1.02E-75 | 0.0243 | 0.38701 | 18.38271605 | 337.9242494 |
| Growth factor receptor-bound protein 14 || id:prot-a-1270 | rs704 | A | G | 17 | 26694861 | 0.2309 | 2.45E-21 | 0.0243 | 0.46665 | 9.502057613 | 90.28909888 |
| Growth factor receptor-bound protein 2 || id:prot-a-1271 | rs62143197 | A | G | 19 | 54320716 | 0.4241 | 1.17E-46 | 0.0296 | 0.22311 | 14.3277027 | 205.2830647 |
| Growth factor receptor-bound protein 7 || id:prot-a-1272 | rs61772167 | T | C | 1 | 7450769 | 0.5078 | 2.24E-08 | 0.0908 | 0.02167 | 5.592511013 | 31.27617943 |
| Growth factor receptor-bound protein 7 || id:prot-a-1272 | rs74480769 | G | A | 5 | 40972211 | -0.4063 | 3.16E-08 | 0.0734 | 0.0317 | -5.535422343 | 30.64090052 |
| Growth factor receptor-bound protein 7 || id:prot-a-1272 | rs704 | A | G | 17 | 26694861 | 0.2106 | 6.61E-18 | 0.0244 | 0.46665 | 8.631147541 | 74.49670787 |
| Growth factor receptor-bound protein 7 || id:prot-a-1272 | rs1065853 | T | G | 19 | 45413233 | 0.2639 | 1.17E-08 | 0.0463 | 0.07778 | 5.699784017 | 32.48753784 |
| Growth hormone receptor || id:prot-a-1211 | rs4866942 | C | T | 5 | 42641962 | -0.1572 | 2.63E-10 | 0.0249 | 0.54384 | -6.313253012 | 39.85716359 |
| Growth/differentiation factor 11 || id:prot-a-1193 | rs10780841 | G | A | 9 | 90094636 | 0.1394 | 4.47E-08 | 0.0255 | 0.39048 | 5.466666667 | 29.88444444 |
| Growth/differentiation factor 11/8 || id:prot-a-1194 | rs4576609 | T | C | 1 | 2938265 | 0.1761 | 4.68E-08 | 0.0322 | 0.1836 | 5.468944099 | 29.90934956 |
| Growth/differentiation factor 11/8 || id:prot-a-1194 | rs1815739 | C | T | 11 | 66328095 | 0.1684 | 8.13E-12 | 0.0246 | 0.55602 | 6.845528455 | 46.86125983 |
| Growth/differentiation factor 11/8 || id:prot-a-1194 | rs57906300 | G | C | 12 | 94988008 | 0.1672 | 3.09E-08 | 0.0302 | 0.27127 | 5.536423841 | 30.65198895 |
| Growth/differentiation factor 11/8 || id:prot-a-1194 | rs11079936 | C | T | 17 | 48907834 | -0.1784 | 7.94E-12 | 0.0261 | 0.66932 | -6.835249042 | 46.72062947 |
| Growth/differentiation factor 15 || id:prot-a-1195 | rs45543339 | T | C | 19 | 18503194 | 0.5755 | 1.45E-99 | 0.0272 | 0.25864 | 21.15808824 | 447.6646978 |
| Growth/differentiation factor 15 || id:prot-a-1195 | rs138185133 | C | T | 19 | 18450140 | 0.4537 | 3.89E-08 | 0.0826 | 0.0269 | 5.492736077 | 30.17014962 |
| Growth/differentiation factor 5 || id:prot-a-1196 | rs117701985 | A | G | 9 | 7740128 | 0.5887 | 2.63E-08 | 0.1058 | 0.01519 | 5.564272212 | 30.96112525 |
| Growth/differentiation factor 5 || id:prot-a-1196 | rs704 | A | G | 17 | 26694861 | 0.8408 | 1.00E-200 | 0.0199 | 0.46665 | 42.25125628 | 1785.168657 |
| Growth/differentiation factor 8 || id:prot-a-1957 | rs3837428 | GTTA | G | 11 | 66328494 | 0.16 | 7.59E-11 | 0.0246 | 0.55053 | 6.504065041 | 42.30286205 |
| GSK3-beta interaction protein || id:prot-a-1282 | rs146071404 | T | C | 1 | 36998494 | 0.4171 | 2.34E-08 | 0.0747 | 0.02861 | 5.583668005 | 31.17734839 |
| GTP-binding protein GEM || id:prot-a-1199 | rs704 | A | G | 17 | 26694861 | 0.9061 | 1.00E-200 | 0.019 | 0.46665 | 47.68947368 | 2274.2859 |
| GTP-binding protein GEM || id:prot-a-1199 | rs117032462 | G | A | 17 | 26833853 | -0.4123 | 2.00E-09 | 0.0687 | 0.03301 | -6.001455604 | 36.01746937 |
| GTP-binding protein GEM || id:prot-a-1199 | rs1065853 | T | G | 19 | 45413233 | 0.276 | 2.40E-09 | 0.0463 | 0.07778 | 5.96112311 | 35.53498873 |
| GTPase KRas || id:prot-a-1679 | rs34222529 | C | T | 11 | 6447776 | 0.1617 | 3.31E-08 | 0.0293 | 0.24309 | 5.518771331 | 30.456837 |
| Guanine deaminase || id:prot-a-1189 | rs13402561 | G | C | 2 | 3639921 | -0.2199 | 2.14E-11 | 0.0328 | 0.81414 | -6.704268293 | 44.94721334 |
| Guanine nucleotide-binding protein G(k) subunit alpha || id:prot-a-1226 | rs62143198 | A | G | 19 | 54320939 | 0.2275 | 8.51E-14 | 0.0305 | 0.21453 | 7.459016393 | 55.63692556 |
| Guanine nucleotide-binding protein G(k) subunit alpha || id:prot-a-1226 | rs7412 | T | C | 19 | 45412079 | 0.8421 | 3.63E-81 | 0.0441 | 0.07776 | 19.0952381 | 364.6281179 |
| Guanylate-binding protein 6 || id:prot-a-1178 | rs570618 | G | T | 1 | 196657064 | -0.1475 | 5.89E-09 | 0.0254 | 0.61149 | -5.807086614 | 33.72225494 |
| Guanylate-binding protein 6 || id:prot-a-1178 | rs74480769 | G | A | 5 | 40972211 | -0.5087 | 3.72E-12 | 0.0732 | 0.0317 | -6.949453552 | 48.29490467 |
| Guanylate-binding protein 6 || id:prot-a-1178 | rs704 | A | G | 17 | 26694861 | 0.2586 | 1.78E-26 | 0.0243 | 0.46665 | 10.64197531 | 113.2516385 |
| Guanylate cyclase activator 2B || id:prot-a-1294 | rs3818727 | A | G | 1 | 42621645 | 0.2435 | 5.25E-10 | 0.0392 | 0.11442 | 6.211734694 | 38.58564791 |
| Guanylyl cyclase-activating protein 1 || id:prot-a-1293 | rs892145 | T | A | 19 | 15586672 | -0.3875 | 1.86E-55 | 0.0247 | 0.3719 | -15.68825911 | 246.1214739 |
| Haloacid dehalogenase-like hydrolase domain-containing protein 2 || id:prot-a-1322 | rs75228657 | G | A | 18 | 44741063 | 0.7021 | 4.68E-38 | 0.0544 | 0.05226 | 12.90625 | 166.5712891 |
| Haloacid dehalogenase-like hydrolase domain-containing protein 2 || id:prot-a-1322 | rs62143198 | A | G | 19 | 54320939 | 0.3973 | 3.72E-40 | 0.03 | 0.21453 | 13.24333333 | 175.3858778 |
| Haptoglobin || id:prot-a-1369 | rs150114847 | G | A | 12 | 127556150 | 0.4932 | 3.47E-08 | 0.0894 | 0.02593 | 5.516778523 | 30.43484528 |
| Haptoglobin || id:prot-a-1369 | rs7202724 | G | A | 16 | 72030439 | -0.3785 | 4.17E-43 | 0.0275 | 0.32574 | -13.76363636 | 189.437686 |
| Haptoglobin || id:prot-a-1369 | rs17321931 | A | G | 16 | 70998557 | 0.2586 | 4.07E-08 | 0.0471 | 0.0735 | 5.49044586 | 30.14499574 |
| Haptoglobin || id:prot-a-1369 | rs217184 | C | T | 16 | 72105965 | 0.8688 | 1.00E-200 | 0.0277 | 0.19565 | 31.36462094 | 983.7394466 |
| Haptoglobin || id:prot-a-1369 | rs117155836 | A | G | 16 | 72818627 | 0.6169 | 4.07E-17 | 0.0734 | 0.02853 | 8.404632153 | 70.63784162 |
| Heat shock 70 kDa protein 1-like || id:prot-a-1387 | rs9881048 | C | A | 3 | 165506252 | -0.1921 | 1.86E-09 | 0.032 | 0.8162 | -6.003125 | 36.03750977 |
| Heat shock 70 kDa protein 1-like || id:prot-a-1387 | rs704 | A | G | 17 | 26694861 | 0.3712 | 1.12E-54 | 0.0238 | 0.46665 | 15.59663866 | 243.2551373 |
| Heat shock 70 kDa protein 6 || id:prot-a-1388 | rs704 | A | G | 17 | 26694861 | 0.187 | 2.19E-14 | 0.0245 | 0.46665 | 7.632653061 | 58.25739275 |
| Hematopoietic progenitor cell antigen CD34 || id:prot-a-440 | rs704 | A | G | 17 | 26694861 | 0.2517 | 3.80E-25 | 0.0243 | 0.46665 | 10.35802469 | 107.2886755 |
| Hematopoietic progenitor cell antigen CD34 || id:prot-a-441 | rs201356756 | A | AT | 3 | 159108093 | 0.3971 | 7.08E-10 | 0.0644 | 0.95919 | 6.166149068 | 38.02139433 |
| Hematopoietic progenitor cell antigen CD34 || id:prot-a-441 | rs2516568 | T | A | 9 | 95187380 | -0.4346 | 2.24E-66 | 0.0253 | 0.32324 | -17.17786561 | 295.079067 |
| Hematopoietic prostaglandin D synthase || id:prot-a-1371 | rs1965049 | G | A | 4 | 95266204 | 0.4396 | 1.35E-71 | 0.0246 | 0.62096 | 17.8699187 | 319.3339943 |
| Heme oxygenase 2 || id:prot-a-1358 | rs10424405 | G | A | 19 | 54321933 | 0.3037 | 3.02E-24 | 0.0299 | 0.2203 | 10.15719064 | 103.1685216 |
| HemK methyltransferase family member 2 || id:prot-a-1989 | rs62143197 | A | G | 19 | 54320716 | 0.3527 | 3.24E-32 | 0.0299 | 0.22311 | 11.79598662 | 139.1453004 |
| Hemoglobin subunit gamma-1 || id:prot-a-1315 | rs967645 | T | C | 17 | 26713970 | 0.1623 | 2.75E-11 | 0.0244 | 0.50945 | 6.651639344 | 44.24430597 |
| Hemoglobin subunit theta-1 || id:prot-a-1316 | rs1065853 | T | G | 19 | 45413233 | -0.9161 | 1.15E-97 | 0.0437 | 0.07778 | -20.96338673 | 439.4635831 |
| Hemoglobin subunit theta-1 || id:prot-a-1316 | rs9304645 | A | G | 19 | 45450328 | -0.1814 | 2.82E-08 | 0.0327 | 0.18091 | -5.547400612 | 30.77365355 |
| Hemoglobin subunit zeta || id:prot-a-1317 | rs1427407 | G | T | 2 | 60718043 | -0.1843 | 2.24E-08 | 0.033 | 0.83433 | -5.584848485 | 31.1905326 |
| Hemoglobin subunit zeta || id:prot-a-1317 | rs2461286 | G | A | 16 | 203254 | -0.8846 | 1.00E-200 | 0.0209 | 0.64686 | -42.32535885 | 1791.436002 |
| Hemoglobin subunit zeta || id:prot-a-1317 | rs12934930 | G | A | 16 | 407554 | 0.2747 | 2.14E-16 | 0.0334 | 0.15828 | 8.224550898 | 67.64323748 |
| Hemojuvelin || id:prot-a-1332 | rs75466623 | A | G | 1 | 145414458 | -0.6048 | 4.90E-09 | 0.1034 | 0.01599 | -5.849129594 | 34.21231701 |
| Hemojuvelin || id:prot-a-1332 | rs12740374 | T | G | 1 | 109817590 | -0.2313 | 1.41E-14 | 0.0301 | 0.22252 | -7.684385382 | 59.0497787 |
| Hemojuvelin || id:prot-a-1332 | rs10972581 | T | C | 9 | 35779571 | -0.1497 | 2.34E-09 | 0.0251 | 0.40071 | -5.964143426 | 35.57100681 |
| Hemojuvelin || id:prot-a-1332 | rs4778090 | G | A | 15 | 93609617 | -0.1475 | 5.89E-09 | 0.0253 | 0.5557 | -5.830039526 | 33.98936087 |
| HEPACAM family member 2 || id:prot-a-1326 | rs704 | A | G | 17 | 26694861 | 0.157 | 1.62E-10 | 0.0245 | 0.46665 | 6.408163265 | 41.06455643 |
| HEPACAM family member 2 || id:prot-a-1327 | rs259446 | C | T | 3 | 22074288 | -0.1588 | 4.27E-09 | 0.027 | 0.71837 | -5.881481481 | 34.59182442 |
| HEPACAM family member 2 || id:prot-a-1327 | rs6998727 | G | C | 8 | 1660453 | -0.4381 | 3.80E-08 | 0.0796 | 0.03099 | -5.503768844 | 30.29147149 |
| Heparan-sulfate 6-O-sulfotransferase 1 || id:prot-a-1378 | rs34827544 | T | C | 2 | 129084425 | -0.3048 | 1.91E-18 | 0.0348 | 0.15729 | -8.75862069 | 76.71343639 |
| Heparan-sulfate 6-O-sulfotransferase 1 || id:prot-a-1378 | rs34748727 | T | TTG | 5 | 138770437 | 0.1481 | 2.19E-08 | 0.0265 | 0.34905 | 5.588679245 | 31.23333571 |
| Heparan-sulfate 6-O-sulfotransferase 1 || id:prot-a-1378 | rs704 | A | G | 17 | 26694861 | -0.1901 | 7.94E-15 | 0.0245 | 0.46665 | -7.759183673 | 60.20493128 |
| Heparan sulfate glucosamine 3-O-sulfotransferase 3A1 || id:prot-a-1375 | rs10418046 | G | T | 19 | 54327869 | 0.3213 | 1.05E-27 | 0.0295 | 0.21727 | 10.89152542 | 118.6253261 |
| Heparan sulfate glucosamine 3-O-sulfotransferase 3B1 || id:prot-a-1376 | rs12475878 | T | G | 2 | 230070049 | -0.164 | 2.04E-08 | 0.0292 | 0.25402 | -5.616438356 | 31.54437981 |
| Heparin-binding EGF-like growth factor || id:prot-a-1313 | rs10820606 | C | A | 9 | 99192919 | -0.1896 | 9.55E-11 | 0.0293 | 0.24318 | -6.470989761 | 41.87370849 |
| Heparin-binding EGF-like growth factor || id:prot-a-1313 | rs7080386 | A | C | 10 | 65048306 | 0.1858 | 1.62E-13 | 0.0252 | 0.41587 | 7.373015873 | 54.36136306 |
| Heparin-binding EGF-like growth factor || id:prot-a-1313 | rs11553699 | G | A | 12 | 122216910 | 0.262 | 3.47E-12 | 0.0377 | 0.14549 | 6.949602122 | 48.29696965 |
| Hepatitis A virus cellular receptor 1 || id:prot-a-1310 | rs67114357 | C | T | 5 | 156484480 | -0.153 | 4.07E-08 | 0.0279 | 0.28035 | -5.483870968 | 30.07284079 |
| Hepatitis A virus cellular receptor 1 || id:prot-a-1310 | rs4704729 | T | G | 5 | 156424516 | -0.4323 | 5.89E-59 | 0.0267 | 0.28026 | -16.19101124 | 262.1488448 |
| Hepatitis A virus cellular receptor 2 || id:prot-a-1311 | rs6874178 | T | A | 5 | 156530149 | -0.734 | 1.05E-145 | 0.0286 | 0.81718 | -25.66433566 | 658.6581251 |
| Hepatitis A virus cellular receptor 2 || id:prot-a-1312 | rs62032164 | C | T | 16 | 16343687 | -0.5931 | 6.17E-90 | 0.0295 | 0.22458 | -20.10508475 | 404.2144326 |
| Hepatitis A virus cellular receptor 2 || id:prot-a-1312 | rs117059302 | G | A | 16 | 18768776 | 0.8549 | 4.79E-18 | 0.0987 | 0.0172 | 8.661600811 | 75.0233286 |
| Hepatocyte growth factor || id:prot-a-1334 | rs5745695 | A | G | 7 | 81358075 | 0.1942 | 1.17E-11 | 0.0286 | 0.76041 | 6.79020979 | 46.106949 |
| Hepatocyte growth factor receptor || id:prot-a-1882 | rs1858830 | G | C | 7 | 116312439 | -0.1646 | 2.00E-10 | 0.0259 | 0.45046 | -6.355212355 | 40.38872408 |
| Hepatocyte growth factor receptor || id:prot-a-1882 | rs6955032 | A | T | 7 | 144369376 | -0.2206 | 3.16E-09 | 0.0372 | 0.12327 | -5.930107527 | 35.16617528 |
| Hepatocyte growth factor receptor || id:prot-a-1882 | rs635634 | T | C | 9 | 136155000 | -0.38 | 3.24E-33 | 0.0316 | 0.17981 | -12.02531646 | 144.6082359 |
| Hepatocyte nuclear factor 4-alpha || id:prot-a-1360 | rs28383251 | T | C | 6 | 32585567 | 0.2402 | 1.05E-12 | 0.0337 | 0.15851 | 7.127596439 | 50.802631 |
| Hepatocyte nuclear factor 4-alpha || id:prot-a-1360 | rs116254005 | A | G | 6 | 47297915 | 0.609 | 1.66E-08 | 0.1079 | 0.01472 | 5.644114921 | 31.85603324 |
| Hepatocyte nuclear factor 4-alpha || id:prot-a-1360 | rs34363414 | T | C | 6 | 32545397 | 0.2495 | 4.90E-12 | 0.0361 | 0.14968 | 6.911357341 | 47.76686029 |
| Hepatocyte nuclear factor 4-alpha || id:prot-a-1360 | rs144515162 | G | A | 6 | 32455083 | 0.3113 | 3.02E-13 | 0.0427 | 0.13145 | 7.290398126 | 53.14990484 |
| Hepatocyte nuclear factor 4-alpha || id:prot-a-1360 | rs141738059 | C | T | 9 | 116832007 | -1.0274 | 1.45E-20 | 0.1105 | 0.01278 | -9.297737557 | 86.44792367 |
| Heterogeneous nuclear ribonucleoprotein A/B || id:prot-a-1362 | rs62143206 | T | G | 19 | 54326212 | 0.6349 | 4.37E-114 | 0.028 | 0.2131 | 22.675 | 514.155625 |
| Heterogeneous nuclear ribonucleoprotein A/B || id:prot-a-1363 | rs62143197 | A | G | 19 | 54320716 | 0.5665 | 6.46E-86 | 0.0288 | 0.22311 | 19.67013889 | 386.9143639 |
| Heterogeneous nuclear ribonucleoprotein D-like || id:prot-a-1365 | rs62143198 | A | G | 19 | 54320939 | 0.6982 | 5.50E-135 | 0.0282 | 0.21453 | 24.75886525 | 613.0014084 |
| Heterogeneous nuclear ribonucleoprotein M || id:prot-a-1367 | rs13276307 | T | C | 8 | 144646352 | 0.1588 | 2.63E-08 | 0.0285 | 0.25219 | 5.571929825 | 31.04640197 |
| Heterogeneous nuclear ribonucleoprotein M || id:prot-a-1367 | rs62143206 | T | G | 19 | 54326212 | 0.5987 | 5.50E-100 | 0.0282 | 0.2131 | 21.23049645 | 450.7339797 |
| Heterogeneous nuclear ribonucleoprotein Q || id:prot-a-2909 | rs74480769 | G | A | 5 | 40972211 | -0.5113 | 2.88E-12 | 0.0732 | 0.0317 | -6.984972678 | 48.78984331 |
| Heterogeneous nuclear ribonucleoprotein Q || id:prot-a-2909 | rs10418046 | G | T | 19 | 54327869 | 0.2738 | 2.24E-20 | 0.0296 | 0.21727 | 9.25 | 85.5625 |
| Heterogeneous nuclear ribonucleoproteins A2/B1 || id:prot-a-1361 | rs62143206 | T | G | 19 | 54326212 | 0.6828 | 7.08E-135 | 0.0276 | 0.2131 | 24.73913043 | 612.0245747 |
| Heterogeneous nuclear ribonucleoproteins C1/C2 || id:prot-a-1364 | rs967645 | T | C | 17 | 26713970 | 0.1703 | 2.75E-12 | 0.0244 | 0.50945 | 6.979508197 | 48.71353467 |
| Heterogeneous nuclear ribonucleoproteins C1/C2 || id:prot-a-1364 | rs2232613 | T | C | 20 | 36997655 | -0.5213 | 1.35E-30 | 0.0453 | 0.0768 | -11.50772627 | 132.4277639 |
| Hexokinase-1 || id:prot-a-1343 | rs7539005 | T | A | 1 | 196667252 | -0.1684 | 3.55E-11 | 0.0254 | 0.61028 | -6.62992126 | 43.95585591 |
| Hexokinase-1 || id:prot-a-1343 | rs74480769 | G | A | 5 | 40972211 | -0.6409 | 1.48E-18 | 0.0729 | 0.0317 | -8.791495199 | 77.29038783 |
| Hexokinase-2 || id:prot-a-1344 | rs13276307 | T | C | 8 | 144646352 | 0.164 | 9.12E-09 | 0.0285 | 0.25219 | 5.754385965 | 33.11295783 |
| Hexokinase-2 || id:prot-a-1344 | rs62143206 | T | G | 19 | 54326212 | 0.5717 | 2.75E-90 | 0.0284 | 0.2131 | 20.13028169 | 405.2282409 |
| High affinity cAMP-specific 3',5'-cyclic phosphodiesterase 7A || id:prot-a-2223 | rs2375895 | G | C | 1 | 102674867 | -0.193 | 3.09E-10 | 0.0307 | 0.2044 | -6.286644951 | 39.52190474 |
| High affinity nerve growth factor receptor || id:prot-a-2121 | rs13402475 | G | C | 2 | 3639909 | -0.198 | 9.12E-10 | 0.0323 | 0.80838 | -6.13003096 | 37.57727957 |
| High mobility group protein B1 || id:prot-a-1351 | rs62143206 | T | G | 19 | 54326212 | 0.6771 | 2.40E-132 | 0.0277 | 0.2131 | 24.44404332 | 597.5112539 |
| High mobility group protein B2 || id:prot-a-1352 | rs62143198 | A | G | 19 | 54320939 | 0.5855 | 1.17E-90 | 0.029 | 0.21453 | 20.18965517 | 407.622176 |
| High mobility group protein B3 || id:prot-a-1353 | rs4632248 | T | G | 19 | 54324995 | 0.6974 | 8.91E-142 | 0.0275 | 0.21439 | 25.36 | 643.1296 |
| Histatin-1 || id:prot-a-1391 | rs3873746 | A | G | 7 | 99935097 | 0.3618 | 6.31E-32 | 0.0308 | 0.19285 | 11.74675325 | 137.9862118 |
| Histidine triad nucleotide-binding protein 1 || id:prot-a-1338 | rs202135714 | CT | C | 5 | 130501795 | 0.2463 | 1.58E-16 | 0.0299 | 0.22467 | 8.237458194 | 67.8557175 |
| Histidine triad nucleotide-binding protein 1 || id:prot-a-1338 | rs56106813 | T | C | 7 | 103451593 | -0.2881 | 1.70E-08 | 0.0511 | 0.0674 | -5.637964775 | 31.7866468 |
| Histo-blood group ABO system transferase || id:prot-a-10 | rs17150482 | C | T | 9 | 136194595 | -0.2787 | 4.37E-25 | 0.0269 | 0.28985 | -10.3605948 | 107.3419245 |
| Histo-blood group ABO system transferase || id:prot-a-10 | rs192129994 | A | G | 9 | 135956068 | 0.6286 | 2.14E-11 | 0.0939 | 0.02093 | 6.694355698 | 44.81439821 |
| Histo-blood group ABO system transferase || id:prot-a-10 | rs687289 | A | G | 9 | 136137106 | 1.296 | 1.00E-200 | 0.0141 | 0.31427 | 91.91489362 | 8448.347669 |
| Histone-lysine N-methyltransferase 2C || id:prot-a-1676 | rs183549995 | A | G | 4 | 6051294 | -0.5727 | 1.86E-08 | 0.1018 | 0.01548 | -5.625736739 | 31.64891385 |
| Histone-lysine N-methyltransferase 2C || id:prot-a-1676 | rs7412 | T | C | 19 | 45412079 | 0.5915 | 6.92E-39 | 0.0453 | 0.07776 | 13.05739514 | 170.4955679 |
| Histone-lysine N-methyltransferase 2D || id:prot-a-1905 | rs528298 | T | A | 1 | 196660995 | 0.7623 | 1.00E-200 | 0.0218 | 0.61415 | 34.96788991 | 1222.753325 |
| Histone-lysine N-methyltransferase EHMT2 || id:prot-a-914 | rs17713196 | T | C | 3 | 165489724 | 0.2248 | 3.47E-13 | 0.0309 | 0.20276 | 7.275080906 | 52.92680219 |
| Histone-lysine N-methyltransferase EHMT2 || id:prot-a-914 | rs35984397 | A | G | 4 | 187161501 | 0.1563 | 2.88E-10 | 0.0248 | 0.5138 | 6.302419355 | 39.72048972 |
| Histone-lysine N-methyltransferase SETD2 || id:prot-a-2703 | rs2719370 | A | G | 8 | 55274324 | -0.1891 | 4.90E-08 | 0.0347 | 0.16491 | -5.449567723 | 29.69778837 |
| Histone-lysine N-methyltransferase SETMAR || id:prot-a-2704 | rs500637 | A | G | 13 | 33516526 | 0.1657 | 3.72E-08 | 0.0301 | 0.73224 | 5.504983389 | 30.30484211 |
| Histone-lysine N-methyltransferase SUV420H2 || id:prot-a-2904 | rs12038333 | A | G | 1 | 196672454 | -0.1391 | 4.07E-08 | 0.0254 | 0.6091 | -5.476377953 | 29.99071548 |
| Histone-lysine N-methyltransferase SUV420H2 || id:prot-a-2904 | rs72797590 | T | C | 5 | 154134687 | -0.4449 | 4.37E-09 | 0.0758 | 0.0287 | -5.86939314 | 34.44977583 |
| Histone-lysine N-methyltransferase SUV420H2 || id:prot-a-2904 | rs704 | A | G | 17 | 26694861 | 0.1395 | 1.38E-08 | 0.0246 | 0.46665 | 5.670731707 | 32.1571981 |
| Histone acetyltransferase KAT6A || id:prot-a-1607 | rs368465 | C | T | 1 | 196671981 | -0.1469 | 8.51E-09 | 0.0255 | 0.61344 | -5.760784314 | 33.18663591 |
| Histone acetyltransferase KAT6A || id:prot-a-1607 | rs74480769 | G | A | 5 | 40972211 | -0.6214 | 1.66E-17 | 0.073 | 0.0317 | -8.512328767 | 72.45974104 |
| Histone deacetylase 8 || id:prot-a-1320 | rs7412 | T | C | 19 | 45412079 | -0.2782 | 1.78E-09 | 0.0462 | 0.07776 | -6.021645022 | 36.26020877 |
| Histone deacetylase complex subunit SAP18 || id:prot-a-2625 | rs41268617 | G | T | 3 | 186359810 | 0.1468 | 2.57E-08 | 0.0264 | 0.30785 | 5.560606061 | 30.92033976 |
| Histone deacetylase complex subunit SAP18 || id:prot-a-2625 | rs10424405 | G | A | 19 | 54321933 | 0.364 | 1.48E-34 | 0.0297 | 0.2203 | 12.25589226 | 150.206895 |
| Histone H1.2 || id:prot-a-1341 | rs3775298 | G | A | 4 | 187150478 | -0.1522 | 7.24E-10 | 0.0247 | 0.51335 | -6.16194332 | 37.96954548 |
| Histone H1.2 || id:prot-a-1341 | rs7080536 | A | G | 10 | 115348046 | -0.4066 | 6.61E-11 | 0.0623 | 0.04384 | -6.526484751 | 42.59500321 |
| Histone H1x || id:prot-a-1303 | rs10424405 | G | A | 19 | 54321933 | 0.3239 | 1.78E-27 | 0.0298 | 0.2203 | 10.86912752 | 118.137933 |
| Histone H2A deubiquitinase MYSM1 || id:prot-a-1983 | rs4485740 | C | A | 3 | 22046575 | 0.144 | 2.63E-08 | 0.0259 | 0.42564 | 5.55984556 | 30.91188265 |
| HLA class II histocompatibility antigen, DM alpha chain || id:prot-a-1345 | rs2853880 | G | C | 14 | 106239868 | -0.3074 | 2.00E-29 | 0.0273 | 0.34034 | -11.26007326 | 126.7892498 |
| HLA class II histocompatibility antigen, DM alpha chain || id:prot-a-1345 | rs73292853 | G | C | 17 | 16866777 | 0.2269 | 1.38E-08 | 0.04 | 0.10891 | 5.6725 | 32.17725625 |
| HLA class II histocompatibility antigen, DP beta 1 chain || id:prot-a-1346 | rs59123177 | A | G | 3 | 186391274 | 0.178 | 3.80E-12 | 0.0256 | 0.35103 | 6.953125 | 48.34594727 |
| HLA class II histocompatibility antigen, DP beta 1 chain || id:prot-a-1346 | rs908830 | C | T | 9 | 139840859 | -0.3673 | 4.90E-09 | 0.0628 | 0.95679 | -5.848726115 | 34.20759716 |
| HLA class II histocompatibility antigen, DQ alpha 2 chain || id:prot-a-1347 | rs9271547 | A | C | 6 | 32590159 | 0.4488 | 5.62E-80 | 0.0237 | 0.55086 | 18.93670886 | 358.5989425 |
| HLA class II histocompatibility antigen, DQ alpha 2 chain || id:prot-a-1347 | rs3135392 | A | C | 6 | 32409242 | -0.1401 | 2.51E-08 | 0.0251 | 0.3916 | -5.581673307 | 31.1550769 |
| HLA class II histocompatibility antigen, DQ alpha 2 chain || id:prot-a-1347 | rs1611236 | A | G | 6 | 29748690 | -0.1467 | 3.39E-08 | 0.0266 | 0.31728 | -5.515037594 | 30.41563966 |
| HLA class II histocompatibility antigen, DQ alpha 2 chain || id:prot-a-1347 | rs9271340 | C | T | 6 | 32582932 | -0.2212 | 6.17E-20 | 0.0242 | 0.46511 | -9.140495868 | 83.54866471 |
| Holo-Transcobalamin-2 || id:prot-a-2939 | rs73099617 | A | G | 3 | 68349712 | -0.264 | 2.95E-08 | 0.0476 | 0.07735 | -5.546218487 | 30.76053951 |
| Holo-Transcobalamin-2 || id:prot-a-2939 | rs9426 | T | C | 19 | 8367158 | 0.6251 | 5.50E-20 | 0.0683 | 0.03391 | 9.1522694 | 83.76403516 |
| Holo-Transcobalamin-2 || id:prot-a-2939 | rs56083844 | C | G | 22 | 30883196 | -0.4396 | 1.58E-18 | 0.05 | 0.06413 | -8.792 | 77.299264 |
| Holo-Transcobalamin-2 || id:prot-a-2939 | rs9619126 | G | A | 22 | 31007292 | -0.9058 | 2.34E-29 | 0.0805 | 0.02275 | -11.25217391 | 126.6114178 |
| Holo-Transcobalamin-2 || id:prot-a-2939 | rs4820885 | C | T | 22 | 31012756 | -0.7247 | 1.00E-200 | 0.0212 | 0.54911 | -34.18396226 | 1168.543276 |
| Homeodomain-interacting protein kinase 3 || id:prot-a-1340 | rs11685700 | G | A | 2 | 71162996 | -0.1589 | 4.79E-10 | 0.0255 | 0.60154 | -6.231372549 | 38.83000384 |
| Human Chorionic Gonadotropin || id:prot-a-527 | rs144071304 | A | G | 12 | 33501384 | -0.5208 | 3.72E-08 | 0.0946 | 0.01863 | -5.505285412 | 30.30816747 |
| Human Chorionic Gonadotropin || id:prot-a-527 | rs79785970 | G | C | 19 | 49517331 | 1.1095 | 1.00E-148 | 0.0427 | 0.07651 | 25.98360656 | 675.1478097 |
| Human Chorionic Gonadotropin || id:prot-a-527 | rs4801790 | G | A | 19 | 49552655 | 0.1519 | 3.89E-08 | 0.0276 | 0.29773 | 5.503623188 | 30.2898682 |
| Hyaluronan and proteoglycan link protein 1 || id:prot-a-1308 | rs72752593 | T | C | 1 | 226773270 | -0.4382 | 2.95E-08 | 0.079 | 0.02664 | -5.546835443 | 30.76738343 |
| Hyaluronidase-1 || id:prot-a-1393 | rs116482870 | T | C | 3 | 50339622 | -0.2784 | 1.95E-08 | 0.0495 | 0.06471 | -5.624242424 | 31.63210285 |
| Hyaluronidase-1 || id:prot-a-1393 | rs34417180 | A | C | 16 | 72219756 | 0.1853 | 2.75E-08 | 0.0334 | 0.16435 | 5.547904192 | 30.77924092 |
| Hydroxycarboxylic acid receptor 2 || id:prot-a-1318 | rs9564162 | C | T | 13 | 64963813 | 0.2118 | 4.47E-08 | 0.0387 | 0.11445 | 5.472868217 | 29.95228652 |
| Hydroxycarboxylic acid receptor 2 || id:prot-a-1318 | rs704 | A | G | 17 | 26694861 | -0.1553 | 2.51E-10 | 0.0245 | 0.46665 | -6.33877551 | 40.18007497 |
| ICOS ligand || id:prot-a-1405 | rs62143197 | A | G | 19 | 54320716 | 0.3726 | 6.31E-36 | 0.0298 | 0.22311 | 12.5033557 | 156.3339039 |
| ICOS ligand || id:prot-a-1405 | rs56124762 | G | A | 21 | 45658474 | -0.183 | 8.13E-11 | 0.0282 | 0.26242 | -6.489361702 | 42.1118153 |
| ICOS ligand || id:prot-a-1406 | rs11794023 | G | A | 9 | 93664706 | 0.201 | 2.75E-08 | 0.0362 | 0.1447 | 5.552486188 | 30.83010287 |
| ICOS ligand || id:prot-a-1406 | rs11558819 | T | C | 21 | 45656774 | -0.5762 | 3.55E-108 | 0.0261 | 0.26874 | -22.07662835 | 487.3775194 |
| Ig Kappa chain V-I region HK102- like || id:prot-a-1760 | rs964184 | C | G | 11 | 116648917 | -0.2232 | 8.71E-10 | 0.0364 | 0.86898 | -6.131868132 | 37.59980679 |
| IGF-like family receptor 1 || id:prot-a-1455 | rs12459634 | C | T | 19 | 36230174 | -0.7007 | 5.75E-100 | 0.033 | 0.14479 | -21.23333333 | 450.8544444 |
| IgLON family member 5 || id:prot-a-1459 | rs78088365 | C | T | 7 | 104223463 | -0.3538 | 1.26E-08 | 0.0622 | 0.04199 | -5.688102894 | 32.35451453 |
| Immunoglobulin-binding protein 1 || id:prot-a-1439 | rs62143197 | A | G | 19 | 54320716 | 0.3246 | 2.29E-27 | 0.0299 | 0.22311 | 10.85618729 | 117.8568025 |
| Immunoglobulin E || id:prot-a-1456 | rs9274055 | T | C | 6 | 32630083 | 0.1747 | 2.29E-10 | 0.0276 | 0.28446 | 6.329710145 | 40.06523052 |
| Immunoglobulin J chain || id:prot-a-1457 | rs10424405 | G | A | 19 | 54321933 | -0.2027 | 1.78E-11 | 0.0301 | 0.2203 | -6.734219269 | 45.34970916 |
| Immunoglobulin lambda-like polypeptide 1 || id:prot-a-1458 | rs58889466 | C | T | 5 | 158253683 | -0.3101 | 5.62E-12 | 0.045 | 0.07858 | -6.891111111 | 47.48741235 |
| Immunoglobulin lambda-like polypeptide 1 || id:prot-a-1458 | rs6947698 | C | G | 7 | 55954993 | 0.1362 | 4.79E-08 | 0.025 | 0.47386 | 5.448 | 29.680704 |
| Immunoglobulin lambda-like polypeptide 1 || id:prot-a-1458 | rs7008811 | G | T | 8 | 130600611 | -0.1663 | 3.02E-09 | 0.028 | 0.27511 | -5.939285714 | 35.2751148 |
| Immunoglobulin lambda-like polypeptide 1 || id:prot-a-1458 | rs7090445 | T | C | 10 | 63721176 | -0.1606 | 7.41E-10 | 0.0261 | 0.66601 | -6.153256705 | 37.86256808 |
| Immunoglobulin lambda-like polypeptide 1 || id:prot-a-1458 | rs1824465 | C | T | 13 | 72505308 | -0.3453 | 1.10E-08 | 0.0604 | 0.04534 | -5.716887417 | 32.68280174 |
| Immunoglobulin lambda-like polypeptide 1 || id:prot-a-1458 | rs2081048 | C | T | 19 | 33753166 | 0.1887 | 1.10E-12 | 0.0265 | 0.34448 | 7.120754717 | 50.70514774 |
| Immunoglobulin lambda-like polypeptide 1 || id:prot-a-1458 | rs139571703 | T | C | 22 | 23915620 | -0.7264 | 9.77E-26 | 0.0693 | 0.03738 | -10.48196248 | 109.8715375 |
| Immunoglobulin lambda-like polypeptide 1 || id:prot-a-1458 | rs117166029 | T | C | 22 | 23916424 | -0.7567 | 7.76E-12 | 0.1106 | 0.0129 | -6.841772152 | 46.80984618 |
| Immunoglobulin superfamily containing leucine-rich repeat protein 2 || id:prot-a-1576 | rs117210485 | A | G | 9 | 136388148 | 0.5153 | 5.37E-09 | 0.0883 | 0.02296 | 5.835787089 | 34.05641095 |
| Immunoglobulin superfamily containing leucine-rich repeat protein 2 || id:prot-a-1576 | rs115478735 | T | A | 9 | 136149711 | -0.4494 | 1.12E-46 | 0.0313 | 0.17824 | -14.35782748 | 206.1472098 |
| Immunoglobulin superfamily containing leucine-rich repeat protein 2 || id:prot-a-1576 | rs4055121 | T | C | 11 | 126232337 | -0.2662 | 5.50E-14 | 0.0354 | 0.14038 | -7.519774011 | 56.54700118 |
| Immunoglobulin superfamily containing leucine-rich repeat protein 2 || id:prot-a-1576 | rs2959011 | T | A | 15 | 74611781 | 0.198 | 3.16E-14 | 0.0261 | 0.34227 | 7.586206897 | 57.55053508 |
| Immunoglobulin superfamily DCC subclass member 3 || id:prot-a-1440 | rs188910381 | T | C | 4 | 162898530 | -0.4739 | 2.29E-08 | 0.0848 | 0.02376 | -5.588443396 | 31.23069959 |
| Immunoglobulin superfamily DCC subclass member 3 || id:prot-a-1440 | rs62143196 | G | A | 19 | 54320636 | 0.5839 | 3.16E-92 | 0.0287 | 0.22404 | 20.34494774 | 413.9168983 |
| Immunoglobulin superfamily DCC subclass member 4 || id:prot-a-1442 | rs184453313 | G | A | 15 | 65694949 | 0.9611 | 3.16E-19 | 0.1072 | 0.01548 | 8.965485075 | 80.37992262 |
| Immunoglobulin superfamily DCC subclass member 4 || id:prot-a-1442 | rs76702386 | C | T | 15 | 65912882 | -0.3955 | 6.76E-19 | 0.0445 | 0.08916 | -8.887640449 | 78.99015276 |
| Immunoglobulin superfamily DCC subclass member 4 || id:prot-a-1442 | rs8036706 | A | G | 15 | 65999235 | 0.7127 | 3.98E-13 | 0.0982 | 0.01761 | 7.257637475 | 52.67330171 |
| Immunoglobulin superfamily member 8 || id:prot-a-1462 | rs13402560 | G | C | 2 | 3639915 | -0.1933 | 3.80E-09 | 0.0328 | 0.81367 | -5.893292683 | 34.73089865 |
| Immunoglobulin superfamily member 8 || id:prot-a-1462 | rs10418046 | G | T | 19 | 54327869 | -0.1834 | 7.76E-10 | 0.0298 | 0.21727 | -6.154362416 | 37.87617675 |
| Importin subunit alpha-1 || id:prot-a-1677 | rs11867412 | T | C | 17 | 56382512 | 0.2109 | 4.17E-10 | 0.0338 | 0.1626 | 6.23964497 | 38.93316936 |
| Inactive gamma-glutamyltranspeptidase 2 || id:prot-a-1208 | rs1065853 | T | G | 19 | 45413233 | -0.8835 | 3.02E-90 | 0.0439 | 0.07778 | -20.12528474 | 405.0270858 |
| Inactive pancreatic lipase-related protein 1 || id:prot-a-2315 | rs55993634 | G | C | 16 | 75236763 | -0.3345 | 1.38E-14 | 0.0435 | 0.09403 | -7.689655172 | 59.13079667 |
| Inactive peptidyl-prolyl cis-trans isomerase FKBP6 || id:prot-a-1115 | rs704 | A | G | 17 | 26694861 | 1.0403 | 1.00E-200 | 0.0168 | 0.46665 | 61.92261905 | 3834.41075 |
| Inactive peptidyl-prolyl cis-trans isomerase FKBP6 || id:prot-a-1115 | rs117032462 | G | A | 17 | 26833853 | -0.4713 | 6.46E-12 | 0.0686 | 0.03301 | -6.870262391 | 47.20050532 |
| Inactive peptidyl-prolyl cis-trans isomerase FKBP6 || id:prot-a-1115 | rs5167 | G | T | 19 | 45448465 | 0.2054 | 1.48E-15 | 0.0257 | 0.34696 | 7.992217899 | 63.87554694 |
| Indoleamine 2,3-dioxygenase 1 || id:prot-a-1410 | rs7010461 | T | C | 8 | 39781444 | 0.25 | 2.45E-20 | 0.0271 | 0.33388 | 9.225092251 | 85.10232704 |
| Indoleamine 2,3-dioxygenase 1 || id:prot-a-1410 | rs4632248 | T | G | 19 | 54324995 | 0.4852 | 2.00E-63 | 0.0289 | 0.21439 | 16.78892734 | 281.8680811 |
| Induced myeloid leukemia cell differentiation protein Mcl-1 || id:prot-a-1868 | rs11580946 | A | G | 1 | 150551327 | 0.5676 | 2.45E-08 | 0.1018 | 0.015 | 5.575638507 | 31.08774476 |
| Inducible T-cell costimulator || id:prot-a-1403 | rs2498589 | T | G | 6 | 118030238 | -0.2825 | 2.09E-16 | 0.0344 | 0.15315 | -8.212209302 | 67.44038163 |
| Inducible T-cell costimulator || id:prot-a-1403 | rs146288564 | T | C | 14 | 103233504 | -0.6982 | 4.37E-08 | 0.1275 | 0.01197 | -5.476078431 | 29.98743499 |
| Inducible T-cell costimulator || id:prot-a-1403 | rs11849582 | G | A | 14 | 106195255 | 0.58 | 9.33E-115 | 0.0255 | 0.66802 | 22.74509804 | 517.3394848 |
| Inducible T-cell costimulator || id:prot-a-1404 | rs77694290 | A | G | 8 | 11474884 | 0.4799 | 3.80E-09 | 0.0815 | 0.02374 | 5.888343558 | 34.67258986 |
| Inhibin beta C chain || id:prot-a-1556 | rs4253282 | T | C | 4 | 187164399 | -0.1712 | 4.57E-12 | 0.0247 | 0.51241 | -6.931174089 | 48.04117425 |
| Inhibitor of growth protein 1 || id:prot-a-1552 | rs62143197 | A | G | 19 | 54320716 | 0.4059 | 1.15E-42 | 0.0296 | 0.22311 | 13.71283784 | 188.0419216 |
| Inosine-5'-monophosphate dehydrogenase 1 || id:prot-a-1549 | rs62143194 | G | C | 19 | 54319624 | 0.2349 | 1.29E-14 | 0.0305 | 0.22364 | 7.701639344 | 59.31524859 |
| Inosine-5'-monophosphate dehydrogenase 2 || id:prot-a-1550 | rs9467170 | A | G | 6 | 24452754 | 0.1472 | 2.19E-08 | 0.0263 | 0.33625 | 5.596958175 | 31.32594081 |
| Inosine-5'-monophosphate dehydrogenase 2 || id:prot-a-1550 | rs9567614 | A | C | 13 | 46649453 | 0.1472 | 2.00E-08 | 0.0262 | 0.32357 | 5.618320611 | 31.56552648 |
| Inositol 1,4,5-trisphosphate receptor-interacting protein-like 1 || id:prot-a-1594 | rs62143206 | T | G | 19 | 54326212 | 0.6626 | 8.51E-126 | 0.0278 | 0.2131 | 23.83453237 | 568.0849335 |
| Inositol monophosphatase 3 || id:prot-a-1548 | rs141514965 | T | A | 8 | 57934602 | -1.128 | 3.89E-32 | 0.0956 | 0.0172 | -11.79916318 | 139.2202517 |
| Inositol polyphosphate 5-phosphatase OCRL-1 || id:prot-a-2141 | rs704 | A | G | 17 | 26694861 | 0.387 | 1.15E-59 | 0.0238 | 0.46665 | 16.2605042 | 264.4039969 |
| Insulin-degrading enzyme || id:prot-a-1409 | rs35694084 | A | AT | 8 | 4276310 | -0.1772 | 2.75E-08 | 0.0319 | 0.25001 | -5.554858934 | 30.85645778 |
| Insulin-like 3 || id:prot-a-1560 | rs614877 | T | C | 17 | 26612996 | -0.1541 | 2.88E-10 | 0.0244 | 0.48058 | -6.31557377 | 39.88647205 |
| Insulin-like growth factor-binding protein 3 || id:prot-a-1449 | rs145188037 | A | G | 7 | 45954465 | 1.0329 | 1.15E-19 | 0.1138 | 0.01185 | 9.076449912 | 82.38194301 |
| Insulin-like growth factor-binding protein 7 || id:prot-a-1451 | rs1718849 | C | T | 4 | 57942323 | -0.3626 | 5.25E-38 | 0.0281 | 0.76629 | -12.90391459 | 166.5110118 |
| Insulin-like growth factor 1 receptor || id:prot-a-1444 | rs635634 | T | C | 9 | 136155000 | -0.2179 | 1.15E-11 | 0.0321 | 0.17981 | -6.788161994 | 46.07914325 |
| Insulin-like growth factor I || id:prot-a-1443 | rs528298 | T | A | 1 | 196660995 | -0.1419 | 2.34E-08 | 0.0254 | 0.61415 | -5.586614173 | 31.21025792 |
| Insulin-like growth factor I || id:prot-a-1443 | rs74480769 | G | A | 5 | 40972211 | -0.51 | 3.31E-12 | 0.0732 | 0.0317 | -6.967213115 | 48.54205859 |
| Insulin-like growth factor I || id:prot-a-1443 | rs2854746 | C | G | 7 | 45960645 | 0.1662 | 3.55E-11 | 0.0251 | 0.41457 | 6.621513944 | 43.84444691 |
| Insulin-like peptide INSL6 || id:prot-a-1563 | rs10418046 | G | T | 19 | 54327869 | -0.1641 | 3.80E-08 | 0.0298 | 0.21727 | -5.506711409 | 30.32387055 |
| Insulin growth factor-like family member 3 || id:prot-a-1453 | rs570618 | G | T | 1 | 196657064 | -0.1994 | 2.82E-15 | 0.0252 | 0.61149 | -7.912698413 | 62.61079617 |
| Insulin growth factor-like family member 3 || id:prot-a-1453 | rs74480769 | G | A | 5 | 40972211 | -0.5484 | 6.46E-14 | 0.0731 | 0.0317 | -7.502051984 | 56.28078396 |
| Insulin growth factor-like family member 3 || id:prot-a-1453 | rs704 | A | G | 17 | 26694861 | 0.2226 | 7.08E-20 | 0.0244 | 0.46665 | 9.12295082 | 83.22823166 |
| Insulin growth factor-like family member 4 || id:prot-a-1454 | rs1071803 | C | T | 14 | 106209119 | 0.4682 | 1.74E-72 | 0.026 | 0.66685 | 18.00769231 | 324.2769822 |
| Insulin receptor || id:prot-a-1564 | rs507666 | A | G | 9 | 136149399 | -0.5786 | 4.79E-80 | 0.0305 | 0.17941 | -18.9704918 | 359.8795593 |
| Insulin receptor || id:prot-a-1564 | rs760459 | T | A | 21 | 46328835 | 0.1683 | 1.82E-08 | 0.0299 | 0.78747 | 5.628762542 | 31.68296775 |
| Integral membrane protein 2A || id:prot-a-1589 | rs10418046 | G | T | 19 | 54327869 | 0.251 | 2.63E-17 | 0.0297 | 0.21727 | 8.451178451 | 71.42241721 |
| Integral membrane protein 2C || id:prot-a-1592 | rs10418046 | G | T | 19 | 54327869 | -0.19 | 1.82E-10 | 0.0298 | 0.21727 | -6.375838926 | 40.65132201 |
| Integrin alpha-5 || id:prot-a-1582 | rs10801553 | C | A | 1 | 196655743 | -0.1521 | 2.00E-09 | 0.0254 | 0.61291 | -5.988188976 | 35.85840722 |
| Integrin alpha-5 || id:prot-a-1582 | rs74480769 | G | A | 5 | 40972211 | -0.4155 | 1.51E-08 | 0.0734 | 0.0317 | -5.660762943 | 32.04423709 |
| Integrin alpha-5 || id:prot-a-1582 | rs59815279 | C | T | 6 | 150531969 | -0.1694 | 3.72E-08 | 0.0308 | 0.22403 | -5.5 | 30.25 |
| Integrin alpha-5 || id:prot-a-1582 | rs10424405 | G | A | 19 | 54321933 | -0.1845 | 9.77E-10 | 0.0302 | 0.2203 | -6.109271523 | 37.32319854 |
| Integrin alpha-I: beta-1 complex || id:prot-a-1581 | rs10424405 | G | A | 19 | 54321933 | 0.3734 | 2.24E-36 | 0.0296 | 0.2203 | 12.61486486 | 159.1348156 |
| Integrin alpha-L || id:prot-a-1583 | rs2159913 | C | A | 14 | 90846319 | 0.5319 | 3.24E-08 | 0.0962 | 0.01729 | 5.529106029 | 30.57101348 |
| Inter-alpha-trypsin inhibitor heavy chain H1 || id:prot-a-1586 | rs1042779 | G | A | 3 | 52821011 | -0.6463 | 1.55E-171 | 0.0231 | 0.36871 | -27.97835498 | 782.7883473 |
| Inter-alpha-trypsin inhibitor heavy chain H1 || id:prot-a-1586 | rs1056524 | A | G | 3 | 126261202 | 0.2822 | 8.32E-26 | 0.0269 | 0.29667 | 10.49070632 | 110.0549191 |
| Inter-alpha-trypsin inhibitor heavy chain H1 || id:prot-a-1586 | rs4298806 | G | T | 10 | 7753089 | 0.5517 | 1.51E-27 | 0.0507 | 0.06238 | 10.8816568 | 118.4104548 |
| Inter-alpha-trypsin inhibitor heavy chain H5 || id:prot-a-1587 | rs7909223 | G | A | 10 | 7700709 | 0.3222 | 2.00E-29 | 0.0286 | 0.74769 | 11.26573427 | 126.9167685 |
| Inter-alpha-trypsin inhibitor heavy chain H5 || id:prot-a-1587 | rs11255233 | C | T | 10 | 7648273 | -0.2195 | 2.40E-14 | 0.0288 | 0.24187 | -7.621527778 | 58.08768567 |
| Inter-alpha-trypsin inhibitor heavy chain H5 || id:prot-a-1587 | rs149562316 | G | GTA | 10 | 7607266 | 0.2416 | 5.01E-17 | 0.0288 | 0.26391 | 8.388888889 | 70.37345679 |
| Intercellular adhesion molecule 1 || id:prot-a-1397 | rs507666 | A | G | 9 | 136149399 | -0.2711 | 1.58E-17 | 0.0318 | 0.17941 | -8.525157233 | 72.67830584 |
| Intercellular adhesion molecule 1 || id:prot-a-1397 | rs5030366 | T | A | 19 | 10391009 | 0.3923 | 7.94E-14 | 0.0525 | 0.06768 | 7.472380952 | 55.8364771 |
| Intercellular adhesion molecule 1 || id:prot-a-1397 | rs5498 | G | A | 19 | 10395683 | -1.1988 | 1.00E-200 | 0.0136 | 0.43051 | -88.14705882 | 7769.903979 |
| Intercellular adhesion molecule 2 || id:prot-a-1398 | rs651007 | T | C | 9 | 136153875 | -0.2253 | 7.59E-14 | 0.0301 | 0.20669 | -7.485049834 | 56.02597102 |
| Intercellular adhesion molecule 2 || id:prot-a-1398 | rs3184504 | C | T | 12 | 111884608 | -0.138 | 1.62E-08 | 0.0244 | 0.51716 | -5.655737705 | 31.98736899 |
| Intercellular adhesion molecule 4 || id:prot-a-1400 | rs72660908 | G | C | 1 | 25583610 | -0.6177 | 4.68E-159 | 0.023 | 0.45371 | -26.85652174 | 721.2727599 |
| Intercellular adhesion molecule 4 || id:prot-a-1400 | rs635634 | T | C | 9 | 136155000 | -0.2105 | 5.62E-11 | 0.0321 | 0.17981 | -6.557632399 | 43.00254268 |
| Intercellular adhesion molecule 5 || id:prot-a-1401 | rs635634 | T | C | 9 | 136155000 | -0.2755 | 6.92E-18 | 0.032 | 0.17981 | -8.609375 | 74.12133789 |
| Intercellular adhesion molecule 5 || id:prot-a-1401 | rs281439 | C | G | 19 | 10400110 | 0.7187 | 3.80E-152 | 0.0274 | 0.78041 | 26.22992701 | 688.0090708 |
| Intercellular adhesion molecule 5 || id:prot-a-1401 | rs117707627 | G | A | 19 | 10726306 | -0.4219 | 1.26E-12 | 0.0594 | 0.04891 | -7.102693603 | 50.44825641 |
| Intercellular adhesion molecule 5 || id:prot-a-1402 | rs635634 | T | C | 9 | 136155000 | -0.4181 | 3.39E-40 | 0.0315 | 0.17981 | -13.27301587 | 176.1729504 |
| Intercellular adhesion molecule 5 || id:prot-a-1402 | rs117707627 | G | A | 19 | 10726306 | -0.4813 | 4.90E-16 | 0.0593 | 0.04891 | -8.116357504 | 65.87525914 |
| Intercellular adhesion molecule 5 || id:prot-a-1402 | rs281439 | C | G | 19 | 10400110 | 0.8918 | 1.00E-200 | 0.0258 | 0.78041 | 34.56589147 | 1194.800853 |
| Interferon-induced protein with tetratricopeptide repeats 2 || id:prot-a-1416 | rs59325138 | T | C | 19 | 45416291 | 0.1436 | 1.62E-08 | 0.0254 | 0.39836 | 5.653543307 | 31.96255193 |
| Interferon-induced protein with tetratricopeptide repeats 3 || id:prot-a-1417 | rs62143198 | A | G | 19 | 54320939 | 0.3291 | 1.20E-27 | 0.0302 | 0.21453 | 10.89735099 | 118.7522587 |
| Interferon alpha-10 || id:prot-a-1418 | rs7162385 | T | C | 15 | 90001851 | -0.4155 | 4.17E-08 | 0.0758 | 0.02782 | -5.481530343 | 30.0471749 |
| Interferon alpha-14 || id:prot-a-1420 | rs137994678 | T | C | 6 | 127819910 | 0.4951 | 1.86E-08 | 0.088 | 0.02656 | 5.626136364 | 31.65341038 |
| Interferon alpha-5 || id:prot-a-1422 | rs33944729 | T | TA | 1 | 196652124 | 0.1411 | 2.63E-08 | 0.0254 | 0.38701 | 5.55511811 | 30.85933722 |
| Interferon alpha-5 || id:prot-a-1422 | rs76639786 | T | C | 11 | 22519757 | -0.572 | 6.46E-09 | 0.0986 | 0.01768 | -5.801217039 | 33.65411913 |
| Interferon alpha/beta receptor 1 || id:prot-a-1426 | rs2257167 | C | G | 21 | 34715699 | -0.2897 | 1.45E-15 | 0.0363 | 0.13427 | -7.980716253 | 63.69183192 |
| Interferon gamma || id:prot-a-1428 | rs7567468 | T | C | 2 | 234642838 | 0.1624 | 4.07E-09 | 0.0276 | 0.27772 | 5.884057971 | 34.62213821 |
| Interferon gamma || id:prot-a-1428 | rs7459901 | T | G | 8 | 58281744 | 0.3041 | 3.09E-08 | 0.0549 | 0.05504 | 5.539162113 | 30.68231691 |
| Interferon gamma receptor 1 || id:prot-a-1430 | rs7080536 | A | G | 10 | 115348046 | 0.6262 | 3.47E-24 | 0.0617 | 0.04384 | 10.14910859 | 103.0044052 |
| Interferon gamma receptor 2 || id:prot-a-1432 | rs4540249 | T | G | 6 | 170492449 | -0.1567 | 4.68E-09 | 0.0267 | 0.65132 | -5.868913858 | 34.44414987 |
| Interferon gamma receptor 2 || id:prot-a-1432 | rs72639485 | C | T | 18 | 31903382 | -0.2512 | 1.82E-08 | 0.0446 | 0.08354 | -5.632286996 | 31.7226568 |
| Interferon lambda-2 || id:prot-a-1436 | rs62143206 | T | G | 19 | 54326212 | -0.1741 | 5.89E-09 | 0.0299 | 0.2131 | -5.822742475 | 33.90432993 |
| Interferon regulatory factor 2 || id:prot-a-1568 | rs62143206 | T | G | 19 | 54326212 | 0.6258 | 1.95E-110 | 0.028 | 0.2131 | 22.35 | 499.5225 |
| Interferon regulatory factor 4 || id:prot-a-1569 | rs10424405 | G | A | 19 | 54321933 | 0.1771 | 4.47E-09 | 0.0302 | 0.2203 | 5.864238411 | 34.38929214 |
| Interferon regulatory factor 6 || id:prot-a-1570 | rs9881048 | C | A | 3 | 165506252 | -0.1795 | 2.00E-08 | 0.032 | 0.8162 | -5.609375 | 31.46508789 |
| Interleukin-1 beta || id:prot-a-1495 | rs13402561 | G | C | 2 | 3639921 | -0.3247 | 2.19E-23 | 0.0326 | 0.81414 | -9.960122699 | 99.20404419 |
| Interleukin-1 beta || id:prot-a-1495 | rs967645 | T | C | 17 | 26713970 | -0.1427 | 5.13E-09 | 0.0244 | 0.50945 | -5.848360656 | 34.20332236 |
| Interleukin-1 receptor-like 1 || id:prot-a-1502 | rs6725806 | G | T | 2 | 103216172 | -0.2485 | 1.29E-17 | 0.0291 | 0.75252 | -8.5395189 | 72.92338305 |
| Interleukin-1 receptor-like 1 || id:prot-a-1502 | rs10179654 | G | T | 2 | 102921783 | -0.8483 | 1.00E-200 | 0.02 | 0.52102 | -42.415 | 1799.032225 |
| Interleukin-1 receptor-like 1 || id:prot-a-1502 | rs6742931 | T | C | 2 | 102851243 | -0.2874 | 4.07E-26 | 0.0272 | 0.26749 | -10.56617647 | 111.6440852 |
| Interleukin-1 receptor-like 1 || id:prot-a-1502 | rs672806 | G | A | 11 | 126188405 | -0.1503 | 1.66E-09 | 0.0249 | 0.58872 | -6.036144578 | 36.43504137 |
| Interleukin-1 receptor-like 1 || id:prot-a-1502 | rs149567682 | C | T | 13 | 36700456 | -0.596 | 3.16E-08 | 0.1078 | 0.01434 | -5.528756957 | 30.56715349 |
| Interleukin-1 receptor-like 2 || id:prot-a-1503 | rs2228139 | G | C | 2 | 102781649 | -0.4249 | 1.62E-18 | 0.0484 | 0.06922 | -8.77892562 | 77.06953504 |
| Interleukin-1 receptor-like 2 || id:prot-a-1503 | rs62143196 | G | A | 19 | 54320636 | -0.1682 | 2.75E-08 | 0.0303 | 0.22404 | -5.551155116 | 30.81532312 |
| Interleukin-1 Receptor accessory protein || id:prot-a-1499 | rs146968867 | T | TTGAG | 3 | 190556018 | 0.2596 | 1.17E-09 | 0.0427 | 0.1077 | 6.079625293 | 36.9618437 |
| Interleukin-1 Receptor accessory protein || id:prot-a-1499 | rs6444442 | G | A | 3 | 190346060 | -1.352 | 1.00E-200 | 0.0246 | 0.84101 | -54.95934959 | 3020.530108 |
| Interleukin-1 Receptor accessory protein || id:prot-a-1499 | rs3935774 | T | C | 3 | 190360571 | 0.7411 | 3.55E-11 | 0.1119 | 0.01707 | 6.622877569 | 43.8625073 |
| Interleukin-1 Receptor accessory protein || id:prot-a-1500 | rs6444442 | G | A | 3 | 190346060 | -1.3827 | 1.00E-200 | 0.0241 | 0.84101 | -57.37344398 | 3291.712075 |
| Interleukin-1 Receptor accessory protein || id:prot-a-1500 | rs146968867 | T | TTGAG | 3 | 190556018 | 0.2843 | 2.57E-11 | 0.0426 | 0.1077 | 6.67370892 | 44.53839075 |
| Interleukin-1 Receptor accessory protein || id:prot-a-1500 | rs3935774 | T | C | 3 | 190360571 | 0.796 | 1.10E-12 | 0.1118 | 0.01707 | 7.119856887 | 50.6923621 |
| Interleukin-1 receptor antagonist protein || id:prot-a-1504 | rs6761276 | C | T | 2 | 113832312 | -0.1907 | 1.51E-14 | 0.0248 | 0.5777 | -7.689516129 | 59.1286583 |
| Interleukin-1 receptor antagonist protein || id:prot-a-1504 | rs7748962 | A | G | 6 | 43759927 | -0.1684 | 4.57E-08 | 0.0308 | 0.77686 | -5.467532468 | 29.89391128 |
| Interleukin-1 receptor type 1 || id:prot-a-1496 | rs55923630 | T | A | 3 | 118806101 | -0.1494 | 1.35E-08 | 0.0263 | 0.3028 | -5.680608365 | 32.2693114 |
| Interleukin-1 receptor type 2 || id:prot-a-1497 | rs7561460 | C | T | 2 | 102617204 | -0.4025 | 6.17E-62 | 0.0242 | 0.39587 | -16.6322314 | 276.6311215 |
| Interleukin-1 receptor type 2 || id:prot-a-1497 | rs3862628 | A | G | 11 | 126242912 | -0.1922 | 1.62E-10 | 0.0301 | 0.23041 | -6.38538206 | 40.77310405 |
| Interleukin-1 receptor type 2 || id:prot-a-1498 | rs62143194 | G | C | 19 | 54319624 | -0.1956 | 1.55E-10 | 0.0306 | 0.22364 | -6.392156863 | 40.85966936 |
| Interleukin-10 || id:prot-a-1464 | rs62143196 | G | A | 19 | 54320636 | -0.1667 | 3.63E-08 | 0.0303 | 0.22404 | -5.501650165 | 30.26815454 |
| Interleukin-10 receptor subunit beta || id:prot-a-1465 | rs62143196 | G | A | 19 | 54320636 | -0.1708 | 1.66E-08 | 0.0303 | 0.22404 | -5.636963696 | 31.77535971 |
| Interleukin-11 receptor subunit alpha || id:prot-a-1467 | rs11575578 | A | G | 9 | 34656479 | 0.5065 | 4.37E-26 | 0.0479 | 0.06849 | 10.57411273 | 111.8118601 |
| Interleukin-12 || id:prot-a-1468 | rs7208047 | G | A | 17 | 49620242 | -0.1485 | 3.55E-08 | 0.0269 | 0.56885 | -5.520446097 | 30.47532511 |
| Interleukin-12 receptor subunit beta-1 || id:prot-a-1473 | rs62143196 | G | A | 19 | 54320636 | -0.2229 | 1.48E-13 | 0.0302 | 0.22404 | -7.380794702 | 54.47613043 |
| Interleukin-12 receptor subunit beta-2 || id:prot-a-1474 | rs12566098 | G | C | 1 | 67889571 | 0.2568 | 6.03E-22 | 0.0267 | 0.68806 | 9.617977528 | 92.50549173 |
| Interleukin-12 receptor subunit beta-2 || id:prot-a-1474 | rs78137303 | G | A | 17 | 13741139 | -0.3568 | 3.39E-09 | 0.0603 | 0.05202 | -5.91708126 | 35.01185064 |
| Interleukin-13 || id:prot-a-1475 | rs10418046 | G | T | 19 | 54327869 | -0.1638 | 4.07E-08 | 0.0298 | 0.21727 | -5.496644295 | 30.21309851 |
| Interleukin-13 receptor subunit alpha-1 || id:prot-a-1476 | rs4241818 | C | T | 4 | 187153786 | 0.1924 | 5.13E-15 | 0.0246 | 0.51359 | 7.821138211 | 61.17020292 |
| Interleukin-13 receptor subunit alpha-1 || id:prot-a-1476 | rs113851752 | G | C | 15 | 68843506 | -0.3881 | 6.03E-09 | 0.0667 | 0.03649 | -5.818590705 | 33.85599779 |
| Interleukin-15 receptor subunit alpha || id:prot-a-1477 | rs8177641 | G | A | 10 | 6016892 | 0.5091 | 1.66E-91 | 0.0251 | 0.31781 | 20.28286853 | 411.3947556 |
| Interleukin-15 receptor subunit alpha || id:prot-a-1477 | rs2296141 | T | C | 10 | 5998659 | -0.2645 | 1.82E-09 | 0.044 | 0.08926 | -6.011363636 | 36.13649277 |
| Interleukin-15 receptor subunit alpha || id:prot-a-1478 | rs8177654 | C | T | 10 | 6015197 | 0.2834 | 1.45E-27 | 0.026 | 0.3245 | 10.9 | 118.81 |
| Interleukin-16 || id:prot-a-1479 | rs4778639 | G | T | 15 | 81600451 | -0.6805 | 4.47E-59 | 0.042 | 0.09142 | -16.20238095 | 262.5171485 |
| Interleukin-16 || id:prot-a-1479 | rs62143206 | T | G | 19 | 54326212 | 0.505 | 4.90E-69 | 0.0288 | 0.2131 | 17.53472222 | 307.4664834 |
| Interleukin-17 receptor A || id:prot-a-1486 | rs5994170 | G | A | 22 | 17615213 | -0.3043 | 5.25E-34 | 0.025 | 0.39114 | -12.172 | 148.157584 |
| Interleukin-17 receptor A || id:prot-a-1486 | rs4819959 | A | G | 22 | 17586631 | 0.9127 | 1.00E-200 | 0.0195 | 0.49643 | 46.80512821 | 2190.720026 |
| Interleukin-17 receptor A || id:prot-a-1486 | rs17807317 | C | A | 22 | 17680519 | 0.1646 | 2.57E-11 | 0.0247 | 0.49754 | 6.663967611 | 44.40846432 |
| Interleukin-17 receptor B || id:prot-a-1487 | rs11706303 | C | T | 3 | 53740752 | 0.2189 | 4.37E-14 | 0.029 | 0.28525 | 7.548275862 | 56.97646849 |
| Interleukin-17 receptor B || id:prot-a-1487 | rs2232346 | C | T | 3 | 53892830 | 1.1818 | 3.55E-77 | 0.0636 | 0.03659 | 18.58176101 | 345.2818421 |
| Interleukin-17 receptor C || id:prot-a-1488 | rs148701877 | CT | C | 7 | 129403296 | -0.3271 | 2.57E-08 | 0.0587 | 0.0483 | -5.572402044 | 31.05166454 |
| Interleukin-17 receptor D || id:prot-a-1489 | rs6776722 | A | G | 3 | 57142659 | -0.4623 | 3.39E-74 | 0.0254 | 0.69533 | -18.2007874 | 331.268662 |
| Interleukin-17A || id:prot-a-1482 | rs114163150 | G | A | 5 | 24337202 | -0.3985 | 1.00E-08 | 0.0695 | 0.03407 | -5.73381295 | 32.87661094 |
| Interleukin-17F || id:prot-a-1485 | rs9274952 | G | T | 6 | 32641868 | 0.1677 | 2.40E-08 | 0.03 | 0.36485 | 5.59 | 31.2481 |
| Interleukin-18 receptor 1 || id:prot-a-1491 | rs2001461 | C | T | 2 | 103007220 | -0.6751 | 2.63E-129 | 0.0279 | 0.77792 | -24.19713262 | 585.5012269 |
| Interleukin-18 receptor 1 || id:prot-a-1492 | rs1420106 | G | A | 2 | 103035044 | -0.9085 | 1.00E-200 | 0.0257 | 0.77555 | -35.35019455 | 1249.636255 |
| Interleukin-18 receptor 1 || id:prot-a-1492 | rs77435902 | G | T | 2 | 102801413 | 0.3504 | 6.17E-10 | 0.0566 | 0.05266 | 6.190812721 | 38.32616214 |
| Interleukin-19 || id:prot-a-1493 | rs3820897 | C | T | 2 | 3642361 | -0.8113 | 3.09E-158 | 0.0303 | 0.82077 | -26.77557756 | 716.9315536 |
| Interleukin-2 || id:prot-a-1512 | rs4241819 | T | C | 4 | 187157140 | 0.1919 | 7.08E-15 | 0.0247 | 0.50721 | 7.769230769 | 60.36094675 |
| Interleukin-2 || id:prot-a-1512 | rs2731674 | G | T | 5 | 176839890 | 0.1617 | 9.55E-09 | 0.0282 | 0.75301 | 5.734042553 | 32.879244 |
| Interleukin-2 receptor subunit beta || id:prot-a-1519 | rs3184504 | C | T | 12 | 111884608 | -0.1473 | 1.70E-09 | 0.0244 | 0.51716 | -6.036885246 | 36.44398347 |
| Interleukin-21 || id:prot-a-1506 | rs28383364 | G | A | 6 | 32606912 | 0.2669 | 5.13E-18 | 0.0308 | 0.19996 | 8.665584416 | 75.09235326 |
| Interleukin-21 || id:prot-a-1506 | rs3129897 | A | G | 6 | 32421469 | -0.3747 | 1.70E-29 | 0.0332 | 0.15627 | -11.28614458 | 127.3770594 |
| Interleukin-21 || id:prot-a-1506 | rs12368181 | G | A | 12 | 7181105 | -0.3688 | 2.00E-24 | 0.0362 | 0.13305 | -10.1878453 | 103.7921919 |
| Interleukin-21 || id:prot-a-1506 | rs11624581 | T | C | 14 | 106188309 | 0.1547 | 3.09E-08 | 0.0279 | 0.65137 | 5.544802867 | 30.74483884 |
| Interleukin-22 || id:prot-a-1507 | rs7870825 | C | T | 9 | 12201384 | 0.1467 | 2.40E-08 | 0.0263 | 0.62611 | 5.577946768 | 31.11349015 |
| Interleukin-22 receptor subunit alpha-1 || id:prot-a-1508 | rs1065853 | T | G | 19 | 45413233 | -0.3498 | 3.16E-14 | 0.0461 | 0.07778 | -7.587852495 | 57.57550548 |
| Interleukin-22 receptor subunit alpha-2 || id:prot-a-1510 | rs11249222 | G | T | 1 | 25305783 | 0.1478 | 1.23E-08 | 0.0259 | 0.44083 | 5.706563707 | 32.56486934 |
| Interleukin-22 receptor subunit alpha-2 || id:prot-a-1510 | rs6928015 | T | G | 6 | 137517888 | 0.1985 | 1.02E-08 | 0.0347 | 0.16974 | 5.720461095 | 32.72367514 |
| Interleukin-22 receptor subunit alpha-2 || id:prot-a-1511 | rs1473233 | C | T | 2 | 8499766 | -0.1895 | 2.40E-08 | 0.0339 | 0.84475 | -5.589970501 | 31.24777021 |
| Interleukin-22 receptor subunit alpha-2 || id:prot-a-1511 | rs10457655 | A | G | 6 | 137539810 | 0.2152 | 1.12E-10 | 0.0334 | 0.16843 | 6.443113772 | 41.51371508 |
| Interleukin-22 receptor subunit alpha-2 || id:prot-a-1511 | rs7406661 | C | T | 17 | 7063667 | 0.1791 | 1.74E-08 | 0.0318 | 0.19726 | 5.632075472 | 31.72027412 |
| Interleukin-23 || id:prot-a-1471 | rs9815073 | A | C | 3 | 188115682 | 0.2146 | 8.71E-15 | 0.0277 | 0.37426 | 7.747292419 | 60.02053982 |
| Interleukin-23 || id:prot-a-1471 | rs4921223 | G | A | 5 | 158792306 | 0.3111 | 8.32E-33 | 0.0261 | 0.67726 | 11.91954023 | 142.0754393 |
| Interleukin-23 receptor || id:prot-a-1513 | rs11581607 | A | G | 1 | 67707690 | -0.42 | 1.23E-17 | 0.0491 | 0.06695 | -8.553971487 | 73.1704282 |
| Interleukin-23 receptor || id:prot-a-1513 | rs1884444 | T | G | 1 | 67633812 | -0.1489 | 1.12E-09 | 0.0245 | 0.53031 | -6.07755102 | 36.93662641 |
| Interleukin-25 || id:prot-a-1515 | rs2508490 | A | G | 11 | 120099679 | -0.8195 | 5.89E-194 | 0.0276 | 0.79812 | -29.69202899 | 881.6165853 |
| Interleukin-27 || id:prot-a-1516 | rs181209 | T | G | 16 | 28514854 | -0.1809 | 8.71E-12 | 0.0265 | 0.32698 | -6.826415094 | 46.59994304 |
| Interleukin-27 receptor subunit alpha || id:prot-a-1517 | rs8176743 | T | C | 9 | 136131415 | 0.4929 | 3.89E-24 | 0.0486 | 0.06688 | 10.14197531 | 102.8596632 |
| Interleukin-27 receptor subunit alpha || id:prot-a-1517 | rs35026308 | C | T | 19 | 14153293 | -0.9457 | 1.00E-200 | 0.0286 | 0.17644 | -33.06643357 | 1093.389029 |
| Interleukin-3 || id:prot-a-1525 | rs1853806 | T | G | 1 | 152591413 | -0.4108 | 1.38E-08 | 0.0724 | 0.03251 | -5.674033149 | 32.19465218 |
| Interleukin-3 receptor subunit alpha || id:prot-a-1530 | rs3128759 | T | C | 6 | 31885930 | -0.1487 | 4.90E-09 | 0.0254 | 0.62573 | -5.854330709 | 34.27318805 |
| Interleukin-3 receptor subunit alpha || id:prot-a-1530 | rs2519093 | T | C | 9 | 136141870 | -0.8577 | 8.32E-198 | 0.0286 | 0.17823 | -29.98951049 | 899.3707394 |
| Interleukin-3 receptor subunit alpha || id:prot-a-1530 | rs41307428 | T | C | 9 | 136336804 | 0.6313 | 5.01E-15 | 0.0807 | 0.02484 | 7.822800496 | 61.19620759 |
| Interleukin-3 receptor subunit alpha || id:prot-a-1531 | rs137152 | C | T | 22 | 27929482 | -0.3495 | 9.55E-09 | 0.0609 | 0.95448 | -5.738916256 | 32.9351598 |
| Interleukin-31 || id:prot-a-1521 | rs704 | A | G | 17 | 26694861 | 0.4487 | 8.71E-82 | 0.0234 | 0.46665 | 19.17521368 | 367.6888195 |
| Interleukin-34 || id:prot-a-1524 | rs368465 | C | T | 1 | 196671981 | -0.1736 | 9.12E-12 | 0.0255 | 0.61344 | -6.807843137 | 46.34672818 |
| Interleukin-34 || id:prot-a-1524 | rs74480769 | G | A | 5 | 40972211 | -0.5788 | 2.34E-15 | 0.0731 | 0.0317 | -7.917920657 | 62.69346752 |
| Interleukin-36 alpha || id:prot-a-1526 | rs10922094 | C | G | 1 | 196661505 | -0.1468 | 7.24E-09 | 0.0254 | 0.61211 | -5.779527559 | 33.40293881 |
| Interleukin-36 alpha || id:prot-a-1526 | rs74480769 | G | A | 5 | 40972211 | -0.494 | 1.55E-11 | 0.0733 | 0.0317 | -6.739427012 | 45.41987645 |
| Interleukin-36 alpha || id:prot-a-1526 | rs704 | A | G | 17 | 26694861 | 0.3353 | 2.24E-44 | 0.024 | 0.46665 | 13.97083333 | 195.184184 |
| Interleukin-37 || id:prot-a-1529 | rs7539005 | T | A | 1 | 196667252 | -0.1696 | 2.57E-11 | 0.0254 | 0.61028 | -6.677165354 | 44.58453717 |
| Interleukin-37 || id:prot-a-1529 | rs74480769 | G | A | 5 | 40972211 | -0.5156 | 1.91E-12 | 0.0732 | 0.0317 | -7.043715847 | 49.61393293 |
| Interleukin-4 receptor subunit alpha || id:prot-a-1533 | rs10418046 | G | T | 19 | 54327869 | -0.1694 | 1.38E-08 | 0.0298 | 0.21727 | -5.684563758 | 32.31426512 |
| Interleukin-5 || id:prot-a-1535 | rs75995134 | T | C | 1 | 15702987 | 0.6632 | 4.37E-08 | 0.1211 | 0.01064 | 5.476465731 | 29.9916769 |
| Interleukin-5 || id:prot-a-1535 | rs704 | A | G | 17 | 26694861 | -0.2887 | 6.92E-33 | 0.0242 | 0.46665 | -11.92975207 | 142.3189844 |
| Interleukin-5 receptor subunit alpha || id:prot-a-1536 | rs9851301 | A | G | 3 | 3137782 | 0.23 | 6.92E-18 | 0.0267 | 0.34355 | 8.61423221 | 74.20499656 |
| Interleukin-5 receptor subunit alpha || id:prot-a-1536 | rs9831674 | A | G | 3 | 3158973 | -0.1659 | 2.69E-08 | 0.0298 | 0.23138 | -5.567114094 | 30.99275934 |
| Interleukin-5 receptor subunit alpha || id:prot-a-1537 | rs340827 | A | G | 3 | 3107730 | 0.1452 | 2.29E-08 | 0.026 | 0.42344 | 5.584615385 | 31.18792899 |
| Interleukin-5 receptor subunit alpha || id:prot-a-1537 | rs77400868 | G | A | 3 | 3150964 | 0.5096 | 6.76E-45 | 0.0362 | 0.13908 | 14.07734807 | 198.1717286 |
| Interleukin-5 receptor subunit alpha || id:prot-a-1537 | rs9275965 | A | C | 6 | 32693482 | -0.1685 | 2.51E-08 | 0.0302 | 0.21796 | -5.579470199 | 31.1304877 |
| Interleukin-6 || id:prot-a-1539 | rs75892156 | A | G | 12 | 34552764 | 0.3168 | 7.24E-09 | 0.0548 | 0.0572 | 5.781021898 | 33.42021418 |
| Interleukin-6 || id:prot-a-1539 | rs11872808 | T | C | 18 | 4331629 | 0.2297 | 2.19E-08 | 0.0411 | 0.11345 | 5.588807786 | 31.23477247 |
| Interleukin-6 receptor subunit alpha || id:prot-a-1540 | rs12126142 | A | G | 1 | 154425456 | 1.1104 | 1.00E-200 | 0.0156 | 0.40039 | 71.17948718 | 5066.519395 |
| Interleukin-6 receptor subunit alpha || id:prot-a-1540 | rs6688376 | T | C | 1 | 154326366 | -0.2021 | 2.57E-14 | 0.0265 | 0.30876 | -7.626415094 | 58.16220719 |
| Interleukin-6 receptor subunit alpha || id:prot-a-1541 | rs6688376 | T | C | 1 | 154326366 | -0.2148 | 5.13E-16 | 0.0265 | 0.30876 | -8.105660377 | 65.70173015 |
| Interleukin-6 receptor subunit alpha || id:prot-a-1541 | rs4129267 | T | C | 1 | 154426264 | 1.1023 | 1.00E-200 | 0.0159 | 0.40005 | 69.32704403 | 4806.239033 |
| Interleukin-6 receptor subunit beta || id:prot-a-1542 | rs11927405 | T | C | 3 | 98402969 | -0.1476 | 5.50E-09 | 0.0253 | 0.44847 | -5.833992095 | 34.03546376 |
| Interleukin-6 receptor subunit beta || id:prot-a-1542 | rs11574765 | G | A | 5 | 55278967 | 0.3809 | 1.05E-22 | 0.0388 | 0.11565 | 9.817010309 | 96.37369141 |
| Interleukin-6 receptor subunit beta || id:prot-a-1542 | rs635634 | T | C | 9 | 136155000 | -0.2316 | 5.25E-13 | 0.0321 | 0.17981 | -7.214953271 | 52.0555507 |
| Interleukin-6 receptor subunit beta || id:prot-a-1542 | rs3862628 | A | G | 11 | 126242912 | -0.2163 | 5.75E-13 | 0.03 | 0.23041 | -7.21 | 51.9841 |
| Interleukin-7 || id:prot-a-1543 | rs72673751 | C | T | 8 | 106578940 | -0.1844 | 8.91E-09 | 0.0321 | 0.18949 | -5.744548287 | 32.99983502 |
| Interleukin-7 receptor subunit alpha || id:prot-a-1544 | rs6451229 | G | A | 5 | 35866218 | 0.2941 | 1.29E-32 | 0.0247 | 0.40041 | 11.90688259 | 141.773853 |
| Interleukin-7 receptor subunit alpha || id:prot-a-1544 | rs34436714 | A | C | 19 | 54327313 | -0.2441 | 3.31E-16 | 0.0299 | 0.21294 | -8.163879599 | 66.6489301 |
| Intersectin-1 || id:prot-a-1595 | rs704 | A | G | 17 | 26694861 | 0.2151 | 1.23E-18 | 0.0244 | 0.46665 | 8.81557377 | 77.7143409 |
| Intestinal-type alkaline phosphatase || id:prot-a-81 | rs550057 | T | C | 9 | 136146597 | -0.2543 | 2.75E-19 | 0.0283 | 0.24577 | -8.985865724 | 80.74578282 |
| Intestinal-type alkaline phosphatase || id:prot-a-81 | rs679574 | G | C | 19 | 49206108 | -0.1949 | 9.77E-16 | 0.0243 | 0.50693 | -8.020576132 | 64.32964148 |
| Isochorismatase domain-containing protein 1 || id:prot-a-1578 | rs13402561 | G | C | 2 | 3639921 | -0.2342 | 9.77E-13 | 0.0328 | 0.81414 | -7.140243902 | 50.98308299 |
| IST1 homolog || id:prot-a-1579 | rs62143197 | A | G | 19 | 54320716 | 0.4552 | 5.75E-54 | 0.0294 | 0.22311 | 15.4829932 | 239.7230783 |
| Isthmin-1 || id:prot-a-1577 | rs704 | A | G | 17 | 26694861 | 0.1986 | 4.68E-16 | 0.0245 | 0.46665 | 8.106122449 | 65.70922116 |
| Janus kinase and microtubule-interacting protein 3 || id:prot-a-1599 | rs1042779 | G | A | 3 | 52821011 | 0.7855 | 1.00E-200 | 0.0218 | 0.36871 | 36.03211009 | 1298.312958 |
| Janus kinase and microtubule-interacting protein 3 || id:prot-a-1599 | rs1714007 | G | A | 4 | 57948114 | -0.1613 | 1.10E-08 | 0.0282 | 0.72835 | -5.719858156 | 32.71677733 |
| Junctional adhesion molecule-like || id:prot-a-86 | rs4938503 | C | G | 11 | 118180155 | 0.1622 | 1.23E-09 | 0.0267 | 0.32533 | 6.074906367 | 36.90448737 |
| Junctional adhesion molecule-like || id:prot-a-86 | rs17121881 | T | A | 11 | 118081345 | 0.9963 | 1.00E-200 | 0.018 | 0.58329 | 55.35 | 3063.6225 |
| Junctional adhesion molecule B || id:prot-a-1600 | rs12038333 | A | G | 1 | 196672454 | -0.1741 | 5.75E-12 | 0.0253 | 0.6091 | -6.881422925 | 47.35398147 |
| Junctophilin-3 || id:prot-a-1603 | rs190079288 | T | G | 4 | 91334832 | 0.7179 | 2.09E-08 | 0.1281 | 0.00988 | 5.604215457 | 31.40723088 |
| Kallikrein-11 || id:prot-a-1656 | rs1048328 | A | G | 19 | 51527364 | -1.1979 | 5.75E-186 | 0.0412 | 0.08261 | -29.07524272 | 845.3697391 |
| Kallikrein-12 || id:prot-a-1657 | rs570618 | G | T | 1 | 196657064 | -0.1458 | 8.91E-09 | 0.0254 | 0.61149 | -5.74015748 | 32.9494079 |
| Kallikrein-12 || id:prot-a-1657 | rs74480769 | G | A | 5 | 40972211 | -0.5356 | 2.45E-13 | 0.0732 | 0.0317 | -7.316939891 | 53.53760936 |
| Kallikrein-12 || id:prot-a-1657 | rs3745540 | G | A | 19 | 51535130 | -0.3011 | 2.51E-34 | 0.0246 | 0.59372 | -12.2398374 | 149.8136195 |
| Kallikrein-14 || id:prot-a-1658 | rs7539005 | T | A | 1 | 196667252 | -0.2069 | 3.16E-16 | 0.0253 | 0.61028 | -8.177865613 | 66.87748598 |
| Kallikrein-14 || id:prot-a-1658 | rs74480769 | G | A | 5 | 40972211 | -0.5488 | 6.17E-14 | 0.0731 | 0.0317 | -7.50752394 | 56.36291571 |
| Kallikrein-14 || id:prot-a-1659 | rs2569491 | A | G | 19 | 51584916 | 0.5749 | 3.63E-119 | 0.0248 | 0.29975 | 23.18145161 | 537.3796989 |
| Kallikrein-14 || id:prot-a-1659 | rs34093024 | C | T | 19 | 51595551 | -0.3431 | 1.58E-18 | 0.0391 | 0.11639 | -8.774936061 | 76.99950288 |
| Kallikrein-14 || id:prot-a-1659 | rs11285439 | CA | C | 19 | 51536476 | -0.1782 | 6.17E-10 | 0.0288 | 0.72319 | -6.1875 | 38.28515625 |
| Kallikrein-15 || id:prot-a-1660 | rs34879232 | G | GA | 17 | 26722039 | 0.1434 | 5.50E-09 | 0.0246 | 0.45209 | 5.829268293 | 33.98036883 |
| Kallikrein-4 || id:prot-a-1662 | rs34436714 | A | C | 19 | 54327313 | -0.1857 | 6.17E-10 | 0.03 | 0.21294 | -6.19 | 38.3161 |
| Kallikrein-5 || id:prot-a-1663 | rs1540206 | A | G | 11 | 132625777 | -0.1461 | 4.57E-08 | 0.0267 | 0.66001 | -5.471910112 | 29.94180028 |
| Kallikrein-6 || id:prot-a-1665 | rs7539005 | T | A | 1 | 196667252 | -0.1838 | 4.57E-13 | 0.0254 | 0.61028 | -7.236220472 | 52.36288673 |
| Kallikrein-6 || id:prot-a-1665 | rs74480769 | G | A | 5 | 40972211 | -0.5078 | 4.07E-12 | 0.0732 | 0.0317 | -6.93715847 | 48.12416764 |
| Kallikrein-6 || id:prot-a-1665 | rs1955054 | G | A | 8 | 123305939 | -0.1818 | 2.63E-08 | 0.0327 | 0.19374 | -5.559633028 | 30.9095194 |
| Kallikrein-7 || id:prot-a-1666 | rs12986064 | C | T | 19 | 54755133 | -0.1702 | 2.24E-11 | 0.0254 | 0.5169 | -6.700787402 | 44.9005518 |
| Kallikrein-7 || id:prot-a-1666 | rs2739433 | C | T | 19 | 51517191 | 0.1463 | 2.88E-08 | 0.0264 | 0.62664 | 5.541666667 | 30.71006944 |
| Kallikrein-7 || id:prot-a-1666 | rs2739419 | G | A | 19 | 51484562 | 0.7031 | 1.12E-66 | 0.0408 | 0.90832 | 17.23284314 | 296.9708826 |
| Kallikrein-8 || id:prot-a-1667 | rs74705037 | A | G | 19 | 51504808 | 0.9287 | 6.31E-58 | 0.0579 | 0.04451 | 16.03972366 | 257.2727351 |
| Kallikrein-8 || id:prot-a-1667 | rs34542531 | C | G | 19 | 51504415 | -1.2026 | 1.41E-30 | 0.1046 | 0.01579 | -11.49713193 | 132.1840426 |
| Kallikrein-8 || id:prot-a-1668 | rs7255673 | A | C | 19 | 51492314 | -0.6073 | 2.82E-09 | 0.1022 | 0.01624 | -5.942270059 | 35.31057345 |
| Kallikrein-8 || id:prot-a-1668 | rs74705037 | A | G | 19 | 51504808 | 0.6557 | 1.12E-28 | 0.059 | 0.04451 | 11.11355932 | 123.5112008 |
| Kallistatin || id:prot-a-2691 | rs10135681 | C | T | 14 | 95007744 | 0.1497 | 3.24E-09 | 0.0253 | 0.37701 | 5.916996047 | 35.01084223 |
| Kallistatin || id:prot-a-2691 | rs10139745 | A | G | 14 | 95035374 | 0.4846 | 4.57E-65 | 0.0284 | 0.22143 | 17.06338028 | 291.1589466 |
| Kallistatin || id:prot-a-2692 | rs10139745 | A | G | 14 | 95035374 | 0.6166 | 4.90E-110 | 0.0277 | 0.22143 | 22.2599278 | 495.5043856 |
| Kallistatin || id:prot-a-2692 | rs10135681 | C | T | 14 | 95007744 | 0.2152 | 1.20E-17 | 0.0252 | 0.37701 | 8.53968254 | 72.92617788 |
| KDEL motif-containing protein 2 || id:prot-a-1621 | rs74911261 | A | G | 11 | 108357137 | -1.3136 | 1.15E-69 | 0.0745 | 0.02654 | -17.63221477 | 310.8949975 |
| Kelch-like protein 13 || id:prot-a-1655 | rs74480769 | G | A | 5 | 40972211 | -0.4638 | 2.51E-10 | 0.0733 | 0.0317 | -6.327421555 | 40.03626354 |
| Kelch-like protein 13 || id:prot-a-1655 | rs28366193 | A | T | 6 | 32369323 | 0.2501 | 2.29E-08 | 0.0448 | 0.08065 | 5.582589286 | 31.16530313 |
| Kelch-like protein 13 || id:prot-a-1655 | rs704 | A | G | 17 | 26694861 | 0.3773 | 1.48E-56 | 0.0238 | 0.46665 | 15.85294118 | 251.3157439 |
| Keratin-associated protein 2-4 || id:prot-a-1687 | rs71640034 | A | G | 4 | 187161048 | -0.1839 | 9.77E-14 | 0.0247 | 0.51294 | -7.44534413 | 55.43314921 |
| Keratin-associated protein 2-4 || id:prot-a-1687 | rs2545801 | C | T | 5 | 176841339 | -0.1585 | 1.62E-08 | 0.0281 | 0.75043 | -5.640569395 | 31.8160231 |
| Keratin, type I cytoskeletal 17 || id:prot-a-1682 | rs186838726 | T | G | 10 | 23878740 | 0.587 | 1.58E-08 | 0.1039 | 0.0164 | 5.649663138 | 31.91869357 |
| Keratinocyte differentiation-associated protein || id:prot-a-1688 | rs3917529 | G | A | 7 | 94940235 | 0.2943 | 1.82E-27 | 0.0271 | 0.28284 | 10.8597786 | 117.9347912 |
| Keratinocyte differentiation-associated protein || id:prot-a-1688 | rs854572 | G | C | 7 | 94954696 | -0.1655 | 1.95E-11 | 0.0247 | 0.54095 | -6.700404858 | 44.89542527 |
| Keratinocyte differentiation-associated protein || id:prot-a-1688 | rs10424405 | G | A | 19 | 54321933 | -0.2482 | 1.48E-16 | 0.03 | 0.2203 | -8.273333333 | 68.44804444 |
| Keratinocyte differentiation-associated protein || id:prot-a-1688 | rs2232613 | T | C | 20 | 36997655 | -0.2761 | 1.95E-09 | 0.046 | 0.0768 | -6.002173913 | 36.02609168 |
| Killer cell immunoglobulin-like receptor 2DL2 || id:prot-a-1640 | rs704 | A | G | 17 | 26694861 | 0.4078 | 1.32E-66 | 0.0237 | 0.46665 | 17.20675105 | 296.0722819 |
| Killer cell immunoglobulin-like receptor 2DL4 || id:prot-a-1642 | rs62143206 | T | G | 19 | 54326212 | -0.17 | 1.32E-08 | 0.0299 | 0.2131 | -5.685618729 | 32.32626033 |
| Killer cell immunoglobulin-like receptor 2DL5A || id:prot-a-1643 | rs429358 | C | T | 19 | 45411941 | 0.3499 | 7.59E-26 | 0.0333 | 0.15301 | 10.50750751 | 110.407714 |
| Killer cell immunoglobulin-like receptor 2DL5A || id:prot-a-1644 | rs12980151 | A | C | 19 | 55374472 | 0.9152 | 1.00E-200 | 0.0228 | 0.26288 | 40.14035088 | 1611.247769 |
| Killer cell immunoglobulin-like receptor 2DL5A || id:prot-a-1644 | rs3765014 | G | C | 19 | 55420924 | -0.2201 | 3.72E-12 | 0.0317 | 0.18112 | -6.943217666 | 48.20827155 |
| Killer cell immunoglobulin-like receptor 2DL5A || id:prot-a-1644 | rs9676587 | G | C | 19 | 55341167 | 0.6259 | 4.90E-145 | 0.0244 | 0.42621 | 25.65163934 | 658.006601 |
| Killer cell immunoglobulin-like receptor 2DS2 || id:prot-a-1645 | rs56393452 | A | C | 6 | 30467120 | -0.1535 | 4.47E-08 | 0.0281 | 0.51477 | -5.462633452 | 29.84036423 |
| Killer cell immunoglobulin-like receptor 2DS2 || id:prot-a-1645 | rs2524139 | C | G | 6 | 31264133 | -0.4291 | 2.00E-52 | 0.0282 | 0.73413 | -15.21631206 | 231.5361526 |
| Killer cell immunoglobulin-like receptor 2DS2 || id:prot-a-1645 | rs9264277 | C | T | 6 | 31224667 | -0.1664 | 6.61E-11 | 0.0255 | 0.62091 | -6.525490196 | 42.5820223 |
| Killer cell immunoglobulin-like receptor 2DS2 || id:prot-a-1645 | rs11066914 | T | G | 12 | 114492128 | -0.1425 | 3.89E-08 | 0.0259 | 0.33866 | -5.501930502 | 30.27123925 |
| Killer cell immunoglobulin-like receptor 2DS2 || id:prot-a-1645 | rs12981397 | C | T | 19 | 55404391 | -0.1744 | 4.68E-10 | 0.028 | 0.28988 | -6.228571429 | 38.79510204 |
| Killer cell immunoglobulin-like receptor 2DS2 || id:prot-a-1645 | rs62132666 | C | T | 19 | 55239639 | -0.2377 | 2.88E-12 | 0.034 | 0.16203 | -6.991176471 | 48.87654844 |
| Killer cell immunoglobulin-like receptor 2DS2 || id:prot-a-1645 | rs11673241 | A | G | 19 | 10894981 | -0.2556 | 4.47E-08 | 0.0467 | 0.07533 | -5.473233405 | 29.9562839 |
| Killer cell immunoglobulin-like receptor 2DS2 || id:prot-a-1645 | rs376891043 | G | C | 19 | 55249060 | 0.6308 | 3.98E-146 | 0.0245 | 0.36418 | 25.74693878 | 662.9048563 |
| Killer cell immunoglobulin-like receptor 3DS1 || id:prot-a-1648 | rs28490042 | A | T | 22 | 46393787 | 0.1473 | 5.62E-09 | 0.0253 | 0.60414 | 5.822134387 | 33.89724882 |
| Killer cell lectin-like receptor subfamily F member 1 || id:prot-a-1672 | rs62143194 | G | C | 19 | 54319624 | -0.2175 | 1.02E-12 | 0.0305 | 0.22364 | -7.131147541 | 50.85326525 |
| Killer cell lectin-like receptor subfamily F member 1 || id:prot-a-1673 | rs11708955 | C | T | 3 | 49540114 | -0.1792 | 7.76E-12 | 0.0262 | 0.31433 | -6.839694656 | 46.78142299 |
| Killer cell lectin-like receptor subfamily F member 1 || id:prot-a-1673 | rs77271561 | T | C | 3 | 36791442 | 0.4267 | 2.00E-08 | 0.076 | 0.02898 | 5.614473684 | 31.52231475 |
| Kin of IRRE-like protein 3 || id:prot-a-1651 | rs62143196 | G | A | 19 | 54320636 | -0.1739 | 8.91E-09 | 0.0302 | 0.22404 | -5.758278146 | 33.1577672 |
| Kinesin-like protein KIF16B || id:prot-a-1635 | rs74480769 | G | A | 5 | 40972211 | -0.5304 | 4.27E-13 | 0.0732 | 0.0317 | -7.245901639 | 52.50309057 |
| Kinesin-like protein KIF16B || id:prot-a-1635 | rs56284011 | C | G | 17 | 43079322 | -0.3615 | 3.39E-08 | 0.0655 | 0.03943 | -5.519083969 | 30.46028786 |
| Kinetochore protein NDC80 homolog || id:prot-a-2018 | rs33944729 | T | TA | 1 | 196652124 | 0.1633 | 1.10E-10 | 0.0253 | 0.38701 | 6.454545455 | 41.66115702 |
| Kinetochore protein NDC80 homolog || id:prot-a-2018 | rs2668196 | T | A | 3 | 165502709 | -0.2326 | 4.79E-13 | 0.0322 | 0.81693 | -7.223602484 | 52.18043285 |
| Kinetochore protein NDC80 homolog || id:prot-a-2018 | rs74480769 | G | A | 5 | 40972211 | -0.4499 | 8.51E-10 | 0.0733 | 0.0317 | -6.137789905 | 37.67246491 |
| Kinetochore protein NDC80 homolog || id:prot-a-2018 | rs72936566 | T | C | 18 | 54038408 | 0.5168 | 4.17E-08 | 0.0942 | 0.0219 | 5.486199575 | 30.09838578 |
| Kit ligand || id:prot-a-1653 | rs6065904 | A | G | 20 | 44534651 | 0.2192 | 3.24E-13 | 0.0301 | 0.20776 | 7.282392027 | 53.03323363 |
| Kremen protein 2 || id:prot-a-1681 | rs74480769 | G | A | 5 | 40972211 | -0.4361 | 2.69E-09 | 0.0733 | 0.0317 | -5.94952251 | 35.3968181 |
| Kunitz-type protease inhibitor 1 || id:prot-a-2823 | rs9439082 | A | G | 1 | 39344055 | -0.3543 | 1.20E-47 | 0.0244 | 0.46027 | -14.5204918 | 210.8446822 |
| Kunitz-type protease inhibitor 1 || id:prot-a-2823 | rs17658212 | T | C | 15 | 41145919 | -0.3421 | 2.29E-11 | 0.0512 | 0.06828 | -6.681640625 | 44.64432144 |
| Kunitz-type protease inhibitor 2 || id:prot-a-2824 | rs1469698 | T | C | 19 | 38993556 | -0.2039 | 4.68E-08 | 0.0373 | 0.12412 | -5.466487936 | 29.88249035 |
| Kunitz-type protease inhibitor 2 || id:prot-a-2824 | rs8112903 | C | T | 19 | 37545386 | -0.4834 | 2.29E-09 | 0.0809 | 0.02484 | -5.975278121 | 35.70394862 |
| Kunitz-type protease inhibitor 2 || id:prot-a-2824 | rs71354995 | G | A | 19 | 38791841 | -0.9908 | 1.00E-200 | 0.0227 | 0.24257 | -43.64757709 | 1905.110986 |
| Kunitz-type protease inhibitor 3 || id:prot-a-2825 | rs6017591 | C | T | 20 | 44141041 | 0.5431 | 6.03E-127 | 0.0227 | 0.54131 | 23.92511013 | 572.4108948 |
| Kv channel-interacting protein 1 || id:prot-a-1616 | rs4795433 | T | C | 17 | 26716821 | 0.2297 | 2.69E-21 | 0.0242 | 0.50963 | 9.491735537 | 90.09304351 |
| Kynureninase || id:prot-a-1689 | rs3816193 | A | G | 2 | 143799907 | -0.5049 | 2.40E-58 | 0.0314 | 0.18044 | -16.07961783 | 258.5541097 |
| Kynurenine--oxoglutarate transaminase 3 || id:prot-a-378 | rs9787133 | G | C | 1 | 89382664 | -0.1716 | 2.88E-12 | 0.0246 | 0.49352 | -6.975609756 | 48.65913147 |
| Kynurenine--oxoglutarate transaminase 3 || id:prot-a-378 | rs10418046 | G | T | 19 | 54327869 | 0.2789 | 4.27E-21 | 0.0296 | 0.21727 | 9.422297297 | 88.77968636 |
| L-lactate dehydrogenase B chain || id:prot-a-1715 | rs10418046 | G | T | 19 | 54327869 | 0.4877 | 1.51E-64 | 0.0288 | 0.21727 | 16.93402778 | 286.7612968 |
| L-Selectin || id:prot-a-2666 | rs3886553 | C | G | 1 | 169572549 | -0.1886 | 3.80E-12 | 0.0272 | 0.31908 | -6.933823529 | 48.07790874 |
| L-Selectin || id:prot-a-2666 | rs4987358 | T | G | 1 | 169665551 | -0.5165 | 6.76E-87 | 0.0261 | 0.27377 | -19.78927203 | 391.6152875 |
| L-Selectin || id:prot-a-2666 | rs75385614 | G | A | 12 | 24576215 | -0.2504 | 1.70E-08 | 0.0444 | 0.10387 | -5.63963964 | 31.80553526 |
| L-Selectin || id:prot-a-2666 | rs632650 | T | G | 12 | 112131698 | -0.1887 | 4.17E-08 | 0.0344 | 0.15501 | -5.485465116 | 30.09032754 |
| Lactadherin || id:prot-a-1888 | rs1961839 | A | G | 15 | 89467454 | -0.2253 | 3.39E-19 | 0.0252 | 0.39742 | -8.94047619 | 79.93211451 |
| Lactase-phlorizin hydrolase || id:prot-a-1713 | rs72721827 | T | C | 1 | 98887921 | -0.2487 | 3.16E-08 | 0.045 | 0.08102 | -5.526666667 | 30.54404444 |
| Lactase-phlorizin hydrolase || id:prot-a-1713 | rs4988235 | A | G | 2 | 136608646 | 0.7759 | 1.00E-200 | 0.0244 | 0.71122 | 31.79918033 | 1011.18787 |
| Lactase-phlorizin hydrolase || id:prot-a-1713 | rs34272309 | G | GT | 2 | 135281899 | -0.2181 | 5.62E-16 | 0.0269 | 0.31746 | -8.107806691 | 65.73652935 |
| Lactase-phlorizin hydrolase || id:prot-a-1713 | rs115478735 | T | A | 9 | 136149711 | -0.1894 | 3.72E-09 | 0.0321 | 0.17824 | -5.900311526 | 34.81367611 |
| Lactase-phlorizin hydrolase || id:prot-a-1713 | rs679574 | G | C | 19 | 49206108 | -0.1406 | 8.13E-09 | 0.0244 | 0.50693 | -5.762295082 | 33.20404461 |
| Lactoperoxidase || id:prot-a-1765 | rs2142887 | G | C | 6 | 119509785 | -0.1346 | 2.75E-08 | 0.0242 | 0.47237 | -5.561983471 | 30.93566013 |
| Lactoperoxidase || id:prot-a-1765 | rs7219860 | A | G | 17 | 56321271 | -0.2621 | 4.79E-18 | 0.0303 | 0.20596 | -8.650165017 | 74.82535481 |
| Lactoperoxidase || id:prot-a-1765 | rs7250982 | G | A | 19 | 5823244 | 0.4818 | 3.31E-14 | 0.0635 | 0.03974 | 7.587401575 | 57.56866266 |
| Lactotransferrin || id:prot-a-1808 | rs146097568 | C | T | 3 | 46464792 | -0.9052 | 3.09E-21 | 0.0957 | 0.01734 | -9.458725183 | 89.46748208 |
| Lactotransferrin || id:prot-a-1808 | rs6441996 | C | G | 3 | 46505266 | -0.4304 | 2.00E-51 | 0.0285 | 0.23776 | -15.10175439 | 228.0629855 |
| Lactotransferrin || id:prot-a-1809 | rs61740470 | T | C | 3 | 46491985 | -1.2313 | 1.86E-26 | 0.1157 | 0.01144 | -10.64217805 | 113.2559536 |
| Lactotransferrin || id:prot-a-1809 | rs73406435 | T | G | 12 | 115254617 | -0.2574 | 1.70E-08 | 0.0456 | 0.08116 | -5.644736842 | 31.86305402 |
| Lamin-B1 || id:prot-a-1758 | rs36105360 | T | C | 5 | 126161690 | 0.8039 | 2.19E-24 | 0.0789 | 0.02519 | 10.18884664 | 103.8125959 |
| Lamina-associated polypeptide 2, isoforms beta/gamma || id:prot-a-3021 | rs4632248 | T | G | 19 | 54324995 | 0.6209 | 1.95E-108 | 0.0281 | 0.21439 | 22.09608541 | 488.2369904 |
| Laminin || id:prot-a-1695 | rs4129858 | G | A | 1 | 183004334 | -0.2937 | 7.08E-34 | 0.0242 | 0.43088 | -12.13636364 | 147.2913223 |
| Laminin || id:prot-a-1695 | rs4997370 | T | G | 1 | 183100563 | -0.2273 | 4.37E-19 | 0.0255 | 0.5836 | -8.91372549 | 79.45450211 |
| Laminin || id:prot-a-1695 | rs507666 | A | G | 9 | 136149399 | -0.1793 | 2.14E-08 | 0.032 | 0.17941 | -5.603125 | 31.39500977 |
| Laminin || id:prot-a-1695 | rs62199218 | A | G | 20 | 60932425 | 0.7524 | 1.10E-31 | 0.0642 | 0.03903 | 11.71962617 | 137.3496375 |
| Laminin subunit alpha-4 || id:prot-a-1696 | rs11207023 | C | T | 1 | 57850250 | 0.1708 | 5.50E-09 | 0.0293 | 0.22988 | 5.829351536 | 33.98133933 |
| Laminin subunit alpha-4 || id:prot-a-1696 | rs4241818 | C | T | 4 | 187153786 | -0.3122 | 4.79E-38 | 0.0242 | 0.51359 | -12.90082645 | 166.431323 |
| Laminin subunit alpha-4 || id:prot-a-1696 | rs75077631 | G | GC | 5 | 176840084 | 0.2101 | 4.47E-14 | 0.0278 | 0.25227 | 7.557553957 | 57.11662181 |
| Laminin subunit gamma-2 || id:prot-a-1697 | rs2276543 | A | G | 1 | 183155305 | 0.6155 | 4.37E-133 | 0.0251 | 0.27901 | 24.52191235 | 601.3241853 |
| Laminin subunit gamma-2 || id:prot-a-1697 | rs183698890 | C | G | 3 | 110959732 | -0.517 | 2.75E-08 | 0.093 | 0.01878 | -5.559139785 | 30.90403515 |
| Layilin || id:prot-a-1703 | rs7539005 | T | A | 1 | 196667252 | -0.1427 | 2.14E-08 | 0.0255 | 0.61028 | -5.596078431 | 31.31609381 |
| Layilin || id:prot-a-1703 | rs74480769 | G | A | 5 | 40972211 | -0.4525 | 6.76E-10 | 0.0733 | 0.0317 | -6.173260573 | 38.1091461 |
| Leiomodin-1 || id:prot-a-1759 | rs116937000 | A | G | 14 | 26843597 | 0.4199 | 3.39E-10 | 0.0669 | 0.04526 | 6.276532138 | 39.39485567 |
| LEM domain-containing protein 1 || id:prot-a-1720 | rs4632248 | T | G | 19 | 54324995 | 0.5131 | 2.04E-71 | 0.0287 | 0.21439 | 17.87804878 | 319.6246282 |
| Leptin receptor || id:prot-a-1724 | rs150117458 | G | GT | 1 | 66080708 | 1.3753 | 1.00E-200 | 0.0219 | 0.82422 | 62.79908676 | 3943.725298 |
| Leptin receptor || id:prot-a-1724 | rs78007650 | A | G | 1 | 65701309 | -0.3908 | 4.79E-11 | 0.0594 | 0.05089 | -6.579124579 | 43.28488023 |
| Leucine-rich PPR motif-containing protein, mitochondrial || id:prot-a-1783 | rs2228243 | G | A | 3 | 186395113 | 0.7499 | 2.14E-165 | 0.0274 | 0.20387 | 27.36861314 | 749.0409851 |
| Leucine-rich PPR motif-containing protein, mitochondrial || id:prot-a-1783 | rs200123836 | T | C | 14 | 106200601 | 0.2895 | 2.82E-24 | 0.0285 | 0.26737 | 10.15789474 | 103.1828255 |
| Leucine-rich repeat-containing protein 15 || id:prot-a-1784 | rs73081778 | T | C | 3 | 194080033 | -0.5026 | 1.32E-10 | 0.0782 | 0.02548 | -6.427109974 | 41.30774262 |
| Leucine-rich repeat-containing protein 15 || id:prot-a-1784 | rs57514363 | G | T | 3 | 194087927 | 0.5927 | 5.89E-54 | 0.0383 | 0.11288 | 15.47519582 | 239.4816857 |
| Leucine-rich repeat-containing protein 19 || id:prot-a-1785 | rs7519758 | T | C | 1 | 196825287 | -0.9699 | 1.00E-200 | 0.0259 | 0.19402 | -37.44787645 | 1402.34345 |
| Leucine-rich repeat-containing protein 19 || id:prot-a-1785 | rs34408013 | A | G | 1 | 196745281 | -0.21 | 2.14E-09 | 0.0351 | 0.20282 | -5.982905983 | 35.795164 |
| Leucine-rich repeat-containing protein 37A2 || id:prot-a-1787 | rs11080055 | C | A | 17 | 26649724 | -0.1425 | 4.47E-09 | 0.0243 | 0.50786 | -5.864197531 | 34.38881268 |
| Leucine-rich repeat-containing protein 74A || id:prot-a-1790 | rs33944729 | T | TA | 1 | 196652124 | 0.1942 | 1.45E-14 | 0.0253 | 0.38701 | 7.675889328 | 58.91927698 |
| Leucine-rich repeat-containing protein 74A || id:prot-a-1790 | rs74480769 | G | A | 5 | 40972211 | -0.4847 | 3.72E-11 | 0.0733 | 0.0317 | -6.61255116 | 43.72583284 |
| Leucine-rich repeat and fibronectin type III domain-containing protein 1 || id:prot-a-1768 | rs2304200 | A | G | 19 | 15580658 | 0.2858 | 9.55E-11 | 0.0442 | 0.0859 | 6.466063348 | 41.80997523 |
| Leucine-rich repeat and transmembrane domain-containing protein 2 || id:prot-a-1800 | rs10922098 | T | C | 1 | 196664651 | -0.1591 | 3.39E-10 | 0.0253 | 0.60979 | -6.288537549 | 39.54570451 |
| Leucine-rich repeat and transmembrane domain-containing protein 2 || id:prot-a-1800 | rs2668196 | T | A | 3 | 165502709 | -0.2055 | 1.78E-10 | 0.0322 | 0.81693 | -6.381987578 | 40.72976544 |
| Leucine-rich repeat and transmembrane domain-containing protein 2 || id:prot-a-1800 | rs5167 | G | T | 19 | 45448465 | 0.1872 | 3.80E-13 | 0.0258 | 0.34696 | 7.255813953 | 52.64683613 |
| Leucine-rich repeat neuronal protein 1 || id:prot-a-1792 | rs429358 | C | T | 19 | 45411941 | 1.1769 | 1.00E-200 | 0.0269 | 0.15301 | 43.75092937 | 1914.143821 |
| Leucine-rich repeat neuronal protein 1 || id:prot-a-1792 | rs199956232 | CT | C | 19 | 45356752 | 0.2304 | 7.94E-17 | 0.0277 | 0.28034 | 8.317689531 | 69.18395913 |
| Leucine-rich repeat neuronal protein 1 || id:prot-a-1793 | rs6801789 | C | T | 3 | 3807592 | 0.246 | 2.29E-22 | 0.0253 | 0.35403 | 9.723320158 | 94.5429549 |
| Leucine-rich repeat neuronal protein 3 || id:prot-a-1794 | rs59753727 | A | C | 10 | 84019699 | 0.3911 | 3.02E-08 | 0.0706 | 0.0409 | 5.539660057 | 30.68783354 |
| Leucine-rich repeat serine/threonine-protein kinase 2 || id:prot-a-1791 | rs74480769 | G | A | 5 | 40972211 | -0.4602 | 3.47E-10 | 0.0733 | 0.0317 | -6.278308322 | 39.41715539 |
| Leucine-rich repeat serine/threonine-protein kinase 2 || id:prot-a-1791 | rs72710445 | G | A | 9 | 27633760 | -0.2288 | 1.70E-08 | 0.0406 | 0.10562 | -5.63546798 | 31.75849936 |
| Leucine-rich repeat serine/threonine-protein kinase 2 || id:prot-a-1791 | rs704 | A | G | 17 | 26694861 | 0.3035 | 2.63E-36 | 0.0241 | 0.46665 | 12.593361 | 158.5927412 |
| Leucine-rich repeat serine/threonine-protein kinase 2 || id:prot-a-1791 | rs4632248 | T | G | 19 | 54324995 | 0.2055 | 5.89E-12 | 0.0299 | 0.21439 | 6.872909699 | 47.23688773 |
| Leucine-rich repeat transmembrane protein FLRT2 || id:prot-a-1123 | rs17796777 | C | A | 14 | 85806774 | -0.2353 | 1.51E-17 | 0.0276 | 0.28551 | -8.525362319 | 72.68180267 |
| Leucine-rich repeat transmembrane protein FLRT3 || id:prot-a-1124 | rs7872945 | T | C | 9 | 120162325 | 0.146 | 1.00E-08 | 0.0255 | 0.41051 | 5.725490196 | 32.78123799 |
| Leucine-rich repeat transmembrane protein FLRT3 || id:prot-a-1124 | rs11908097 | C | T | 20 | 14689146 | 0.4444 | 2.45E-57 | 0.0278 | 0.25127 | 15.98561151 | 255.5397754 |
| Leucine-rich repeat, immunoglobulin-like domain and transmembrane domain-containing protein 2 || id:prot-a-1771 | rs17580 | A | T | 14 | 94847262 | 0.339 | 2.34E-09 | 0.0568 | 0.04977 | 5.968309859 | 35.62072257 |
| Leucine-rich repeat, immunoglobulin-like domain and transmembrane domain-containing protein 3 || id:prot-a-1773 | rs7528639 | G | A | 1 | 161624045 | 0.1852 | 7.59E-10 | 0.0301 | 0.29462 | 6.15282392 | 37.85724219 |
| Leucine-rich repeats and immunoglobulin-like domains protein 3 || id:prot-a-1770 | rs11172791 | C | T | 12 | 59272973 | -0.4624 | 4.79E-14 | 0.0613 | 0.04525 | -7.543230016 | 56.90031908 |
| Leukemia inhibitory factor || id:prot-a-1736 | rs4795433 | T | C | 17 | 26716821 | -0.2008 | 1.38E-16 | 0.0243 | 0.50963 | -8.263374486 | 68.28335789 |
| Leukemia inhibitory factor receptor || id:prot-a-1737 | rs635634 | T | C | 9 | 136155000 | -0.2996 | 6.03E-21 | 0.0319 | 0.17981 | -9.39184953 | 88.20683759 |
| Leukocyte-specific transcript 1 protein || id:prot-a-1802 | rs10107851 | C | T | 8 | 11703002 | 0.4843 | 9.55E-10 | 0.0792 | 0.02576 | 6.11489899 | 37.39198966 |
| Leukocyte cell-derived chemotaxin 1 || id:prot-a-1719 | rs10800456 | G | A | 1 | 169520098 | -0.1568 | 1.12E-08 | 0.0274 | 0.29939 | -5.722627737 | 32.74846822 |
| Leukocyte cell-derived chemotaxin 1 || id:prot-a-1719 | rs17080528 | T | C | 3 | 49389842 | 0.1634 | 5.37E-10 | 0.0263 | 0.31216 | 6.212927757 | 38.60047131 |
| Leukocyte immunoglobulin-like receptor subfamily A member 4 || id:prot-a-1738 | rs876036 | C | T | 7 | 50307710 | -0.1982 | 6.03E-14 | 0.0264 | 0.31725 | -7.507575758 | 56.36369376 |
| Leukocyte immunoglobulin-like receptor subfamily A member 4 || id:prot-a-1738 | rs73061013 | G | A | 19 | 54859176 | 0.272 | 1.58E-08 | 0.0481 | 0.0704 | 5.654885655 | 31.97773177 |
| Leukocyte immunoglobulin-like receptor subfamily A member 4 || id:prot-a-1738 | rs148933445 | A | G | 19 | 45302504 | -0.5136 | 3.47E-09 | 0.0869 | 0.02431 | -5.910241657 | 34.93095645 |
| Leukocyte immunoglobulin-like receptor subfamily A member 4 || id:prot-a-1738 | rs2241384 | A | G | 19 | 54849942 | -0.3088 | 3.39E-21 | 0.0327 | 0.17042 | -9.443425076 | 89.17827717 |
| Leukocyte immunoglobulin-like receptor subfamily A member 5 || id:prot-a-1739 | rs1092801 | C | G | 1 | 196729035 | 0.2952 | 8.32E-18 | 0.0343 | 0.16915 | 8.606413994 | 74.07036184 |
| Leukocyte immunoglobulin-like receptor subfamily A member 5 || id:prot-a-1739 | rs759819 | C | T | 19 | 54815577 | -0.5429 | 2.51E-111 | 0.0242 | 0.3305 | -22.4338843 | 503.2791647 |
| Leukocyte immunoglobulin-like receptor subfamily A member 5 || id:prot-a-1739 | rs417902 | T | A | 19 | 54767800 | -0.1738 | 8.13E-12 | 0.0254 | 0.59055 | -6.842519685 | 46.82007564 |
| Leukocyte immunoglobulin-like receptor subfamily A member 5 || id:prot-a-1740 | rs759819 | C | T | 19 | 54815577 | -0.2488 | 2.82E-22 | 0.0256 | 0.3305 | -9.71875 | 94.45410156 |
| Leukocyte immunoglobulin-like receptor subfamily A member 6 || id:prot-a-1741 | rs7080536 | A | G | 10 | 115348046 | -0.3838 | 7.24E-10 | 0.0623 | 0.04384 | -6.160513644 | 37.95192835 |
| Leukocyte immunoglobulin-like receptor subfamily A member 6 || id:prot-a-1741 | rs117376661 | G | C | 19 | 54742900 | -0.4119 | 1.29E-18 | 0.0468 | 0.07914 | -8.801282051 | 77.46256575 |
| Leukocyte immunoglobulin-like receptor subfamily A member 6 || id:prot-a-1741 | rs35361042 | G | C | 19 | 54748737 | 1.3399 | 1.00E-200 | 0.0376 | 0.09112 | 35.6356383 | 1269.898717 |
| Leukocyte immunoglobulin-like receptor subfamily A member 6 || id:prot-a-1741 | rs1132604 | A | G | 19 | 54744195 | 1.0924 | 5.37E-44 | 0.0785 | 0.03161 | 13.91592357 | 193.6529287 |
| Leukocyte immunoglobulin-like receptor subfamily B member 1 || id:prot-a-1742 | rs141232332 | G | GTTT | 6 | 31075601 | 0.2096 | 2.14E-17 | 0.0247 | 0.43881 | 8.48582996 | 72.0093101 |
| Leukocyte immunoglobulin-like receptor subfamily B member 1 || id:prot-a-1742 | rs9265984 | T | C | 6 | 31316151 | 0.1901 | 4.27E-13 | 0.0262 | 0.31455 | 7.255725191 | 52.64554804 |
| Leukocyte immunoglobulin-like receptor subfamily B member 1 || id:prot-a-1742 | rs2114511 | C | G | 19 | 55145093 | -1.8056 | 1.00E-200 | 0.046 | 0.05123 | -39.25217391 | 1540.733157 |
| Leukocyte immunoglobulin-like receptor subfamily B member 1 || id:prot-a-1742 | rs73614672 | G | A | 19 | 55158865 | 0.7448 | 1.23E-55 | 0.0474 | 0.06723 | 15.71308017 | 246.9008884 |
| Leukocyte immunoglobulin-like receptor subfamily B member 2 || id:prot-a-1743 | rs386056 | T | C | 19 | 54782919 | -1.1495 | 1.00E-200 | 0.0233 | 0.20112 | -49.33476395 | 2433.918934 |
| Leukocyte immunoglobulin-like receptor subfamily B member 2 || id:prot-a-1743 | rs7245916 | G | T | 19 | 54801469 | -0.2488 | 3.72E-11 | 0.0376 | 0.16612 | -6.617021277 | 43.78497057 |
| Leukocyte immunoglobulin-like receptor subfamily B member 2 || id:prot-a-1744 | rs145212847 | T | C | 1 | 61273443 | 0.4549 | 2.63E-08 | 0.0818 | 0.02756 | 5.561124694 | 30.92610787 |
| Leukocyte immunoglobulin-like receptor subfamily B member 2 || id:prot-a-1744 | rs437236 | G | C | 19 | 54786341 | -0.5918 | 1.17E-11 | 0.0872 | 0.0261 | -6.786697248 | 46.05925953 |
| Leukocyte immunoglobulin-like receptor subfamily B member 2 || id:prot-a-1744 | rs373032 | A | T | 19 | 54783375 | -0.7172 | 7.59E-146 | 0.0279 | 0.79202 | -25.70609319 | 660.8032271 |
| Leukocyte immunoglobulin-like receptor subfamily B member 3 || id:prot-a-1745 | rs56250107 | A | G | 8 | 54394562 | -0.1921 | 3.80E-08 | 0.0349 | 0.14983 | -5.504297994 | 30.29729641 |
| Leukocyte immunoglobulin-like receptor subfamily B member 4 || id:prot-a-1746 | rs6993770 | T | A | 8 | 106581528 | -0.1523 | 2.75E-08 | 0.0274 | 0.28225 | -5.558394161 | 30.89574564 |
| Leukocyte immunoglobulin-like receptor subfamily B member 4 || id:prot-a-1746 | rs11592462 | G | C | 10 | 73550117 | -0.3224 | 2.88E-41 | 0.024 | 0.48722 | -13.43333333 | 180.4544444 |
| Leukocyte immunoglobulin-like receptor subfamily B member 4 || id:prot-a-1746 | rs3740129 | A | G | 10 | 73767859 | 0.1576 | 3.02E-10 | 0.025 | 0.45966 | 6.304 | 39.740416 |
| Leukocyte immunoglobulin-like receptor subfamily B member 4 || id:prot-a-1746 | rs11111026 | T | G | 12 | 102192803 | -0.2361 | 1.48E-13 | 0.032 | 0.18176 | -7.378125 | 54.43672852 |
| Leukocyte immunoglobulin-like receptor subfamily B member 5 || id:prot-a-1747 | rs11668526 | T | C | 19 | 54749060 | -0.1678 | 1.00E-11 | 0.0247 | 0.5951 | -6.793522267 | 46.1519448 |
| Leukocyte immunoglobulin-like receptor subfamily B member 5 || id:prot-a-1747 | rs12975366 | C | T | 19 | 54759361 | -1.1607 | 1.00E-200 | 0.0152 | 0.40526 | -76.36184211 | 5831.13093 |
| Ligand-dependent nuclear receptor corepressor-like protein || id:prot-a-1711 | rs7623886 | T | C | 3 | 153137136 | 0.1416 | 2.82E-08 | 0.0255 | 0.58743 | 5.552941176 | 30.83515571 |
| Ligand-dependent nuclear receptor corepressor-like protein || id:prot-a-1711 | rs62143197 | A | G | 19 | 54320716 | -0.1711 | 1.66E-08 | 0.0303 | 0.22311 | -5.646864686 | 31.88708079 |
| LIM and cysteine-rich domains protein 1 || id:prot-a-1757 | rs10418046 | G | T | 19 | 54327869 | -0.1761 | 3.55E-09 | 0.0298 | 0.21727 | -5.909395973 | 34.92096077 |
| Limbic system-associated membrane protein || id:prot-a-1801 | rs1920189 | T | G | 3 | 116122953 | 0.2248 | 1.05E-11 | 0.0331 | 0.83519 | 6.791540785 | 46.12502624 |
| Lipase member N || id:prot-a-1753 | rs142109544 | A | T | 10 | 90670602 | -0.7636 | 2.75E-08 | 0.1375 | 0.00939 | -5.553454545 | 30.84085739 |
| Lipase member N || id:prot-a-1753 | rs1441733 | G | A | 10 | 90596420 | -0.3908 | 8.13E-11 | 0.0601 | 0.04514 | -6.50249584 | 42.28245215 |
| Lipase member N || id:prot-a-1753 | rs10509554 | T | C | 10 | 90525792 | 1.0165 | 1.00E-200 | 0.0213 | 0.30679 | 47.72300469 | 2277.485177 |
| Lipase member N || id:prot-a-1753 | rs4934407 | T | C | 10 | 90212431 | -0.3675 | 8.71E-13 | 0.0514 | 0.06275 | -7.149805447 | 51.11971794 |
| Lon protease homolog, mitochondrial || id:prot-a-1761 | rs62143197 | A | G | 19 | 54320716 | 0.3533 | 2.57E-32 | 0.0299 | 0.22311 | 11.81605351 | 139.6191206 |
| Low-density lipoprotein receptor-related protein 1, soluble || id:prot-a-1776 | rs62143194 | G | C | 19 | 54319624 | 0.2222 | 3.16E-13 | 0.0305 | 0.22364 | 7.285245902 | 53.07480785 |
| Low-density lipoprotein receptor-related protein 1B || id:prot-a-1778 | rs13276307 | T | C | 8 | 144646352 | 0.1645 | 8.13E-09 | 0.0285 | 0.25219 | 5.771929825 | 33.3151739 |
| Low-density lipoprotein receptor-related protein 1B || id:prot-a-1778 | rs62143194 | G | C | 19 | 54319624 | 0.7083 | 1.58E-139 | 0.0282 | 0.22364 | 25.11702128 | 630.8647578 |
| Low-density lipoprotein receptor-related protein 1B || id:prot-a-1779 | rs597331 | A | G | 2 | 21262427 | 0.1467 | 3.09E-09 | 0.0248 | 0.46301 | 5.915322581 | 34.99104123 |
| Low-density lipoprotein receptor-related protein 1B || id:prot-a-1779 | rs964184 | C | G | 11 | 116648917 | -0.2104 | 7.59E-09 | 0.0364 | 0.86898 | -5.78021978 | 33.41094071 |
| Low-density lipoprotein receptor-related protein 1B || id:prot-a-1779 | rs1065853 | T | G | 19 | 45413233 | -0.3734 | 5.13E-16 | 0.046 | 0.07778 | -8.117391304 | 65.89204159 |
| Low affinity immunoglobulin epsilon Fc receptor || id:prot-a-1072 | rs3104415 | C | A | 6 | 32582577 | -0.1417 | 2.24E-08 | 0.0253 | 0.34156 | -5.600790514 | 31.36885438 |
| Low affinity immunoglobulin epsilon Fc receptor || id:prot-a-1072 | rs12973524 | A | G | 19 | 7758263 | -0.3062 | 5.75E-36 | 0.0245 | 0.48403 | -12.49795918 | 156.1989838 |
| Low affinity immunoglobulin epsilon Fc receptor || id:prot-a-1072 | rs12980031 | T | G | 19 | 7764436 | -0.2932 | 7.24E-22 | 0.0305 | 0.75076 | -9.613114754 | 92.41197528 |
| Low affinity immunoglobulin gamma Fc region receptor II-a || id:prot-a-1074 | rs9651051 | G | T | 1 | 161318682 | -0.5275 | 3.72E-12 | 0.0759 | 0.03084 | -6.949934124 | 48.30158433 |
| Low affinity immunoglobulin gamma Fc region receptor II-a || id:prot-a-1074 | rs115264949 | A | G | 1 | 161501932 | 0.5929 | 2.45E-09 | 0.0994 | 0.01611 | 5.964788732 | 35.57870462 |
| Low affinity immunoglobulin gamma Fc region receptor II-a || id:prot-a-1074 | rs111322113 | T | C | 1 | 161589768 | -0.2255 | 1.86E-16 | 0.0274 | 0.32894 | -8.229927007 | 67.73169855 |
| Low affinity immunoglobulin gamma Fc region receptor II-a || id:prot-a-1074 | rs1801274 | G | A | 1 | 161479745 | 1.2389 | 1.00E-200 | 0.0126 | 0.53257 | 98.32539683 | 9667.883661 |
| Low affinity immunoglobulin gamma Fc region receptor II-b || id:prot-a-1075 | rs6665610 | A | G | 1 | 161641384 | 1.3308 | 1.00E-200 | 0.0224 | 0.20453 | 59.41071429 | 3529.632972 |
| Low affinity immunoglobulin gamma Fc region receptor II-b || id:prot-a-1075 | rs115438555 | A | T | 1 | 161466549 | -0.4025 | 1.00E-08 | 0.0702 | 0.03117 | -5.733618234 | 32.87437805 |
| Low affinity immunoglobulin gamma Fc region receptor II-b || id:prot-a-1075 | rs11590749 | T | G | 1 | 161481567 | -0.4947 | 3.39E-39 | 0.0378 | 0.13067 | -13.08730159 | 171.2774628 |
| Low affinity immunoglobulin gamma Fc region receptor II-b || id:prot-a-1075 | rs112252354 | C | G | 1 | 161375210 | -0.377 | 9.55E-10 | 0.0616 | 0.04282 | -6.12012987 | 37.45598963 |
| Low affinity immunoglobulin gamma Fc region receptor III-B || id:prot-a-1076 | rs10919543 | G | A | 1 | 161508617 | 0.4353 | 3.16E-67 | 0.0251 | 0.32443 | 17.34262948 | 300.7667974 |
| Low affinity immunoglobulin gamma Fc region receptor III-B || id:prot-a-1076 | rs10897054 | T | C | 11 | 60118636 | -0.1631 | 2.57E-09 | 0.0274 | 0.29041 | -5.952554745 | 35.43290799 |
| Low affinity immunoglobulin gamma Fc region receptor III-B || id:prot-a-1076 | rs10774624 | A | G | 12 | 111833788 | -0.1477 | 3.24E-09 | 0.025 | 0.50985 | -5.908 | 34.904464 |
| Low molecular weight phosphotyrosine protein phosphatase || id:prot-a-18 | rs6754120 | G | A | 2 | 340363 | -0.2639 | 1.10E-08 | 0.0462 | 0.084 | -5.712121212 | 32.62832874 |
| Low molecular weight phosphotyrosine protein phosphatase || id:prot-a-18 | rs79716074 | G | A | 2 | 277003 | 1.1572 | 1.00E-200 | 0.0163 | 0.33713 | 70.99386503 | 5040.128872 |
| Luteinizing hormone || id:prot-a-529 | rs139643250 | T | C | 19 | 49517146 | -0.6763 | 2.75E-50 | 0.0454 | 0.07635 | -14.89647577 | 221.9049904 |
| Lutropin subunit beta || id:prot-a-1735 | rs75287599 | T | C | 19 | 49517140 | -0.4342 | 2.40E-21 | 0.0458 | 0.07782 | -9.480349345 | 89.8770237 |
| Lymphocyte activation gene 3 protein || id:prot-a-1693 | rs10774624 | A | G | 12 | 111833788 | -0.1439 | 8.13E-09 | 0.025 | 0.50985 | -5.756 | 33.131536 |
| Lymphocyte antigen 6 complex locus protein G6c || id:prot-a-1810 | rs62143198 | A | G | 19 | 54320939 | 0.5468 | 4.17E-78 | 0.0292 | 0.21453 | 18.7260274 | 350.6641021 |
| Lymphocyte function-associated antigen 3 || id:prot-a-454 | rs704 | A | G | 17 | 26694861 | 0.1524 | 5.50E-10 | 0.0246 | 0.46665 | 6.195121951 | 38.37953599 |
| Lymphotactin || id:prot-a-3236 | rs4656599 | T | C | 1 | 168503386 | 0.4295 | 2.04E-43 | 0.0311 | 0.18989 | 13.81028939 | 190.724093 |
| Lymphotactin || id:prot-a-3237 | rs62143196 | G | A | 19 | 54320636 | -0.2238 | 1.17E-13 | 0.0302 | 0.22404 | -7.410596026 | 54.91693347 |
| Lymphotoxin alpha2:beta1 || id:prot-a-1805 | rs11709130 | A | G | 3 | 8730717 | 0.4361 | 3.47E-08 | 0.079 | 0.03491 | 5.520253165 | 30.473195 |
| LysM and putative peptidoglycan-binding domain-containing protein 3 || id:prot-a-1819 | rs4632248 | T | G | 19 | 54324995 | -0.1769 | 3.39E-09 | 0.0299 | 0.21439 | -5.91638796 | 35.00364649 |
| LysM and putative peptidoglycan-binding domain-containing protein 4 || id:prot-a-1820 | rs2817502 | T | C | 1 | 245929440 | -0.1853 | 4.90E-08 | 0.034 | 0.16003 | -5.45 | 29.7025 |
| Lysophosphatidylcholine acyltransferase 2 || id:prot-a-1764 | rs13276307 | T | C | 8 | 144646352 | 0.1726 | 1.45E-09 | 0.0285 | 0.25219 | 6.056140351 | 36.67683595 |
| Lysophosphatidylcholine acyltransferase 2 || id:prot-a-1764 | rs62143194 | G | C | 19 | 54319624 | 0.5225 | 8.91E-71 | 0.0294 | 0.22364 | 17.77210884 | 315.8478527 |
| Lysosomal acid phosphatase || id:prot-a-19 | rs111335641 | G | A | 4 | 38971884 | 0.5168 | 3.89E-08 | 0.0941 | 0.02102 | 5.492029756 | 30.16239084 |
| Lysosomal acid phosphatase || id:prot-a-19 | rs28929474 | T | C | 14 | 94844947 | 1.1964 | 6.17E-49 | 0.0814 | 0.02269 | 14.6977887 | 216.0249926 |
| Lysosomal acid phosphatase || id:prot-a-19 | rs10139058 | T | C | 14 | 107141916 | -0.2625 | 1.23E-25 | 0.0251 | 0.38314 | -10.45816733 | 109.3732639 |
| Lysosomal Pro-X carboxypeptidase || id:prot-a-2356 | rs4455806 | C | T | 8 | 55457873 | 0.1991 | 1.70E-10 | 0.0312 | 0.19177 | 6.381410256 | 40.72239686 |
| Lysosomal Pro-X carboxypeptidase || id:prot-a-2356 | rs2229437 | G | T | 11 | 82564294 | 0.291 | 2.75E-20 | 0.0315 | 0.18088 | 9.238095238 | 85.34240363 |
| Lysosomal Pro-X carboxypeptidase || id:prot-a-2356 | rs10860794 | A | C | 12 | 102217720 | -0.2229 | 1.48E-16 | 0.027 | 0.294 | -8.255555556 | 68.15419753 |
| Lysosomal protective protein || id:prot-a-717 | rs77430073 | T | C | 8 | 85510894 | 0.4132 | 2.45E-08 | 0.0741 | 0.02857 | 5.576248313 | 31.09454525 |
| Lysosomal protective protein || id:prot-a-717 | rs6993770 | T | A | 8 | 106581528 | -0.2596 | 1.12E-21 | 0.0271 | 0.28225 | -9.579335793 | 91.76367424 |
| Lysosomal protective protein || id:prot-a-717 | rs7897379 | C | T | 10 | 65301725 | 0.1707 | 8.51E-12 | 0.025 | 0.47974 | 6.828 | 46.621584 |
| Lysosomal protective protein || id:prot-a-717 | rs7964859 | G | C | 12 | 102220783 | -0.2566 | 2.00E-21 | 0.027 | 0.2939 | -9.503703704 | 90.32038409 |
| Lysosomal protective protein || id:prot-a-717 | rs34242148 | CT | C | 15 | 42778158 | 0.1757 | 1.51E-08 | 0.031 | 0.20136 | 5.667741935 | 32.12329865 |
| Lysozyme-like protein 2 || id:prot-a-1822 | rs704 | A | G | 17 | 26694861 | 0.1999 | 2.95E-16 | 0.0244 | 0.46665 | 8.192622951 | 67.11907081 |
| Lysozyme-like protein 2 || id:prot-a-1822 | rs4632248 | T | G | 19 | 54324995 | 0.3881 | 4.90E-40 | 0.0293 | 0.21439 | 13.24573379 | 175.4494636 |
| Lysozyme C || id:prot-a-1821 | rs4761234 | C | T | 12 | 69732105 | -0.4735 | 3.80E-89 | 0.0237 | 0.47308 | -19.97890295 | 399.1565632 |
| Lysozyme g-like protein 1 || id:prot-a-1814 | rs32896 | G | T | 5 | 76246859 | 0.2375 | 1.41E-08 | 0.0419 | 0.10391 | 5.668257757 | 32.12914599 |
| Lysozyme g-like protein 1 || id:prot-a-1814 | rs112226238 | G | A | 14 | 106530826 | 0.2083 | 8.32E-14 | 0.0279 | 0.37733 | 7.465949821 | 55.74040673 |
| Lysyl oxidase homolog 2 || id:prot-a-1762 | rs4632248 | T | G | 19 | 54324995 | -0.1868 | 4.17E-10 | 0.0299 | 0.21439 | -6.247491639 | 39.03115178 |
| M-phase inducer phosphatase 2 || id:prot-a-472 | rs704 | A | G | 17 | 26694861 | 0.2078 | 1.78E-17 | 0.0244 | 0.46665 | 8.516393443 | 72.52895727 |
| M-phase inducer phosphatase 2 || id:prot-a-472 | rs429358 | C | T | 19 | 45411941 | 0.1974 | 4.57E-09 | 0.0337 | 0.15301 | 5.857566766 | 34.31108841 |
| Macrophage-stimulating protein receptor || id:prot-a-1955 | rs33944729 | T | TA | 1 | 196652124 | 0.1585 | 3.89E-10 | 0.0253 | 0.38701 | 6.264822134 | 39.24799638 |
| Macrophage-stimulating protein receptor || id:prot-a-1955 | rs74480769 | G | A | 5 | 40972211 | -0.4727 | 1.12E-10 | 0.0733 | 0.0317 | -6.448840382 | 41.58754227 |
| Macrophage mannose receptor 1 || id:prot-a-1937 | rs118160793 | G | A | 10 | 17856444 | 0.6537 | 8.91E-96 | 0.0315 | 0.17552 | 20.75238095 | 430.6613152 |
| Macrophage mannose receptor 1 || id:prot-a-1937 | rs72778312 | T | C | 10 | 18257485 | 0.4363 | 3.39E-08 | 0.079 | 0.02517 | 5.52278481 | 30.50115206 |
| Macrophage metalloelastase || id:prot-a-1912 | rs79470110 | C | G | 11 | 103607702 | -0.4532 | 3.47E-08 | 0.0822 | 0.02626 | -5.513381995 | 30.39738102 |
| Macrophage metalloelastase || id:prot-a-1912 | rs28381684 | T | A | 11 | 102737192 | -0.7789 | 5.13E-111 | 0.0348 | 0.12464 | -22.38218391 | 500.9621565 |
| Macrophage migration inhibitory factor || id:prot-a-1900 | rs62143198 | A | G | 19 | 54320939 | 0.4418 | 7.59E-50 | 0.0298 | 0.21453 | 14.82550336 | 219.7955498 |
| Macrophage scavenger receptor types I and II || id:prot-a-1952 | rs4795433 | T | C | 17 | 26716821 | -0.1394 | 1.17E-08 | 0.0244 | 0.50963 | -5.713114754 | 32.63968019 |
| MAGUK p55 subfamily member 6 || id:prot-a-1931 | rs33944729 | T | TA | 1 | 196652124 | 0.1447 | 1.12E-08 | 0.0254 | 0.38701 | 5.696850394 | 32.45410441 |
| MAGUK p55 subfamily member 6 || id:prot-a-1931 | rs704 | A | G | 17 | 26694861 | 0.3127 | 1.51E-38 | 0.0241 | 0.46665 | 12.97510373 | 168.3533169 |
| MAGUK p55 subfamily member 6 || id:prot-a-1931 | rs77258336 | G | C | 17 | 5128858 | -0.4003 | 2.82E-08 | 0.0721 | 0.03196 | -5.552011096 | 30.82482721 |
| MAGUK p55 subfamily member 7 || id:prot-a-1932 | rs6993770 | T | A | 8 | 106581528 | -0.1746 | 1.78E-10 | 0.0274 | 0.28225 | -6.372262774 | 40.60573286 |
| Major prion protein || id:prot-a-2379 | rs71477232 | A | G | 11 | 114030651 | -0.2833 | 4.07E-08 | 0.0516 | 0.06799 | -5.490310078 | 30.14350475 |
| Malate dehydrogenase, cytoplasmic || id:prot-a-1870 | rs62143197 | A | G | 19 | 54320716 | 0.345 | 7.76E-31 | 0.0299 | 0.22311 | 11.53846154 | 133.1360947 |
| Malectin || id:prot-a-1904 | rs55688436 | T | C | 10 | 73643968 | 0.1728 | 1.70E-08 | 0.0306 | 0.20963 | 5.647058824 | 31.88927336 |
| Malectin || id:prot-a-1904 | rs62143197 | A | G | 19 | 54320716 | 0.166 | 4.47E-08 | 0.0303 | 0.22311 | 5.478547855 | 30.0144866 |
| Malignant T-cell-amplified sequence 1 || id:prot-a-1869 | rs10424405 | G | A | 19 | 54321933 | 0.3637 | 1.58E-34 | 0.0297 | 0.2203 | 12.24579125 | 149.9594032 |
| Mammaglobin-B || id:prot-a-2648 | rs6993770 | T | A | 8 | 106581528 | -0.1544 | 1.74E-08 | 0.0274 | 0.28225 | -5.635036496 | 31.75363632 |
| Mammaglobin-B || id:prot-a-2648 | rs10733789 | C | T | 10 | 64948684 | 0.1578 | 6.31E-09 | 0.0272 | 0.31231 | 5.801470588 | 33.65706099 |
| Mannan-binding lectin serine protease 1 || id:prot-a-1855 | rs3032928 | CTTT | C | 17 | 26679563 | -0.1802 | 4.07E-13 | 0.0248 | 0.45639 | -7.266129032 | 52.79663111 |
| Mannose-binding protein C || id:prot-a-1863 | rs545971 | T | C | 9 | 136143372 | 0.2341 | 5.37E-19 | 0.0263 | 0.314 | 8.901140684 | 79.23030548 |
| Mannose-binding protein C || id:prot-a-1863 | rs7899547 | G | T | 10 | 54536839 | 0.9477 | 1.00E-200 | 0.0202 | 0.65277 | 46.91584158 | 2201.096192 |
| Mannose-binding protein C || id:prot-a-1863 | rs7907283 | A | G | 10 | 54669056 | -0.2569 | 5.89E-09 | 0.0441 | 0.08565 | -5.825396825 | 33.93524817 |
| Mannose-binding protein C || id:prot-a-1863 | rs1992528 | G | A | 10 | 54500275 | 0.2337 | 2.04E-09 | 0.039 | 0.88558 | 5.992307692 | 35.90775148 |
| Mannosyl-oligosaccharide 1,2-alpha-mannosidase IB || id:prot-a-1833 | rs7512060 | T | A | 1 | 117961171 | -0.3419 | 4.07E-19 | 0.0383 | 0.1125 | -8.92689295 | 79.68941775 |
| Mannosyl-oligosaccharide 1,2-alpha-mannosidase IB || id:prot-a-1833 | rs651007 | T | C | 9 | 136153875 | 0.2073 | 6.46E-12 | 0.0302 | 0.20669 | 6.864238411 | 47.11776896 |
| Mannosyl-oligosaccharide 1,2-alpha-mannosidase IB || id:prot-a-1833 | rs10418046 | G | T | 19 | 54327869 | -0.1628 | 4.90E-08 | 0.0298 | 0.21727 | -5.463087248 | 29.84532228 |
| MANSC domain-containing protein 1 || id:prot-a-1838 | rs5795487 | A | AT | 11 | 126101305 | 0.1661 | 1.74E-09 | 0.0276 | 0.74667 | 6.018115942 | 36.21771949 |
| MANSC domain-containing protein 1 || id:prot-a-1838 | rs2160588 | A | G | 12 | 12487447 | 0.5024 | 8.71E-42 | 0.0371 | 0.1197 | 13.54177898 | 183.3797778 |
| MANSC domain-containing protein 4 || id:prot-a-1839 | rs36138811 | C | T | 12 | 27927881 | 0.6369 | 7.41E-117 | 0.0277 | 0.23178 | 22.99277978 | 528.6679222 |
| MAP kinase-activated protein kinase 2 || id:prot-a-1851 | rs62143197 | A | G | 19 | 54320716 | 0.3858 | 1.62E-38 | 0.0297 | 0.22311 | 12.98989899 | 168.7374758 |
| MAP kinase-activated protein kinase 3 || id:prot-a-1852 | rs1926446 | A | C | 13 | 46629995 | -0.5757 | 1.48E-114 | 0.0253 | 0.71127 | -22.75494071 | 517.7873268 |
| MAP kinase-activated protein kinase 3 || id:prot-a-1852 | rs62143198 | A | G | 19 | 54320939 | 0.3113 | 6.17E-25 | 0.0302 | 0.21453 | 10.30794702 | 106.2537718 |
| MAP kinase-activated protein kinase 5 || id:prot-a-1854 | rs2048493 | C | G | 3 | 165544302 | -0.168 | 4.17E-11 | 0.0255 | 0.64561 | -6.588235294 | 43.40484429 |
| MAP kinase-activated protein kinase 5 || id:prot-a-1854 | rs704 | A | G | 17 | 26694861 | 0.6329 | 6.17E-181 | 0.0221 | 0.46665 | 28.63800905 | 820.1355623 |
| MAP kinase-activated protein kinase 5 || id:prot-a-1854 | rs41290108 | C | G | 19 | 45377842 | -0.3841 | 3.98E-09 | 0.0653 | 0.03644 | -5.882082695 | 34.59889683 |
| MAP kinase-activated protein kinase 5 || id:prot-a-1854 | rs5167 | G | T | 19 | 45448465 | 0.7552 | 1.00E-200 | 0.0224 | 0.34696 | 33.71428571 | 1136.653061 |
| Maspardin || id:prot-a-2815 | rs9854049 | A | G | 3 | 137243429 | -0.1387 | 3.89E-08 | 0.0252 | 0.6267 | -5.503968254 | 30.29366654 |
| Maspardin || id:prot-a-2815 | rs117824171 | C | T | 10 | 82835722 | -0.5288 | 1.55E-08 | 0.0935 | 0.01815 | -5.655614973 | 31.98598073 |
| Maspardin || id:prot-a-2815 | rs10418046 | G | T | 19 | 54327869 | 0.6116 | 1.55E-105 | 0.028 | 0.21727 | 21.84285714 | 477.1104082 |
| Mast cell-expressed membrane protein 1 || id:prot-a-1865 | rs7539005 | T | A | 1 | 196667252 | -0.1933 | 2.57E-14 | 0.0254 | 0.61028 | -7.61023622 | 57.91569533 |
| Mast cell-expressed membrane protein 1 || id:prot-a-1865 | rs74480769 | G | A | 5 | 40972211 | -0.628 | 7.41E-18 | 0.0729 | 0.0317 | -8.614540466 | 74.21030745 |
| Matrilin-2 || id:prot-a-1857 | rs17831160 | A | G | 8 | 99045866 | 0.4244 | 3.72E-10 | 0.0677 | 0.03438 | 6.268833087 | 39.29826827 |
| Matrilin-3 || id:prot-a-1858 | rs74480769 | G | A | 5 | 40972211 | -0.4487 | 9.55E-10 | 0.0733 | 0.0317 | -6.121418827 | 37.47176845 |
| Matrilin-3 || id:prot-a-1858 | rs143821447 | A | G | 17 | 63620384 | -1.1384 | 1.62E-08 | 0.2016 | 0.00432 | -5.646825397 | 31.88663706 |
| Matrilin-3 || id:prot-a-1858 | rs62143196 | G | A | 19 | 54320636 | -0.1854 | 8.71E-10 | 0.0302 | 0.22404 | -6.139072848 | 37.68821543 |
| Matrilin-4 || id:prot-a-1859 | rs11697677 | G | A | 20 | 43925554 | -0.2298 | 2.45E-16 | 0.028 | 0.25354 | -8.207142857 | 67.35719388 |
| Matrilysin || id:prot-a-1919 | rs139188992 | C | G | 8 | 139894405 | -0.6797 | 3.39E-08 | 0.1231 | 0.01336 | -5.521527214 | 30.48726277 |
| Matrix-remodeling-associated protein 7 || id:prot-a-1974 | rs9900613 | T | C | 17 | 74674857 | -0.229 | 1.91E-20 | 0.0247 | 0.43051 | -9.271255061 | 85.9561704 |
| Matrix-remodeling-associated protein 8 || id:prot-a-1975 | rs6589573 | G | C | 11 | 116685766 | -0.2023 | 3.02E-08 | 0.0365 | 0.87511 | -5.542465753 | 30.71892663 |
| Matrix metalloproteinase-16 || id:prot-a-1915 | rs28761328 | A | T | 18 | 4746271 | 0.2167 | 7.24E-09 | 0.0375 | 0.12814 | 5.778666667 | 33.39298844 |
| Matrix metalloproteinase-16 || id:prot-a-1916 | rs3851539 | A | G | 8 | 89322905 | -0.1467 | 7.76E-09 | 0.0254 | 0.58982 | -5.775590551 | 33.35744621 |
| Matrix metalloproteinase-17 || id:prot-a-1917 | rs74480769 | G | A | 5 | 40972211 | -0.4224 | 8.51E-09 | 0.0734 | 0.0317 | -5.754768392 | 33.11735925 |
| Matrix metalloproteinase-17 || id:prot-a-1917 | rs13209980 | A | T | 6 | 144639010 | -0.397 | 3.24E-08 | 0.0718 | 0.03116 | -5.529247911 | 30.57258246 |
| Matrix metalloproteinase-17 || id:prot-a-1917 | rs62143196 | G | A | 19 | 54320636 | -0.1849 | 9.77E-10 | 0.0302 | 0.22404 | -6.122516556 | 37.48520898 |
| Matrix metalloproteinase-9 || id:prot-a-1921 | rs398076 | A | G | 14 | 88414211 | 0.156 | 1.02E-09 | 0.0255 | 0.41095 | 6.117647059 | 37.42560554 |
| Matrix metalloproteinase-9 || id:prot-a-1921 | rs62143194 | G | C | 19 | 54319624 | 0.1974 | 1.05E-10 | 0.0306 | 0.22364 | 6.450980392 | 41.61514802 |
| Matrix metalloproteinase-9 || id:prot-a-1921 | rs2250889 | C | G | 20 | 44642406 | -0.6221 | 2.09E-26 | 0.0585 | 0.95338 | -10.63418803 | 113.0859551 |
| MAX gene-associated protein || id:prot-a-1891 | rs13402560 | G | C | 2 | 3639915 | -0.1847 | 1.86E-08 | 0.0328 | 0.81367 | -5.631097561 | 31.70925974 |
| MAX gene-associated protein || id:prot-a-1891 | rs704 | A | G | 17 | 26694861 | 0.4008 | 3.24E-64 | 0.0237 | 0.46665 | 16.91139241 | 285.9951931 |
| Mediator of RNA polymerase II transcription subunit 1 || id:prot-a-1874 | rs116255295 | A | G | 6 | 42906241 | 0.1541 | 4.27E-08 | 0.0281 | 0.26129 | 5.483985765 | 30.07409987 |
| Mediator of RNA polymerase II transcription subunit 1 || id:prot-a-1875 | rs6119 | G | A | 14 | 95054012 | 0.6874 | 6.17E-70 | 0.0389 | 0.09924 | 17.67095116 | 312.2625148 |
| Mediator of RNA polymerase II transcription subunit 4 || id:prot-a-1876 | rs5167 | G | T | 19 | 45448465 | 0.4187 | 2.63E-63 | 0.0249 | 0.34696 | 16.81526104 | 282.753004 |
| Mediator of RNA polymerase II transcription subunit 4 || id:prot-a-1876 | rs62143194 | G | C | 19 | 54319624 | 0.1675 | 4.47E-08 | 0.0306 | 0.22364 | 5.473856209 | 29.9631018 |
| Melanocortin-2 receptor accessory protein || id:prot-a-1936 | rs4632248 | T | G | 19 | 54324995 | -0.1963 | 5.13E-11 | 0.0299 | 0.21439 | -6.565217391 | 43.1020794 |
| Melanocyte protein PMEL || id:prot-a-2310 | rs3213122 | A | C | 12 | 56363666 | 0.2643 | 6.61E-09 | 0.0456 | 0.07964 | 5.796052632 | 33.59422611 |
| Melanocyte protein PMEL || id:prot-a-2310 | rs34714188 | A | T | 16 | 89781756 | -0.2849 | 1.38E-11 | 0.0421 | 0.09853 | -6.767220903 | 45.79527874 |
| Melanoma-associated antigen 10 || id:prot-a-1827 | rs33944729 | T | TA | 1 | 196652124 | 0.1483 | 4.79E-09 | 0.0253 | 0.38701 | 5.861660079 | 34.35905888 |
| Melanoma-associated antigen 10 || id:prot-a-1827 | rs704 | A | G | 17 | 26694861 | 0.2591 | 1.38E-26 | 0.0243 | 0.46665 | 10.66255144 | 113.6900032 |
| Melanoma-associated antigen 3 || id:prot-a-1828 | rs13402475 | G | C | 2 | 3639909 | -0.2748 | 1.32E-17 | 0.0322 | 0.80838 | -8.534161491 | 72.83191235 |
| Melanoma-associated antigen 3 || id:prot-a-1828 | rs5167 | G | T | 19 | 45448465 | 0.277 | 2.04E-27 | 0.0255 | 0.34696 | 10.8627451 | 117.9992311 |
| Melanoma-derived growth regulatory protein || id:prot-a-1896 | rs2604877 | C | T | 19 | 41275048 | 1.4417 | 1.00E-200 | 0.0424 | 0.06803 | 34.00235849 | 1156.160383 |
| Melanoma-derived growth regulatory protein || id:prot-a-1896 | rs201285225 | CTTACTCTCTTGA | C | 19 | 41399598 | -1.0682 | 1.26E-31 | 0.0913 | 0.98172 | -11.69989047 | 136.887437 |
| Membrane-associated progesterone receptor component 1 || id:prot-a-2259 | rs9814412 | T | C | 3 | 186383180 | 0.1586 | 2.29E-08 | 0.0284 | 0.24288 | 5.584507042 | 31.1867189 |
| Membrane-associated progesterone receptor component 2 || id:prot-a-2260 | rs34127959 | T | C | 12 | 95285575 | 0.3747 | 7.94E-09 | 0.0649 | 0.03882 | 5.773497689 | 33.33327556 |
| Membrane-associated progesterone receptor component 2 || id:prot-a-2261 | rs1061170 | T | C | 1 | 196659237 | 0.3764 | 1.66E-52 | 0.0247 | 0.61265 | 15.2388664 | 232.2230491 |
| Membrane-associated progesterone receptor component 2 || id:prot-a-2262 | rs74480769 | G | A | 5 | 40972211 | -0.4043 | 3.63E-08 | 0.0734 | 0.0317 | -5.508174387 | 30.33998508 |
| Membrane protein FAM174A || id:prot-a-1034 | rs6586145 | A | T | 10 | 90433345 | -0.8776 | 1.23E-107 | 0.0398 | 0.0893 | -22.05025126 | 486.2135805 |
| Mesothelin || id:prot-a-1947 | rs704 | A | G | 17 | 26694861 | 0.1975 | 6.31E-16 | 0.0244 | 0.46665 | 8.094262295 | 65.5170821 |
| Metalloproteinase inhibitor 4 || id:prot-a-2980 | rs4135273 | A | G | 3 | 12439329 | 0.3727 | 1.00E-08 | 0.065 | 0.04068 | 5.733846154 | 32.87699172 |
| Metalloproteinase inhibitor 4 || id:prot-a-2980 | rs454615 | C | T | 3 | 12077010 | 0.4903 | 1.66E-53 | 0.0318 | 0.83083 | 15.41823899 | 237.7220937 |
| Metallothionein-1F || id:prot-a-1958 | rs9881048 | C | A | 3 | 165506252 | -0.1802 | 1.78E-08 | 0.032 | 0.8162 | -5.63125 | 31.71097656 |
| Metallothionein-1F || id:prot-a-1958 | rs704 | A | G | 17 | 26694861 | 0.3996 | 8.71E-64 | 0.0237 | 0.46665 | 16.86075949 | 284.2852107 |
| Methyl-CpG-binding domain protein 4 || id:prot-a-1862 | rs76102861 | G | A | 4 | 19983426 | 0.3388 | 4.47E-08 | 0.0619 | 0.04572 | 5.473344103 | 29.95749567 |
| MHC class I polypeptide-related sequence B || id:prot-a-1898 | rs9378193 | C | T | 6 | 31293942 | -0.5902 | 3.47E-52 | 0.0388 | 0.14196 | -15.21134021 | 231.3848709 |
| MHC class I polypeptide-related sequence B || id:prot-a-1898 | rs1611236 | A | G | 6 | 29748690 | -0.1603 | 1.58E-09 | 0.0266 | 0.31728 | -6.026315789 | 36.31648199 |
| MHC class I polypeptide-related sequence B || id:prot-a-1898 | rs1131202 | T | A | 6 | 31324539 | 0.8756 | 1.05E-40 | 0.0655 | 0.04349 | 13.36793893 | 178.7017913 |
| MHC class I polypeptide-related sequence B || id:prot-a-1898 | rs142730696 | T | TTTTTC | 6 | 26360443 | -0.3171 | 8.51E-19 | 0.0358 | 0.13836 | -8.857541899 | 78.4560485 |
| MHC class I polypeptide-related sequence B || id:prot-a-1898 | rs3134900 | G | C | 6 | 31473957 | -0.7115 | 2.45E-101 | 0.0333 | 0.13955 | -21.36636637 | 456.5216117 |
| MICAL-like protein 2 || id:prot-a-1897 | rs4795433 | T | C | 17 | 26716821 | 0.3457 | 1.00E-47 | 0.0238 | 0.50963 | 14.52521008 | 210.981728 |
| MICOS complex subunit MIC10 || id:prot-a-1902 | rs117032462 | G | A | 17 | 26833853 | -0.4317 | 3.31E-10 | 0.0687 | 0.03301 | -6.283842795 | 39.48668027 |
| MICOS complex subunit MIC10 || id:prot-a-1902 | rs704 | A | G | 17 | 26694861 | 1.1589 | 1.00E-200 | 0.0142 | 0.46665 | 81.61267606 | 6660.628893 |
| MICOS complex subunit MIC10 || id:prot-a-1902 | rs8103996 | C | A | 19 | 57609860 | 0.1361 | 4.57E-08 | 0.0249 | 0.52231 | 5.465863454 | 29.8756633 |
| MICOS complex subunit MIC26 || id:prot-a-138 | rs34436714 | A | C | 19 | 54327313 | -0.1716 | 1.12E-08 | 0.03 | 0.21294 | -5.72 | 32.7184 |
| Microfibril-associated glycoprotein 4 || id:prot-a-1886 | rs139356332 | C | G | 17 | 19289286 | -0.4654 | 4.57E-10 | 0.0747 | 0.03335 | -6.230254351 | 38.81606927 |
| Microfibrillar-associated protein 1 || id:prot-a-1884 | rs78803644 | A | T | 5 | 72860314 | 0.3643 | 3.47E-08 | 0.066 | 0.03682 | 5.51969697 | 30.46705464 |
| Microfibrillar-associated protein 2 || id:prot-a-1885 | rs4920605 | A | G | 1 | 17315425 | 0.2073 | 1.32E-17 | 0.0243 | 0.55354 | 8.530864198 | 72.77564396 |
| Microfibrillar-associated protein 2 || id:prot-a-1885 | rs1863622 | C | T | 3 | 186396616 | 0.1896 | 4.68E-10 | 0.0304 | 0.21611 | 6.236842105 | 38.89819945 |
| Microfibrillar-associated protein 2 || id:prot-a-1885 | rs73047825 | G | A | 12 | 9023610 | -0.3007 | 4.90E-11 | 0.0457 | 0.08104 | -6.579868709 | 43.29467223 |
| Mineralocorticoid receptor || id:prot-a-2089 | rs62143206 | T | G | 19 | 54326212 | 0.3683 | 4.68E-36 | 0.0294 | 0.2131 | 12.52721088 | 156.9310125 |
| Mitochondrial import inner membrane translocase subunit TIM14 || id:prot-a-847 | rs62054235 | T | C | 16 | 80977137 | 0.3962 | 2.88E-08 | 0.0714 | 0.03357 | 5.549019608 | 30.79161861 |
| Mitochondrial import inner membrane translocase subunit TIM14 || id:prot-a-847 | rs4632248 | T | G | 19 | 54324995 | -0.2085 | 2.88E-12 | 0.0299 | 0.21439 | -6.973244147 | 48.62613394 |
| Mitochondrial sodium/hydrogen exchanger 9B2 || id:prot-a-2764 | rs7144298 | A | G | 14 | 106248399 | -0.2577 | 1.17E-20 | 0.0276 | 0.63198 | -9.336956522 | 87.17875709 |
| Mitochondrial ubiquitin ligase activator of NFKB 1 || id:prot-a-1969 | rs11906233 | A | G | 20 | 50397185 | -0.2163 | 4.47E-08 | 0.0395 | 0.11563 | -5.475949367 | 29.98602147 |
| Mitochondrial ubiquitin ligase activator of NFKB 1 || id:prot-a-1970 | rs11086556 | G | A | 20 | 36969972 | -0.4757 | 3.47E-26 | 0.0449 | 0.08272 | -10.59465479 | 112.2467101 |
| Mitogen-activated protein kinase 1 || id:prot-a-1845 | rs62143198 | A | G | 19 | 54320939 | 0.5009 | 9.12E-65 | 0.0295 | 0.21453 | 16.97966102 | 288.3088883 |
| Mitogen-activated protein kinase 13 || id:prot-a-1846 | rs12210904 | A | C | 6 | 36098191 | 0.2538 | 1.48E-21 | 0.0266 | 0.28948 | 9.541353383 | 91.03742439 |
| Mitogen-activated protein kinase 13 || id:prot-a-1846 | rs62143206 | T | G | 19 | 54326212 | 0.6531 | 1.05E-121 | 0.0278 | 0.2131 | 23.49280576 | 551.9119223 |
| Mitogen-activated protein kinase 14 || id:prot-a-1847 | rs62143198 | A | G | 19 | 54320939 | 0.5128 | 4.57E-68 | 0.0294 | 0.21453 | 17.44217687 | 304.229534 |
| Mitogen-activated protein kinase 3 || id:prot-a-1848 | rs7201780 | T | C | 16 | 30147265 | 0.1469 | 3.24E-09 | 0.0248 | 0.44698 | 5.923387097 | 35.0865147 |
| Mitogen-activated protein kinase 3 || id:prot-a-1848 | rs62143198 | A | G | 19 | 54320939 | 0.626 | 3.72E-105 | 0.0287 | 0.21453 | 21.81184669 | 475.756656 |
| Mitogen-activated protein kinase 8 || id:prot-a-1849 | rs62143198 | A | G | 19 | 54320939 | 0.6033 | 8.13E-97 | 0.0289 | 0.21453 | 20.87543253 | 435.7836831 |
| Mitogen-activated protein kinase kinase kinase 3 || id:prot-a-1843 | rs62143206 | T | G | 19 | 54326212 | 0.6266 | 9.33E-111 | 0.028 | 0.2131 | 22.37857143 | 500.8004592 |
| Mitotic-spindle organizing protein 1 || id:prot-a-1988 | rs147137561 | A | G | 3 | 114416592 | -0.795 | 4.07E-08 | 0.1448 | 0.00782 | -5.490331492 | 30.14373989 |
| Mitotic checkpoint serine/threonine-protein kinase BUB1 || id:prot-a-279 | rs10418046 | G | T | 19 | 54327869 | 0.4622 | 1.23E-57 | 0.0289 | 0.21727 | 15.99307958 | 255.7785946 |
| Mitotic spindle assembly checkpoint protein MAD1 || id:prot-a-1825 | rs4795433 | T | C | 17 | 26716821 | 0.1783 | 2.51E-13 | 0.0244 | 0.50963 | 7.307377049 | 53.39775934 |
| MOB kinase activator 1A || id:prot-a-1925 | rs10424405 | G | A | 19 | 54321933 | 0.17 | 1.82E-08 | 0.0302 | 0.2203 | 5.629139073 | 31.6872067 |
| Mono [ADP-ribose] polymerase PARP16 || id:prot-a-2184 | rs2304456 | G | T | 3 | 186445052 | 1.0054 | 2.88E-199 | 0.0334 | 0.12046 | 30.10179641 | 906.1181469 |
| Mothers against decapentaplegic homolog 2 || id:prot-a-2771 | rs62143197 | A | G | 19 | 54320716 | 0.4113 | 7.94E-44 | 0.0296 | 0.22311 | 13.89527027 | 193.0785359 |
| Mothers against decapentaplegic homolog 4 || id:prot-a-2772 | rs10418046 | G | T | 19 | 54327869 | 0.4275 | 5.01E-49 | 0.029 | 0.21727 | 14.74137931 | 217.308264 |
| Mucin-1 || id:prot-a-1967 | rs704 | A | G | 17 | 26694861 | 0.4443 | 4.79E-80 | 0.0235 | 0.46665 | 18.90638298 | 357.4513173 |
| Mucin-4 || id:prot-a-1968 | rs175902 | C | T | 14 | 76314533 | 0.2381 | 4.90E-08 | 0.0436 | 0.09099 | 5.461009174 | 29.8226212 |
| Mucosal addressin cell adhesion molecule 1 || id:prot-a-1826 | rs117991338 | C | G | 17 | 70760246 | -0.6264 | 3.39E-08 | 0.1135 | 0.01192 | -5.518942731 | 30.45872887 |
| Muellerian-inhibiting factor || id:prot-a-84 | rs34038940 | A | T | 11 | 6443672 | 0.2526 | 2.63E-18 | 0.0289 | 0.2467 | 8.740484429 | 76.39606805 |
| Multifunctional protein ADE2 || id:prot-a-2167 | rs10903330 | G | A | 8 | 10673104 | -0.1401 | 3.98E-08 | 0.0255 | 0.63034 | -5.494117647 | 30.18532872 |
| Multimerin-2 || id:prot-a-1922 | rs61802332 | T | C | 1 | 161589756 | -0.1504 | 8.91E-09 | 0.0262 | 0.48882 | -5.740458015 | 32.95285823 |
| Multimerin-2 || id:prot-a-1922 | rs72798113 | C | T | 10 | 20636925 | 0.3939 | 1.51E-08 | 0.0696 | 0.03743 | 5.659482759 | 32.0297451 |
| Multimerin-2 || id:prot-a-1922 | rs58452280 | C | A | 14 | 106252435 | 1.1833 | 1.15E-199 | 0.0392 | 0.1235 | 30.18622449 | 911.2081489 |
| Multimerin-2 || id:prot-a-1922 | rs112702477 | G | T | 14 | 106029909 | 1.0054 | 7.41E-17 | 0.1206 | 0.0133 | 8.336650083 | 69.49973461 |
| Multimerin-2 || id:prot-a-1923 | rs4820092 | T | C | 22 | 33160204 | -0.191 | 3.89E-12 | 0.0275 | 0.72825 | -6.945454545 | 48.23933884 |
| Multiple coagulation factor deficiency protein 2 || id:prot-a-1867 | rs112124468 | G | C | 14 | 105921253 | 0.7539 | 4.37E-12 | 0.1089 | 0.01793 | 6.922865014 | 47.92606 |
| Multiple coagulation factor deficiency protein 2 || id:prot-a-1867 | rs12147642 | T | G | 14 | 106205022 | -1.1603 | 1.00E-200 | 0.018 | 0.6618 | -64.46111111 | 4155.234846 |
| Multiple coagulation factor deficiency protein 2 || id:prot-a-1867 | rs189483948 | C | T | 14 | 106742304 | 0.6316 | 1.20E-11 | 0.0932 | 0.02426 | 6.776824034 | 45.92534399 |
| Multiple inositol polyphosphate phosphatase 1 || id:prot-a-1903 | rs3864106 | C | G | 3 | 186656602 | -0.3114 | 6.76E-37 | 0.0245 | 0.5906 | -12.71020408 | 161.5492878 |
| Multiple inositol polyphosphate phosphatase 1 || id:prot-a-1903 | rs187099314 | T | C | 9 | 136130741 | 0.3164 | 1.58E-10 | 0.0495 | 0.06733 | 6.391919192 | 40.85663096 |
| Multiple inositol polyphosphate phosphatase 1 || id:prot-a-1903 | rs3120102 | C | T | 10 | 89181557 | -0.1478 | 4.47E-09 | 0.0252 | 0.4204 | -5.865079365 | 34.39915596 |
| Muscle, skeletal receptor tyrosine-protein kinase || id:prot-a-1971 | rs76442786 | A | G | 3 | 194107287 | -0.3037 | 2.95E-08 | 0.0548 | 0.05635 | -5.541970803 | 30.71344038 |
| Myc box-dependent-interacting protein 1 || id:prot-a-250 | rs35103166 | C | T | 2 | 127882182 | 0.1569 | 1.12E-09 | 0.0258 | 0.36524 | 6.081395349 | 36.98336939 |
| Myc target protein 1 || id:prot-a-1979 | rs1126477 | T | C | 3 | 46501268 | 0.4647 | 1.07E-61 | 0.028 | 0.23937 | 16.59642857 | 275.4414413 |
| Myelin protein P0 || id:prot-a-1934 | rs7022 | G | C | 1 | 230417664 | 0.2019 | 3.31E-14 | 0.0266 | 0.31068 | 7.590225564 | 57.61152411 |
| Myelin protein P0 || id:prot-a-1934 | rs7527527 | C | G | 1 | 230241154 | 0.1758 | 1.15E-11 | 0.0259 | 0.43776 | 6.787644788 | 46.07212176 |
| Myelin regulatory factor || id:prot-a-1982 | rs33944729 | T | TA | 1 | 196652124 | 0.1468 | 6.92E-09 | 0.0253 | 0.38701 | 5.802371542 | 33.66751551 |
| Myelin regulatory factor || id:prot-a-1982 | rs704 | A | G | 17 | 26694861 | 0.1568 | 1.70E-10 | 0.0245 | 0.46665 | 6.4 | 40.96 |
| Myeloblastin || id:prot-a-2395 | rs1639114 | A | C | 6 | 31219224 | 0.5987 | 2.00E-09 | 0.0998 | 0.01469 | 5.998997996 | 35.98797696 |
| Myeloblastin || id:prot-a-2395 | rs10425544 | C | T | 19 | 836043 | 0.5656 | 1.41E-96 | 0.0271 | 0.71281 | 20.87084871 | 435.5923258 |
| Myeloblastin || id:prot-a-2395 | rs113254919 | A | G | 19 | 866597 | -0.1804 | 1.91E-11 | 0.0269 | 0.66003 | -6.706319703 | 44.97472395 |
| Myeloblastin || id:prot-a-2396 | rs28929474 | T | C | 14 | 94844947 | -0.4859 | 6.17E-09 | 0.0836 | 0.02269 | -5.812200957 | 33.78167996 |
| Myeloblastin || id:prot-a-2396 | rs376558419 | C | G | 19 | 879821 | -0.1825 | 9.55E-10 | 0.0298 | 0.25251 | -6.124161074 | 37.50534886 |
| Myeloblastin || id:prot-a-2396 | rs10425544 | C | T | 19 | 836043 | 0.5991 | 8.32E-110 | 0.0269 | 0.71281 | 22.27137546 | 496.0141651 |
| Myeloid cell surface antigen CD33 || id:prot-a-439 | rs62114140 | T | C | 19 | 51575009 | -0.2485 | 4.68E-09 | 0.0424 | 0.10014 | -5.860849057 | 34.34955166 |
| Myeloid cell surface antigen CD33 || id:prot-a-439 | rs12459419 | T | C | 19 | 51728477 | -0.9436 | 1.00E-200 | 0.0209 | 0.32917 | -45.14832536 | 2038.371283 |
| Myeloid zinc finger 1 || id:prot-a-1987 | rs12790672 | T | C | 11 | 6447423 | 0.1733 | 3.09E-09 | 0.0293 | 0.24342 | 5.914675768 | 34.98338944 |
| Myeloperoxidase || id:prot-a-1930 | rs4925496 | G | A | 1 | 229633371 | 0.1882 | 1.70E-11 | 0.028 | 0.2642 | 6.721428571 | 45.17760204 |
| Myeloperoxidase || id:prot-a-1930 | rs56213534 | G | A | 11 | 17324948 | 0.1537 | 1.26E-08 | 0.027 | 0.30462 | 5.692592593 | 32.40561043 |
| Myeloperoxidase || id:prot-a-1930 | rs34097845 | T | C | 17 | 56358429 | -0.5736 | 8.51E-31 | 0.0497 | 0.0673 | -11.54124748 | 133.2003935 |
| Myeloperoxidase || id:prot-a-1930 | rs13036464 | C | G | 20 | 17665867 | 0.1402 | 2.69E-08 | 0.0252 | 0.39951 | 5.563492063 | 30.95244394 |
| Myocardial zonula adherens protein || id:prot-a-1985 | rs12445395 | T | A | 16 | 81844255 | 0.1768 | 3.89E-08 | 0.0322 | 0.80794 | 5.49068323 | 30.14760233 |
| Myosin-binding protein C, slow-type || id:prot-a-1977 | rs59931471 | C | T | 12 | 102045639 | -0.1973 | 5.62E-09 | 0.0339 | 0.15552 | -5.820058997 | 33.87308673 |
| Myotonin-protein kinase || id:prot-a-834 | rs10418046 | G | T | 19 | 54327869 | -0.2038 | 7.76E-12 | 0.0298 | 0.21727 | -6.838926174 | 46.77091122 |
| Myotubularin-related protein 1 || id:prot-a-1963 | rs74480769 | G | A | 5 | 40972211 | -0.4717 | 1.23E-10 | 0.0733 | 0.0317 | -6.435197817 | 41.41177095 |
| N-acetyl-D-glucosamine kinase || id:prot-a-1995 | rs56693109 | T | G | 2 | 71284183 | -0.2248 | 1.70E-08 | 0.0398 | 0.13869 | -5.648241206 | 31.90262872 |
| N-acetyl-D-glucosamine kinase || id:prot-a-1995 | rs7606102 | G | A | 2 | 71276399 | -0.432 | 6.03E-39 | 0.0331 | 0.17031 | -13.05135952 | 170.3379852 |
| N-acetyl-D-glucosamine kinase || id:prot-a-1995 | rs4632248 | T | G | 19 | 54324995 | 0.5042 | 8.51E-69 | 0.0288 | 0.21439 | 17.50694444 | 306.4931038 |
| N-acetylated-alpha-linked acidic dipeptidase 2 || id:prot-a-1991 | rs1534166 | G | A | 3 | 133537067 | -0.2827 | 2.14E-24 | 0.0277 | 0.27555 | -10.20577617 | 104.1578673 |
| N-acetylated-alpha-linked acidic dipeptidase 2 || id:prot-a-1991 | rs79920061 | A | G | 6 | 25857920 | -0.4754 | 6.03E-27 | 0.0442 | 0.08065 | -10.75565611 | 115.6841383 |
| N-acetylated-alpha-linked acidic dipeptidase 2 || id:prot-a-1991 | rs74736153 | C | G | 8 | 37526464 | 0.4986 | 5.89E-09 | 0.0857 | 0.02892 | 5.817969662 | 33.84877098 |
| N-acetylglucosamine-1-phosphodiester alpha-N-acetylglucosaminidase || id:prot-a-1996 | rs12599777 | G | A | 16 | 5079466 | -0.4178 | 1.41E-39 | 0.0317 | 0.2042 | -13.17981073 | 173.7074108 |
| N-acetylglucosamine-1-phosphotransferase subunit gamma || id:prot-a-1232 | rs11248874 | G | A | 16 | 1419947 | -0.2953 | 3.16E-11 | 0.0445 | 0.91522 | -6.635955056 | 44.03589951 |
| N-acetylglucosamine-6-sulfatase || id:prot-a-1235 | rs13292932 | A | C | 9 | 136145419 | -0.1671 | 7.24E-10 | 0.0271 | 0.30497 | -6.166051661 | 38.02019308 |
| N-acetylglucosamine-6-sulfatase || id:prot-a-1236 | rs1293303 | C | G | 8 | 11720227 | 0.17 | 7.08E-12 | 0.0248 | 0.4485 | 6.85483871 | 46.98881374 |
| N-acetyllactosaminide beta-1,3-N-acetylglucosaminyltransferase 2 || id:prot-a-213 | rs4073090 | G | A | 2 | 62563774 | 0.1677 | 1.82E-11 | 0.0249 | 0.42992 | 6.734939759 | 45.35941356 |
| N-acetyllactosaminide beta-1,3-N-acetylglucosaminyltransferase 2 || id:prot-a-213 | rs2519093 | T | C | 9 | 136141870 | 0.3146 | 4.17E-23 | 0.0318 | 0.17823 | 9.893081761 | 97.87306673 |
| N-acetyllactosaminide beta-1,3-N-acetylglucosaminyltransferase 2 || id:prot-a-213 | rs67047091 | T | C | 19 | 41938684 | 0.4512 | 1.58E-78 | 0.024 | 0.38255 | 18.8 | 353.44 |
| N-acetyllactosaminide beta-1,3-N-acetylglucosaminyltransferase 2 || id:prot-a-213 | rs679574 | G | C | 19 | 49206108 | -0.1418 | 6.17E-09 | 0.0244 | 0.50693 | -5.81147541 | 33.77324644 |
| N-acetyllactosaminide beta-1,6-N-acetylglucosaminyl-transferase, isoform C || id:prot-a-1186 | rs3104408 | A | G | 6 | 32683112 | -0.1498 | 4.47E-08 | 0.0274 | 0.55752 | -5.467153285 | 29.88976504 |
| N-acetylserotonin O-methyltransferase-like protein || id:prot-a-189 | rs10461878 | C | T | 5 | 29907434 | 0.1815 | 3.09E-08 | 0.0328 | 0.17707 | 5.533536585 | 30.62002714 |
| N-acetylserotonin O-methyltransferase-like protein || id:prot-a-189 | rs12972849 | A | G | 19 | 54338067 | -0.2144 | 9.77E-13 | 0.0301 | 0.63243 | -7.122923588 | 50.73604044 |
| N-acetyltransferase 14 || id:prot-a-2002 | rs10418046 | G | T | 19 | 54327869 | 0.4457 | 1.95E-53 | 0.029 | 0.21727 | 15.36896552 | 236.2051011 |
| N-acylethanolamine-hydrolyzing acid amidase || id:prot-a-1990 | rs9996608 | T | C | 4 | 76848231 | -0.5485 | 3.89E-101 | 0.0257 | 0.30045 | -21.34241245 | 455.4985692 |
| N-acylethanolamine-hydrolyzing acid amidase || id:prot-a-1990 | rs10745925 | C | T | 12 | 102218899 | -0.3098 | 5.50E-31 | 0.0268 | 0.29402 | -11.55970149 | 133.6266986 |
| N-lysine methyltransferase SMYD2 || id:prot-a-2784 | rs5758009 | C | T | 22 | 22718635 | 0.2627 | 6.61E-11 | 0.0402 | 0.89356 | 6.534825871 | 42.70394916 |
| N-terminal pro-BNP || id:prot-a-2078 | rs198389 | G | A | 1 | 11919271 | 0.2934 | 3.55E-31 | 0.0253 | 0.41259 | 11.59683794 | 134.4866503 |
| N-terminal pro-BNP || id:prot-a-2078 | rs2175388 | T | C | 1 | 155596497 | 0.183 | 2.82E-08 | 0.033 | 0.20246 | 5.545454545 | 30.75206612 |
| N-terminal Xaa-Pro-Lys N-methyltransferase 1 || id:prot-a-2116 | rs4632248 | T | G | 19 | 54324995 | 0.5681 | 4.27E-89 | 0.0284 | 0.21439 | 20.00352113 | 400.1408575 |
| NAD-dependent protein deacetylase sirtuin-2 || id:prot-a-2736 | rs1552641 | A | G | 3 | 126264060 | 0.2606 | 9.12E-20 | 0.0286 | 0.24933 | 9.111888112 | 83.02650496 |
| NAD-dependent protein deacetylase sirtuin-2 || id:prot-a-2736 | rs35524242 | G | A | 10 | 7772035 | -0.7953 | 6.76E-52 | 0.0525 | 0.05595 | -15.14857143 | 229.4792163 |
| NAD-dependent protein deacetylase sirtuin-2 || id:prot-a-2736 | rs9891968 | A | G | 17 | 64132846 | 0.4341 | 5.37E-15 | 0.0555 | 0.04964 | 7.821621622 | 61.17776479 |
| NAD-dependent protein deacylase sirtuin-5, mitochondrial || id:prot-a-2737 | rs6993770 | T | A | 8 | 106581528 | -0.2125 | 6.76E-15 | 0.0273 | 0.28225 | -7.783882784 | 60.58883119 |
| NAD-dependent protein deacylase sirtuin-5, mitochondrial || id:prot-a-2737 | rs10733789 | C | T | 10 | 64948684 | 0.1647 | 1.29E-09 | 0.0271 | 0.31231 | 6.077490775 | 36.93589412 |
| NAD kinase || id:prot-a-1994 | rs62143206 | T | G | 19 | 54326212 | 0.3447 | 1.32E-31 | 0.0295 | 0.2131 | 11.68474576 | 136.5332835 |
| NAD(P)H dehydrogenase [quinone] 1 || id:prot-a-2083 | rs77944668 | A | G | 16 | 69718112 | -0.7498 | 1.62E-154 | 0.0283 | 0.19326 | -26.49469965 | 701.9691094 |
| NADH dehydrogenase [ubiquinone] 1 beta subcomplex subunit 11, mitochondrial || id:prot-a-2022 | rs7212510 | A | T | 17 | 26703682 | 0.1512 | 5.50E-10 | 0.0244 | 0.50911 | 6.196721311 | 38.39935501 |
| NADH dehydrogenase [ubiquinone] 1 beta subcomplex subunit 4 || id:prot-a-2023 | rs570618 | G | T | 1 | 196657064 | -0.1618 | 1.66E-10 | 0.0253 | 0.61149 | -6.395256917 | 40.89931103 |
| NADH dehydrogenase [ubiquinone] 1 beta subcomplex subunit 4 || id:prot-a-2023 | rs704 | A | G | 17 | 26694861 | 0.6326 | 3.63E-180 | 0.0221 | 0.46665 | 28.62443439 | 819.3582441 |
| NADH dehydrogenase [ubiquinone] iron-sulfur protein 4, mitochondrial || id:prot-a-2025 | rs1061170 | T | C | 1 | 196659237 | 0.8715 | 1.00E-200 | 0.0205 | 0.61265 | 42.51219512 | 1807.286734 |
| NADH dehydrogenase [ubiquinone] iron-sulfur protein 4, mitochondrial || id:prot-a-2025 | rs138985516 | T | TC | 20 | 56950177 | 0.2818 | 2.75E-08 | 0.0507 | 0.91399 | 5.558185404 | 30.89342499 |
| Natural cytotoxicity triggering receptor 1 || id:prot-a-2013 | rs2278428 | C | A | 19 | 55418054 | -0.2459 | 3.55E-08 | 0.0446 | 0.08091 | -5.513452915 | 30.39816304 |
| Natural cytotoxicity triggering receptor 1 || id:prot-a-2014 | rs2278428 | C | A | 19 | 55418054 | -0.3557 | 1.15E-15 | 0.0444 | 0.08091 | -8.011261261 | 64.180307 |
| Natural cytotoxicity triggering receptor 3 || id:prot-a-2016 | rs986475 | G | A | 6 | 31556709 | -0.5526 | 1.12E-31 | 0.0472 | 0.07066 | -11.70762712 | 137.0685327 |
| Natural cytotoxicity triggering receptor 3 || id:prot-a-2016 | rs2734331 | G | A | 6 | 31930351 | -0.4493 | 1.51E-11 | 0.0666 | 0.0355 | -6.746246246 | 45.51183841 |
| Natural cytotoxicity triggering receptor 3 ligand 1 || id:prot-a-2017 | rs76711159 | A | G | 3 | 72188039 | -0.2236 | 3.02E-08 | 0.0404 | 0.11515 | -5.534653465 | 30.63238898 |
| Nectin-3 || id:prot-a-2458 | rs7539005 | T | A | 1 | 196667252 | -0.1485 | 5.50E-09 | 0.0255 | 0.61028 | -5.823529412 | 33.91349481 |
| Neogenin || id:prot-a-2033 | rs629301 | T | G | 1 | 109818306 | -0.2333 | 7.08E-15 | 0.03 | 0.77642 | -7.776666667 | 60.47654444 |
| Neogenin || id:prot-a-2033 | rs12903656 | C | G | 15 | 73326961 | 0.2861 | 5.50E-12 | 0.0415 | 0.10075 | 6.893975904 | 47.52690376 |
| Nesprin-2 || id:prot-a-2910 | rs10418046 | G | T | 19 | 54327869 | 0.3017 | 1.58E-24 | 0.0295 | 0.21727 | 10.22711864 | 104.5939558 |
| Netrin-1 || id:prot-a-2118 | rs72809988 | A | G | 17 | 8986397 | -0.5788 | 1.58E-52 | 0.038 | 0.11047 | -15.23157895 | 232.0009972 |
| Netrin-1 || id:prot-a-2118 | rs8071180 | A | G | 17 | 9096958 | 0.3225 | 1.55E-37 | 0.0252 | 0.40276 | 12.79761905 | 163.7790533 |
| Netrin-4 || id:prot-a-2119 | rs17288108 | G | A | 12 | 96131895 | -0.2779 | 7.24E-18 | 0.0323 | 0.18191 | -8.60371517 | 74.02391473 |
| Netrin-G1 || id:prot-a-2120 | rs115668827 | C | G | 1 | 107678268 | 1.0036 | 1.15E-70 | 0.0565 | 0.04877 | 17.76283186 | 315.5181956 |
| Netrin-G1 || id:prot-a-2120 | rs4942475 | C | T | 13 | 46651521 | 0.1743 | 2.51E-11 | 0.0261 | 0.32468 | 6.67816092 | 44.59783327 |
| Netrin receptor UNC5C || id:prot-a-3168 | rs57091121 | T | A | 4 | 96444053 | 0.2694 | 1.95E-25 | 0.0259 | 0.33692 | 10.4015444 | 108.1921259 |
| Netrin receptor UNC5C || id:prot-a-3168 | rs987383 | T | A | 4 | 96547352 | 0.2115 | 1.41E-10 | 0.033 | 0.82957 | 6.409090909 | 41.07644628 |
| Neural cell adhesion molecule 1 || id:prot-a-2007 | rs62302077 | T | C | 4 | 40034518 | -0.1429 | 1.12E-08 | 0.025 | 0.51725 | -5.716 | 32.672656 |
| Neural cell adhesion molecule 1 || id:prot-a-2007 | rs2012888 | A | G | 14 | 106211784 | -0.1953 | 2.95E-12 | 0.028 | 0.31578 | -6.975 | 48.650625 |
| Neural cell adhesion molecule 1 || id:prot-a-2007 | rs10418046 | G | T | 19 | 54327869 | -0.1805 | 1.41E-09 | 0.0298 | 0.21727 | -6.05704698 | 36.68781812 |
| Neural cell adhesion molecule 1, 120 kDa isoform || id:prot-a-2006 | rs7113211 | A | G | 11 | 112642632 | 0.2063 | 1.10E-16 | 0.0249 | 0.48904 | 8.285140562 | 68.64355414 |
| Neural cell adhesion molecule 1, 120 kDa isoform || id:prot-a-2006 | rs1273044 | C | T | 11 | 112993848 | 0.233 | 2.75E-14 | 0.0306 | 0.79818 | 7.614379085 | 57.97876885 |
| Neural cell adhesion molecule 1, 120 kDa isoform || id:prot-a-2006 | rs2288158 | G | T | 11 | 113133676 | 0.2823 | 1.66E-14 | 0.0368 | 0.13347 | 7.671195652 | 58.84724273 |
| Neural cell adhesion molecule 2 || id:prot-a-2008 | rs3830160 | G | A | 16 | 419092 | -0.1958 | 3.63E-15 | 0.0249 | 0.5962 | -7.863453815 | 61.8339059 |
| Neural cell adhesion molecule 2 || id:prot-a-2008 | rs11911765 | C | T | 21 | 22380044 | 0.1787 | 1.32E-13 | 0.0241 | 0.4947 | 7.414937759 | 54.98130197 |
| Neural cell adhesion molecule 2 || id:prot-a-2008 | rs34399466 | T | A | 21 | 22825412 | 0.3384 | 5.50E-31 | 0.0292 | 0.27373 | 11.5890411 | 134.3058735 |
| Neural cell adhesion molecule L1-like protein || id:prot-a-545 | rs1015456 | C | T | 3 | 107776 | -0.2082 | 3.24E-16 | 0.0255 | 0.39178 | -8.164705882 | 66.66242215 |
| Neural cell adhesion molecule L1-like protein || id:prot-a-545 | rs1865166 | G | T | 3 | 437044 | 0.5301 | 1.07E-08 | 0.0927 | 0.98123 | 5.718446602 | 32.70063154 |
| Neural cell adhesion molecule L1-like protein || id:prot-a-545 | rs60843925 | C | T | 11 | 126238832 | -0.2118 | 3.09E-09 | 0.0357 | 0.14078 | -5.932773109 | 35.19779677 |
| Neural cell adhesion molecule L1 || id:prot-a-1690 | rs635634 | T | C | 9 | 136155000 | -0.2121 | 3.98E-11 | 0.0321 | 0.17981 | -6.607476636 | 43.65874749 |
| Neural cell adhesion molecule L1 || id:prot-a-1690 | rs142483848 | T | C | 17 | 58720255 | 0.3565 | 3.24E-08 | 0.0645 | 0.04309 | 5.527131783 | 30.54918575 |
| Neuralized-like protein 4 || id:prot-a-2038 | rs7412 | T | C | 19 | 45412079 | -0.2711 | 4.57E-09 | 0.0463 | 0.07776 | -5.855291577 | 34.28443945 |
| Neurensin-1 || id:prot-a-2101 | rs4241818 | C | T | 4 | 187153786 | -0.1843 | 6.92E-14 | 0.0246 | 0.51359 | -7.491869919 | 56.12811488 |
| Neurensin-1 || id:prot-a-2101 | rs1801020 | G | A | 5 | 176836532 | -0.1625 | 1.00E-08 | 0.0283 | 0.75032 | -5.74204947 | 32.97113212 |
| Neurensin-1 || id:prot-a-2101 | rs704 | A | G | 17 | 26694861 | 0.2127 | 2.95E-18 | 0.0244 | 0.46665 | 8.717213115 | 75.98980449 |
| Neurexin-1 || id:prot-a-2103 | rs4253272 | T | C | 4 | 187163614 | -0.3002 | 8.51E-35 | 0.0244 | 0.51236 | -12.30327869 | 151.3706665 |
| Neurexin-1 || id:prot-a-2103 | rs2731673 | C | T | 5 | 176839898 | -0.241 | 6.17E-18 | 0.0279 | 0.75237 | -8.637992832 | 74.61492016 |
| Neurexin-2-beta || id:prot-a-2104 | rs57330047 | C | A | 16 | 84358139 | -0.1617 | 2.82E-08 | 0.0291 | 0.24151 | -5.556701031 | 30.87692635 |
| Neurexin-2-beta || id:prot-a-2104 | rs13469 | T | C | 17 | 26676135 | 0.1457 | 2.09E-09 | 0.0243 | 0.50763 | 5.995884774 | 35.95063422 |
| Neurexin-3-beta || id:prot-a-2105 | rs3862628 | A | G | 11 | 126242912 | -0.1849 | 7.76E-10 | 0.0301 | 0.23041 | -6.142857143 | 37.73469388 |
| Neurexin-3-beta || id:prot-a-2105 | rs7149771 | A | G | 14 | 79529757 | 0.1664 | 4.07E-11 | 0.0252 | 0.62387 | 6.603174603 | 43.60191484 |
| Neurexin-3-beta || id:prot-a-2106 | rs13402560 | G | C | 2 | 3639915 | -0.2472 | 4.07E-14 | 0.0327 | 0.81367 | -7.559633028 | 57.14805151 |
| Neurexin-3-beta || id:prot-a-2106 | rs76801637 | A | G | 19 | 58788784 | 0.6427 | 3.63E-09 | 0.1089 | 0.01328 | 5.90174472 | 34.83059074 |
| Neurexin-3-beta || id:prot-a-2107 | rs74480769 | G | A | 5 | 40972211 | -0.4615 | 3.09E-10 | 0.0733 | 0.0317 | -6.296043656 | 39.64016572 |
| Neurexophilin-1 || id:prot-a-2132 | rs3216676 | G | GA | 1 | 57404564 | -0.4801 | 6.03E-81 | 0.0252 | 0.67654 | -19.0515873 | 362.9629787 |
| Neurexophilin-1 || id:prot-a-2132 | rs12092641 | C | T | 1 | 57426950 | 0.4848 | 2.82E-18 | 0.0556 | 0.05228 | 8.71942446 | 76.02836292 |
| Neurexophilin-3 || id:prot-a-2133 | rs964184 | C | G | 11 | 116648917 | -0.2169 | 2.63E-09 | 0.0364 | 0.86898 | -5.958791209 | 35.50719267 |
| Neuroendocrine convertase 1 || id:prot-a-2211 | rs13169290 | A | G | 5 | 95729406 | -1.0976 | 1.00E-200 | 0.0209 | 0.26011 | -52.51674641 | 2758.008654 |
| Neuroendocrine convertase 1 || id:prot-a-2211 | rs12523440 | T | C | 5 | 95155596 | -0.3642 | 1.95E-11 | 0.0543 | 0.0559 | -6.70718232 | 44.98629468 |
| Neuroendocrine convertase 1 || id:prot-a-2211 | rs5869723 | A | ATATTGTTCCC | 5 | 95850990 | 0.1673 | 1.23E-09 | 0.0275 | 0.44011 | 6.083636364 | 37.0106314 |
| Neuroepithelial cell-transforming gene 1 protein || id:prot-a-2034 | rs12487289 | T | C | 3 | 55161785 | 0.2973 | 2.14E-08 | 0.0531 | 0.0723 | 5.598870056 | 31.34734591 |
| Neurofascin || id:prot-a-2039 | rs11801063 | C | T | 1 | 204809143 | 0.5238 | 1.15E-34 | 0.0427 | 0.08497 | 12.26697892 | 150.4787719 |
| Neurofascin || id:prot-a-2039 | rs6667532 | G | A | 1 | 204948659 | 0.8226 | 4.07E-100 | 0.0387 | 0.10515 | 21.25581395 | 451.8096268 |
| Neurofascin || id:prot-a-2039 | rs72755537 | A | C | 1 | 204977727 | 0.3692 | 2.14E-15 | 0.0465 | 0.07992 | 7.939784946 | 63.04018499 |
| Neurofascin || id:prot-a-2039 | rs2497833 | T | A | 10 | 18271970 | -0.1722 | 6.92E-12 | 0.0251 | 0.47911 | -6.860557769 | 47.0672529 |
| Neurogenic locus notch homolog protein 1 || id:prot-a-2067 | rs8176743 | T | C | 9 | 136131415 | 0.3553 | 4.17E-13 | 0.049 | 0.06688 | 7.251020408 | 52.57729696 |
| Neuron-specific protein family member 1 || id:prot-a-2110 | rs16840716 | G | A | 4 | 7621490 | 0.2736 | 6.92E-09 | 0.0472 | 0.07504 | 5.796610169 | 33.60068946 |
| Neuron-specific protein family member 2 || id:prot-a-2111 | rs6993770 | T | A | 8 | 106581528 | -0.1901 | 3.55E-12 | 0.0273 | 0.28225 | -6.963369963 | 48.48852125 |
| Neuronal growth regulator 1 || id:prot-a-2028 | rs6699841 | A | G | 1 | 72645850 | -0.1351 | 3.89E-08 | 0.0246 | 0.51787 | -5.491869919 | 30.1606352 |
| Neuronal growth regulator 1 || id:prot-a-2029 | rs2126077 | T | A | 1 | 72712607 | 0.1375 | 3.24E-08 | 0.0249 | 0.46822 | 5.522088353 | 30.49345978 |
| Neuronal pentraxin-1 || id:prot-a-2080 | rs62069681 | C | T | 17 | 78624702 | 0.514 | 7.59E-35 | 0.0417 | 0.09319 | 12.32613909 | 151.9337048 |
| Neuronal pentraxin-2 || id:prot-a-2081 | rs9287090 | A | G | 1 | 169510380 | 0.2126 | 3.39E-14 | 0.028 | 0.26341 | 7.592857143 | 57.65147959 |
| Neuronal pentraxin-2 || id:prot-a-2081 | rs5112 | G | C | 19 | 45430280 | 0.1818 | 1.07E-11 | 0.0268 | 0.53475 | 6.78358209 | 46.01698597 |
| Neuropeptide S || id:prot-a-2079 | rs150566898 | T | C | 5 | 119010356 | -0.3042 | 4.07E-08 | 0.0554 | 0.05726 | -5.490974729 | 30.15080348 |
| Neuropeptide S || id:prot-a-2079 | rs11057102 | T | G | 12 | 17083997 | 0.1539 | 5.62E-09 | 0.0264 | 0.37497 | 5.829545455 | 33.98360021 |
| Neuropeptide W || id:prot-a-2082 | rs4253311 | G | A | 4 | 187174683 | -0.2284 | 2.24E-20 | 0.0247 | 0.51063 | -9.246963563 | 85.50633513 |
| Neuropeptide W || id:prot-a-2082 | rs2731673 | C | T | 5 | 176839898 | -0.1754 | 4.17E-10 | 0.0281 | 0.75237 | -6.241992883 | 38.96247515 |
| Neuropeptide W || id:prot-a-2082 | rs35327014 | G | A | 16 | 2076202 | -0.4266 | 1.62E-49 | 0.0288 | 0.23117 | -14.8125 | 219.4101563 |
| Neuropilin-1 || id:prot-a-2099 | rs10864728 | G | A | 1 | 230304914 | 0.1892 | 5.37E-14 | 0.0252 | 0.60091 | 7.507936508 | 56.36911061 |
| Neuropilin-1 || id:prot-a-2099 | rs2506149 | T | C | 10 | 33480713 | -0.2805 | 6.92E-28 | 0.0256 | 0.35807 | -10.95703125 | 120.0565338 |
| Neuropilin-2 || id:prot-a-2100 | rs16837641 | A | G | 2 | 206634869 | 0.2094 | 1.07E-14 | 0.0271 | 0.32186 | 7.726937269 | 59.70555956 |
| Neuropilin-2 || id:prot-a-2100 | rs12820248 | A | C | 12 | 26460521 | 0.1537 | 3.63E-08 | 0.0279 | 0.27033 | 5.508960573 | 30.3486466 |
| Neurotrophin-3 || id:prot-a-2114 | rs116414447 | C | A | 2 | 100189713 | 0.2995 | 3.39E-08 | 0.0542 | 0.05808 | 5.525830258 | 30.53480004 |
| Neurotrophin-4 || id:prot-a-2115 | rs10418046 | G | T | 19 | 54327869 | -0.1648 | 3.31E-08 | 0.0298 | 0.21727 | -5.530201342 | 30.58312689 |
| Neutral ceramidase || id:prot-a-179 | rs1369862 | G | A | 10 | 52006008 | -0.2894 | 2.95E-30 | 0.0253 | 0.3857 | -11.43873518 | 130.8446625 |
| Neutral ceramidase || id:prot-a-179 | rs10740617 | C | A | 10 | 52027609 | 0.6665 | 2.00E-124 | 0.0281 | 0.79354 | 23.71886121 | 562.5843771 |
| Neutrophil-activating peptide 2 || id:prot-a-2337 | rs861442 | G | A | 2 | 224902897 | -0.1775 | 3.09E-12 | 0.0255 | 0.64065 | -6.960784314 | 48.45251826 |
| Neutrophil-activating peptide 2 || id:prot-a-2337 | rs6993770 | T | A | 8 | 106581528 | -0.2029 | 1.07E-13 | 0.0273 | 0.28225 | -7.432234432 | 55.23810866 |
| Neutrophil-activating peptide 2 || id:prot-a-2337 | rs7178458 | C | T | 15 | 101991773 | -0.1548 | 1.82E-08 | 0.0275 | 0.7153 | -5.629090909 | 31.68666446 |
| Neutrophil collagenase || id:prot-a-1920 | rs371346021 | G | GAC | 6 | 31221935 | 0.4233 | 5.37E-23 | 0.0429 | 0.08743 | 9.867132867 | 97.36031102 |
| Neutrophil collagenase || id:prot-a-1920 | rs12614 | T | C | 6 | 31914179 | 1.3264 | 1.00E-200 | 0.0368 | 0.08711 | 36.04347826 | 1299.132325 |
| Neutrophil collagenase || id:prot-a-1920 | rs3828785 | A | G | 6 | 32628907 | 0.9359 | 6.31E-12 | 0.1362 | 0.00788 | 6.871512482 | 47.21768379 |
| Neutrophil collagenase || id:prot-a-1920 | rs78237972 | C | T | 9 | 135055836 | 0.4619 | 2.14E-08 | 0.0825 | 0.02503 | 5.598787879 | 31.34642571 |
| Neutrophil collagenase || id:prot-a-1920 | rs61751507 | T | C | 10 | 101829514 | -0.7764 | 3.16E-40 | 0.0585 | 0.04472 | -13.27179487 | 176.1405391 |
| Neutrophil collagenase || id:prot-a-1920 | rs704 | A | G | 17 | 26694861 | -0.2151 | 1.23E-18 | 0.0244 | 0.46665 | -8.81557377 | 77.7143409 |
| Neutrophil cytosol factor 2 || id:prot-a-2009 | rs17580 | A | T | 14 | 94847262 | 0.6643 | 1.32E-32 | 0.0559 | 0.04977 | 11.88372093 | 141.2228231 |
| Neutrophil cytosol factor 2 || id:prot-a-2009 | rs28929474 | T | C | 14 | 94844947 | 1.0455 | 3.16E-37 | 0.082 | 0.02269 | 12.75 | 162.5625 |
| Neutrophil cytosol factor 2 || id:prot-a-2009 | rs4632248 | T | G | 19 | 54324995 | 0.653 | 1.15E-121 | 0.0278 | 0.21439 | 23.48920863 | 551.7429222 |
| Neutrophil gelatinase-associated lipocalin || id:prot-a-1709 | rs59925331 | G | C | 17 | 38169273 | 0.1533 | 2.40E-09 | 0.0257 | 0.38407 | 5.964980545 | 35.5809929 |
| Next to BRCA1 gene 1 protein || id:prot-a-2004 | rs62143197 | A | G | 19 | 54320716 | 0.3082 | 9.55E-25 | 0.03 | 0.22311 | 10.27333333 | 105.5413778 |
| NHL repeat-containing protein 3 || id:prot-a-2047 | rs3917532 | T | A | 7 | 94940119 | 0.3568 | 4.37E-40 | 0.0269 | 0.28253 | 13.26394052 | 175.9321181 |
| NHP2-like protein 1 || id:prot-a-2048 | rs4632248 | T | G | 19 | 54324995 | 0.7014 | 1.23E-143 | 0.0275 | 0.21439 | 25.50545455 | 650.5282116 |
| Nicotinamide phosphoribosyltransferase || id:prot-a-1998 | rs4632248 | T | G | 19 | 54324995 | 0.4632 | 1.62E-57 | 0.029 | 0.21439 | 15.97241379 | 255.1180024 |
| Nidogen-1 || id:prot-a-2049 | rs72765476 | T | C | 1 | 236230636 | -0.7777 | 3.63E-08 | 0.1412 | 0.00941 | -5.507790368 | 30.33575474 |
| Nidogen-1 || id:prot-a-2049 | rs927826 | T | G | 10 | 20220846 | 0.1786 | 5.37E-11 | 0.0272 | 0.70585 | 6.566176471 | 43.11467344 |
| Nidogen-1 || id:prot-a-2049 | rs1844915 | T | A | 12 | 125950020 | 0.2821 | 2.57E-08 | 0.0507 | 0.06323 | 5.564102564 | 30.95923734 |
| Nidogen-2 || id:prot-a-2050 | rs112838462 | A | AT | 10 | 65214017 | 0.1671 | 1.58E-09 | 0.0277 | 0.6819 | 6.032490975 | 36.39094736 |
| Nidogen-2 || id:prot-a-2050 | rs1151582 | T | C | 14 | 52482768 | -0.3942 | 2.57E-64 | 0.0233 | 0.45663 | -16.91845494 | 286.2341174 |
| Nischarin || id:prot-a-2052 | rs9856575 | A | G | 3 | 52510592 | 0.4603 | 2.34E-08 | 0.0824 | 0.97626 | 5.586165049 | 31.20523995 |
| Nischarin || id:prot-a-2052 | rs10418046 | G | T | 19 | 54327869 | 0.3406 | 4.57E-31 | 0.0294 | 0.21727 | 11.58503401 | 134.2130131 |
| NKG2-D type II integral membrane protein || id:prot-a-1675 | rs2617149 | C | G | 12 | 10528333 | -0.2001 | 2.04E-13 | 0.0272 | 0.71841 | -7.356617647 | 54.11982321 |
| NKG2-E type II integral membrane protein || id:prot-a-1670 | rs12085435 | A | G | 1 | 57415310 | 0.6312 | 1.17E-30 | 0.0548 | 0.05166 | 11.51824818 | 132.670041 |
| NKG2-E type II integral membrane protein || id:prot-a-1670 | rs704 | A | G | 17 | 26694861 | 0.2888 | 6.61E-33 | 0.0242 | 0.46665 | 11.9338843 | 142.4175944 |
| NKG2-E type II integral membrane protein || id:prot-a-1671 | rs2242572 | A | G | 6 | 31910929 | 0.2395 | 1.15E-08 | 0.042 | 0.09382 | 5.702380952 | 32.51714853 |
| NKG2-E type II integral membrane protein || id:prot-a-1671 | rs112689088 | C | T | 17 | 34307457 | -0.6711 | 1.17E-59 | 0.0412 | 0.09753 | -16.28883495 | 265.3261441 |
| NKG2D ligand 1 || id:prot-a-3159 | rs12147435 | A | G | 14 | 106935395 | -0.1862 | 3.39E-08 | 0.0337 | 0.17826 | -5.525222552 | 30.52808425 |
| NKG2D ligand 4 || id:prot-a-2487 | rs1926447 | G | A | 13 | 46629944 | -0.4253 | 4.68E-58 | 0.0265 | 0.71131 | -16.0490566 | 257.5722179 |
| NmrA-like family domain-containing protein 1 || id:prot-a-2062 | rs11557236 | A | G | 16 | 4519439 | -0.4905 | 1.55E-28 | 0.0443 | 0.0821 | -11.07223476 | 122.5943826 |
| NmrA-like family domain-containing protein 1 || id:prot-a-2062 | rs62143197 | A | G | 19 | 54320716 | 0.2205 | 3.02E-13 | 0.0302 | 0.22311 | 7.301324503 | 53.3093395 |
| Noggin || id:prot-a-2064 | rs114694170 | C | T | 5 | 88180196 | 0.3383 | 1.78E-10 | 0.053 | 0.06212 | 6.383018868 | 40.74292987 |
| Noggin || id:prot-a-2064 | rs34377578 | C | A | 10 | 104336426 | 0.1627 | 7.59E-09 | 0.0282 | 0.25966 | 5.769503546 | 33.28717117 |
| Noggin || id:prot-a-2064 | rs79084672 | G | A | 17 | 54856140 | 1.2369 | 8.71E-27 | 0.1154 | 0.01187 | 10.71837088 | 114.8834744 |
| Non-histone chromosomal protein HMG-14 || id:prot-a-1357 | rs1042445 | T | C | 3 | 186395436 | -0.681 | 1.07E-131 | 0.0279 | 0.21507 | -24.40860215 | 595.7798589 |
| Non-histone chromosomal protein HMG-14 || id:prot-a-1357 | rs10424405 | G | A | 19 | 54321933 | 0.2871 | 8.91E-22 | 0.0299 | 0.2203 | 9.602006689 | 92.19853245 |
| Non-receptor tyrosine-protein kinase TYK2 || id:prot-a-3127 | rs55924146 | A | T | 1 | 237402000 | -0.147 | 4.90E-08 | 0.0269 | 0.29994 | -5.464684015 | 29.86277138 |
| Non-receptor tyrosine-protein kinase TYK2 || id:prot-a-3127 | rs967645 | T | C | 17 | 26713970 | -0.1347 | 3.39E-08 | 0.0244 | 0.50945 | -5.520491803 | 30.47582975 |
| Non-secretory ribonuclease || id:prot-a-2555 | rs56204594 | C | A | 14 | 21433367 | 0.2366 | 3.16E-22 | 0.0244 | 0.46429 | 9.696721311 | 94.02640419 |
| Normal mucosa of esophagus-specific gene 1 protein || id:prot-a-2061 | rs2286798 | C | A | 3 | 52821177 | 0.2769 | 9.55E-28 | 0.0254 | 0.36185 | 10.9015748 | 118.8443332 |
| Normal mucosa of esophagus-specific gene 1 protein || id:prot-a-2061 | rs10964730 | G | A | 9 | 20826029 | 0.1912 | 4.27E-08 | 0.0349 | 0.1505 | 5.478510029 | 30.01407213 |
| Normal mucosa of esophagus-specific gene 1 protein || id:prot-a-2061 | rs10418046 | G | T | 19 | 54327869 | 0.3681 | 3.09E-36 | 0.0293 | 0.21727 | 12.56313993 | 157.8324849 |
| NTF2-related export protein 1 || id:prot-a-2134 | rs1042464 | T | A | 3 | 186395572 | 0.2352 | 7.08E-22 | 0.0245 | 0.47358 | 9.6 | 92.16 |
| NTF2-related export protein 1 || id:prot-a-2134 | rs10424405 | G | A | 19 | 54321933 | 0.4849 | 4.07E-62 | 0.0292 | 0.2203 | 16.60616438 | 275.7646955 |
| Nuclear factor erythroid 2-related factor 1 || id:prot-a-2040 | rs62143197 | A | G | 19 | 54320716 | 0.315 | 7.94E-26 | 0.03 | 0.22311 | 10.5 | 110.25 |
| Nuclear migration protein nudC || id:prot-a-2125 | rs10418046 | G | T | 19 | 54327869 | 0.4125 | 1.38E-45 | 0.0291 | 0.21727 | 14.17525773 | 200.9379318 |
| Nuclear pore complex-interacting protein family member B3 || id:prot-a-2074 | rs570618 | G | T | 1 | 196657064 | -0.1682 | 3.02E-11 | 0.0253 | 0.61149 | -6.648221344 | 44.19884704 |
| Nuclear pore complex-interacting protein family member B3 || id:prot-a-2074 | rs704 | A | G | 17 | 26694861 | 0.3099 | 7.41E-38 | 0.0241 | 0.46665 | 12.85892116 | 165.3518534 |
| Nuclear protein localization protein 4 homolog || id:prot-a-2075 | rs62143197 | A | G | 19 | 54320716 | 0.2087 | 5.25E-12 | 0.0303 | 0.22311 | 6.887788779 | 47.44163426 |
| Nuclear receptor-binding protein || id:prot-a-2093 | rs1065853 | T | G | 19 | 45413233 | -0.4854 | 2.45E-26 | 0.0457 | 0.07778 | -10.6214442 | 112.8150769 |
| Nuclear receptor coactivator 2 || id:prot-a-2012 | rs72679194 | T | G | 4 | 155473212 | -0.1757 | 2.88E-08 | 0.0317 | 0.18059 | -5.542586751 | 30.72026789 |
| Nuclear receptor coactivator 2 || id:prot-a-2012 | rs113358888 | T | TA | 14 | 106920387 | -0.266 | 2.45E-15 | 0.0336 | 0.77974 | -7.916666667 | 62.67361111 |
| Nuclear receptor subfamily 1 group D member 2 || id:prot-a-2086 | rs17863787 | G | T | 2 | 234611094 | 0.1875 | 3.24E-12 | 0.0269 | 0.306 | 6.970260223 | 48.58452758 |
| Nuclear RNA export factor 1 || id:prot-a-2131 | rs41272307 | A | G | 5 | 52386315 | 0.5685 | 4.07E-08 | 0.1036 | 0.01456 | 5.487451737 | 30.11212657 |
| Nuclear RNA export factor 1 || id:prot-a-2131 | rs10418046 | G | T | 19 | 54327869 | 0.584 | 3.09E-95 | 0.0282 | 0.21727 | 20.70921986 | 428.8717871 |
| Nucleolin || id:prot-a-2010 | rs10424405 | G | A | 19 | 54321933 | 0.241 | 1.07E-15 | 0.0301 | 0.2203 | 8.006644518 | 64.10635644 |
| Nucleoside diphosphate-linked moiety X motif 8, mitochondrial || id:prot-a-2128 | rs74480769 | G | A | 5 | 40972211 | -0.4736 | 1.05E-10 | 0.0733 | 0.0317 | -6.46111869 | 41.74605473 |
| Nucleoside diphosphate kinase A || id:prot-a-2059 | rs6441996 | C | G | 3 | 46505266 | -0.331 | 2.40E-30 | 0.0289 | 0.23776 | -11.4532872 | 131.1777876 |
| Nucleoside diphosphate kinase B || id:prot-a-2060 | rs6504695 | G | A | 17 | 49212866 | -0.1598 | 6.76E-11 | 0.0245 | 0.49071 | -6.52244898 | 42.54234069 |
| Nucleoside diphosphate kinase B || id:prot-a-2060 | rs10418046 | G | T | 19 | 54327869 | 0.3315 | 1.95E-29 | 0.0294 | 0.21727 | 11.2755102 | 127.1371304 |
| Nucleosome assembly protein 1-like 2 || id:prot-a-1999 | rs73165061 | A | G | 3 | 165481945 | 0.1881 | 1.23E-09 | 0.031 | 0.20247 | 6.067741935 | 36.8174922 |
| Nucleosome assembly protein 1-like 2 || id:prot-a-1999 | rs704 | A | G | 17 | 26694861 | 0.2465 | 3.80E-24 | 0.0243 | 0.46665 | 10.14403292 | 102.9014039 |
| Nutritionally-regulated adipose and cardiac enriched protein homolog || id:prot-a-2092 | rs1542819 | C | T | 7 | 45978059 | 0.1898 | 1.55E-14 | 0.0247 | 0.46737 | 7.684210526 | 59.04709141 |
| O-acetyl-ADP-ribose deacetylase MACROD1 || id:prot-a-1824 | rs4795433 | T | C | 17 | 26716821 | 0.1544 | 2.51E-10 | 0.0244 | 0.50963 | 6.327868852 | 40.04192421 |
| Obg-like ATPase 1 || id:prot-a-2143 | rs145495932 | T | C | 2 | 175079756 | 0.1967 | 5.37E-10 | 0.0317 | 0.19196 | 6.205047319 | 38.50261223 |
| Obg-like ATPase 1 || id:prot-a-2143 | rs62143197 | A | G | 19 | 54320716 | 0.2116 | 2.63E-12 | 0.0303 | 0.22311 | 6.98349835 | 48.7692492 |
| OCIA domain-containing protein 1 || id:prot-a-2140 | rs6934384 | T | C | 6 | 135613187 | -0.3785 | 1.12E-08 | 0.0663 | 0.03452 | -5.708898944 | 32.59152716 |
| OCIA domain-containing protein 1 || id:prot-a-2140 | rs7212510 | A | T | 17 | 26703682 | -0.1348 | 3.39E-08 | 0.0244 | 0.50911 | -5.524590164 | 30.52109648 |
| Odorant-binding protein 2a || id:prot-a-2138 | rs6993770 | T | A | 8 | 106581528 | -0.1557 | 1.32E-08 | 0.0274 | 0.28225 | -5.682481752 | 32.29059886 |
| Odorant-binding protein 2b || id:prot-a-2139 | rs697449 | T | G | 9 | 138467959 | 0.799 | 1.70E-172 | 0.0285 | 0.18891 | 28.03508772 | 785.9661434 |
| Odorant-binding protein 2b || id:prot-a-2139 | rs4454354 | C | T | 9 | 136089529 | 0.358 | 2.69E-33 | 0.0298 | 0.78613 | 12.01342282 | 144.3223278 |
| Odorant-binding protein 2b || id:prot-a-2139 | rs2590499 | G | A | 9 | 138440202 | -0.4019 | 2.34E-48 | 0.0275 | 0.71684 | -14.61454545 | 213.5849388 |
| Olfactomedin-like protein 3 || id:prot-a-2145 | rs62143198 | A | G | 19 | 54320939 | -0.1783 | 5.50E-09 | 0.0306 | 0.21453 | -5.826797386 | 33.95156777 |
| Oligoribonuclease, mitochondrial || id:prot-a-2526 | rs945456 | G | C | 14 | 95688959 | -0.1434 | 1.45E-08 | 0.0253 | 0.44275 | -5.66798419 | 32.12604477 |
| Oncostatin-M-specific receptor subunit beta || id:prot-a-2156 | rs357253 | T | C | 5 | 38907422 | 0.2089 | 8.32E-13 | 0.0292 | 0.23185 | 7.154109589 | 51.18128401 |
| Oncostatin-M || id:prot-a-2155 | rs570618 | G | T | 1 | 196657064 | -0.1732 | 7.59E-12 | 0.0253 | 0.61149 | -6.845849802 | 46.86565952 |
| Oncostatin-M || id:prot-a-2155 | rs74480769 | G | A | 5 | 40972211 | -0.5281 | 5.37E-13 | 0.0732 | 0.0317 | -7.214480874 | 52.04873429 |
| Opalin || id:prot-a-2148 | rs704 | A | G | 17 | 26694861 | -0.1935 | 2.57E-15 | 0.0245 | 0.46665 | -7.897959184 | 62.37775927 |
| Opalin || id:prot-a-2148 | rs59993071 | C | A | 22 | 16912487 | -0.5438 | 2.24E-08 | 0.0973 | 0.01852 | -5.588900308 | 31.23580666 |
| Opioid-binding protein/cell adhesion molecule || id:prot-a-2149 | rs3032928 | CTTT | C | 17 | 26679563 | -0.1393 | 2.29E-08 | 0.0249 | 0.45639 | -5.59437751 | 31.29705972 |
| Origin recognition complex subunit 6 || id:prot-a-2151 | rs3775298 | G | A | 4 | 187150478 | 0.1367 | 3.24E-08 | 0.0247 | 0.51335 | 5.534412955 | 30.62972676 |
| Origin recognition complex subunit 6 || id:prot-a-2151 | rs2731674 | G | T | 5 | 176839890 | 0.155 | 3.80E-08 | 0.0282 | 0.75301 | 5.496453901 | 30.21100548 |
| Ornithine decarboxylase || id:prot-a-2142 | rs378528 | C | T | 21 | 44452455 | 0.1707 | 3.39E-08 | 0.0309 | 0.79033 | 5.524271845 | 30.51757941 |
| Osteocalcin || id:prot-a-246 | rs71631868 | C | T | 1 | 196815711 | 0.1677 | 4.27E-08 | 0.0306 | 0.20641 | 5.480392157 | 30.03469819 |
| Osteocalcin || id:prot-a-246 | rs2526393 | G | C | 3 | 50189173 | 0.2234 | 3.39E-08 | 0.0405 | 0.89302 | 5.516049383 | 30.42680079 |
| Osteocalcin || id:prot-a-246 | rs9271374 | A | G | 6 | 32587041 | 0.2069 | 4.68E-11 | 0.0314 | 0.81128 | 6.589171975 | 43.41718731 |
| Osteocalcin || id:prot-a-246 | rs62143194 | G | C | 19 | 54319624 | 0.2574 | 2.69E-17 | 0.0304 | 0.22364 | 8.467105263 | 71.69187154 |
| Osteoclast-associated immunoglobulin-like receptor || id:prot-a-2153 | rs9526310 | C | T | 13 | 47891517 | 0.486 | 1.78E-09 | 0.0808 | 0.02409 | 6.014851485 | 36.17843839 |
| Out at first protein homolog || id:prot-a-2135 | rs117554512 | T | C | 11 | 120098329 | 0.6816 | 2.14E-56 | 0.0431 | 0.08626 | 15.81438515 | 250.0947777 |
| Out at first protein homolog || id:prot-a-2135 | rs2444239 | T | A | 11 | 120056416 | 0.3699 | 3.47E-52 | 0.0243 | 0.56183 | 15.22222222 | 231.7160494 |
| OX-2 membrane glycoprotein || id:prot-a-422 | rs635634 | T | C | 9 | 136155000 | -0.1893 | 3.98E-09 | 0.0322 | 0.17981 | -5.878881988 | 34.56125342 |
| Oxidized low-density lipoprotein receptor 1 || id:prot-a-2146 | rs62143206 | T | G | 19 | 54326212 | 0.1912 | 1.55E-10 | 0.0299 | 0.2131 | 6.394648829 | 40.89153365 |
| Oxidoreductase HTATIP2 || id:prot-a-1389 | rs74371675 | T | C | 9 | 29580118 | -0.6418 | 4.68E-08 | 0.1174 | 0.01225 | -5.466780239 | 29.88568618 |
| Oxidoreductase HTATIP2 || id:prot-a-1389 | rs10437608 | A | G | 11 | 20385606 | 0.1453 | 5.89E-09 | 0.025 | 0.55596 | 5.812 | 33.779344 |
| Oxidoreductase HTATIP2 || id:prot-a-1389 | rs62143198 | A | G | 19 | 54320939 | 0.2882 | 2.00E-21 | 0.0303 | 0.21453 | 9.511551155 | 90.46960538 |
| Oxidoreductase HTATIP2 || id:prot-a-1390 | rs2127870 | C | G | 14 | 65796846 | -0.6699 | 2.51E-123 | 0.0284 | 0.78457 | -23.58802817 | 556.3950729 |
| Oxysterol-binding protein-related protein 11 || id:prot-a-2152 | rs11575194 | A | G | 2 | 217543728 | 0.415 | 8.71E-12 | 0.0608 | 0.04484 | 6.825657895 | 46.5896057 |
| Oxysterol-binding protein-related protein 11 || id:prot-a-2152 | rs112597211 | C | T | 14 | 106369433 | -0.2065 | 1.45E-08 | 0.0364 | 0.14002 | -5.673076923 | 32.18380178 |
| Oxysterols receptor LXR-beta || id:prot-a-2087 | rs7539005 | T | A | 1 | 196667252 | -0.157 | 6.76E-10 | 0.0255 | 0.61028 | -6.156862745 | 37.90695886 |
| Oxysterols receptor LXR-beta || id:prot-a-2087 | rs74480769 | G | A | 5 | 40972211 | -0.4961 | 1.26E-11 | 0.0733 | 0.0317 | -6.768076398 | 45.80685813 |
| Oxysterols receptor LXR-beta || id:prot-a-2087 | rs704 | A | G | 17 | 26694861 | 0.3385 | 2.88E-45 | 0.024 | 0.46665 | 14.10416667 | 198.9275174 |
| Oxytocin-neurophysin 1 || id:prot-a-2159 | rs704 | A | G | 17 | 26694861 | 0.1518 | 6.31E-10 | 0.0246 | 0.46665 | 6.170731707 | 38.0779298 |
| P-selectin || id:prot-a-2667 | rs6019 | G | C | 1 | 169541513 | -0.3417 | 1.35E-10 | 0.0532 | 0.05782 | -6.422932331 | 41.25405973 |
| P-selectin || id:prot-a-2667 | rs74227709 | A | G | 1 | 247722588 | 0.3465 | 1.20E-13 | 0.0467 | 0.07125 | 7.419700214 | 55.05195127 |
| P-selectin || id:prot-a-2667 | rs6136 | G | T | 1 | 169563951 | -0.8073 | 3.02E-105 | 0.0371 | 0.10788 | -21.76010782 | 473.5022922 |
| P-selectin || id:prot-a-2667 | rs9820851 | A | G | 3 | 72366285 | 0.176 | 1.86E-08 | 0.0313 | 0.20096 | 5.623003195 | 31.61816493 |
| P-selectin || id:prot-a-2667 | rs6993770 | T | A | 8 | 106581528 | -0.1788 | 6.31E-11 | 0.0274 | 0.28225 | -6.525547445 | 42.58276946 |
| P-selectin || id:prot-a-2667 | rs2519093 | T | C | 9 | 136141870 | -0.4555 | 4.27E-48 | 0.0313 | 0.17823 | -14.55271565 | 211.7815329 |
| P-selectin glycoprotein ligand 1 || id:prot-a-2668 | rs35381129 | T | C | 1 | 76482025 | -0.1937 | 9.77E-10 | 0.0317 | 0.20082 | -6.110410095 | 37.33711152 |
| P2X purinoceptor 6 || id:prot-a-2160 | rs11150417 | C | T | 16 | 81881952 | -0.1687 | 2.88E-08 | 0.0304 | 0.2208 | -5.549342105 | 30.7951978 |
| p53 and DNA damage-regulated protein 1 || id:prot-a-2238 | rs10424405 | G | A | 19 | 54321933 | 0.2608 | 3.72E-18 | 0.03 | 0.2203 | 8.693333333 | 75.57404444 |
| Paired immunoglobulin-like type 2 receptor alpha || id:prot-a-2272 | rs1048372 | C | T | 6 | 32610436 | 0.1687 | 3.31E-11 | 0.0254 | 0.60918 | 6.641732283 | 44.11260773 |
| Paired immunoglobulin-like type 2 receptor alpha || id:prot-a-2272 | rs11624464 | A | G | 14 | 106215480 | -0.2298 | 1.17E-17 | 0.0269 | 0.65429 | -8.542750929 | 72.97859344 |
| Palmitoyl-protein thioesterase 1 || id:prot-a-2351 | rs4308964 | T | C | 1 | 40444888 | -0.2071 | 1.23E-11 | 0.0306 | 0.71215 | -6.767973856 | 45.80547012 |
| Palmitoyl-protein thioesterase 1 || id:prot-a-2351 | rs7533094 | A | G | 1 | 40559686 | -0.8621 | 2.29E-46 | 0.0603 | 0.04198 | -14.29684909 | 204.3998938 |
| Palmitoyl-protein thioesterase 1 || id:prot-a-2351 | rs73165061 | A | G | 3 | 165481945 | 0.2693 | 2.14E-18 | 0.0308 | 0.20247 | 8.743506494 | 76.4489058 |
| Palmitoyl-protein thioesterase 1 || id:prot-a-2351 | rs3957147 | T | C | 6 | 32682135 | 0.2264 | 1.70E-11 | 0.0336 | 0.17073 | 6.738095238 | 45.40192744 |
| Palmitoyl-protein thioesterase 1 || id:prot-a-2351 | rs202046353 | T | A | 12 | 9119414 | 0.2802 | 5.89E-10 | 0.0452 | 0.08385 | 6.199115044 | 38.42902733 |
| Pancreatic hormone || id:prot-a-2352 | rs73174306 | T | A | 3 | 169194244 | 0.4922 | 2.40E-14 | 0.0645 | 0.03897 | 7.631007752 | 58.23227931 |
| Pancreatic hormone || id:prot-a-2352 | rs2438988 | G | A | 10 | 49370090 | -0.2209 | 1.74E-15 | 0.0278 | 0.64331 | -7.946043165 | 63.13960199 |
| Pantothenate kinase 3 || id:prot-a-2173 | rs7259081 | A | T | 19 | 54339243 | -0.1822 | 6.76E-09 | 0.0314 | 0.75126 | -5.802547771 | 33.66956063 |
| Pappalysin-1 || id:prot-a-2177 | rs4328821 | G | A | 3 | 128316435 | -0.226 | 1.05E-08 | 0.0395 | 0.10985 | -5.721518987 | 32.73577952 |
| Pappalysin-1 || id:prot-a-2177 | rs28731213 | A | T | 19 | 4544645 | -0.1556 | 3.98E-10 | 0.0249 | 0.48428 | -6.248995984 | 39.04995081 |
| Parathyroid hormone-related protein || id:prot-a-2432 | rs1973612 | T | C | 4 | 187169167 | -0.2464 | 1.17E-23 | 0.0246 | 0.5115 | -10.01626016 | 100.3254676 |
| Parathyroid hormone-related protein || id:prot-a-2432 | rs2731674 | G | T | 5 | 176839890 | -0.2061 | 2.09E-13 | 0.0281 | 0.75301 | -7.334519573 | 53.79517737 |
| Parathyroid hormone-related protein || id:prot-a-2432 | rs10843115 | T | C | 12 | 28307717 | 0.1862 | 1.45E-11 | 0.0276 | 0.27137 | 6.746376812 | 45.51360008 |
| Parathyroid hormone-related protein || id:prot-a-2432 | rs2231495 | C | T | 22 | 17669306 | 0.1833 | 2.40E-12 | 0.0262 | 0.33252 | 6.996183206 | 48.94657945 |
| Parathyroid hormone/parathyroid hormone-related peptide receptor || id:prot-a-2429 | rs1801690 | G | C | 17 | 64208285 | 0.3261 | 3.39E-09 | 0.0552 | 0.0511 | 5.907608696 | 34.8998405 |
| PAX-interacting protein 1 || id:prot-a-2189 | rs9274954 | T | C | 6 | 32641878 | 0.2158 | 5.13E-13 | 0.0299 | 0.36579 | 7.217391304 | 52.09073724 |
| PAX-interacting protein 1 || id:prot-a-2189 | rs141738059 | C | T | 9 | 116832007 | -0.6493 | 5.50E-09 | 0.1114 | 0.01278 | -5.828545781 | 33.97194592 |
| PAX-interacting protein 1 || id:prot-a-2189 | rs11080055 | C | A | 17 | 26649724 | 0.1682 | 4.17E-12 | 0.0243 | 0.50786 | 6.9218107 | 47.91146336 |
| PDZK1-interacting protein 1 || id:prot-a-2242 | rs429358 | C | T | 19 | 45411941 | 0.3527 | 3.09E-26 | 0.0333 | 0.15301 | 10.59159159 | 112.1818124 |
| Peptidase inhibitor 15 || id:prot-a-2264 | rs7539005 | T | A | 1 | 196667252 | -0.1701 | 2.19E-11 | 0.0254 | 0.61028 | -6.696850394 | 44.8478052 |
| Peptidase inhibitor 15 || id:prot-a-2264 | rs74480769 | G | A | 5 | 40972211 | -0.4379 | 2.40E-09 | 0.0734 | 0.0317 | -5.965940054 | 35.59244073 |
| Peptide chain release factor 1-like, mitochondrial || id:prot-a-1965 | rs147159109 | A | G | 4 | 128692323 | 0.4661 | 4.27E-09 | 0.0794 | 0.02611 | 5.870277078 | 34.46015297 |
| Peptide chain release factor 1-like, mitochondrial || id:prot-a-1965 | rs503366 | C | T | 6 | 153333550 | 0.1652 | 1.15E-11 | 0.0243 | 0.49736 | 6.798353909 | 46.21761588 |
| Peptidoglycan recognition protein 1 || id:prot-a-2257 | rs141114903 | G | C | 6 | 32487814 | -0.165 | 2.57E-08 | 0.0296 | 0.58877 | -5.574324324 | 31.07309167 |
| Peptidoglycan recognition protein 1 || id:prot-a-2257 | rs8102493 | C | T | 19 | 46530389 | -0.2168 | 5.13E-17 | 0.0259 | 0.33338 | -8.370656371 | 70.06788808 |
| Peptidyl-glycine alpha-amidating monooxygenase || id:prot-a-2172 | rs4241818 | C | T | 4 | 187153786 | -0.1829 | 1.07E-13 | 0.0246 | 0.51359 | -7.43495935 | 55.27862053 |
| Peptidyl-glycine alpha-amidating monooxygenase || id:prot-a-2172 | rs115515074 | T | C | 5 | 102310794 | 0.5611 | 3.63E-09 | 0.0951 | 0.01856 | 5.900105152 | 34.81124081 |
| Peptidyl-glycine alpha-amidating monooxygenase || id:prot-a-2172 | rs2731673 | C | T | 5 | 176839898 | -0.1901 | 1.20E-11 | 0.028 | 0.75237 | -6.789285714 | 46.09440051 |
| Peptidyl-glycine alpha-amidating monooxygenase || id:prot-a-2172 | rs148194384 | T | C | 5 | 101961684 | -0.6215 | 4.90E-10 | 0.0999 | 0.01702 | -6.221221221 | 38.70359348 |
| Peptidyl-glycine alpha-amidating monooxygenase || id:prot-a-2172 | rs257309 | G | A | 5 | 102418604 | -0.4664 | 3.80E-79 | 0.0248 | 0.35171 | -18.80645161 | 353.6826223 |
| Peptidyl-glycine alpha-amidating monooxygenase || id:prot-a-2172 | rs1116046 | T | A | 5 | 101578769 | -0.4849 | 9.55E-24 | 0.0483 | 0.07445 | -10.03933747 | 100.7882969 |
| Peptidyl-prolyl cis-trans isomerase-like 1 || id:prot-a-2343 | rs12194408 | G | C | 6 | 36839598 | -1.106 | 1.91E-55 | 0.0705 | 0.03024 | -15.68794326 | 246.1115638 |
| Peptidyl-prolyl cis-trans isomerase-like 1 || id:prot-a-2343 | rs62143196 | G | A | 19 | 54320636 | 0.2592 | 6.76E-18 | 0.0301 | 0.22404 | 8.611295681 | 74.15441331 |
| Peptidyl-prolyl cis-trans isomerase-like 2 || id:prot-a-2344 | rs74480769 | G | A | 5 | 40972211 | -0.4442 | 1.41E-09 | 0.0734 | 0.0317 | -6.051771117 | 36.62393365 |
| Peptidyl-prolyl cis-trans isomerase-like 2 || id:prot-a-2344 | rs704 | A | G | 17 | 26694861 | 0.1723 | 2.09E-12 | 0.0245 | 0.46665 | 7.032653061 | 49.45820908 |
| Peptidyl-prolyl cis-trans isomerase A || id:prot-a-2339 | rs62143198 | A | G | 19 | 54320939 | 0.6335 | 5.13E-108 | 0.0287 | 0.21453 | 22.07317073 | 487.2248662 |
| Peptidyl-prolyl cis-trans isomerase B || id:prot-a-2340 | rs13402560 | G | C | 2 | 3639915 | -0.1969 | 1.95E-09 | 0.0328 | 0.81367 | -6.00304878 | 36.03659466 |
| Peptidyl-prolyl cis-trans isomerase B || id:prot-a-2340 | rs1348079 | C | T | 4 | 68718552 | -0.1386 | 3.89E-08 | 0.0252 | 0.60776 | -5.5 | 30.25 |
| Peptidyl-prolyl cis-trans isomerase E || id:prot-a-2341 | rs12086750 | C | G | 1 | 40210468 | -0.3832 | 4.79E-53 | 0.025 | 0.37062 | -15.328 | 234.947584 |
| Peptidyl-prolyl cis-trans isomerase E || id:prot-a-2341 | rs62143197 | A | G | 19 | 54320716 | 0.2036 | 1.74E-11 | 0.0303 | 0.22311 | 6.719471947 | 45.15130325 |
| Peptidyl-prolyl cis-trans isomerase FKBP14 || id:prot-a-1113 | rs13402561 | G | C | 2 | 3639921 | -0.2768 | 2.69E-17 | 0.0327 | 0.81414 | -8.464831804 | 71.65337747 |
| Peptidyl-prolyl cis-trans isomerase FKBP2 || id:prot-a-1114 | rs967645 | T | C | 17 | 26713970 | -0.1627 | 2.45E-11 | 0.0244 | 0.50945 | -6.668032787 | 44.46266125 |
| Peptidyl-prolyl cis-trans isomerase FKBP7 || id:prot-a-1116 | rs13382684 | C | G | 2 | 179313682 | 0.5569 | 1.00E-70 | 0.0313 | 0.17324 | 17.79233227 | 316.5670875 |
| Peptidyl-prolyl cis-trans isomerase FKBP7 || id:prot-a-1116 | rs12976701 | T | C | 19 | 17563679 | 0.1448 | 2.40E-08 | 0.0259 | 0.38034 | 5.590733591 | 31.25630208 |
| Peptidyl-prolyl cis-trans isomerase H || id:prot-a-2342 | rs62143197 | A | G | 19 | 54320716 | 0.5219 | 5.75E-72 | 0.0291 | 0.22311 | 17.9347079 | 321.6537476 |
| Peptidyl-prolyl cis-trans isomerase NIMA-interacting 4 || id:prot-a-2275 | rs62143206 | T | G | 19 | 54326212 | 0.705 | 2.04E-145 | 0.0275 | 0.2131 | 25.63636364 | 657.2231405 |
| Peregrin || id:prot-a-273 | rs1610041 | C | T | 12 | 122283781 | 0.1446 | 2.34E-08 | 0.0259 | 0.38362 | 5.583011583 | 31.17001834 |
| Periostin || id:prot-a-2332 | rs7981539 | A | C | 13 | 38118276 | 0.1733 | 5.50E-11 | 0.0264 | 0.36765 | 6.564393939 | 43.09126779 |
| Periostin || id:prot-a-2333 | rs9646097 | T | G | 13 | 38196942 | -0.6543 | 1.45E-13 | 0.0885 | 0.02027 | -7.393220339 | 54.65970698 |
| Periostin || id:prot-a-2333 | rs1333352 | C | T | 13 | 38281790 | 0.2504 | 4.27E-08 | 0.0457 | 0.91191 | 5.479212254 | 30.02176692 |
| Periostin || id:prot-a-2333 | rs149549354 | G | A | 16 | 29305173 | -0.3526 | 3.63E-08 | 0.064 | 0.04774 | -5.509375 | 30.35321289 |
| Peroxiredoxin-1 || id:prot-a-2359 | rs61783167 | T | C | 1 | 46238843 | -0.2396 | 7.94E-09 | 0.0415 | 0.09403 | -5.773493976 | 33.33323269 |
| Peroxisomal carnitine O-octanoyltransferase || id:prot-a-669 | rs31644 | C | G | 7 | 87022024 | 0.2534 | 1.78E-10 | 0.0397 | 0.88828 | 6.382871537 | 40.74104905 |
| Peroxisomal NADH pyrophosphatase NUDT12 || id:prot-a-2126 | rs74692061 | G | A | 5 | 102903643 | -0.3756 | 2.75E-28 | 0.0341 | 0.15698 | -11.01466276 | 121.3227956 |
| Peroxisomal targeting signal 1 receptor || id:prot-a-2250 | rs35389082 | C | T | 1 | 199430777 | 0.1628 | 1.66E-08 | 0.0288 | 0.23799 | 5.652777778 | 31.9538966 |
| Persephin || id:prot-a-2423 | rs1993743 | A | C | 1 | 75579863 | -0.259 | 3.16E-08 | 0.0468 | 0.08154 | -5.534188034 | 30.6272372 |
| Persulfide dioxygenase ETHE1, mitochondrial || id:prot-a-992 | rs3775298 | G | A | 4 | 187150478 | -0.1759 | 9.33E-13 | 0.0246 | 0.51335 | -7.150406504 | 51.12831317 |
| Persulfide dioxygenase ETHE1, mitochondrial || id:prot-a-992 | rs2731674 | G | T | 5 | 176839890 | -0.1792 | 1.91E-10 | 0.0281 | 0.75301 | -6.377224199 | 40.66898849 |
| Pescadillo homolog || id:prot-a-2248 | rs142855181 | G | A | 13 | 114795996 | 0.1701 | 1.45E-08 | 0.03 | 0.23163 | 5.67 | 32.1489 |
| PH and SEC7 domain-containing protein 1 || id:prot-a-2398 | rs74480769 | G | A | 5 | 40972211 | -0.4186 | 1.17E-08 | 0.0734 | 0.0317 | -5.702997275 | 32.52417792 |
| PH and SEC7 domain-containing protein 1 || id:prot-a-2398 | rs1303 | G | T | 14 | 94844843 | 0.3571 | 4.90E-39 | 0.0273 | 0.25927 | 13.08058608 | 171.1017322 |
| PH and SEC7 domain-containing protein 1 || id:prot-a-2398 | rs429358 | C | T | 19 | 45411941 | 0.663 | 1.86E-96 | 0.0318 | 0.15301 | 20.8490566 | 434.6831613 |
[truncated: 230,100 more chars]
